# Supplementary material for: RNF26 binds perinuclear vimentin filaments to integrate ER and endolysosomal responses to proteotoxic stress
Source: EMBO J. 2023 Jul 31;42(18):e111252. doi: 10.15252/embj.2022111252 (PMC10505911; doi:10.15252/embj.2022111252)
Supplement: Supplementary file 1 — Expanded View Figures PDF [file EMBJ-42-e111252-s013.pdf]

# Expanded View Figures

**Figure EV1. (Related to Fig 1): RNF26 controls perinuclear ER organization.**

A, B Validation of CRISPR-Cas9-generated RNF26 knockout U2OS cells. (A) DNA sequence analysis of RNF26 5′ CDS region, showing a CRISPR-induced 42 base pair deletion that spans the ATG start codon sequence. (B) WB analysis of RNF26 KO lysates using a validated RNF26 antibody. Vinculin was used as a loading control. MW markers as indicated.

C, D Effect of RNF26 knockout on the intracellular distribution of mCherry-KDEL. (C) Representative confocal z-projections of U2OS parental and RNF26 KO cells ectopically expressing mCherry-KDEL (*white*). Cell and nuclear boundaries are demarcated using dashed and continuous lines, respectively. (D) Quantification of Cherry-KDEL signal distribution expressed as perinuclearity ratio. Plotted are $n_{Parental} = 89$, $n_{RNF26\ KO} = 49$ technical replicates from three independent experiments.

E–H Effect of RNF26 depletion on the intracellular distribution of ER markers. U2OS cells transfected with the indicated siRNAs were fixed and immunostained against endogenous (E) VAP-A or (G) CLIMP63. Representative confocal fluorescence images are shown. Cell and nuclear boundaries are demarcated using dashed and continuous lines, respectively. Quantification of signal distribution as perinuclearity ratio for (F) VAP-A ($n_{siC} = 59$, $n_{siRNF26\#1} = 48$, $n_{siRNF26\#2} = 103$ technical replicates) and (H) CLIMP63 ($n_{control} = 75$, $n_{siRNF26\#2} = 74$ cells, $n_{R26\ KO} = 49$ technical replicates) from three independent experiments.

Data information: Graphs report median (red line) and 95% confidence interval (error bars) of sample values (open circles). Scale bar = 10 μm. Significance was assessed using a Mann–Whitney U; ***$P < 0.001$.

© 2023 The Authors

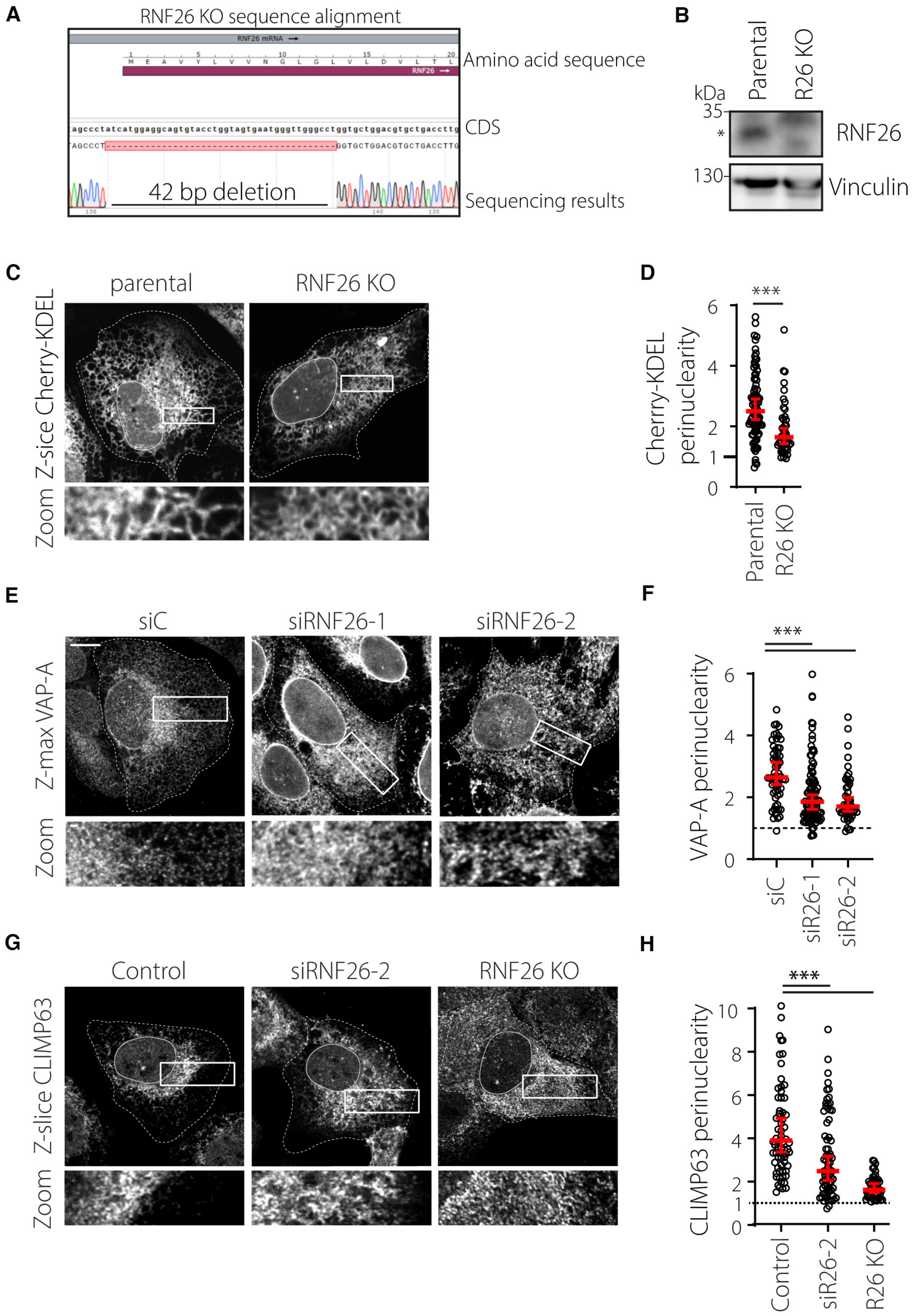


**Figure EV1.**

 © 2023 The Authors

**Figure EV2. (Related to Fig 2): Recombinant RNF26 binds vimentin *in vitro* and identification and synthesis of C-terminal vimentin binding peptide.**

A Identification of interactors of the RNF26 cytoplasmic tail, originally described in and (Fig 2B). Shown is a photograph of the silver-stained SDS–PAGE gel from specific bands were cut out and analyzed by LC–MS. Mass-spec analysis results can be accessed via PRIDE#040760 and correspond to the numbers on this photograph.

B Affinity purification of recombinant RNF26 304–432 fused to a C-terminal GST-3C-Strep tag from *E. coli*. P—pellet, S—soluble fraction, FT—flow-through, E—elution fraction. GST domain was cleaved off using overnight 3C digestion before incubation with NHS-Rhodamine and use in downstream experiments.

C Deposition of RNF26 tail region on assembled vimentin filaments as in (Fig 1H), using GST-3C-STREP-RNF26 304–432 and his-Vim. His-tagged vimentin was (+NaCl) or was not (−NaCl) allowed to assemble into filaments for 1 h in PBS ± 100 mM NaCl at RT in wells containing standard coverslips before addition of GST-3C-STREP-RNF26 RING or not. After wash-out of excess RNF26, coverslips were fixed in PFA, blocked, and immunostained with anti-his and anti-GST antibodies. Samples were imaged on a spinning-disk confocal microscope. Shown are single channel panels of GST- and his- signals. Scale bar = 1 μM.

D Homology analysis of RNF26 C-terminus (aa 419–433) in H. Sapiens.

E Alphafold structure prediction of RNF26 RING domain. C-terminal vimentin interaction peptide (422–433) is indicated.

F Analysis of synthesized RNF26 C terminal peptide. Shown are *m/z* spectra of LC–MS analyzed peptide RNF26 422–433.

© 2023 The Authors 

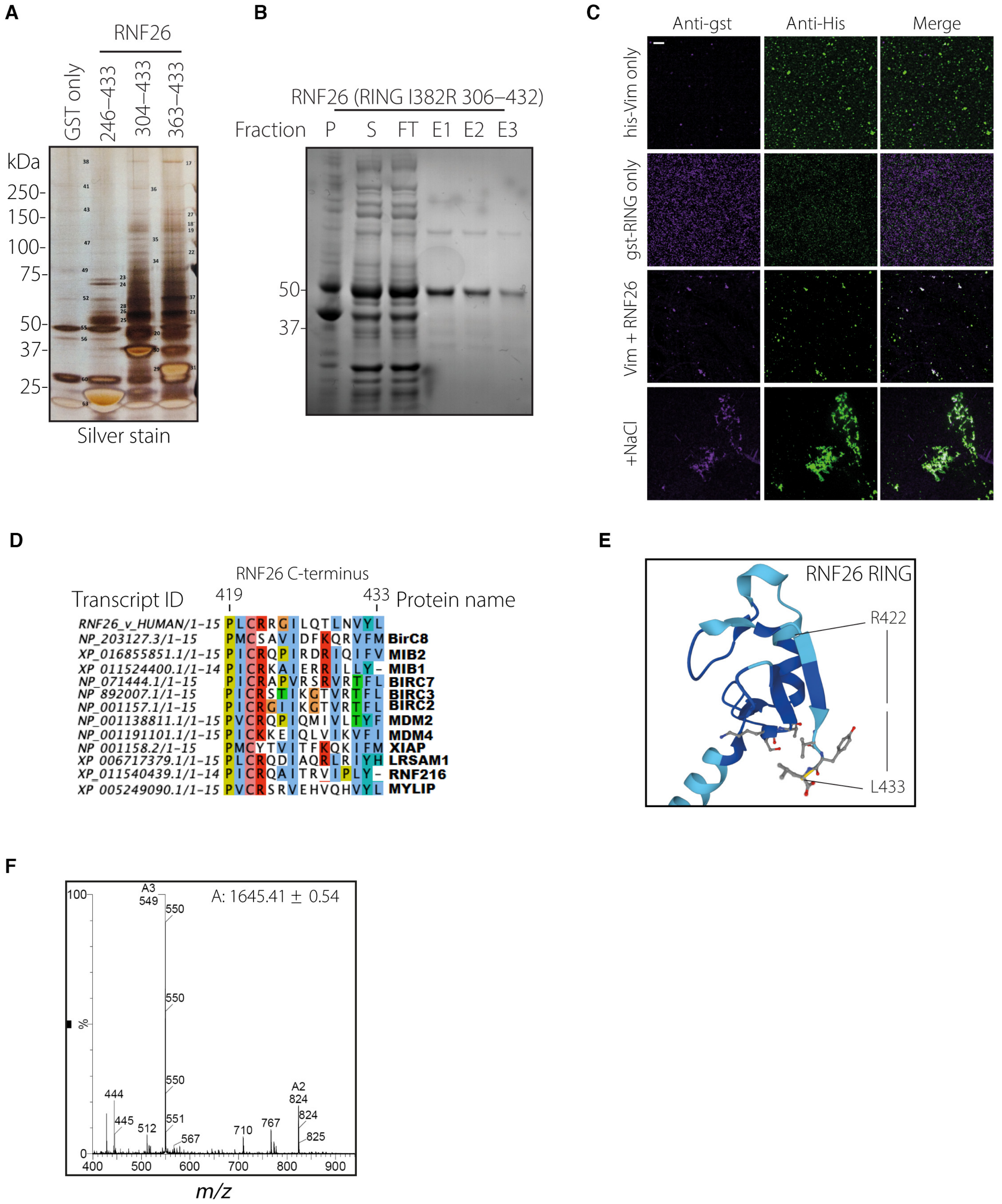


**Figure EV2.**

 © 2023 The Authors

**Figure EV3. (Related to Fig 4): Supporting data on vimentin-mediated RNF26 and endosome organization.**

A, B Knockout validation and RNF26 distribution in HeLa Vim KO cells. (A) Representative image of HeLa Vim KO cells that were transfected with vimentin-IRES-GFP and RFP-RNF26 I382R. Shown are merged images of GFP and RFP-RNF26 signals with indicated zoom ins of (1) a GFP-negative Vim KO#1 cell and (2) a GFP-positive vimentin re-expressing cell, showing vimentin-dependent filamentous perinuclear distribution of RNF26 I382R. (B) Validation of vimentin knockout. Representative immunoblots against endogenous vimentin and Vinculin (loading control) of a Vim KO clone and their parental HeLa cells are shown. Molecular weight markers are indicated.

C Analysis of inactive RNF26 I382R lacking its C-terminal vimentin binding motif with respect to vimentin and VAP-A. Representative image of a U2OS cell transfected with RFP-RNF26 I382R/ΔC and immunostained for endogenous vimentin and VAP-A. Images show overlay of either vimentin (green) or VAP-A (green) with RFP signals, zoom-ins of overlay and single channels that indicate aggregation of RNF26 in vimentin-negative/VAP-A positive regions.

D, E Effect of vimentin depletion on the intracellular distribution early endosomes. (D) U2OS cells transfected with the indicated siRNAs were fixed and immunostained against endogenous EEA1. (E) Quantification of signal distribution as perinuclearity ratio for EEA1 ($n_{\text{Parental}} = 69$, $n_{\text{Vim KO}} = 74$) technical replicates from *t* independent experiments.

F–H Effect of vimentin knockout on the intracellular distribution of endolysosomes in another knockout clone. (G) Validation of vimentin knockout. Representative immunoblots against endogenous vimentin and Vinculin (loading control) of a Vim KO clonal cell line and their parental U2OS cells are shown. Same blot as in Fig 4B. Molecular weight markers are as indicated. (E) Representative confocal images of parental and vimentin KO#2 U2OS cells, fixed and immunostained against endogenous LAMP1. Parental image same as in Fig 4E. (H) Quantification of LAMP1 signal distribution expressed as perinuclearity ratio. Graph reports on $n_{\text{PARENTAL}} = 132$, $n_{\text{Vim KO\#2}} = 96$ technical replicates from three independent experiments. Values were capped at 6.

Data information: Graphs report median (red line) and 95% confidence interval (error bars) of sample values (open circles). Scale bar = 10 µm. Significance was assessed using a Mann–Whitney U test; $***P < 0.001$.

© 2023 The Authors 

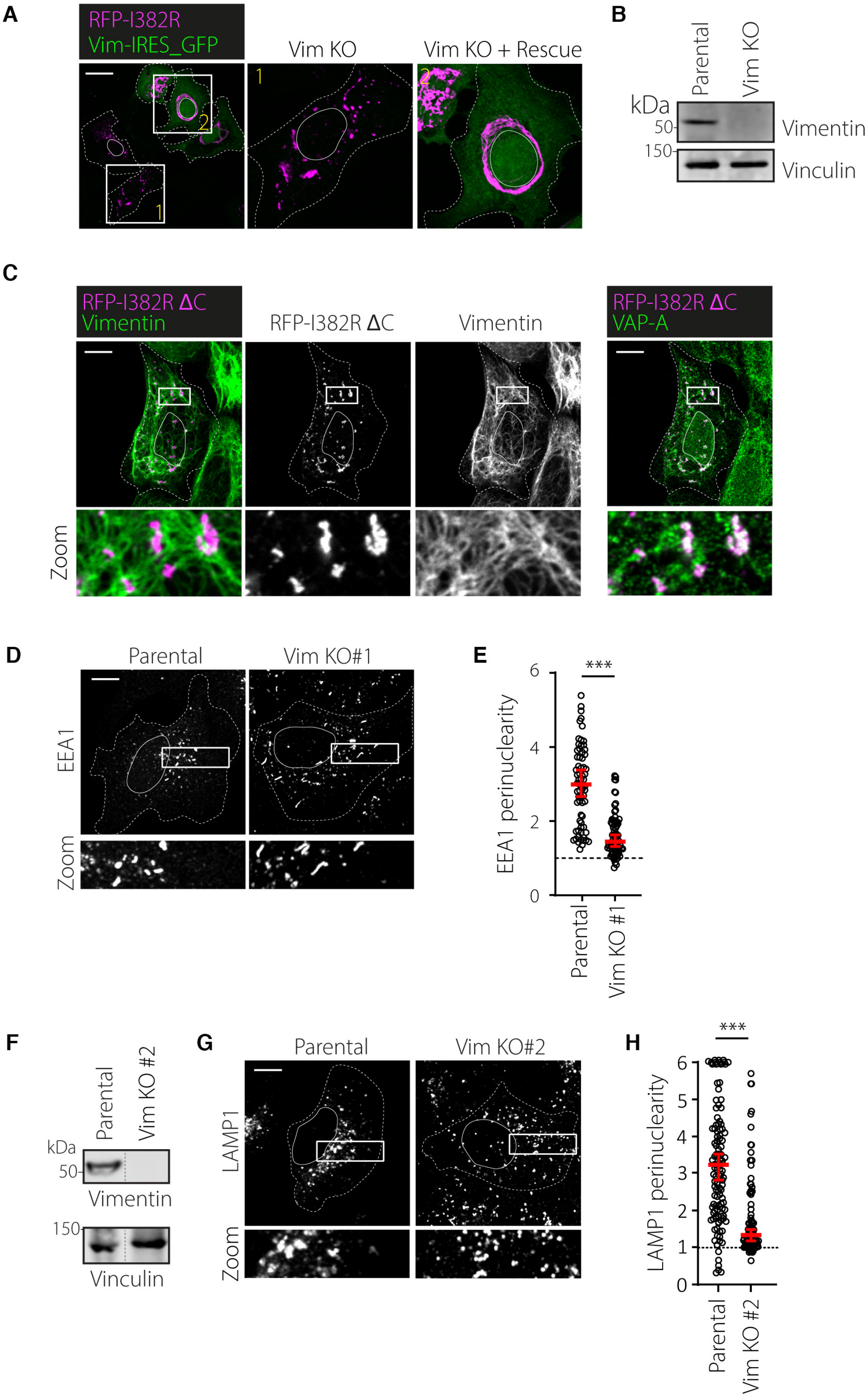


**Figure EV3.**

 © 2023 The Authors

**Figure EV4. (Related to Fig 5): vimentin IFs interact with and organize perinuclear ER membranes.**

A Zero tilt EM image of a perinuclear zoom-in region showing raw data used for tomography in (Fig 5A). Pseudo-colored annotation of the ER (cyan), mitochondria (red), lysosome (green) Golgi apparatus (magenta), and intermediate filaments (yellow) as manually drawn in. Scale bar = 1 µm.

B Examples of vimentin-ER connections from select regions in (A) and adjoined serial sections used for tomography. Arrows indicate vimentin (yellow), ER structures (cyan) and densities that bridged both elements (white). Scale bar = 100 nm.

C Zero-tilt EM image of region showing coalescence of microtubules, actin fibers, and Ifs. We discriminate between the three cytoskeletal elements based on their thickness and general appearance (left). While microtubules have a thick (~24.3 nm) and hollow tube-like appearance, actin filaments often appear branched are thin (~7.8 nm), and IFs are sized in between (~12.7 nm) and are not branched but appear as long stretches. Our identification method was validated by the lack of IFs in Vim KO U2OS cells, whereas the actin and microtubule cytoskeleton remained detectable (right).

D–G Effect of vimentin ablation on the intracellular distribution of ER markers. Parental and two Vim KO U2OS cell lines were fixed and immunostained against endogenous (D) VAP-A or (F) CLIMP63. Representative confocal fluorescence images are shown with boxed zoom-ins highlighting perinuclear regions. Cell and nuclear boundaries are demarcated using dashed and continuous lines, respectively. Quantification of signal distribution as perinuclearity ratio for (E) VAP-A ($n_{\text{Parental}} = 95$, $n_{\text{Vim KO\#1}} = 55$, $n_{\text{Vim KO\#2}} = 26$ technical replicates) and (G) CLIMP63 ($n_{\text{Parental}} = 127$, $n_{\text{Vim KO\#2}} = 69$ technical replicates) from three independent experiments.

Data information: Graphs report median (red line) and 95% confidence interval (error bars) of sample values (open circles). Scale bar = 10 µm. Significance was assessed using a Mann–Whitney U test; *$P < 0{,}05$, ***$P < 0.001$.

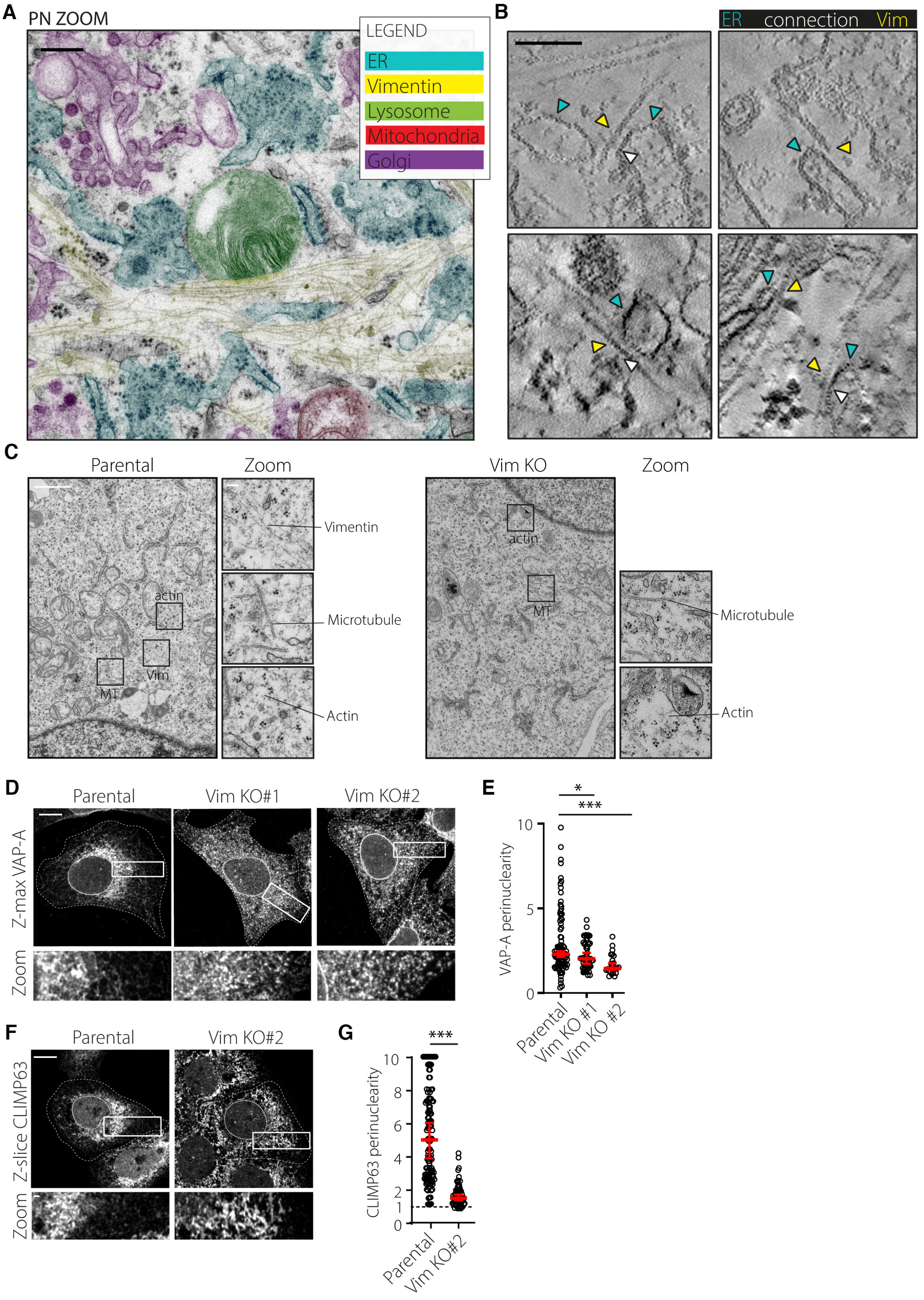


**Figure EV4.**

 © 2023 The Authors

**Figure EV5. (Related to Fig 6): RNF26 and vimentin are required for ER and endosome organization during ER stress.**

A, B Calnexin distribution under Tunicamycin-induced ER stress as a function of RNF26 or vimentin. (A) Shown are representative Z-slices of parental HeLa cells, HeLa Vim KO cells, or HeLa cells silenced for RNF26 (si#1 or #2) that are treated overnight with tunicamycin (5 μg/ml). (B) Perinuclearity analysis of Calnexin signal in cells from (A) ($n_{siC}$ = 71, $n_{siC+}$ = 58, $n_{siRNF26\#1}$ = 36, $n_{siRNF26\#2}$ = 41, $n_{Vim\ KO}$ = 68) technical replicates from three independent experiments.

C, D Tunicamycin-induced late endosome clustering as a function of RNF26. (D) Representative Z-slices of parental U2OS cells, U2OS RNF26 KO cells, or U2OS cells silenced for RNF26 (si#2) or HERP1 (pooled siRNAs), treated with tunicamycin (5 μg/ml) as indicated before fixation and immunostaining for CD63. (E) Perinuclearity analysis of CD63 signal in U2OS cells from (D). $n_{control-}$ = 113, $n_{control+}$ = 228, $n_{siHERP1}$ = 35, $n_{siRNF26\#1}$ = 86, $n_{R26\ KO}$ = 90 technical replicates from three independent experiments.

E, F Tunicamycin-induced UPR signaling as a function of RNF26 or vimentin. XBP1 and XBP1s (E) or BiP (F) transcripts isolated from U2OS cells transfected or genetically modified as indicated and treated in the absence (−) or presence (+) of tunicamycin (2.5 μg/ml, O/N) were detected by qPCR and normalized to GAPDH; $n_{siC\ (-)}$ = 4, $n_{siC\ (+)}$ = 4, $n_{siRNF26\#1\ (+)}$ = 3, $n_{siRNF26\#2\ (+)}$ = 4, $n_{PARENTAL\ (+)}$ = 4, $n_{Vim\ KO\ (+)}$ = 4 independent experiments (error bar = mean and SEM).

G Calnexin distribution under Cyclopiazonic acid-induced ER stress as a function of RNF26 or vimentin. Shown are representative images of siC, Vim KO#2 cells and RNF26 depleted (si#2) U2OS cells treated with cyclopiazonic acid (10 μM) overnight, stained for calnexin and imaged by confocal microscopy.

Data information: Cell and nuclear boundaries are demarcated using dashed and continuous lines, respectively. Scale bar = 10 μm. Significance in (B) and (D) was assessed using a Mann–Whitney U test (error bars reflecting median and 95% confidence interval), and Student's *t*-test (E, F with error bars reflecting mean ± SD); $^{**}P < 0.01$, $^{***}P < 0.001$, $^{****}P < 0.0001$.

© 2023 The Authors

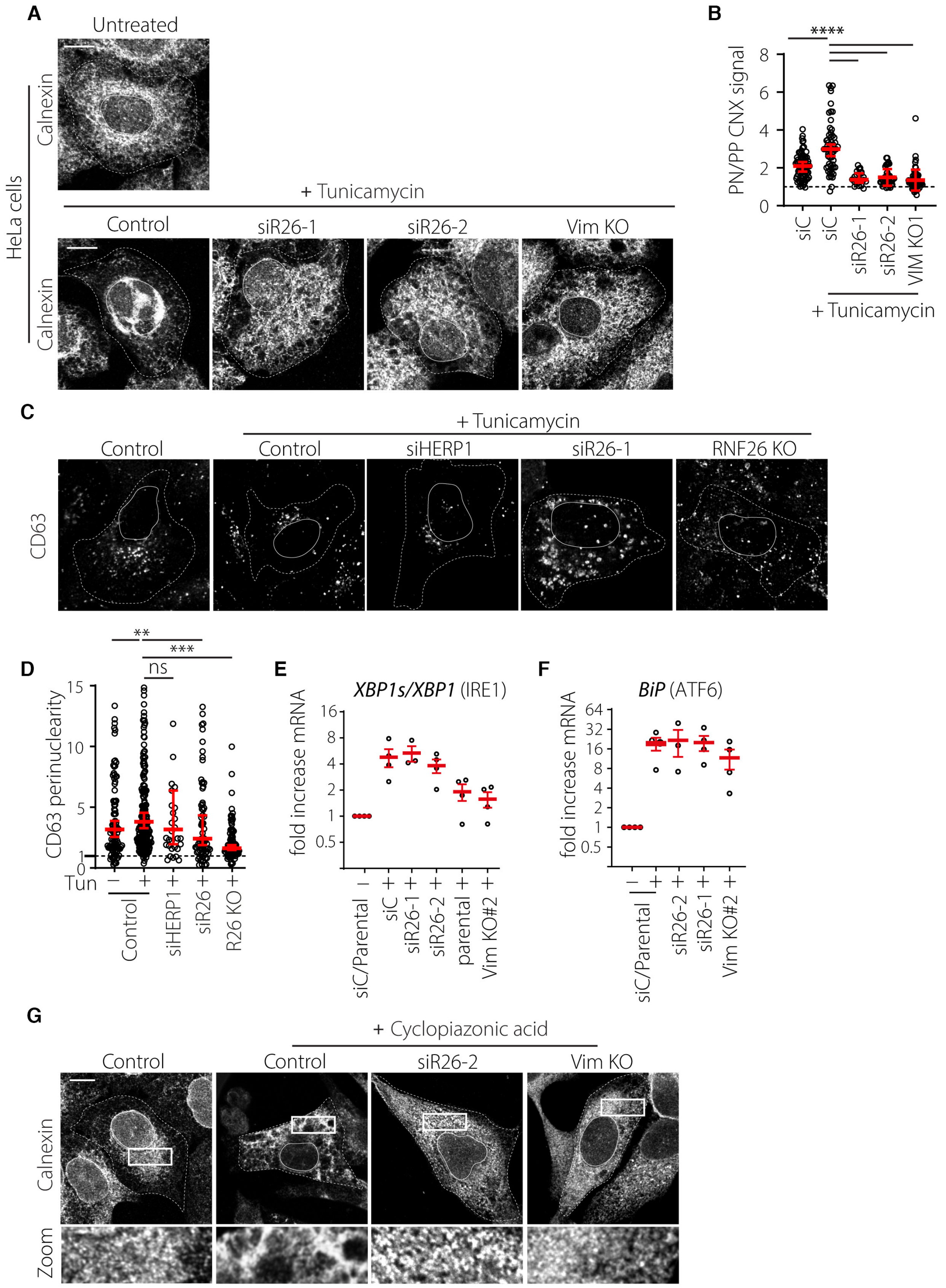


**Figure EV5.**


© 2023 The Authors
