# Supplementary material for: RNF26 binds perinuclear vimentin filaments to integrate ER and endolysosomal responses to proteotoxic stress
Source: EMBO J. 2023 Jul 31;42(18):e111252. doi: 10.15252/embj.2022111252 (PMC10505911; doi:10.15252/embj.2022111252)
Supplement: Supplementary file 2 — Table EV1 [file EMBJ-42-e111252-s006.pdf]

**Table EV1: Mass spectrometry hit list of identified RNF26 RING domain interactors.** Hit list showing protein names, accession numbers, molecular weight (MW), number of uniquepeptides identified and sequence coverage of identified peptides					
**MS/MS sample name**	**Protein name**	**Protein accession#**	**MW (Da)**	**Exclusive un**	**% seq coverage**
GST-RNF26 246-433	Vimentin	IPI00418471	53.652,70	16	40,80%
GST-RNF26 246-433	78 kDa glucose-regulated protein	IPI00003362	72.334,70	18	35,60%
GST-RNF26 304-433	Activating signal cointegrator 1 complex subunit 3	IPI00430472	251.466,80	5	2,54%
GST-RNF26 304-433	ADP/ATP translocase 2	IPI00007188	32.853,50	9	28,90%
GST-RNF26 246-433	Carbonyl reductase [NADPH] 1	IPI00295386	30.374,80	12	42,20%
GST-RNF26 246-433	cDNA FLJ56389, highly similar to Elongation factor 1-gamma	IPI00000875,IPI00937615	56.150,90	13	21,10%
GST-RNF26 363-433	DNA damage-binding protein 1	IPI00293464,IPI00977083	126.970,20	2	1,58%
GST-RNF26 304-433	DnaJ homolog subfamily C member 13	IPI00307259	254.422,90	17	9,05%
GST-RNF26 246-433	Glutathione S-transferase P	IPI00219757	23.356,70	8	43,30%
GST-RNF26 246-433	GMP synthase [glutamine-hydrolyzing]	IPI00029079,IPI00945620,II	76.716,70	3	3,90%
GST-RNF26 304-433	Heterogeneous nuclear ribonucleoprotein F	IPI00003881	45.671,90	12	31,10%
GST-RNF26 363-433	Hornerin	IPI00398625	282.372,70	5	2,07%
GST-RNF26 363-433	Isoform 1 of ATP-dependent zinc metalloprotease YME1L1	IPI00045946,IPI00099529,II	86.458,50	3	5,43%
GST-RNF26 363-433	Isoform 1 of Calpain-15	IPI00024190	117.311,90	15	16,60%
GST-RNF26 363-433	Isoform 1 of Epidermal growth factor receptor substrate 15	IPI00292134	98.658,00	6	7,59%
GST-RNF26 246-433	Isoform 1 of Heat shock cognate 71 kDa protein	IPI00003865	70.899,80	36	46,00%
GST-RNF26 246-433	Isoform 1 of Heterogeneous nuclear ribonucleoprotein K	IPI00216049,IPI00216746,II	50.978,50	3	6,70%
GST-RNF26 304-433	Isoform 1 of Mitochondrial inner membrane protein	IPI00009960,IPI00554469,II	83.679,00	15	21,50%
GST-RNF26 363-433	Isoform 1 of RanBP-type and C3HC4-type zinc finger-containing protein 1	IPI00783058	57.570,00	19	40,60%
GST-RNF26 304-433	Isoform 1 of RING finger protein 31	IPI00094740	119.652,60	4	3,92%
GST-RNF26 304-433	Isoform 1 of Sequestosome-1	IPI00179473	47.687,40	4	15,70%
GST-RNF26 363-433	Isoform 1 of TOM1-like protein 2	IPI00446294	55.556,70	8	17,00%
GST-RNF26 363-433	Isoform 1 of Ubiquitin carboxyl-terminal hydrolase 15	IPI00000728	112.423,40	6	5,40%
GST-RNF26 246-433	Isoform 1 of UPF0378 protein KIAA0100	IPI00373894,IPI00784567,II	253.702,80	2	0,45%
GST-RNF26 304-433	Isoform 1AB of Catenin delta-1	IPI00182469,IPI00182540,II	107.351,00	3	2,91%
GST-RNF26 304-433	Isoform 2 of ATPase WRNIP1	IPI00102997,IPI00290314	69.460,20	8	12,00%
GST-RNF26 304-433	Isoform 2 of Coiled-coil domain-containing protein 50	IPI00217059,IPI00383423	56.339,70	2	4,77%
GST-RNF26 304-433	Isoform 2 of Dedicator of cytokinesis protein 7	IPI00183572,IPI00513791,II	241.417,60	2	1,13%
GST-RNF26 304-433	Isoform 2 of MAP7 domain-containing protein 2	IPI00554579,IPI00844217,II	86.350,90	3	1,68%
GST-RNF26 363-433	Isoform 2 of Sharpin	IPI00382790,IPI00873534	33.877,10	4	14,40%
GST-RNF26 363-433	Isoform 2 of Tax1-binding protein 1	IPI00106559,IPI00657950,II	86.257,10	23	25,80%
GST-RNF26 246-433	Isoform Mitochondrial of Glutathione reductase, mitochondrial	IPI00016862,IPI00759575,II	56.257,40	4	8,05%
GST-RNF26 363-433	Kelch-like ECH-associated protein 1	IPI00106502	69.664,70	9	17,00%
GST-RNF26 246-433	Keratin, type I cytoskeletal 10	IPI00009865	58.828,80	15	25,00%
GST-RNF26 246-433	Keratin, type II cytoskeletal 1	IPI00220327	66.040,30	23	34,80%
GST-RNF26 246-433	Keratin, type II cytoskeletal 2 epidermal	IPI00021304	65.866,40	11	18,60%
GST-RNF26 363-433	Keratin, type II cytoskeletal 2 epidermal	IPI00021304	65.866,40	5	8,37%
GST-RNF26 246-433	Keratin, type II cytoskeletal 5	IPI00009867	62.379,60	4	7,12%
GST-RNF26 246-433	Keratin, type II cytoskeletal 6C	IPI00299145	60.026,70	7	20,40%
GST-RNF26 363-433	Myosin	IPI00844172	145.021,60	41	30,90%
GST-RNF26 363-433	Poly [ADP-ribose] polymerase 10	IPI00064457,IPI00792479,II	109.997,20	16	17,00%
GST-RNF26 246-433	Protein RCC2	IPI00465044	56.084,80	21	40,00%
GST-RNF26 246-433	RING finger protein 26	IPI00012431	47.737,90	4	9,01%
GST-RNF26 246-433	Stress-70 protein, mitochondrial	IPI00007765,IPI00966238	73.681,30	31	37,00%
GST-RNF26 304-433	Toll-interacting protein	IPI00100154	30.281,40	5	17,20%
GST-RNF26 246-433	Trypsin-1	IPI00011694,IPI00945846,II	26.558,10	2	7,29%
GST-RNF26 246-433	Tubulin alpha-1B chain	IPI00930688	50.151,70	2	5,32%
GST-RNF26 246-433	Ubiquitin-40S ribosomal protein S27a	IPI00179330,IPI00456429,II	17.965,60	5	25,00%
