# Supplementary material for: RNF26 binds perinuclear vimentin filaments to integrate ER and endolysosomal responses to proteotoxic stress
Source: EMBO J. 2023 Jul 31;42(18):e111252. doi: 10.15252/embj.2022111252 (PMC10505911; doi:10.15252/embj.2022111252)
Supplement: Supplementary file 3 — Movie EV1 [file EMBJ-42-e111252-s007.zip › Movie EV1 legend.docx]

**Movie EV1 (related to Fig. 1):** Live cell recording of ER dynamics as a function of RNF26. Shown are 60 sec (3 frames/ sec) movies of siC-treated (**Movie EV1**) or siRNF26 (si#1)-treated (Movie EV2) cells expressing mCherry-KDEL. Stills, zooms, and time color coded images are shown in Fig. 1B.
