# Supplementary material for: RNF26 binds perinuclear vimentin filaments to integrate ER and endolysosomal responses to proteotoxic stress
Source: EMBO J. 2023 Jul 31;42(18):e111252. doi: 10.15252/embj.2022111252 (PMC10505911; doi:10.15252/embj.2022111252)
Supplement: Supplementary file 4 — Movie EV2 [file EMBJ-42-e111252-s010.zip › Movie EV2 legend.docx]

**Movie EV2 (related to Fig. 1):** Live cell recording of ER dynamics as a function of RNF26. Shown are 60 sec (3 frames/ sec) movies of siC-treated (Movie EV1) or siRNF26 (si#1)-treated (**Movie EV2**) cells expressing mCherry-KDEL. Stills, zooms, and time color coded images are shown in Fig. 1B.
