# Supplementary material for: RNF26 binds perinuclear vimentin filaments to integrate ER and endolysosomal responses to proteotoxic stress
Source: EMBO J. 2023 Jul 31;42(18):e111252. doi: 10.15252/embj.2022111252 (PMC10505911; doi:10.15252/embj.2022111252)
Supplement: Supplementary file 5 — Movie EV3 [file EMBJ-42-e111252-s018.zip › Movie EV3 legend.docx]

**Movie EV3 (related to Fig. 4):** Endosome maturation as a function of Vimentin. Parental (**Movie EV3**) or Vim KO#2 (Movie EV4) U2OS cells were incubated with SiR-lysosome (magenta) to visualize proteolytic compartments followed by 5µM SR101 (green) prior to imaging with a spinning-disc confocal microscope. Movie shows 2hr timelapse at a 5hz speed with 10min/frame each.
