# Supplementary material for: RNF26 binds perinuclear vimentin filaments to integrate ER and endolysosomal responses to proteotoxic stress
Source: EMBO J. 2023 Jul 31;42(18):e111252. doi: 10.15252/embj.2022111252 (PMC10505911; doi:10.15252/embj.2022111252)
Supplement: Supplementary file 7 — Movie EV5 [file EMBJ-42-e111252-s015.zip › Movie EV5 legend.docx]

**Movie EV5 (related to Fig. 5):** Fly-over movie of 3D TEM tomography of a perinuclear region of a U2OS cell, accompanying Fig. 5A. Zoom ins are accompanied by original TEM micrographs, showing close connections of the ER and intermediate filaments. Ultrastructures were manually annotated and pseudocolored as following: ER (cyan), mitochondria (red), lysosome (green) Golgi apparatus (magenta), intermediate filaments (yellow) and microtubule (grey).
