# Supplementary material for: RNF26 binds perinuclear vimentin filaments to integrate ER and endolysosomal responses to proteotoxic stress
Source: EMBO J. 2023 Jul 31;42(18):e111252. doi: 10.15252/embj.2022111252 (PMC10505911; doi:10.15252/embj.2022111252)
Supplement: Supplementary file 8 — Movie EV6 [file EMBJ-42-e111252-s017.zip › Movie EV6 legend.docx]

**Movie EV6 (related to Fig. 5):** Live cell recording of a U2OS cell transiently expressing GFP-Vimentin (green) and mCherry-KDEL (magenta). Shown is an overlay of both channels over 60 frames (1 frame/sec). Stills, zooms, and time color coded images are shown in Fig. 5B.
