# Supplementary material for: RNF26 binds perinuclear vimentin filaments to integrate ER and endolysosomal responses to proteotoxic stress
Source: EMBO J. 2023 Jul 31;42(18):e111252. doi: 10.15252/embj.2022111252 (PMC10505911; doi:10.15252/embj.2022111252)
Supplement: Supplementary file 10 — Movie EV8 [file EMBJ-42-e111252-s012.zip › Movie EV8 legend.docx]

**Movie EV8 (related to Fig. 5):** Live cell recording of ER dynamics as a function of Vimentin. Shown are 60 sec (3 frames/ sec) movies of U2OS WT (Movie EV7) or Vim KO#1 (**Movie EV8**) cells expressing mCherry-KDEL. Stills, zooms, and time color coded image are shown in Fig. 5D.
