# Supplementary material for: RNF26 binds perinuclear vimentin filaments to integrate ER and endolysosomal responses to proteotoxic stress
Source: EMBO J. 2023 Jul 31;42(18):e111252. doi: 10.15252/embj.2022111252 (PMC10505911; doi:10.15252/embj.2022111252)
Supplement: Supplementary file 11 — PDF+ [file EMBJ-42-e111252-s014.pdf]

Article

SOURCE DATA TRANSPARENT PROCESS OPEN ACCESS

THE EMBO JOURNAL

RNF26 binds perinuclear vimentin filaments to integrate ER and endolysosomal responses to proteotoxic stress

Tom Cremer[1,2], Lenard M Voortman[1], Erik Bos[1], Marlieke LM Jongsma[1,2], Laurens R ter Haar[1], Jimmy JLL Akkermans[1,2], Cami MP Talavera Ormeño[1], Ruud HM Wijdeven[1,2,3], Jelle de Vries[1], Robbert Q Kim[1], George MC Janssen[1], Peter A van Veelen[1], Roman I Koning[1], Jacques Neefjes[1,2,*] & Ilana Berlin[1,2,**]

Abstract

Proteotoxic stress causes profound endoplasmic reticulum (ER) membrane remodeling into a perinuclear quality control compartment (ERQC) for the degradation of misfolded proteins. Subsequent return to homeostasis involves clearance of the ERQC by endolysosomes. However, the factors that control perinuclear ER integrity and dynamics remain unclear. Here, we identify vimentin intermediate filaments as perinuclear anchors for the ER and endolysosomes. We show that perinuclear vimentin filaments engage the ER-embedded RING finger protein 26 (RNF26) at the C-terminus of its RING domain. This restricts RNF26 to perinuclear ER subdomains and enables the corresponding spatial retention of endolysosomes through RNF26-mediated membrane contact sites (MCS). We find that both RNF26 and vimentin are required for the perinuclear coalescence of the ERQC and its juxtaposition with proteolytic compartments, which facilitates efficient recovery from ER stress via the Sec62-mediated ER-phagy pathway. Collectively, our findings reveal a scaffolding mechanism that underpins the spatiotemporal integration of organelles during cellular proteostasis.

Keywords endolysosomes; ERphagy; ER stress; intermediate filaments; RNF26
Subject Categories Cell Adhesion, Polarity & Cytoskeleton; Membranes & Trafficking
DOI 10.15252/embj.2022111252 | Received 23 March 2022 | Revised 28 June 2023 | Accepted 3 July 2023 | Published online 31 July 2023
The EMBO Journal (2023) 42: e111252

Introduction

In mammalian cells, the perinuclear region stages a get-together for diverse membranous organelles that collectively execute essential processes of cellular biology (Neefjes *et al*, 2017; Cohen *et al*, 2018). For instance, perinuclear localization of the "rough" endoplasmic reticulum (ER), Golgi, mitochondria, and endolysosomes has been associated with diverse cellular functions ranging from biosynthesis and energy production (Bravo *et al*, 2011; Leitman *et al*, 2013) to nutrient sensing and degradation (Korolchuk *et al*, 2011; Johnson *et al*, 2016; Starling *et al*, 2016), modulation of signaling responses (Jia & Bonifacino, 2019; Cremer *et al*, 2021), and maintenance of cell polarity (Ang & Fölsch, 2012). However, mechanisms driving cross-compartmental integration in perinuclear space remain elusive. Transport and anchorage of organelles in cellular space are known to involve various cytoskeletal elements and molecular motors. While the roles of microtubule and actin networks with respect to organellar organization and motility have been extensively studied (Hirokawa *et al*, 2009; Reck-Peterson *et al*, 2018; Svitkina, 2018), contributions of intermediate filaments (IFs) in this context are only beginning to emerge (Chang *et al*, 2009; Lowery *et al*, 2015; Schwarz & Leube, 2016). In this study, we identify a key role for vimentin IFs as perinuclear anchors for the ER and endolysosomes and reveal its dynamic molecular interplay with the ER-embedded protein RNF26 in the context of the ER stress response.

The ER is a master coordinator of cellular homeostasis (Goyal & Blackstone, 2013). To facilitate its breadth of function, the ER membrane network is divided into biochemically and morphologically distinct regions (Goyal & Blackstone, 2013; Leitman *et al*, 2013; Westrate *et al*, 2015). The densely packed perinuclear ER segment accommodates protein biosynthesis, folding, and quality control, as well as forms the front line of stress responses, while the dynamic peripheral network of ER tubules manages calcium sensing and uptake (Wu *et al*, 2018; Cremer *et al*, 2020). The perinuclear ER subdomain is rich in ER sheets and is defined by the presence of CLIMP63, a coiled-coil protein that acts as a luminal spacer between opposing membranes and integrates various signals to affect ER

1 Department of Cell and Chemical Biology, Leiden University Medical Center, Leiden, The Netherlands
2 Oncode Institute, Leiden University Medical Center, Leiden, The Netherlands
3 Alzheimer Center Amsterdam, Department of Neurology, Amsterdam Neuroscience, Amsterdam University Medical Center, Amsterdam, The Netherlands
*Corresponding author. Tel: +31 715268722; E-mail: j.j.c.neefjes@lumc.nl
**Corresponding author. Tel: +31 715268729; E-mail: i.berlin@lumc.nl

© 2023 The Authors. Published under the terms of the CC BY 4.0 license

morphology (Vedrenne *et al*, 2005; Shibata *et al*, 2010; Shen *et al*, 2019; Sandoz *et al*, 2023). Much of the enquiry into the mechanisms of perinuclear ER organization has been focused on the contributions of the microtubule network and its associated proteins. In addition to CLIMP63, previous studies have identified Kinectin, p180/RRBP1, VIMP, and the long isoform of Syntaxin 5 as potent microtubule binding organizers of the ER (Klopfenstein *et al*, 1998; Ogawa-Goto *et al*, 2007; Miyazaki *et al*, 2012; Noda *et al*, 2014; Zheng *et al*, 2022). Movement of ER membranes is also driven by the microtubule network, either through direct interactions with microtubules themselves, binding of their associated motor proteins, or hitchhiking along with other organelles utilizing these transport highways (Waterman-Storer & Salmon, 1998; Rodriguez-Garcia *et al*, 2020; Spits *et al*, 2021). With respect to the latter, it is becoming clear that physical contacts between the ER and endolysosomes have a profound impact on ER organization and dynamics (Lu *et al*, 2020; Spits *et al*, 2021). Interactions between organelles take place at specialized membrane contact sites (MCSs) driven by reversible *in trans* pairings of dedicated tether proteins (Eisenberg-Bord *et al*, 2016; Wu *et al*, 2018). Besides influencing organelle motility, these physical interactions provide unique opportunities for communication and material exchange (Prinz *et al*, 2020), and ER MCSs are well-established to play important roles in lipid homeostasis and calcium signaling (Jain & Holthuis, 2017; Cremer *et al*, 2020). To effectively integrate its plethora of functions, the ER network is subject to continuous remodeling.

As the organelle responsible for production of all membrane and secretory proteins, the ER regularly experiences proteotoxic stress, which is implicated in various human pathologies (Hetz & Saxena, 2017; Ren *et al*, 2021; Ajoolabady *et al*, 2023). During times of stress, the ER relies heavily on compartmentalization to segregate misfolded proteins away from the healthy ER segments and ensure their efficient clearance through the ER quality control compartment (ERQC) (Leitman *et al*, 2013; Oikonomou & Hendershot, 2020). Here, concentration of chaperone-bound misfolded substrates with the ER-associated protein degradation (ERAD) machinery promotes their ubiquitin-dependent disposal by proteasomes and thus facilitates return to homeostasis (Leitman *et al*, 2014). When the ER stress response enters the resolution phase, additional clearance mechanisms are engaged, which include direct engulfment of excess ER membranes and chaperones by endolysosomes via the Sec62-mediated ERphagy pathway (Reggiori & Molinari, 2022). Yet, the molecular mechanisms underpinning formation and disposal of the perinuclear ERQC domains remain unclear. We have previously shown that perinuclear congregation of proteolytic compartments is instructed by the ER-embedded ubiquitylation complex comprised of the E3 ligase RING finger protein 26 (RNF26) and its cognate conjugating enzyme UBE2J1 (Jongsma *et al*, 2016; Cremer *et al*, 2021). RNF26 resides in the perinuclear ER membrane, and this feature enables it to spatially restrict various endolysosomal species by attracting ubiquitin-binding vesicle adaptors through a ubiquitin-dependent ER MCS (Jongsma *et al*, 2016). However, what ensures the perinuclear retention of RNF26—and that of other ER membrane-embedded proteins—is unknown. Moreover, whether RNF26 and its molecular partners contribute to the global governance of ER architecture and dynamics has not been explored.

Here, we reveal that the vimentin cytoskeleton collaborates with RNF26 to maintain the integrity of the perinuclear ER subdomain and promote the architecture and function of the endolysosomal cloud. We observe that perinuclear vimentin filaments preferentially engage RNF26 in its catalytically inactive state through a direct interaction involving the C-terminus of its atypical RING domain. This interaction restricts RNF26 within the perinuclear ER segment, where its ubiquitin ligase activity then enables the corresponding spatial arrangement of endolysosomes in perinuclear space. Through this dynamic interplay, vimentin and RNF26 guard organellar distribution in steady state and drive acute perinuclear coalescence of ER with endolysosomes under proteotoxic stress conditions. The resulting juxtaposition of biosynthetic and proteolytic compartments in turn facilitates the recovery from ER stress via Sec62-mediated ERphagy. Vimentin IFs have thus far been appreciated predominantly for their structural contributions to cell rigidity (Toivola *et al*, 2005; Lowery *et al*, 2015; Etienne-Manneville, 2018) and as markers of the epithelial-to-mesenchymal transition during cancer progression (Ivaska, 2011; Usman *et al*, 2021). Our findings now position IFs, along with the microtubule and actin networks (Svitkina, 2018; Zheng *et al*, 2022), as dynamic regulators of organellar compartmentalization and mobilization in response to stress.

Results

RNF26 regulates the integrity of the perinuclear ER subdomain

Under steady-state conditions, adherent cells centralize their membranous organelles in perinuclear (PN) space (Fig 1A). While the cell's largest organelle—the ER—extends throughout the cytoplasm, its membranes tend to pack more densely near the nucleus and remain less mobile over time, as compared to their highly dynamic tubule counterparts reaching outwards into the cell periphery (PP) (Fig 1B, Movie EV1). How perinuclear/peripheral compartmentalization of the ER is established and maintained in molecular terms is not well-defined. We have previously shown that RNF26 preferentially localizes within the PN ER subdomain and from this vantage point engages vesicles of the endolysosomal system to control cargo transport and signaling events (Jongsma *et al*, 2016). We now found that loss of RNF26 also disturbs the organization of the ER network in space and time (Fig 1B–J). Specifically, concentration of ER membranes in the PN region of U2OS cells expressing the fluorescent ER marker mCherry-KDEL diminished in response to either transient depletion or complete ablation of RNF26 (Figs 1B and EV1A–C). To quantify perinuclear localization in an unbiased manner, we developed a Fiji plugin to calculate the perinuclearity coefficient by comparing the average signal intensity per pixel between binned PN and PP areas (Fig 1C). With this approach, we determined that perinuclearity of mCherry-KDEL strongly diminished in live cells where RNF26 was either silenced or knocked-out (KO) (Figs 1D and EV1D), while the total area of the cell occupied by the ER network remained unchanged (Fig 1E). A similar effect was observed in fixed cells immunolabeled against endogenous VAP-A (Fig EV1E and F). Furthermore, defective compartmentalization of the ER induced by loss of RNF26 correlated with altered membrane dynamics, as illustrated by enhanced motility of the PN ER segment in RNF26-depleted cells (Fig 1B and F; Movies EV1 and EV2).

Upon closer inspection of ER morphology, it became evident that ER sheets, normally retained in the PN region, had spread into the

 © 2023 The Authors

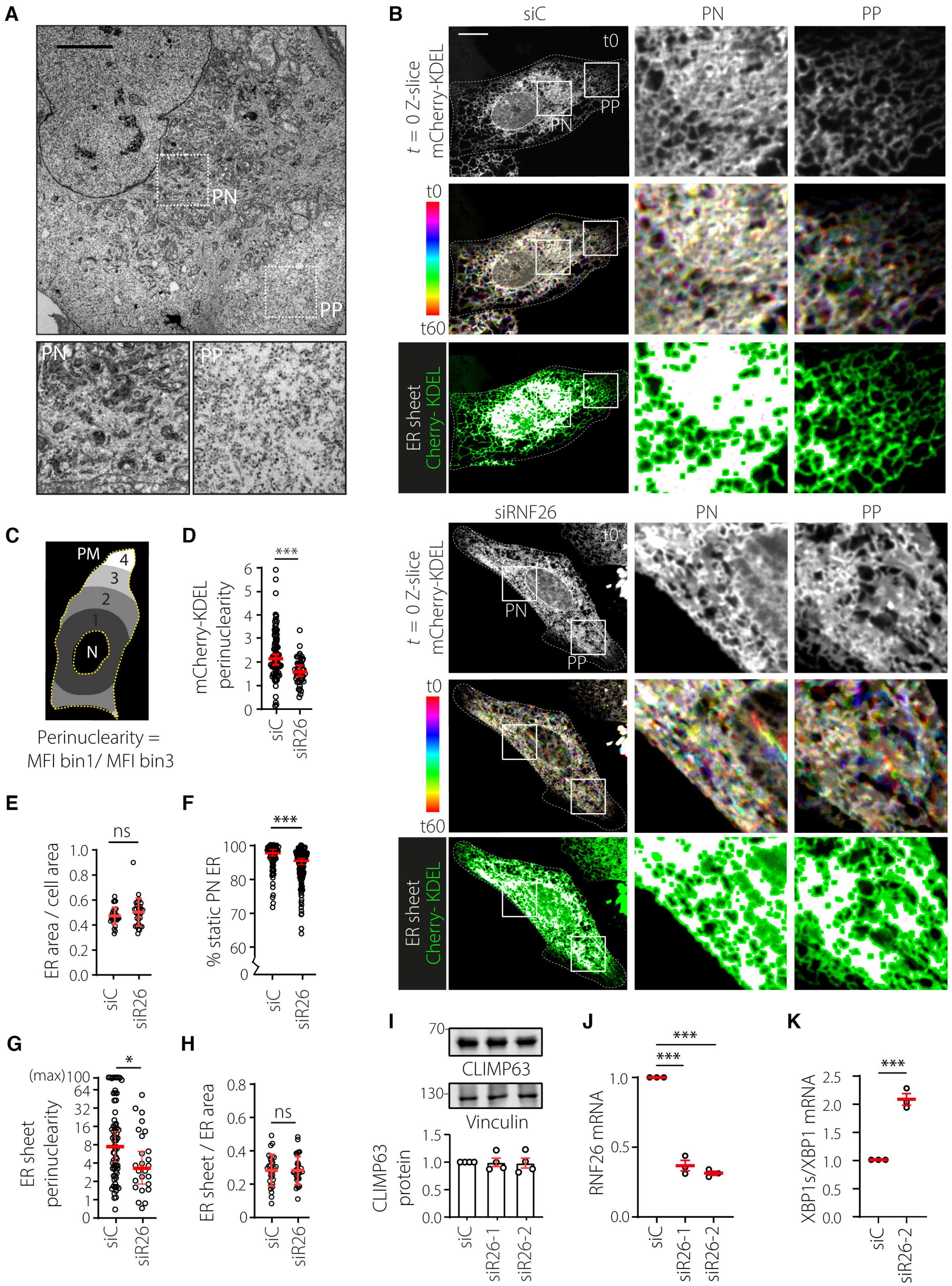
A
PN
PP
B
siC
PN
PP
t = 0 Z-slice
mCherry-KDEL
t0
t60
ER sheet
Cherry- KDEL
siRNF26
C
PM
4
3
2
1
N
Perinuclearity =
MFI bin1/ MFI bin3
D
mCherry-KDEL
perinuclearity
***
siC
siR26
E
ER area / cell area
ns
F
% static PN ER
***
G
ER sheet
perinuclearity
(max)
*
H
ER sheet / ER area
ns
I
CLIMP63
Vinculin
CLIMP63
protein
siR26-1
siR26-2
J
RNF26 mRNA
***
K
XBP1s/XBP1 mRNA
***

Figure 1.

Figure 1. RNF26 controls ER organization and dynamics.

A Intracellular distribution of organelles at steady state. EM micrograph of a wild-type U2OS cell. Zoom-ins highlight select perinuclear (PN) and peripheral (PP) cell regions. Scale bar = 1 μm.

B–H Analysis of ER architecture and dynamics as a function of RNF26 in U2OS cells ectopically expressing mCherry-KDEL and transfected with either control siRNA (siC) or siRNAs targeting RNF26 (siRNF26-1 or siRNF26-2). (B) Time-lapse imaging. Top panels: representative confocal fluorescence images of mCherry-KDEL (*white*) at the start of time-lapse $t = 0$ (top). Middle panels: time color coded overlays of 60x1s frames, each assigned a different color LUT (white–same ER location across all frames; color—ER movement between frames). Bottom panels: overlays of twice eroded ER sheet masks (*magenta*) with mCherry-KDEL signal (*green*). Masks were generated using ImageJ software. Zoom-ins show select PN and PP regions. Cell and nuclear boundaries are demarcated using dashed and continuous lines, respectively. Scale bar = 10 μm. See also Movies EV1 and EV2. (C) Strategy for quantifying intracellular distribution of fluorescent signals as perinuclearity ratio. Space between manually annotated nucleus (N) and plasma membrane (PM) was classified into four bins using a custom Fiji plugin (detailed under Materials and Methods section). Mean fluorescence intensity (MFI) of bin1 (PN)/bin3 (PP) yields a perinuclearity ratio. (D) Quantification of mCherry-KDEL signal distribution as perinuclearity ratio, $n_{siC} = 99$, $n_{siR26} = 46$ technical replicates from three independent experiments. (E) Quantification of ER occupancy (area) between N and PM defined as in (C). ER area was calculated using particle analysis on thresholded mCherry-KDEL signal using Fiji. Graph reports $n_{siC} = 29$, $n_{siR26} = 30$ technical replicates from three independent experiments. (F) Quantification of perinuclear ER dynamics. Movement of mCherry-KDEL-positive pixels from 5 × 5 μm ROIs binned into static (0–60 nm/s) or dynamic (61–780 nm/s) vectors using custom membrane displacement analysis (MDA) for Fiji as previously described (Spits *et al*, 2021), $n_{siC} = 84$, $n_{siR26} = 147$ technical replicates (4 ≥ ROIs per cell, 20 ≥ cells) from three independent experiments. (G) Quantification of ER sheet mask (B, bottom panel) distribution as perinuclearity ratio (max MFI bin1/bin3 = 100), $n_{siC} = 70$, $n_{siRNF26} = 26$ technical replicates from three independent experiments. (H) Quantification of ER sheet (B, bottom panel) abundance as ER sheet mask area relative to total ER area on the sample set in (E).

I CLIMP63 abundance as a function of RNF26. Top: representative immunoblot of U2OS cells transfected with the indicated siRNAs stained against endogenous CLIMP63 and Vinculin (loading control). Bottom: quantification of CLIMP63 abundance, normalized to Vinculin and expressed relative to control (siC = 1.0), $n = 4$ independent experiments.

J, K Effect of RNF26 depletion on basal ER stress using IRE1-mediated XBP1 splicing as a readout. (J) Relative abundance of RNF26 transcript normalized to GAPDH; $n = 3$ independent experiments. All transcripts were detected by qPCR. Plots report values relative to control (siC = 1.0). (K) Quantification of IRE1-dependent XBP1 splicing. XBP-s and XBP1 transcripts isolated from U2OS cells transfected with the indicated siRNAs were detected by qPCR, normalized to GAPDH, and expressed as ratio, $n = 3$ independent experiments.

Data information: Graphs report mean or median (red line) of sample values (open circles). Significance was assessed using Student's *t*-test (E, G and I–K with error bars reflecting ± SD) or Mann–Whitney U test (D, F and H with error bars reflecting median and 95% confidence interval); *$P < 0.05$, ***$P < 0.001$, ns: not significant. See also Fig EV1.
Source data are available online for this figure.

periphery of RNF26-depleted cells (Fig 1B and G), without affecting either the overall abundance of sheets per cell or protein levels of the ER sheet-associated marker CLIMP63 (Fig 1H and I). Instead, loss of RNF26 caused marked redistribution of CLIMP63 toward the cell periphery (Fig EV1G and H), further substantiating a positive role for RNF26 in the maintenance of PN ER integrity. The PN ER segment is associated with protein synthesis and quality control, and as such is highly sensitive to stress. We therefore investigated whether loss of RNF26 has physiological implications in this context. Indeed, enhanced ER stress-associated splicing of the XBP1 transcript was observed in response to RNF26 depletion (Fig 1J and K), indicating that the perinuclear architectural changes brought upon by loss of RNF26 coincide with elevation in basal ER stress. Taken together, these observations reveal that RNF26 regulates ER compartmentalization, dynamics, and homeostasis.

RNF26 directly binds vimentin IFs

To understand how RNF26 contributes to the spatiotemporal compartmentalization of the ER, we probed the interacting partners of its soluble C-terminal fragment containing the RING domain (Fig 2A and B), which was previously established to govern the perinuclear distribution of RNF26 within the ER membrane (Jongsma *et al*, 2016). Among the proteins coprecipitating with purified soluble GST-fusion fragments of RNF26 from cell lysates was vimentin (Figs 2B and EV2A; Table EV1), a building block of type III IFs (Herrmann & Aebi, 2016). To examine whether the association between RNF26 and vimentin can also take place inside the cell, we employed the proximity biotinylation technique (Branon *et al*, 2018). Although biotinylation of endogenous vimentin by the wild-type RNF26 fused to a promiscuous biotin ligase moiety TurboID was rather modest, it was still sensitive to the removal of the RING-containing C-terminal fragment of RNF26 (Fig 2C and D) used as bait in the identification of vimentin (Fig EV2A). Previously characterized interactions of RNF26 with the endosomal adaptors TOLLIP, EPS15, and TAX1BP1 (Fig 2B) have been shown to strongly rely on the E3 ligase activity of RNF26 (Jongsma *et al*, 2016). By contrast, binding of vimentin was instead enhanced by catalytic inactivation of RNF26, which was achieved through point mutation of isoleucine 382 to arginine (I382R, Fig 2C and D). This difference in preference supports a model wherein the RING domain of RNF26 could discriminate between binding partners depending on its activity status (Fig 2B).

Next, to explore whether RNF26 and vimentin interact directly with one another, we fluorescently labeled the purified catalytically inactive RING domain of RNF26, generating Rhodamine-RING-I382R (304–433) (Fig EV2B). Recovery of Rhodamine-RING-I382R by recombinant His-tagged vimentin precipitated using Ni-NTA beads implied direct binding between these proteins (Fig 2E), which was further supported by the deposition of Rhodamine-RING-I382R onto *in vitro* assembled vimentin filaments (Figs 2F and EV2C). Seeking to pinpoint the region in RNF26 responsible for binding to vimentin, we consulted the structure prediction software AlphaFold (Jumper *et al*, 2021). This revealed a β-sheet at the C-terminus of RNF26 RING domain, a feature conserved in RNF26 genes across species but found only in a subset of RING-containing proteins (Figs 2A and EV2D and E). Strikingly, removal of the last 10 residues from the C-terminus of catalytically inactive RNF26 (I382R-ΔC) abrogated proximity-based biotinylation of endogenous vimentin (Fig 2A and G), suggesting that a productive interaction with vimentin is mediated by this sequence. Indeed, concentration-dependent binding of purified vimentin to the Rhodamine-labeled

 © 2023 The Authors

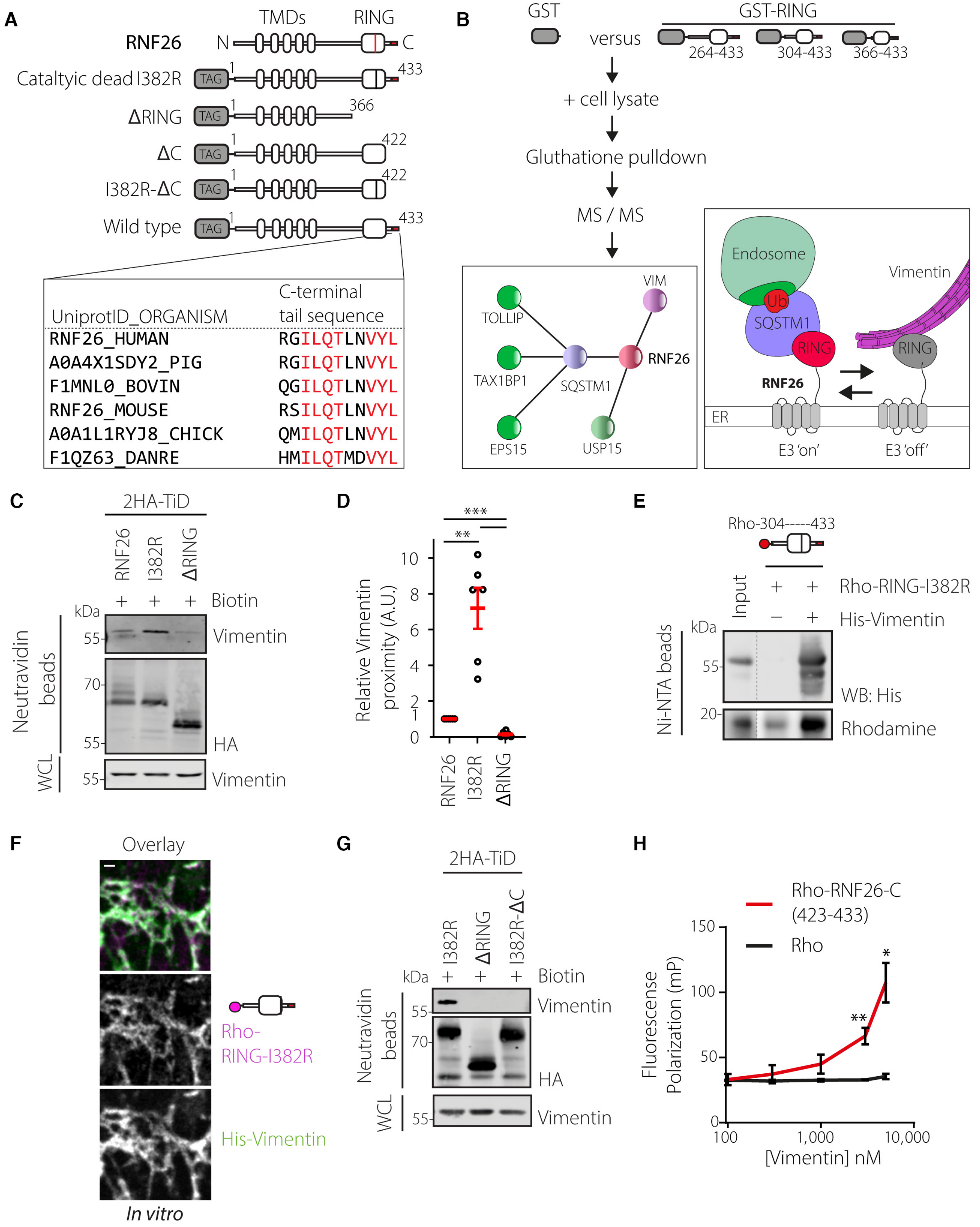
A
TMDs
RING
RNF26
N
C
Catalytic dead I382R
TAG
1
433
ΔRING
366
ΔC
422
I382R-ΔC
Wild type
UniprotID_ORGANISM
C-terminal tail sequence
RNF26_HUMAN RGILQTLNVYL
A0A4X1SDY2_PIG RGILQTLNVYL
F1MNL0_BOVIN QGILQTLNVYL
RNF26_MOUSE RSILQTLNVYL
A0A1L1RYJ8_CHICK QMILQTLNVYL
F1QZ63_DANRE HMILQTMDVYL
B
GST
versus
GST-RING
264-433
304-433
366-433
+ cell lysate
Gluthatione pulldown
MS / MS
VIM
TOLLIP
TAX1BP1
SQSTM1
RNF26
EPS15
USP15
Endosome
Ub
SQSTM1
RING
Vimentin
RNF26
ER
E3 'on'
E3 'off'
C
2HA-TiD
RNF26
I382R
ΔRING
Biotin
kDa
Vimentin
HA
Neutravidin beads
WCL
D
Relative Vimentin proximity (A.U.)
E
Rho-304-----433
Input
Rho-RING-I382R
His-Vimentin
Ni-NTA beads
WB: His
Rhodamine
F
Overlay
Rho-RING-I382R
His-Vimentin
In vitro
G
2HA-TiD
I382R
ΔRING
I382R-ΔC
H
Rho-RNF26-C (423-433)
Rho
Fluorescense Polarization (mP)
[Vimentin] nM

Figure 2.

◀ **Figure 2. RNF26 C-terminus interacts directly with vimentin.**

A Top: Schematic representation of RNF26 domain organization and constructs used for cell-based assays in this study. TMD: transmembrane domains; RING: E3 ubiquitin ligase domain containing I382 (red line) crucial for catalytic activity and C-terminal extension (red bar). Bottom: primary structure homology analysis of C-terminal residues 423–433 of RNF26 in human, pig, bovine, mouse, chicken, and zebrafish species. Red residues indicate full conservation.

B Protein interactome of the RING domain of RNF26. Top: methodology of interactor identification by ms/ms in co-precipitates of recombinant RNF26 fragment GST-RNF26 (304–433) from human melanoma (MelJuSo) cells. Left: select interactors of RNF26 RING domain. Right: working model. Enzymatically active RNF26 ubiquitinates SQSTM1 to recruit endosomal adapters in a ubiquitin-dependent membrane contact site described previously. Conversely, enzymatically inactive RNF26 binds vimentin IFs. See also Table EV1.

C, D Proximity biotinylation of endogenous vimentin by 2HA-TurboID-RNF26, catalytically inactive point mutant (I382R), or RING domain truncation mutant (ΔRING) in HeLa cells following 30-min incubation with biotin prior to lysis. (C) Representative immunoblots of neutravidin precipitates and lysate inputs stained against HA and vimentin. (D) Quantifications of vimentin biotinylation by 2HA-TurboID-RNF26 and mutants normalized to auto biotinylated HA-tagged RNF26 species and expressed relative to RNF26 WT, $n = 6$ independent experiments performed in U2OS and HeLa cells.

E *In vitro* co-precipitation of purified recombinant Rhodamine-labeled RING-I382R (304–433) with purified recombinant His-tagged vimentin on Ni-NTA beads. Rhodamine in-gel fluorescent scan and immunoblot against vimentin are shown, representative of three independent experiments.

F *In vitro* deposition of Rhodamine-RING-I382R (*magenta*) on pre-assembled His-vimentin filaments (*green*). Representative confocal fluorescence images are shown. Scale bar = 1 μm.

G Proximity-based biotinylation of endogenous vimentin by 2HA-TurboID-I382R, -ΔRING, and -I382R-ΔC lacking amino acids 423–433. Immunoblots of neutravidin precipitates and lysate inputs against HA and vimentin are shown and are representative of three independent experiments.

H *In vitro* peptide binding assay. Rhodamine-linked C-terminal peptide of RNF26 (Rho-RGILQTLNVYL) or free Rhodamine were incubated at 10 nM with increasing concentrations of His-vimentin and binding was measured using fluorescence polarization; $n = 3$ independent experiments.

Data information: WCL: whole cell lysate. Graphs in (D) and (H) report mean (red line) and SEM (error bars) of sample values (open circles). Significance was assessed using Student's *t*-test; $^*P < 0.05$, $^{**}P < 0.01$, $^{***}P < 0.001$ (See also Fig EV2).
Source data are available online for this figure.

synthetic peptide of RNF26 containing the C-terminal amino acids 423–433 was observed using fluorescence polarization (Figs 2H and EV2F). In summary, these results indicate that the C-terminal sequence of RNF26 is both necessary and sufficient to support a direct interaction with vimentin and suggest that catalytic inactivity on the part of RNF26 promotes vimentin binding.

Vimentin controls PN localization of RNF26 and the endolysosomal system

Vimentin IFs are commonly associated with structural integrity and mechanical properties of cells. Having identified vimentin as an interactor of RNF26, which concentrates within the PN ER segment (Jongsma *et al*, 2016), we proceeded to investigate whether vimentin influences distribution of RNF26 in the ER membrane. In U2OS cells, the vimentin cytoskeleton takes the form of perinuclear filaments (Fig 3A) and could therefore enable perinuclear anchorage of RNF26. Upon ectopic expression of wild-type (WT) RNF26, partial colocalization with vimentin structures could be observed, which increased appreciably upon expression of catalytically inactive RNF26 (I382R) (Fig 3A and B) in agreement with enhanced complex formation (Fig 2C and D). Also, RNF26-I382R (but not WT) displayed a pronounced filamentous distribution aligned to vimentin fibers (Fig 3A), further supporting the notion that inactive RNF26 more stably associates with vimentin (Fig 2C and D). As expected, removing either the entire RING (ΔRING) domain or only the vimentin-interacting motif (ΔC) from the C-terminus of RNF26 reduced colocalization with vimentin (Fig 3A and B), even though all examined variants of RNF26 continued to reside in VAP-A-positive ER membranes (Fig 3A and C). In line with our hypothesis on the role of vimentin IF filaments as perinuclear anchors for RNF26, we found that perinuclearity of RNF26 was negatively affected upon removal of either its RING domain or the vimentin-interacting motif (Fig 3D). Reciprocally, overexpression of full-length RNF26, especially in its catalytically inactive form, was found to draw vimentin further into the PN area (Fig 3E), while removal of RNF26 caused redistribution of the vimentin network throughout cytoplasmic space (Fig 3F and G) without affecting overall vimentin abundance (Fig 3H). Altogether, these results suggest a spatial codependency between RNF26 and the vimentin cytoskeleton for perinuclear localization.

We next tested whether the presence of vimentin is critical for the perinuclear retention of RNF26 by examining the effects of vimentin ablation. Wild-type RNF26 was found to disperse throughout the ER in vimentin KO cells (Fig 4A and B), resulting in a marked reduction of RNF26 perinuclearity and a corresponding increase in positivity of the general ER marker VAP-A for RNF26 (Fig 4C and D). However, positivity of RNF26 for VAP-A in the same cells remained unchanged (Fig 4D), indicating that RNF26 continued to reside in the ER. In parallel, catalytically inactive RNF26 lost its perinuclear filamentous distribution upon vimentin ablation and formed aggregate-like clusters (Fig EV3A and B) that resembled the appearance of I382R-ΔC mutant of RNF26 in parental cells (Fig EV3C). Aggregates induced by vimentin ablation could in turn be rescued by reintroduction of vimentin into KO cells (Fig EV3A), confirming vimentin dependency. Taken together, these results indicate that vimentin is required for steady-state retention of RNF26 within the PN ER subdomain in both enzymatically active and inactive states.

Since RNF26 informs perinuclear localization of the endolysosomal cloud, we next investigated the impact of vimentin ablation in this context. As predicted, loss of vimentin phenocopied RNF26 silencing, leading to the dispersion of early and late endosomal compartments carrying markers EEA1 (Fig EV3D and E) and LAMP1 (Figs 4E and F, and EV3F–H), respectively. Furthermore, perinuclear arrangement of LAMP1-positive structures, disturbed in response to vimentin ablation, was readily rescued by re-expression of vimentin in vimentin KO cells (Fig 4E and F), indicating that the presence of this cytoskeletal element spatially constrains vesicles of the endolysosomal system. We have previously demonstrated that clustering of endolysosomes in perinuclear space promotes trafficking of endocytosed cargoes toward proteolytic compartments (Jongsma *et al*, 2016). Therefore, we examined whether vimentin influences

 © 2023 The Authors

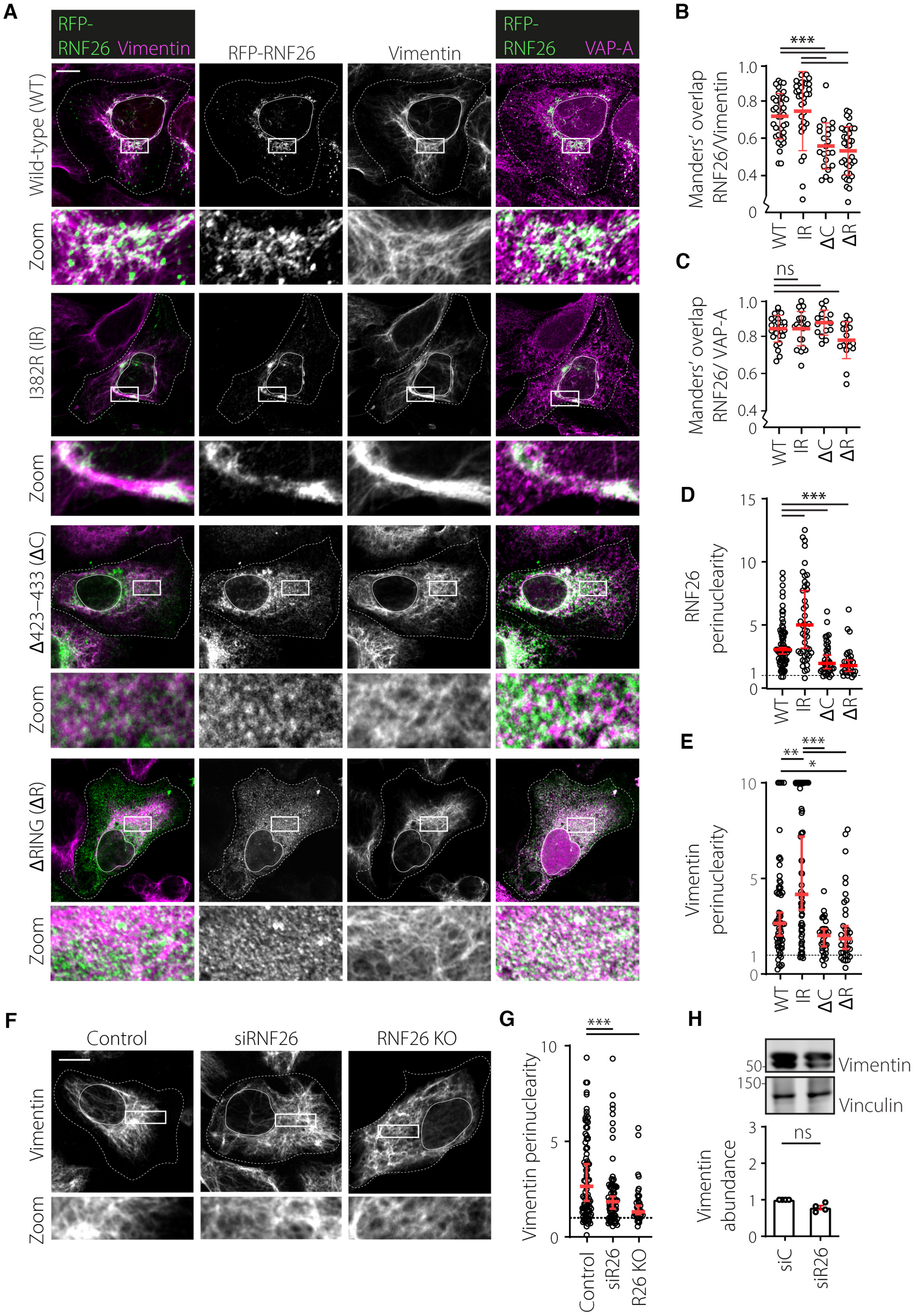
A
RFP-RNF26 Vimentin
RFP-RNF26
Vimentin
RFP-RNF26 VAP-A
Wild-type (WT)
Zoom
I382R (IR)
Zoom
Δ423–433 (ΔC)
Zoom
ΔRING (ΔR)
Zoom
B
***
Manders' overlap RNF26/Vimentin
WT
IR
ΔC
ΔR
C
ns
Manders' overlap RNF26/ VAP-A
D
***
RNF26 perinuclearity
E
**
***
*
Vimentin perinuclearity
F
Control
siRNF26
RNF26 KO
Vimentin
Zoom
G
***
Vimentin perinuclearity
Control
siR26
R26 KO
H
50
150
Vimentin
Vinculin
ns
Vimentin abundance
siC
siR26

Figure 3.

Figure 3. RNF26 colocalizes with the vimentin cytoskeleton via its C-terminal motif.

A–E Analysis of RNF26 sequence determinants with respect to the intracellular distribution of RNF26 and its colocalization with vimentin filaments. (A) Representative confocal fluorescence images of U2OS cells ectopically expressing RFP-RNF26 (*green*) wild-type (WT) or mutants (I382R, ΔRING, ΔC), fixed and immunostained against endogenous vimentin (*magenta*) and VAP-A (*magenta*). Indicated single channels and their corresponding color overlays are shown. Zoom-ins highlight PN regions. (B) Colocalization (Manders') analysis of RFP-RNF26 and mutants overlap with vimentin. Plot reports $n_{WT} = 38$, $n_{I382R} = 31$, $n_{\Delta RING} = 34$, $n_{\Delta C} = 22$ cells analyzed from three independent experiments. (C) Colocalization (Manders') analysis of RFP-RNF26 and mutants with VAP-A. Plot reports $n_{WT} = 29$, $n_{I382R} = 21$, $n_{\Delta RING} = 17$, $n_{\Delta C} = 16$ cells analyzed from three independent experiments. (D) Perinuclearity of RFP-RNF26 as a function of its RING domain determinants. Plot reports $n_{WT} = 72$, $n_{I382R} = 48$, $n_{\Delta RING} = 37$, $n_{\Delta C} = 28$ cells analyzed from three independent experiments. (E) Intracellular distribution of vimentin as a function of RNF26 (mutant) overexpression. Plot reports $n_{WT}59$, $n_{I382R} = 58$, $n_{\Delta RING} = 36$, $n_{\Delta C} = 32$ technical replicates from three independent experiments. Values capped at 10.

F, G Analysis of vimentin cytoskeleton distribution as a function of RNF26. (F) Representative confocal fluorescence images of control cells, cells silenced for RNF26 (si#1), or RNF26 KO cells. Zoom-ins highlight PN regions. (G) Perinuclearity of vimentin in the presence or absence of RNF26. Plot reports $n_{control} = 98$, $n_{siRNF26} = 132$, $n_{RNF26\ KO} = 41$ technical replicates from three independent experiments.

H Vimentin abundance as a function of RNF26. Top: representative immunoblot of U2OS cells transfected with siRNF26#1 stained against endogenous vimentin and Vinculin (loading control). Same samples as in Fig 1I. Bottom: quantification of vimentin abundance, normalized to Vinculin and expressed relative to control (siC = 1.0), $n = 4$ independent experiments.

Data information: Cell and nuclear boundaries are demarcated using dashed and continuous lines, respectively. Scale bar = 10 μm. Statistical analyses were performed using Students' t-test (B, D and H error bars indicate mean ± SD) or Mann–Whitney U test (C, E and G error bars indicate median and 95% confidence interval); $*P < 0.05$, $**P < 0.01$, $***P < 0.001$, ns: not significant.
Source data are available online for this figure.

endosomal traffic by comparing the fate of nascent endosomes in parental and vimentin KO cells. In line with the mislocalization of endolysosomes observed in response to vimentin ablation, transport of newly acquired materials from extracellular space to proteolytic compartments was inhibited under these conditions (Fig 4G and H; Movies EV3 and EV4), signifying that vimentin positively contributes to the spatiotemporal integrity of the endosomal system. Collectively, the data presented in Figs 3 and 4 demonstrate that vimentin retains RNF26 in the PN ER segment and correspondingly facilitates accumulation of endocytic organelles in the perinuclear cloud.

Vimentin regulates ER architecture and dynamics

Our findings discussed thus far suggest that the interplay between the vimentin cytoskeleton and the ER-embedded RNF26 could regulate the integrity of the PN ER subdomain in space and time. Examination of 3D reconstructions of TEM tomograms collected from a perinuclear cell region under steady-state conditions revealed close apposition of ER membranes with intermediate filaments, corresponding to an average distance of about 30 nm (Figs 5A and EV4A–C; Movie EV5). Since the bulk of vimentin filaments accumulates in the PN cytoplasm (Figs 3A and 5B), we reasoned that perinuclear ER membranes could be immobilized onto the vimentin network. In support of this, ER membranes closely associated with vimentin exhibited markedly less mobility than their vimentin-free counterparts (Fig 5B and C; Movie EV6), leading us to examine whether vimentin positively regulates ER organization and dynamics. We noted that removal of vimentin dramatically impacted the PN/PP dichotomy of the ER hallmarked by reduced perinuclearity of mCherry-KDEL-positive membranes (Fig 5D and E) and VAP-A positive ER (Fig EV4D and E), while the total ER occupancy in cellular space remained unaffected (Fig 5F). Furthermore, similar to the phenotype observed with RNF26 depletion (Fig 1B and F), cells lacking vimentin displayed enhanced mobility of the PN ER (Fig 5G; Movies EV7 and EV8), indicating that the presence of this cytoskeletal element restricts PN ER dynamics.

Once again reflecting RNF26 loss-of-function (Figs 1B and G, and EV1G and H), vimentin ablation resulted in pronounced spreading of ER sheets and their associated marker CLIMP63 into the cell periphery (Figs 5D and H, and EV4F and G). Additionally, in contrast to the circumstances of RNF26 depletion (Fig 1B and H), the abundance of ER sheets per cell was also elevated in cells lacking the vimentin cytoskeleton (Fig 5F), without affecting protein levels of CLIMP63 (Fig 5I). These observations suggest that vimentin likely exhibits additional, RNF26-indepent function(s), in ER membrane remodeling. Overall, however, the effects of vimentin ablation resembled those incurred with loss of RNF26, indicating that both RNF26 and vimentin are required to control the spatiotemporal integrity of the ER and confine its sheet-like structures to the perinuclear subdomain.

Vimentin and RNF26 mediate PN ER rearrangements during ER stress

The ER adapts its morphology to accommodate a shifting balance of functions during periods of stress. Cells treated with Tunicamycin to inhibit N-linked glycosylation and subsequent glycoprotein folding undergo a pronounced expansion and coalescence of ER membranes in the PN area (Fig 6A and B). This so-called ER quality control center (ERQC) forms in response to unfolded protein response (UPR) signaling and facilitates segregation and degradation of misfolded ER proteins. Functions carried out at the ERQC are orchestrated by chaperones such as calnexin working in concert with the members of the ERAD machinery, which include the ubiquitin associated protein HERP1 and the E3 ubiquitin ligase HRD1 (Leitman *et al*, 2014). Additionally, it has been demonstrated that PERK-mediated UPR signaling and HERP1 are critical for the establishment of the ERQC (Kondratyev *et al*, 2007). In parallel to the ER, endolysosomal compartments (carrying the late maturation marker CD63) have been described to strongly cluster in the perinuclear region following ER stress (Bae *et al*, 2019). We noted that cells upregulate RNF26 expression in parallel with that of HERP1 in response to Tunicamycin administration (Fig 6C), suggesting a positive role for RNF26 in the ER stress response. Accordingly, we found that ERQC formation strongly depends on the presence of RNF26 and its perinuclear anchor vimentin, as cells lacking either protein failed to concentrate

 © 2023 The Authors

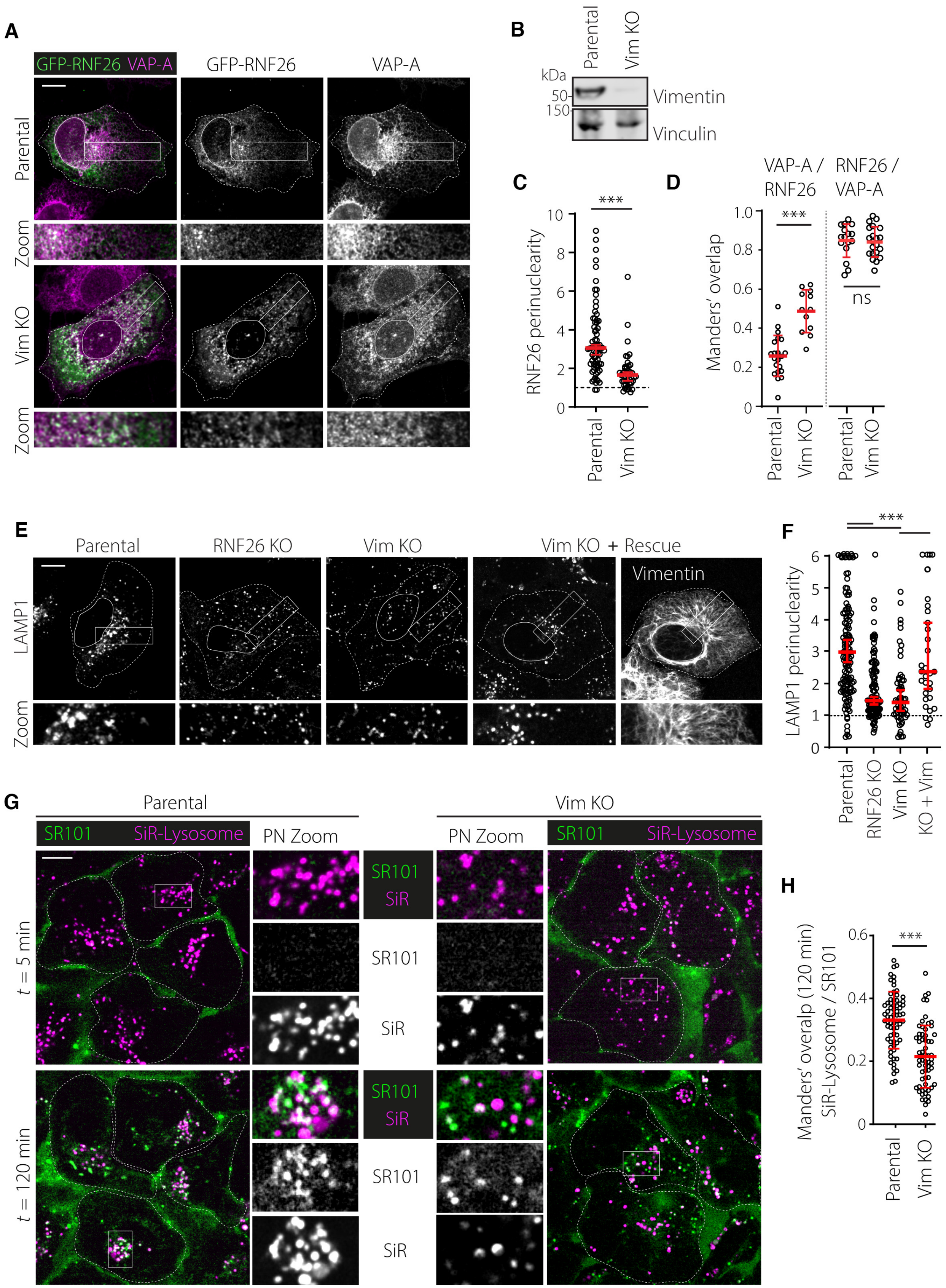
A
GFP-RNF26 VAP-A
GFP-RNF26
VAP-A
Parental
Zoom
Vim KO
Zoom
B
Parental
Vim KO
kDa
50
150
Vimentin
Vinculin
C
RNF26 perinuclearity
***
Parental
Vim KO
D
VAP-A / RNF26
RNF26 / VAP-A
Manders' overlap
***
ns
Parental
Vim KO
Parental
Vim KO
E
Parental
RNF26 KO
Vim KO
Vim KO + Rescue
Vimentin
LAMP1
Zoom
F
***
LAMP1 perinuclearity
Parental
RNF26 KO
Vim KO
KO + Vim
G
Parental
Vim KO
SR101 SiR-Lysosome
PN Zoom
SR101
SiR
t = 5 min
t = 120 min
H
Manders' overalp (120 min) SiR-Lysosome / SR101
***
Parental
Vim KO

Figure 4.

◀ **Figure 4. Vimentin controls perinuclear localization of RNF26 and the endolysosomal system.**

A–D Effect of vimentin ablation on intracellular distribution of RNF26. (A) Representative confocal images of parental and vimentin knockout (Vim KO) U2OS cells ectopically expressing GFP-RNF26 (*green*), fixed and immunostained against endogenous VAP-A (*magenta*). (B) Validation of vimentin knockout. Immunoblots against endogenous vimentin and Vinculin (loading control) of a Vim KO clonal cell line and their parental U2OS cells are shown, representative of three independent experiments. Molecular weight markers are as indicated. (C) Quantification of GFP-RNF26 signal distribution expressed as perinuclearity ratio. Graph reports $n_{\text{Parental}} = 73$, $n_{\text{Vim KO}} = 43$ technical replicates from three independent experiments. (D) Colocalization (Manders') analysis of VAP-A overlapping with GFP-RNF26 (*left plot*) and vice versa (*right plot*). Plots report on subset of cells from data set in (B), same as in (C).

E, F Effect of vimentin or RNF26 ablation on the intracellular distribution of endolysosomes. (E) Representative confocal images of parental, RNF26 KO, vimentin KO #1 U2OS cells, vimentin KO#1 cells ectopically complemented with untagged wild type vimentin (Vim KO + Rescue), fixed and immunostained against endogenous LAMP1. (F) Quantification of LAMP1 signal distribution expressed as perinuclearity ratio. Graph reports on $n_{\text{Parental}} = 145$, $n_{\text{R26 KO}} = 64$, $n_{\text{Vim KO\#1}} = 119$, $n_{\text{Vim KO\#1/Rescue}} = 32$ technical replicates from three independent experiments.Values capped at 6.

G, H Effects of vimentin ablation on trafficking of extracellular materials to lysosomes. (G) Time-lapse imaging. Representative confocal overlays of parental and vimentin KO#2 U2OS cells, stained with SiR-Lysosome (*magenta*) to mark late endocytic compartments and incubated with the cell impermeable dye SR101 (*green*), taken soon after the start ($t = 5$ min) and at the end ($t = 120$ min) of time-lapse are shown. Zoom-ins highlight select PN regions in overlay and single channels. (H) Colocalization (Manders') of SiR-Lysosome overlapping SR101 at $t = 120$ min following SR101 addition. Graph reports on $n_{\text{Parental}} = 71$, $n_{\text{Vim KO}} = 69$ technical replicates from three independent experiments. See also Movies EV3 and EV4.

Data information: Cell and nuclear boundaries are demarcated using dashed and continuous lines, respectively. Zoom-ins designate regions encompassing perinuclear and peripheral areas. All scale bars = 10 μm. Graphs report mean (red line) of sample values (open circles). Statistical analyses were performed using Students' t-test (D and H error bars indicate mean ± SD) or Mann–Whitney U test (C and F error bars indicate median and 95% confidence interval); ***$P < 0.001$, ns: not significant. See also Fig EV3.
Source data are available online for this figure.

calnexin-positive ER membranes in the presence of Tunicamycin (Figs 6D and E, and EV5A and B). The same detrimental effect on ERQC formation was observed with depletion of UBE2J1 (Fig 6D and E), the ubiquitin conjugating enzyme for the ligase activity of RNF26 (Cremer *et al*, 2021). Furthermore, RNF26 depletion rendered cells unable to strongly cluster CD63-positive compartments in the perinuclear region after Tunicamycin treatment (Fig EV5C and D). Since perinuclear localization of endolysosomes relies on RNF26-mediated ubiquitylation (Jongsma *et al*, 2020), these observations imply that perinuclear coalescence of ER membranes and endolysosomes in response to proteotoxic stress benefits from the ubiquitin ligase function of RNF26.

Notably, the absence of either RNF26 or vimentin did not affect Tunicamycin-induced activation of the UPR through either the PERK-eiF2a-ATF4-CHOP axis (Fig 6F and G), IRE1-dependent cleavage of XBP1 mRNA (Fig EV5E), or ATF6-induced upregulation of BiP (Fig EV5F), indicating that inability to assemble the ERQC is not a result of failure to detect ER stress. In fact, Tunicamycin treatment still upregulated HERP1 after removal of RNF26 or vimentin, indicating that these morphological changes rely on RNF26- and vimentin-mediated anchoring of the ER (Fig 6F). In parallel, RNF26 and vimentin appear to generally facilitate stress-induced morphological changes of the ER, as their removal also rendered the ER unresponsive to cyclopiazonic acid-induced calcium deficiency (Fig EV5G). These results reveal that RNF26 is necessary to achieve organellar rearrangements during ER stress in alignment with the vimentin cytoskeleton.

To further characterize the contribution of the vimentin/RNF26 axis to ER physiology, we examined its interplay with the ERQC machinery. Striking colocalization between RNF26 and HERP1 (as well as its homolog HERP2) was observed at discrete ER subdomains, independently of the RING domain of RNF26 (Fig 7A and B). In line with these observations, HERP1 and HRD1 were readily co-isolated with full-length RNF26 (wild-type or catalytically inactive) as well as RNF26 lacking the RING domain (Fig 7C), implying that transmembrane determinants of RNF26 are sufficient to bring it in contact with the key players of the ERQC. This was further substantiated by the observations that HERP1-positive ER subdomains exhibit close juxtaposition to vimentin filaments (Fig 7D and E), and HERP1 perinuclear distribution is facilitated by both vimentin and RNF26 (Fig 7F and G). Taken together with the data presented in Fig 6, these findings indicate that RNF26 and vimentin promote compartmentalization of the ER quality control machinery in response to acute proteotoxic stress.

RNF26 and vimentin are required for efficient Sec62-mediated recovERphagy

Given that RNF26 engages in ER MCSs with proteolytic compartments (Cremer *et al*, 2021) and regulates formation of the ERQC (Fig 6), we considered whether it could facilitate recovERphagy. After resolving ER stress, ER luminal load returns to physiological levels through the disposal of ER chaperones in endolysosomes (Fumagalli *et al*, 2016). Here, endolysosomes directly engulf ER subdomains decorated with idle Sec62 ERphagy receptors, without the need for an intermediate step of autophagosome formation (Loi *et al*, 2019). Sec62-GFP locates to ERQC subdomains formed by HERP1 overexpression, some of which are juxtaposed to CD63-positive late compartments (Fig 8A), indicating that recovERphagy originates from pre-established ERQC domains. Since vimentin and RNF26 enforce positioning of endolysosomes and organize perinuclear ERQC domains, we hypothesized that these proteins take part in the recovERphagy flux. To visualize this, Sec62 fused to a tandem HALO-GFP tag (Sec62-HALO-GFP) was followed into acidic compartments, where GFP fluorescence becomes quenched, while the HALO-tag fragment sensitive to lysosomal inhibitors remains fluorescent (Fig 8B and C) (Rudinskiy *et al*, 2022). Quantification of the HALO-tag signal by in-gel fluorescence revealed that complete removal of either RNF26 or vimentin reduced lysosomal conversion of full-length Sec62 (Fig 8C and D). Accordingly, CD63-positive late endosomal compartments remained largely unoccupied by HALO-positive signal following a 7-h chase period in the presence of Bafilomycin under conditions of either RNF26 or vimentin ablation (Fig 8E and F). Furthermore, while both wild-type and inactive RNF26 variants colocalized with Sec62, only catalytically competent RNF26 trafficked along with Sec62-HALO-GFP into proteolytic

 © 2023 The Authors

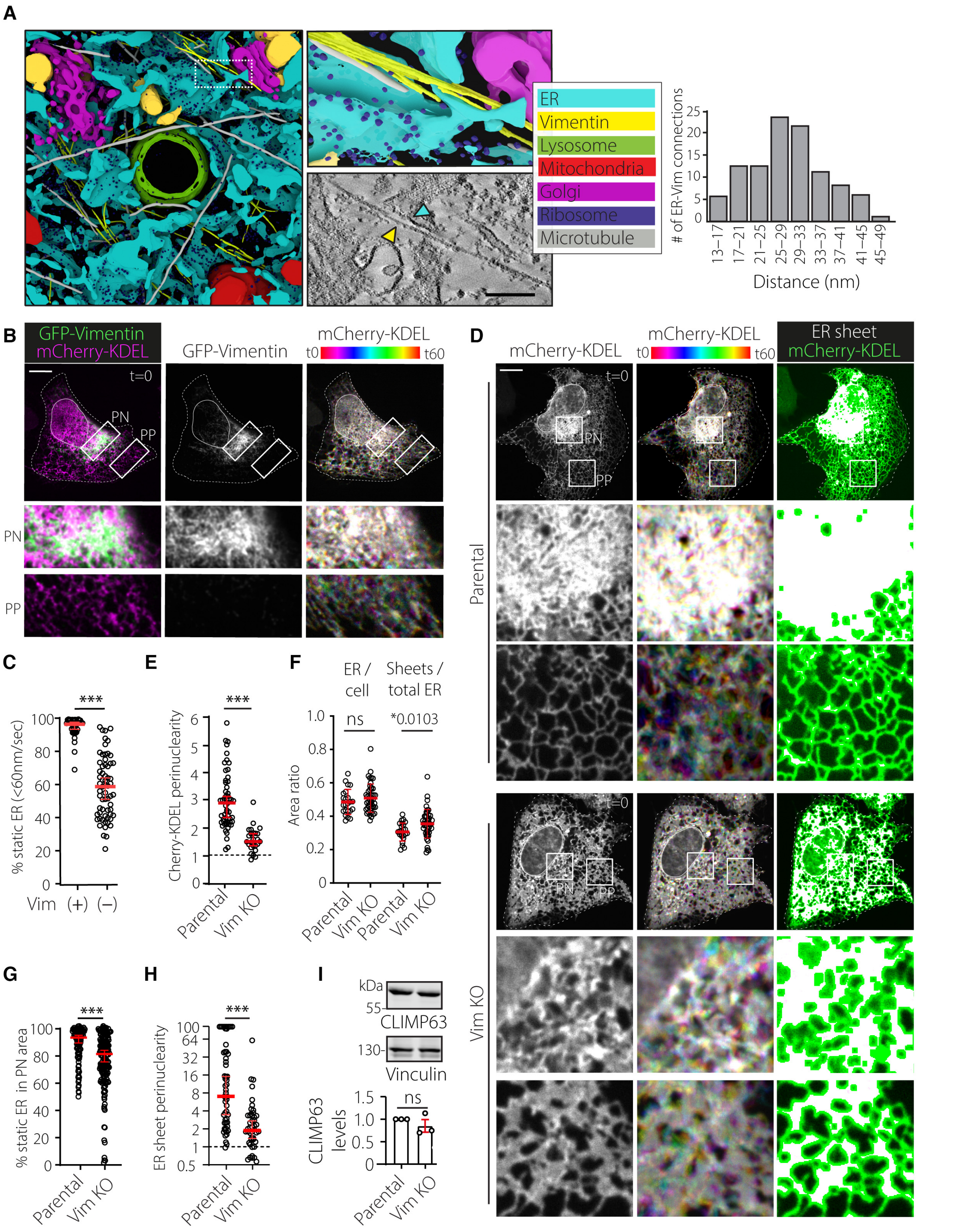
A
ER
Vimentin
Lysosome
Mitochondria
Golgi
Ribosome
Microtubule
# of ER-Vim connections
Distance (nm)
13–17
17–21
21–25
25–29
29–33
33–37
37–41
41–45
45–49
B
GFP-Vimentin
mCherry-KDEL
t=0
PN
PP
t0
t60
D
ER sheet
Parental
Vim KO
C
% static ER (<60nm/sec)
Vim (+) (–)
***
E
Cherry-KDEL perinuclearity
Parental
Vim KO
F
ER / cell
Sheets / total ER
ns
*0.0103
Area ratio
G
% static ER in PN area
H
ER sheet perinuclearity
I
kDa
55
130
CLIMP63
Vinculin
CLIMP63 levels

Figure 5.

◀ **Figure 5. Vimentin controls perinuclear ER integrity and dynamics.**

A Ultrastructure of the perinuclear region. Left: 3D rendering of a PN area from a U2OS cell, constructed from tomograms of three serial EM sections. Manual color annotation of organelles is provided in the legend. Zoom-ins of renders and tomogram slices highlight a long bundle of filaments juxtaposed to ER membranes. Right: Quantification of vimentin-ER contacts found in EM tomography. Graph depicts 100 measurements from 16 tomograms within five serial sections where shortest distances were measured on positions where vimentin (yellow) was running parallel to the ER (turquoise) and where a density between the two was present. See also Movie EV5. Scale bar = 100 nm.

B, C Spatiotemporal relationship between the ER and vimentin cytoskeleton. (B) Time-lapse imaging. A representative U2OS cell ectopically expressing mCherry-KDEL (*magenta*) and GFP-VHH-vimentin nanobody (*green*) is shown. Left panels: confocal fluorescence image overlays at $t = 0$. Middle panels: single channel image of GFP-VHH-vimentin nanobody (*white*) at $t = 0$. Right panels: time color coded overlays of 60x1s frames. Zoom-ins highlight select cell regions spanning perinuclear and peripheral areas. Scale bar = 10 μm. (C) Quantification of Cherry-KDEL-positive membrane dynamics in vimentin positive (+) ER versus negative (–) regions within the same cells. Graph reports on 3 ≥ ROIs PN or PP in $n = 5$ from three independent experiments. See also Movie EV6.

D–H Effect of vimentin ablation on ER organization and dynamics. Analysis of parental versus vimentin KO#1 U2OS cells ectopically expressing mCherry-KDEL. (D) Time-lapse imaging. Left panels: representative confocal fluorescence images of Cherry-KDEL (*white*) at the start of time-lapse $t = 0$ (top). Middle panels: time color coded (TCC) overlays of 60x1s frames. Right panels: overlays of twice eroded ER sheet masks (*magenta*) with mCherry-KDEL signal (*green*). Masks were generated using ImageJ software. Zoom-ins show select PN and PP regions. Scale bar = 10 μm. (E) Quantification of Cherry-KDEL distribution expressed as perinuclearity ratio. Graph reports on $n_{PARENTAL} = 89$, $n_{Vim\ KO} = 54$ cells analyzed from three independent experiments. (F) Quantification of ER occupancy or ER sheet (area) between the nucleus and plasma membrane defined in (D). ER area was calculated using particle analysis on thresholded Cherry-KDEL signal using ImageJ. Graph reports on $n_{Parental} = 23$, $n_{Vim\ KO} = 45$ cells from three independent experiments from (E). (G) Quantification of perinuclear Cherry-KDEL-positive membrane dynamics. Graph reports on $n_{Parental} = 16$, $n_{Vim\ KO} = 26$ cells (4 ≥ ROIs analyzed per cell) from three independent experiments. (H) Quantification of ER sheet mask distribution expressed as perinuclearity ratio. Graph reports on $n_{Parental} = 59$, $n_{Vim\ KO} = 48$ cells analyzed from three independent experiments. Values capped at 100. See also Movies EV7 and EV8.

I CLIMP63 abundance as a function of vimentin knockout. Top: immunoblots of parental versus vimentin KO#1 U2OS cell against endogenous CLIMP63 and Vinculin (loading control), representative of three independent experiments. Molecular weight markers are as indicated. Bottom: quantification of CLIMP63 abundance, normalized to Vinculin levels and expressed relative to parental from three independent experiments.

Data information: Cell and nuclear boundaries are demarcated using dashed and continuous lines, respectively. For TCC, each assigned a different color LUT (white—same ER location across all frames; color—ER movement between frames). Movement of Cherry-KDEL-positive pixels in each successive frame in 5 × 5 μm ROIs was binned into static (0–60 nm/s) or dynamic (61–780 nm/s) vectors using membrane displacement analysis (MDA) for Fiji. Graphs report mean or median (red line) of sample values (open circles). Statistical analyses were performed using Students' t-test (F and I; error bars indicate mean ± SD) or Mann–Whitney U test (C, E, G, and H error bars indicate median and 95% confidence interval); *$P < 0.05$, ***$P < 0.001$, ns: not significant. See also Fig EV4.
Source data are available online for this figure.

compartments (Fig 8G), and engulfment of Sec62-positive membranes by lysosomes proved sensitive to ubiquitin ligase activity of RNF26 (Fig 8G and H). Together with the data presented in Figs 6 and 7, these results imply that RNF26 and vimentin help coalesce ERQC domains with endolysosomes for efficient Sec62-dependent recovERphagy. Collectively, our findings demonstrate that ERQC compartmentalization within the ER membrane is achieved through anchorage upon the vimentin cytoskeleton, which constitutes a physiologically relevant aspect of this organelle's behavior and function.

Discussion

Transport and anchorage of organelles in cellular space relies on a multitude of factors, notably including various types of cytoskeletal elements. While the roles of the microtubule and actin networks with respect to organellar organization and motility have been extensively studied (Reck-Peterson *et al*, 2018; Svitkina, 2018; Zheng *et al*, 2022), contributions of intermediate filaments in this context are only beginning to come to light (Etienne-Manneville, 2018). Furthermore, in addition to the cytoskeleton, physical interactions between membrane-enclosed compartments have emerged as important regulators of organelle behavior (Wu *et al*, 2018; Prinz *et al*, 2020). How these different modes of spatiotemporal control are integrated to deliver and retain organelles at their site of function remains unclear.

Here, we report that the vimentin cytoskeleton is instrumental for the spatiotemporal integrity of biosynthetic and proteolytic organelles. Vimentin filaments have been previously described to function in cellular stiffness and mechanical support during global rearrangements, such as cell migration (Toivola *et al*, 2005; Lowery *et al*, 2015; Patteson *et al*, 2019). Additionally, vimentin filaments have also been reported to serve as anchors for intracellular organelles including the nucleus (Ketema *et al*, 2013), mitochondria (Tang *et al*, 2008; Nekrasova *et al*, 2011), lipid droplets (Franke *et al*, 1987), ERGIC/Golgi (Gao *et al*, 2002; Risco *et al*, 2002), and melanosomes (Gao *et al*, 2002; Risco *et al*, 2002; Chang *et al*, 2009). Our current findings demonstrate that vimentin IFs anchor the ER network and endolysosomes in cellular space and, in so doing, regulate membrane dynamics and functional interplay between these organelles. We show that vimentin partners with RNF26 embedded in the ER membrane and that both proteins facilitate compartmentalization of this expansive organelle between its denser, less mobile perinuclear segment and the sparser, highly dynamic periphery. Loss of either RNF26 or vimentin results in the homogenization of ER membrane constituents and alters perinuclear ER dynamics. The same perturbations disorganize the endolysosomal system and inhibit maturation of endocytosed materials toward proteolytic compartments. Lastly, under conditions of proteotoxic stress, the vimentin/RNF26 regulatory axis facilitates acute perinuclear coalescence of ER membranes to form the ERQC and promotes the Sec62-mediated recovERphagy pathway. Collectively, our observations place a third cytoskeletal component—the intermediate filament—in control of organellar organization and behavior with implications for stress response.

A hallmark of perturbed ER architecture incurred with loss of either vimentin or RNF26 is the spreading of normally perinuclearly retained ER sheets to the cell periphery. We also find that vimentin ablation increases the overall abundance of ER sheets, which is not

 © 2023 The Authors

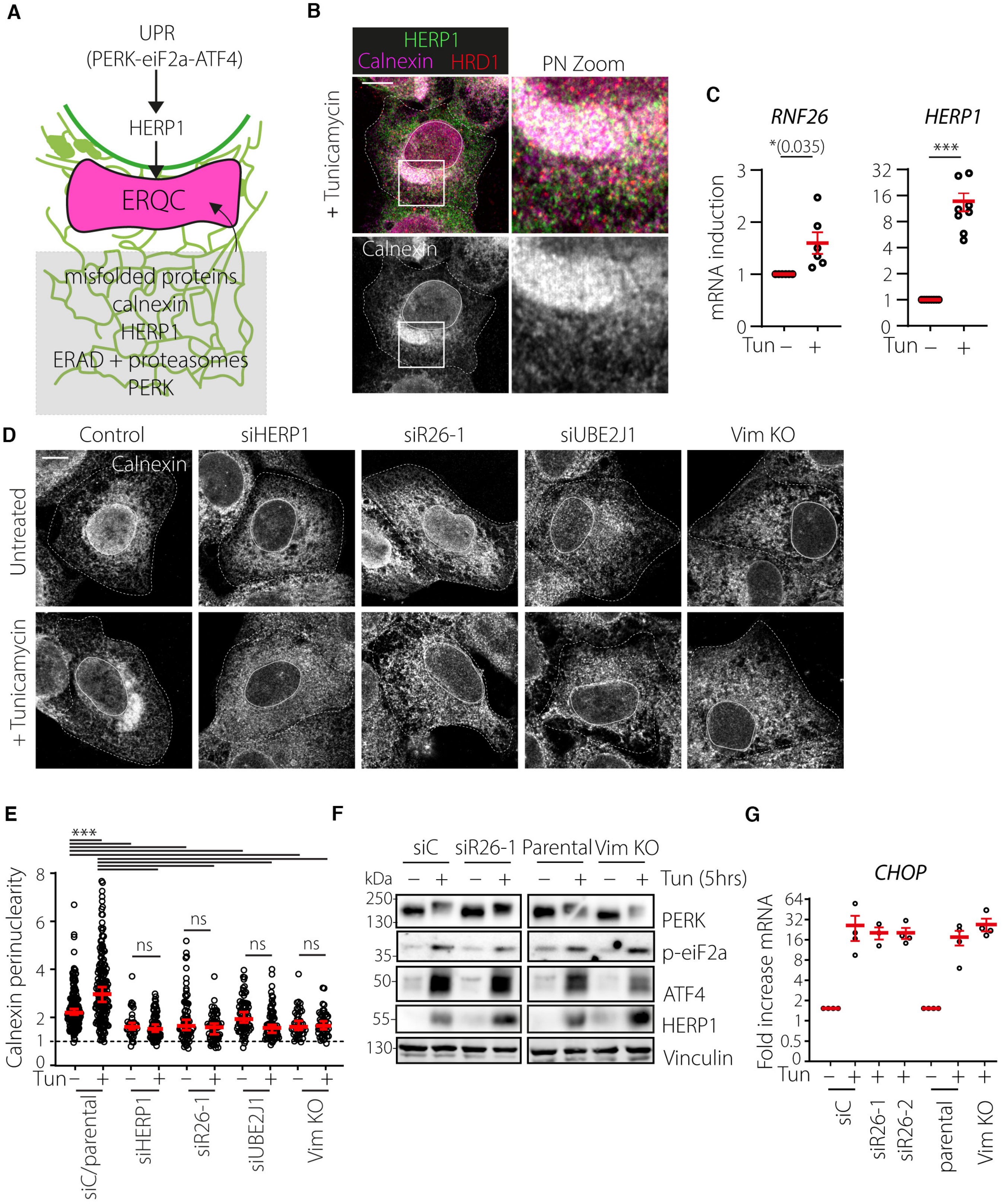
A
UPR
(PERK-eiF2a-ATF4)
HERP1
ERQC
misfolded proteins
calnexin
HERP1
ERAD + proteasomes
PERK
B
HERP1
Calnexin HRD1
PN Zoom
+ Tunicamycin
Calnexin
C
RNF26
*(0.035)
HERP1
***
mRNA induction
Tun – +
D
Control
siHERP1
siR26-1
siUBE2J1
Vim KO
Calnexin
Untreated
+ Tunicamycin
E
***
ns
Calnexin perinuclearity
Tun – +
siC/parental
siHERP1
siR26-1
siUBE2J1
Vim KO
F
siC
siR26-1
Parental
Vim KO
kDa
Tun (5hrs)
PERK
p-eiF2a
ATF4
HERP1
Vinculin
G
CHOP
Fold increase mRNA
Tun
siC
siR26-1
siR26-2
parental
Vim KO

Figure 6.

Figure 6. RNF26 and vimentin control ER reorganization during ER stress.

A, B Proteotoxic stress and the ER quality control center (ERQC). (A) Schematic representation of ERQC formation in response to ER stress. Accumulation of misfolded proteins is sensed through the unfolded protein response (UPR) signaling pathway, which in turn drives morphological changes in the ER membrane. Misfolded proteins are compartmentalized at the perinuclear ERQC harboring chaperones (Calnexin), UPR signaling members (PERK and IRE1) and components of ER-associated protein degradation (ERAD) machinery (HRD1 and HERP1). Formation of ERQC requires PERK and HERP1. (B) A representative confocal overlay of fixed U2OS cells treated with tunicamycin (2.5 µg/ml, 16 h), fixed and immunostained against endogenous HERP1 (*green*), calnexin (*magenta*) and HRD1 (*red*).

C Induction of RNF26 and HERP1 transcripts following tunicamycin treatment. RNF26 (*left*) and HERP1 (*right*) mRNA isolated from U2OS cells treated overnight in the presence (+) or absence (−) of tunicamycin (2.5 µg/ml) was detected by qPCR and normalized to GAPDH. Graph reports on $n_{RNF26} = 6$, $n_{HERP1} = 8$ independent experiments.

D, E Consequences of RNF26, UBE2J1, and vimentin loss on ERQC formation. (D) Representative confocal images of fixed U2OS cells transfected or genetically modified as indicated, treated in the absence (−) or presence (+) of tunicamycin (5 µg/ml, O/N), fixed and immunostained against endogenous calnexin (*white*). (E) Quantification of calnexin distribution expressed as perinuclearity ratio. Graph reports on $n_{control\ (-)} = 168$, $n_{control\ (+)} = 178$, $n_{siHERP1\ (-)} = 36$, $n_{siHERP1\ (+)} = 74$, $n_{siRNF26\ (-)} = 73$, $n_{siRNF26\ (+)} = 61$, $n_{siUBE2J1\ (-)} = 73$, $n_{siUBE2J1\ (+)} = 77$, $n_{Vim\ KO\ (-)} = 43$, $n_{Vim\ KO\ (+)} = 48$ technical replicates from three independent experiments.

F, G Consequences of RNF26, UBE2J1, and vimentin loss on induction of PERK-mediated UPR signaling by tunicamycin. (F) U2OS cells, transfected or genetically modified as indicated, were treated in the absence (−) or presence (+) of tunicamycin (2.5 µg/ml, 5 h). Immunoblots of whole cell lysates against endogenous PERK, phospho-eIF2a, ATF4, HERP1, and Vinculin (loading control) are shown, representative of three independent experiments. (G) CHOP transcripts isolated from U2OS cells transfected or genetically modified as indicated and treated in the absence (−) or presence (+) of tunicamycin (2.5 µg/ml, O/N) were detected by qPCR and normalized to GAPDH; $n_{siC\ (-)} = 4$, $n_{siC\ (+)} = 4$, $n_{siRNF26\#1\ (+)} = 3$, $n_{siRNF26\#2\ (+)} = 4$, $n_{PARENTAL\ (+)} = 4$, $n_{Vim\ KO\ (+)} = 4$ independent experiments.

Data information: Cell and nuclear boundaries are demarcated using dashed and continuous lines, respectively. Scale bar = 10 µm. Graphs report mean or median (red line) of sample values (open circles). Statistical analyses were performed using Students' t-test (C and G error bars indicate mean ± SEM) or Mann–Whitney U test (E error bars indicate median and 95% confidence interval); $*P < 0.05$, $***P < 0.001$ (See also Fig EV4).
Source data are available online for this figure.

the case under conditions of RNF26 loss. This implies the involvement of additional factors that connect the ER to the vimentin cytoskeleton. In line with this, RRBP1/p180 has previously been shown to bind both microtubules and vimentin IFs with consequences for the organization of the perinuclear ER (Ogawa-Goto *et al*, 2007; Zheng *et al*, 2022). Dispersion of ER membranes from the perinuclear area has also been documented in response to deregulated microtubules or actin fibers (Konietzny *et al*, 2022; Zheng *et al*, 2022). Similarly, IFs could be envisioned to organize different ER subdomains through selective engagement of ER-associated binding partners. Relative contribution of different cytoskeletal elements to perinuclear retention of organelles may, however, be cell type dependent, as for instance epithelial cells do not appreciably express vimentin. These cells may instead achieve ER compartmentalization using other types of IFs or compensate through continuous dynein-mediated transport to sustain perinuclear clustering of biosynthetic and proteolytic organelles (Reck-Peterson *et al*, 2018). Furthermore, while we find that vimentin filaments are determinant of RNF26 localization and perinuclear ER identity, overexpression of RNF26 in turn appears to affect the distribution of vimentin. This back-and-forth feeds into the paradigm of two-way signaling between the cytoskeleton and the ER previously postulated in the context of the microtubule network (Farias *et al*, 2019; Tikhomirova *et al*, 2022). Generally, the interplay between vimentin and microtubules (Hookway *et al*, 2015; Gan *et al*, 2016; Schaedel *et al*, 2021) or actin filaments (Jiu *et al*, 2015; Duarte *et al*, 2019; Serres *et al*, 2020) underscores the complexity of the cell's cytoskeletal foundations. In addition, sensitivity of vimentin filaments to electrophilic and oxidative reactions (Viedma-Poyatos *et al*, 2020), as well as post-translational modifications including phosphorylation (Sihag *et al*, 2007), presents a wide range of opportunities for demand-based regulation of organellar architecture through cytoskeletal alterations.

We have previously demonstrated that ubiquitylation mediated by the RING domain of RNF26 retains endosomes and lysosomes in a perinuclear cloud through engagement of their associated adaptor proteins at (reversible) ER MCSs (Jongsma *et al*, 2016; Cremer *et al*, 2021). We now show that RNF26 employs its extreme C-

Figure 7. Interplay between RNF26, vimentin and HERP1 in the perinuclear ER.

A, B Analysis of localization of RNF26 sequence variants and HERP1 or HERP2 in the ER. (A) Representative confocal images of U2OS cells transfected with GFP-RNF26 WT, RFP-RNF26 I382R, GFP-RNF26 ΔRING and HERP1 or GFP-RNF26 WT with HA-HERP2 that were stained for HA and endogenous VAP-A. Indicated single channels and their corresponding color overlays are shown. Zoom-ins highlight PN regions. (B) Line graph analysis of RNF26 WT (green), HA-HERP1 (magenta) and VAP-A (red) signals from (A) (overlay zoom image).

C Analysis of determinants for RNF26 complex formation with ERQC proteins HERP1 and HRD1. U2OS cells were transfected with RFP-RNF26 variants (WT, I382R, ΔRING) or EV with either HA-HERP1 or HA-HRD1 before lysis in 1% DMNG detergent, pulldown with RFP-TRAP beads and analysis by SDS–PAGE and WB. Representative images of three independent replicates.

D, E Analysis of localization of ERQC domains with vimentin. (D) Representative confocal images of U2OS cells transfected with HA-HERP1 that were stained for endogenous vimentin. Indicated single channels and their corresponding color overlays are shown. Zoom-ins highlight PN regions where vimentin surrounds HERP1-labeled ERQC domains. (E) Line graph analysis of HA-HERP1 (magenta) and vimentin (green) signals from (E) (bottom zoom image).

F, G Analysis of HERP1-induced ERQC domain localization as a function of vimentin and RNF26. (F) Representative confocal fluorescence images of control cells, RNF26 KO cells, or vimentin KO#2 cells, transfected with HA-HERP1 and immunostained for HA. Zoom-ins highlight PN regions. (G) Perinuclearity analysis of HA-HERP1 in the presence or absence of RNF26 or vimentin. Plot reports median (red line) and 95% confidence interval (error bar) of sample values (open circle), $n_{Parental} = 79$, $n_{R26\ KO} = 51$, $n_{Vim\ KO} = 45$ technical replicates from three independent experiments (Mann–Whitney U test; $**P < 0.01$, $***P < 0.001$). Graphs report median (red line) and 95% confidence interval (error bars) of sample values (open circles).

Data information: Cell and nuclear boundaries are demarcated using dashed and continuous lines, respectively. Scale bar = 10 µm.
Source data are available online for this figure.

 © 2023 The Authors

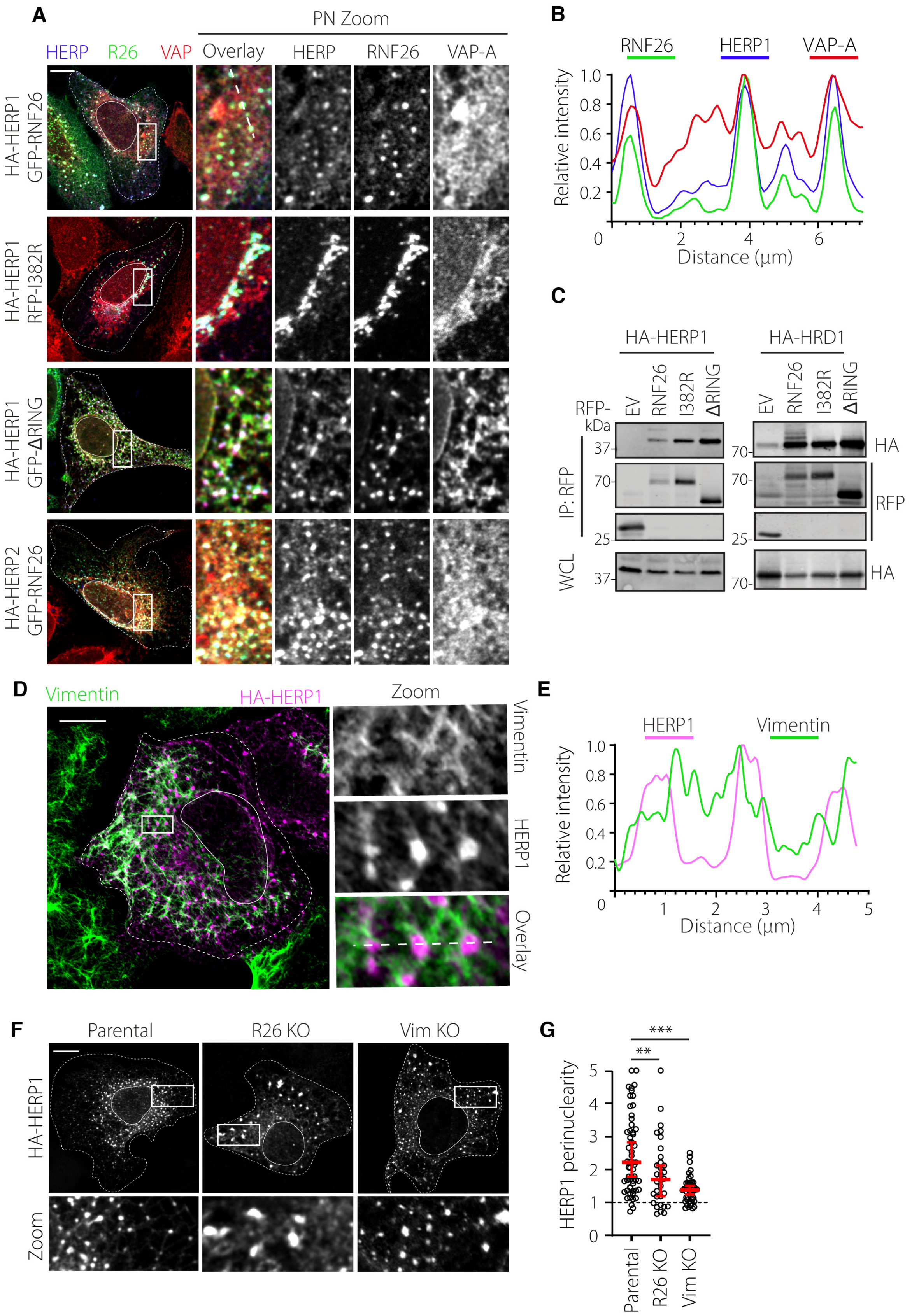
A
PN Zoom
HERP
R26
VAP
Overlay
HERP
RNF26
VAP-A
HA-HERP1 GFP-RNF26
HA-HERP1 RFP-I382R
HA-HERP1 GFP-ΔRING
HA-HERP2 GFP-RNF26
B
RNF26
HERP1
VAP-A
Relative intensity
Distance (μm)
C
HA-HERP1
HA-HRD1
RFP-
kDa
EV
RNF26
I382R
ΔRING
HA
RFP
IP: RFP
WCL
D
Vimentin
HA-HERP1
Zoom
Vimentin
HERP1
Overlay
E
HERP1
Vimentin
Relative intensity
Distance (μm)
F
Parental
R26 KO
Vim KO
HA-HERP1
Zoom
G
HERP1 perinuclearity
Parental
R26 KO
Vim KO

Figure 7.

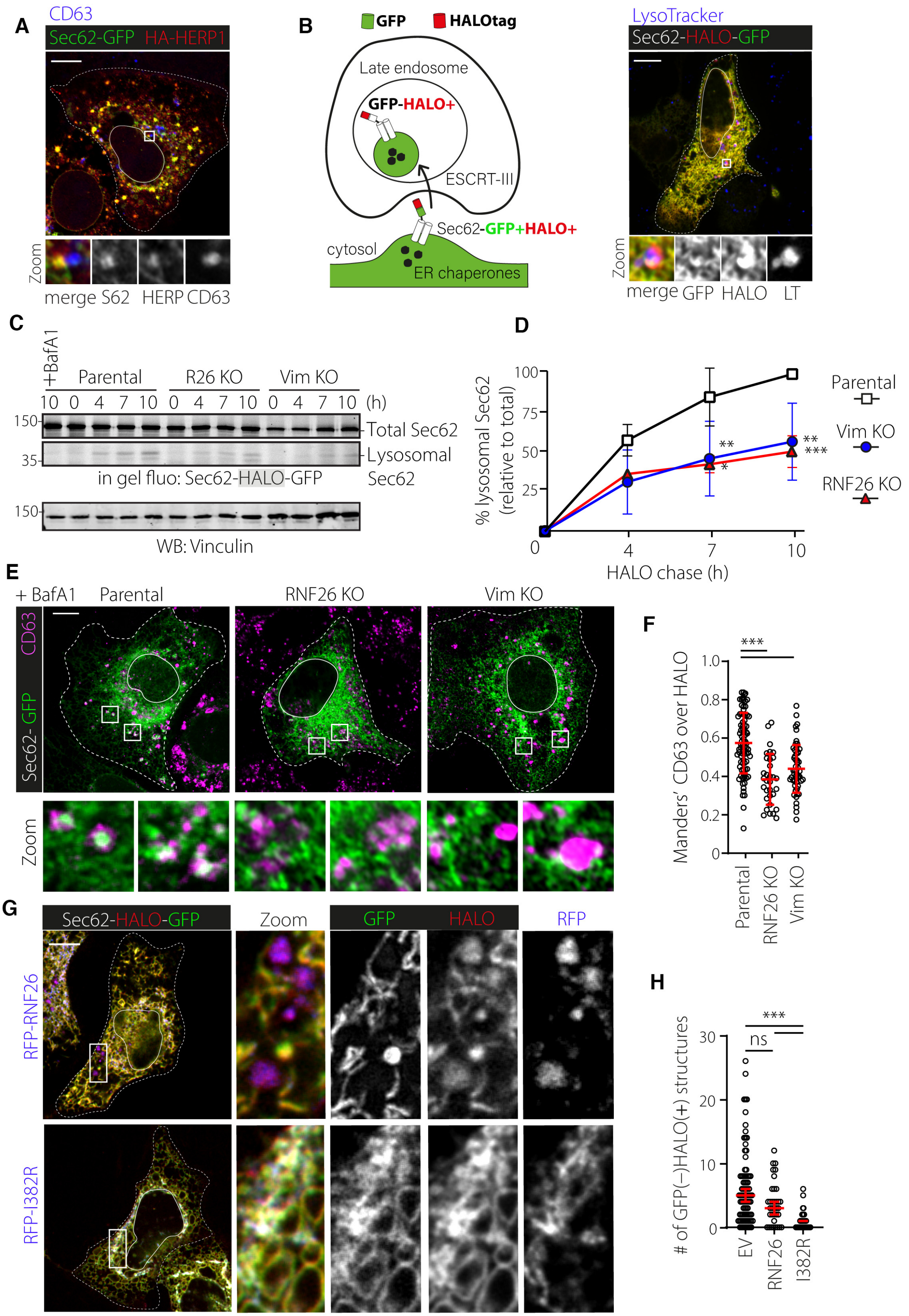

Figure 8.

 © 2023 The Authors

Figure 8. RNF26 and vimentin control Sec62-mediated transfer of ERQC domains to late endosomes.

A Coalescence of ERQC domains with Sec62 that are juxtaposed to late endosomes in U2OS cells. Cells were transfected overnight with Sec62-HALO-GFP and HA-HERP1 and stained for CD63 and HA before imaging by Airyscan microscopy. Boxed zoom-ins show merged and single channel images of three examples of juxtapositioning of HERP1- and Sec62-domains to CD63-positive late endosomes.

B (Left) Schematic representation of transfer of ER chaperones such as Calnexin to lysosomes as part of ER stress recovery (or recovERphagy). In short, removal of ER stressors frees Sec62 from the translocon complex after which Sec62 selects ER chaperones into ER domains that are engulfed into late endosomes in an ESCRT-dependent manner. RecovERphagy is induced by overexpressed Sec62-HALO-GFP, which can be followed into proteolytic compartments where GFP is degraded, but a protease-resistant fluorescent HALO-tag fragment accumulates over time. (Right) Live cell snapshot of a U2OS cell expressing Sec62-HALO-GFP, labeled with JaneliaFluor 646 for 5 h and LysoTracker (LT) prior to imaging. Overlay images show overlap of GFP (green), HALOtag (magenta), and LT (red) signals and zooms also provide single channel images.

C, D Lysosomal processing of Sec62-HALO-GFP over time in the presence or absence of RNF26 or vimentin. (C) Parental, Vim KO#2 or RNF26 KO U2OS cells were transfected with Sec62-HALO-GFP overnight, incubated with 6-chlorohexanol for 15 min, washed three times with PBS, pulsed with TAMRA HALOtag ligand (200 nM) for 1 h, and incubated with 6-chlorohexanol with or without Bafilomycin A1 (100 nM) for the indicated times before cell lysis and analysis by SDS–PAGE. Shown is a representative in-gel fluorescence scan with indication of full-length Sec62, protease-resistant HALO fragment and molecular weight markers (MW), as well as a Vinculin blot (loading control) (D). Quantification of Sec62 accumulation in LEs from (B). Unprocessed and protease-resistant (lysosomal) band intensities in (B) were calculated with ImageJ and ratios of lysosomal Sec62 versus unprocessed Sec62 were normalized to transfer in parental cells after 10 h. Error bars show mean ± SD of $n_{\text{Parental}} = 6$, $n_{\text{R26 KO}} = 4$, $n_{\text{Vim KO}} = 6$ independent experiments.

E, F Sec62-mediated transfer of ER to lysosomes as a function of RNF26 or vimentin. (E) Representative Airyscan images of Parental, RNF26 KO or Vim KO#2 U2OS cells that were transfected with Sec62-HALO-GFP, incubated with 6-chlorohexanol for 15 min, washed three times with PBS, pulsed with JF646 HALOtag ligand for 1 h, and incubated with 6-chlorohexanol and Bafilomycin A1 for 8 h before fixation and immunostaining for CD63 to visualize LEs. (F) Colocalization (Manders') analysis of CD63 over HALO signals from (D). Plot reports $n_{\text{Parental}} = 106$, $n_{\text{R26 KO}} = 69$, $n_{\text{Vim KO}} = 68$ technical replicates from three independent experiments.

G, H Sec62-mediated transfer of ER to lysosomes as a function of RNF26 overexpression and activity. (G) Representative spinning disk live cell images of RNF26 WT and I382R inactive mutant with Sec62. U2OS cells were transfected overnight with Sec62-HALO-GFP and RFP-RNF26 WT or I382R, incubated for 5 h with JF646 and imaged by spinning disk microscopy. Images show overlay of GFP (green), JF646 (magenta), and RFP signals, zoom-ins of overlay and single channels that show transfer of RNF26 into LEs (WT RNF26). (H) Analysis of Sec62 transfer to proteolytic compartments as a function of RNF26 WT or I382R overexpression. Number of GFP-negative/HALO-positive signals was manually counted from cells in (B) and (G). Plot reports $n_{\text{EV}} = 86$, $n_{\text{R26 WT}} = 35$, $n_{\text{R26 IR}} = 40$ technical replicates from three independent experiments.

Data information: Cell and nuclear boundaries are demarcated using dashed and continuous lines, respectively. Scale bar = 10 µm. Graphs report mean (in D and F) or median (in H) of sample values (open circles). Statistical analyses were performed using Students' t-test ((D) (paired) and (F) (unpaired) and Mann–Whitney U test (H); *$P < 0.05$, **$P < 0.01$, ***$P < 0.001$).
Source data are available online for this figure.

terminus to bind directly to vimentin filaments, and that this interaction becomes strengthened under conditions of ubiquitin ligase inactivity. The resulting dynamic interplay between vimentin, RNF26, and endosomal adaptors supports functional integration of biosynthetic and proteolytic organelles. Collectively, these findings prompt the question of how RNF26 activity is regulated to allow selective engagement of different binding partners. RNF26 has been shown to reside in two nearly identical protein complexes, only one of which is supportive of (auto)ubiquitylation (Fenech *et al*, 2020). This implies that specific interactions taking place within the ER membrane could regulate the activity status of RNF26. For instance, RNF26 can engage two ER-associated conjugating enzymes, UBE2J1 and UBE2J2, where the former complex results in productive substrate ubiquitylation, while the latter does not (Cremer *et al*, 2021). As E2–E3 interactions are notoriously transient, availability of these ER-embedded E2s could influence preference for ER-endolysosome versus ER-cytoskeleton interactions. In this case, idle or UBE2J2-bound RNF26 could mimic the ligase inactive state, allowing for stronger vimentin binding and maintenance of perinuclear ER identity, while the catalytically active UBE2J1/RNF26 complex would instead favor ER-endolysosome MCS formation, resulting in perinuclear accumulation of endolysosomes. Association of RNF26 with ER transmembrane proteins HERP1 and HERP2 (Fenech *et al*, 2020) with partially redundant functions could offer another avenue for controlling RNF26 ligase activity, since expression of HERP1 is ER stress-induced, while that of HERP2 is constitutive (Yamamoto *et al*, 2004; Huang *et al*, 2014). Because HERP1 is instrumental to the organization of ERQC protein complexes (including the aforementioned E2 enzymes) and modulation of their attendant E3 activities (Kny *et al*, 2011; Leitman *et al*, 2014; Schulz *et al*, 2017), relative abundance of HERP1 and HERP2 within the perinuclear ER subdomain could be envisioned to fine-tune RNF26 function from steady state to ER stress conditions.

Upon ER stress, RNF26 is recruited into the ERQC, inducing perinuclear coalescence of ER membranes with proteolytic endolysosomes. Notably, the ERQC is home to various E3 ubiquitin ligases governing proteasomal degradation of surplus and misfolded ER proteins through the conventional ERAD pathway (Oikonomou & Hendershot, 2020; Christianson & Carvalho, 2022). Meanwhile, perinuclear integration of ERQC domains with proteolytic organelles facilitates lysosomal clearance of ERQC components through Sec62-mediated ERphagy, which we now show is reliant upon RNF26 and vimentin. Given that this type of ERphagy is mediated by the ESCRT machinery, and we and others have previously implicated RNF26 in other ESCRT-dependent processes, namely the downregulation of EGFR (Cremer *et al*, 2021) and degradation of STING (Qin *et al*, 2014; Assil & Paludan, 2023; Kuchitsu *et al*, 2023), interplay between RNF26, vimentin, and ESCRT complexes could be interesting to pursue in future studies. For instance, K63-linked ubiquitin conjugates have been detected on RNF26 (Fenech *et al*, 2020), and this linkage type is known to be preferred by the ESCRT-0 members Hrs and STAM (Nathan *et al*, 2013). In this light, phosphorylation-induced removal of UBE2J1 (Elangovan *et al*, 2017) during stress recovery could be envisioned to modulate RNF26–ESCRT interactions.

Complementary to our findings on its role in perinuclear juxtaposition of biosynthetic and proteolytic organelles, vimentin was also shown to coordinate cytosolic protein turnover by bringing

© 2023 The Authors

proteasomes into the perinuclear cytoplasm (Morrow & Moore, 2020). Taking these observations together suggests that vimentin plays an active role in centralizing degradative capabilities for both cytoplasmic (proteasomes) and membrane-enclosed (endo-lysosomes) substrates in the vicinity of the ERQC, thereby ensuring that a variety of disposal routes are on hand in response to proteotoxic stress. Further along these lines, compartmentalization of misfolded proteins within the ER during ER stress may reflect vimentin-mediated "caging" previously alluded to in the context of cytoplasmic protein aggregates (Johnston *et al*, 1998). Exploiting vimentin scaffolding for spatiotemporal integration of membrane-bound and unbound organelles within the perinuclear space may thus offer a multitude of options for efficient disposal of unwanted ER proteins and membranes *en route* to homeostasis.

Materials and Methods

Antibodies and reagents

(*Confocal microscopy*) mouse anti-EEA1 (1:200, mAb 610457, BD transduction laboratories), mouse anti-CD63 NKI-C3 (1:500, Vennegoor & Rümke, 1986), rabbit anti-LAMP1 (11215-R017, 1:200, Sino Biological), rabbit anti-VAP-A (1:100, 15272-1-AP, Proteintech), goat anti-CNX (AB0041 SicGen, 1:100), mouse anti-CLIMP63 (16686-1-AP, ProteinTech, 1:100), rabbit anti-HRD1 (AP-2148A, Abgent, 1:100), mouse anti-HERP1 (19-Y, 1:40, SCT) followed by anti-Rabbit/Mouse/goat Alexa-dye coupled antibodies (1:400, Invitrogen). SiR-lysosome (1:2,000, Spirochrome) or LysoTracker Red (Life Technologies, 1:10,000) was used to visualize lysosomes in live microscopy.

(*Western blotting*) mouse anti-vimentin V9 (1:200, SC6260), anti-RFP (Rocha *et al*, 2009), mouse anti-Vinculin (1:5,000, Merck 9131) and rabbit anti-HA (1:1,000, CST#3724), rabbit anti-ATF4 (1:500 CST#11815), mouse anti-HERP1 (1:200 19-Y Santa Cruz Biotech), rabbit anti-PERK (1:1,000 Proteintech 20582-1-AP), rabbit anti-p-eiF2α (1:500 CST#3398T) followed by secondary Rabbit anti-mouse-HRP, sheep anti-rabbit-HRP (Invitrogen), or goat anti-rabbit or goat anti-mouse IRdye 680 (1:20,000) and IRdye 800 (1:10,000) antibodies (LiCor). Halo-PEG(2)-NH2*HCl was purchased from Iris Biotech (Marktredwitz, Germany).

5(6)-Carboxytetramethylrhodamine succinimidyl ester was purchased through VWR (Amsterdam, the Netherlands). Diisopropylethylamine (DiPEA) and dimethylformamide (DMF) were purchased from Biosolve (Valkenswaard, the Netherlands). RFP-TRAP beads were obtained from Chromotek (Planegg, Germany). Recombinant His-vimentin was obtained from Sino Biologicals (Beijing, China). Bafilomycin was from Tebu-Bio (Heerhugowaard, the Netherlands). Tunicamycin was obtained from Merck (Rahway, US) and CPA from Tocris bioscience (Bristol, UK).

Cell culture

HeLa cells (CCL-2) were cultured in DMEM medium (Gibco). U2OS and HEK293T cells were obtained from ATCC and cultured in DMEM medium (Gibco). MelJuSo cells were cultured in IMDM. All media were supplemented with 8% fetal calf serum (FCS, Sigma). All cell lines were cultured at 37°C and 5% CO2 and routinely tested (negatively) for mycoplasma. All cell lines were sequence verified.

siRNA transfections

Sequences of the siRNA oligos targeting RNF26 were obtained from Dharmacon (siGenome library) (siRNF26-1: CAGGAGGGAUAACCG-GAUUUU; siRNF26-2: GAGAGGAUGUCAUGCGGCU, siHERP1 pool of 4 siRNAs). Gene silencing was performed in a 48- or 24-well plate (IF) or 12-well plate (WB)—reagent volumes were scaled up accordingly. In a 24-well plate, 65 µl siRNA [50 nM] was mixed with 26 µl 1× siRNA buffer (GE Healthcare) containing 1.15 µl Dharmafect 1 transfection reagent. The mix was incubated on a shaker at RT for 30 min before the addition of 18,000 HeLa or 30,000 U2OS cells (and coverslips). Cells were cultured for 3 days before analysis. Nontargeting siRNA or reagent-only was used as a negative control.

Constructs

RNF26 (and mutants) were expressed from C1/N1 CMV promoter vector series (Clontech) and have been previously described (Jongsma *et al*, 2016). ER-targeted mCherry was a kind gift from M. Davidson (Addgene plasmid #54501). Vimentin was subcloned between EcoRI and BamHI sites of the C1-IRES-GFP vector of C1-GFP/RFP vector (Clontech). HERP1 and HERP2 were obtained from a pDONR library (LUMC) and subcloned in C1-2HA (Clontech) using standard PCR methods. HRD1 plasmid was kindly provided by E. Wiertz (UMC Utrecht) and subcloned into N1-2HA (Clontech) using standard PCR methods. pMRX-INU-SEC62-FLAG was a gift from Noboru Mizushima (Addgene plasmid # 128263). From this, Sec62-HALO-GFP was subcloned using standard PCR methods. For protein purification, RNF26 was subcloned into pRP265, and 3C protease sequence followed by 2xSTREP tag was inserted between GST and RNF26 sequence. Mutants were created by standard (mutagenesis) PCR methods and sequence verified.

Protein purification

GST-3C-Strep-RNF26 tail (aa 304–433) was transformed into *BL21 E. coli* and when bacterial growth reached OD600 (1.2), protein expression was induced overnight at 18°C with 100 µM IPTG in the presence of 15 mM $ZnCl_2$. The next day, bacteria were pelleted and lysed in PBS containing protease inhibitors (Roche, EDTA-free) by ice-cold sonication. Lysates were cleared using centrifugation and protein was purified on gravity-flow columns loaded with glutathione-coupled resin beads (GE Healthcare) and eluted in PBS supplemented with 20 mM GSH. Next, eluates were incubated with recombinant GST-3C protease to remove GST and RNF26 was reverse-purified using GSH-beads. Protein-containing fractions were pooled and concentrated using spin filters (Amicon) before being incubated overnight at 4°C with surplus NHS-Rhodamine to label free amines. Reaction was quenched with 100 mM Tris and protein was diluted to 1 mg/ml, aliquoted, and stored in −80°C until further use.

Solid phase peptide synthesis

SPPS was performed using a Syro II synthesizer (Multisyntech GmbH, Witten, Germany) using standard 9-fluorenylmethoxycarbonyl (Fmoc)

 © 2023 The Authors

based solid-phase peptide synthesis. Amino acids were double coupled in fourfold excess to preloaded Fmoc amino acid HMPA resin (0.2 mmol/g, Rapp Polymere GmbH) in N-methyl-2-pyrrolidone (NMP) for 45 min on a 2 μmol scale. The resin was swelled by the addition of 100 μl NMP for 5 min (×2). Fmoc was removed by treatment with 20% piperidine in NMP three times for 3, 5, and 5 min, followed by five washing steps using NMP. Amino acids were coupled by adding a fourfold excess in the presence of four equivalents benzotriazol-1-yloxytripyrrolidinophosphonium hexafluorophosphate (PyBOP) and eight equivalents N,N-diisopropylethylamine (DiPEA) in NMP for 45 min, followed by three washing steps with NMP. After completion of all coupling cycles, the resin was washed with Et_2O and dried under vacuum.

Peptide cleavage and lyophilization

Cleavage of the peptide from the resin was performed using a 3-h incubation in 400 μl 90% Trifluoric Acid, 5% MilliQ, 2.5% Phenol, and 2.5% Tri-isopropylsilane. Next, the solution was filtered, and the peptide was precipitated by adding cold 50% Ether/Pentane solution. Then, the peptide was washed three times in 1 ml of ether, dried on air, and dissolved in lyophilization mix (75% MilliQ, 24% Acetonitile, 1% Acetic acid). Of this solution, 2 μl was analyzed by LC–MS, and the remainder was frozen in liquid nitrogen and lyophilized on the Christ lyophilizer at −90°C and pressure of < 0.100 mBar.

Fluorescence polarization assay

Rhodamine-labeled RNF26 c-terminus peptide was reconstituted in DMSO to 1 mM and stored in −80°C. Before use, peptides were diluted to 100 nM in FP-buffer (10 mM PIPES + 0.1% CHAPS + 0.2% BSA). In a black low-adherence 384-well plate (X), 1 μl peptide was mixed with 9 μl his-Vim serial dilutions that were freshly prepared from reconstituted his-Vim stock. Reactions were incubated for 30 min at 37°C before fluorescence polarization measurement with a Clariostar (BMG LabTech) and compared with Rhodamine-only control. FP-baseline was set to 30 mP on Rho-only and Rho-RNF26 only controls.

In vitro interaction studies

Three microgram his-Vim and 3 μg Rho-RNF26tail were incubated in PBS for 30 min at 37°C. Then, his-Vim was precipitated on Ni-NTA beads and subsequently washed three times with PBS before addition of 1× SB and analysis by SDS–PAGE and detection of in-gel Rhodamine fluorescence using a Typhoon scanner and WB detection of vimentin.

DNA transfections

Cells were seeded in culture plates to reach approximately 70% confluency on the day of transfection. For IF, cells were transfected with Effectene (Qiagen) (200 ng DNA per well of a 24-well plate), according to the manufacturer's protocol. Larger dishes of HeLa cells were transfected with PEI at a ratio of 3 μg PEI per μg DNA in 200 μl DMEM medium. After 15–30 min, the mix was added dropwise to the cells, which were then cultured overnight prior to analysis.

CRISPR/Cas9-mediated knockout

gRNA sequences targeting the vimentin (TCCTACCGCAG-GATGTTCGG) or RNF26 gene (GTACCTGGTAGTGAATGGGT) were cloned into the BbsI site of PX440 (containing the Cas9 gene and a puromycin resistance gene). This plasmid was transfected into HeLa or U2OS cells and the next day, cells were selected with 200 μg/ml puromycin for 3 days. Then, cells were diluted and cultured in a 15 cm dish, allowing separated colonies to grow. These were isolated, expanded, and analyzed for loss of vimentin or RNF26 by western blot.

qPCR

RNA was isolated from 24-well plate cultures using RNA mini kit according to the manufacturer's protocol, reverse transcribed into cDNA using Roche kit, and diluted one-fourth before analysis. qPCR was performed using 1.5 μl DNA, 2× SYBR master mix, 333 nM primers, and 5% DMSO in a 384 well format. PCRs were performed using a BioRAD Opus system and analyzed in the Maestro software. Relative transcript abundance was determined using ΔcT method in MS Excel and normalized to GAPDH housekeeping gene.

qPCR primer list:
GAPDH: Fw TACTAGCGGTTTTACGGGCG; Rv TCGAACAGGAGGA GCAGAGAGCGA
BiP: Fw TAGCGTATGGTGCTGCTGTC; Rv TTTGTCAGGGGTCTTT CACC
CHOP: Fw AGAACCAGCAGAGGTCACA; Rv GCTGTGCCACTTT CCTTTC
HERP: Fw CGTTGTTATGTACCTGCATC; Rv TCAGGAGGAGGACCA TCATTT
RNF26: Fw TCGGCACTCAGAACCTCTTT; Rv CTAGGAAGGCAGCC ACTACG
XBP1: Fw CCTTGTAGTTGAGAACCAGG; Rv GGGGCTTGGTATATA TGTGG
XBP1-spliced: Fw TGCTGAGTCCGCAGCAGGTG; Rv GCTGGCAGG CTCTGGGGAAG.

Co-immunoprecipitations

U2OS cells were lysed in 1% DMNG buffer (150 mM NaCl, 50 mM Tris–HCl pH 7.5, 5 mM MgCl2, 1% DMNG, protease inhibitors) (Roche diagnostics, EDTA free) for 90 min, rotating at 4°C. After 15 min 20,000 *g* centrifugation, postnuclear lysates were incubated with RFP-TRAP beads (Chromotek) and rotated for 90 min at 4°C. Beads were washed four times in 0.2% DMNG lysis buffer. Samples were boiled for 10 min in 2× Laemmli buffer prior to analyses by SDS–PAGE and western blotting.

TAMRA-HALOtag ligand synthesis

The reaction was performed under an inert nitrogen atmosphere. 5 (6)-Carboxytetramethylrhodamine succinimidyl ester (10 mg; 0.019 mmol) was dissolved in DMF.DiPEA (9.9 μl; 0.057 mmol) was added followed by a solution of Halo-PEG(2)-NH2*HCl (5.4 mg; 0.021 mmol) in DMF (0.25 ml). The mixture was stirred overnight at ambient temperature before the crude mixture was purified by HPLC.

© 2023 The Authors

ER-phagy assay

Monitoring delivery of Sec62 into proteolytic compartments was performed according to a method developed as described previously (Rudinskiy *et al*, 2022). U2OS cells were transfected overnight with Sec62-HALO-GFP and the next day, cells were incubated for 15 min at 37°C with 25 μM *black* HALO-tag ligand 6-Chlorohexanol. Next, cells were washed 2× with PBS and pulsed at 37°C with 200 nM JF646- (for microscopy) or 100 nM TAMRA- (for gel analysis) HALOtag ligands. After 1 h, cells were washed 1× with PBS and medium was replaced for the indicated times before washing and fixation (for microscopy) or cell lysis in 1× LSB + DTT (for SDS–PAGE analysis). For Sec62-colocalization studies, transfected cells were simply incubated with JF646 ligand for 5 h before fixation.

Immunofluorescence confocal microscopy

Cells grown on coverslips (Menzel Gläser) were fixed with 3.7% paraformaldehyde, washed three times with PBS, permeabilized with 0.1% TX100 for 10 min, and blocked in 0.5% BSA for 1 h. Next, coverslips were incubated with primary antibodies in 0.5% BSA for 1 h at RT, washed, and incubated with Alexa-labeled anti mouse/rabbit/rat secondary fluorescent antibody or streptavidin. After washing, coverslips were mounted on glass slides with ProLong Gold with DAPI (Life Technologies). Samples were imaged with a Leica SP8 confocal microscope equipped with appropriate solid-state lasers, HCX PL 63× oil immersion objectives and HyD detectors (Leica Microsystems, Wetzlar, Germany). Data were collected using 2,048 × 2,048 scanning format with line averaging without digital zoom, or 1,024 × 1,024 scanning format with digital zoom in the range of 1.0–2.0 with line averaging. Images were smoothed with 1× pixel average filter in ImageJ.

Overlap analysis was performed using JaCoP analysis tool in Fiji with manual threshold selection. Where indicated, super-resolution images were acquired with a Zeiss Airyscan microscope with 63× objective, 2× pixel sampling, and deconvolved using the Zen software.

Organelle distribution analysis

To analyze intracellular pixel distribution, we developed a ImageJ plugin that segments the cytoplasm into areas of varying distance to the nucleus. In short, the plugin uses thresholding to segment the nuclei, followed by optional manual correction. The outer edges of the cell are manually annotated. Then, using a Euclidian distance map, we measure for each pixel within the cytoplasm (outside nucleus and within the cell) the relative (normalized to the maximum distance per cell) distance to the nucleus. These distance values are then segmented into a selectable number of bins (in our Case 4), resulting in the segmentation shown in Fig 1D. Per bin, total signal was divided by pixel number, resulting in fluorescence intensity (MFI). ImageJ plugin results were imported into MS Excel where MFI value was compared across bins 1 and 3.

ER sheet analysis

Endoplasmic reticulum sheets were visualized using transient transfection of mCherry-KDEL and subsequent live cell microscopy. Using Fiji, images were smoothened, and total ER was masked using manual thresholding. In parallel, ER sheets were masked by making a binary image which was then eroded twice to remove ER tubules. The total area of the obtained sheet-mask was measured using particle analysis and normalized to total ER area.

Live microscopy

For live cell microscopy, cells were seeded in four-chamber live cell dishes and imaged under conditions of 37°C and 5% CO_2 with a Leica SP8 confocal microscope equipped with solid state lasers or an Andor Dragonfly spinning disk module. Cells were incubated with SR101 (5 μM) as indicated. Data were collected using 63× or 100× oil immersion objectives in a 2,048 × 2,048 scanning format at three frames/s. Time color coded images were made with Fiji.

ER membrane dynamics analysis

Endoplasmic reticulum membrane displacement was quantified using a custom developed Fiji plugin that has been described earlier (Spits *et al*, 2021). The method is based on optical flow analysis, which defines corresponding areas between frames and generates a vector map. Live cell movies were simplified by removing every second and third frame, leaving a one frame/s video for analysis. Membrane displacement (60 nm/s) was binned into seven displacement bins, ranging from 0 to 60 nm/s (resulting in displacement bins of nm/s). The first two bins (i.e., 0 and < 60 nm/s displacement) were classified as "static ER." ROIs of 5 × 5 μm from at least 10 cells were compared across conditions.

Proximity biotinylation pulldowns

HeLa cells transfected to express proteins of interest and were treated as indicated in the figure legends before three washes with PBS and subsequent cell lysis in 0.5% TX-100 lysis buffer (150 mM NaCl, 50 mM Tris–HCl pH 7.5, 5 mM $MgCl_{22}$, 0.5% (v/v) TX-100 and protease inhibitors (Roche diagnostics, EDTA free)). Nuclei were lysed by adding 1:4 SDS buffer (2% SDS, EDTA) and samples were sonicated (6x1s pulses, 70% power, Fisher Scientific). Samples were diluted to 0.2% SDS with TX-100 lysis buffer and centrifuged for 20 min at 20,000 *g*. After spinning, samples were incubated with Neutravidin-agarose beads (Pierce) overnight at 4°C. Beads were washed four times with 1% SDS in PBS before elution in a 2× Laemmle sample buffer by boiling for 10 min and SDS–PAGE.

SDS–PAGE and western blotting

Samples were separated by 10% SDS–PAGE and transferred to nitrocellulose in ethanol-containing transfer buffer at 300 mA for 2–3 h. The membranes were blocked with 5% milk/PBS before incubation with primary antibody diluted in blocking buffer for 1 h at RT. After washing twice with PBS/0.1% Tween-20, proteins were detected with secondary antibodies. Fluorescent signals were directly imaged with an Odyssey Fx laser scanning fluorescence imager (Li-COR Biosciences) and quantified using the LiCor ImageStudio software.

Mass-spectrometry

Mass-spectrometry was performed as described previously (Jongsma *et al*, 2016).

 © 2023 The Authors

Electron microscopy and tomography

3D tomography and rendering

Adherent U2OS cells cultured in a 5-cm-diameter Petri dish were fixed for 2 h at room temperature in 0.1 M Cacodylate buffer containing 1.5% glutaraldehyde. The fixed cells were incubated for 1 h on ice in a Cacodylate buffer containing 1% Osmium tetroxide and subsequently in water containing 1% uranyl acetate. The cells were then dehydrated through a series of ethanol steps and embedded in Epon. The flat embedded cells were sectioned with an ultramicrotome using a diamond knife at a nominal section thickness of 200 nm. The sections were transferred to a formvar and carbon coated 0.5 × 2 mm copper slot grid and stained for 20 min with 7% uranyl acetate in water and for 10 min with lead citrate according to Reynolds (1963). Electron microscopy images of serial sections were recorded by a Tecnai 20 TEM (Thermo Fisher Scientific) equipped with an EAGLE 4 × 4K digital camera. The tilt series for electron tomography were collected using the Xplore3D (Thermo Fisher Scientific) software. The angular tilt range was set from −60° to 60° with 2° increments. Alignments of the tilt series and weighted-back projection reconstructions for tomography were performed using IMOD (Kremer *et al*, 1996). The drawing and annotation of subcellular structures in the tomograms and the alignment of the serial tomograms was done manually using AMIRA (Thermo Fisher Scientific). Generated surfaces were exported to Cinema 4D (Maxon), which was used to smooth the surfaces, and to render images and movies (Redshift, Maxon).

Statistics

All error bars in WB quantification correspond to SD or the mean from three or more independent experiments. For microscopy, data of cells from at least three independent experiments were used. In distance bin analysis and membrane displacement analysis, error bars indicate median and 95% confidence interval. Statistical evaluations report on Student's *t*-test (for gel and qPCR (paired) and colocalization (unpaired) analysis) with error bars corresponding to mean and SEM (gel and qPCR) or standard deviation (colocalization), or Mann–Whitney U nonparametrical test (for non-normal distributions of perinuclearity and membrane displacement analysis), with error bars corresponding to median and 95% confidence interval, as described in the legends, $*P < 0.05$, $**P < 0.01$, $***P < 0.001$, $****P < 0.0001$; ns: not significant.

Data availability

The mass spectrometry proteomics data have been deposited to the ProteomeXchange Consortium via the PRIDE (Perez-Riverol *et al*, 2022) partner repository with the dataset identifier PXD040760 (http://www.ebi.ac.uk/pride/archive/projects/PXD040760).

Expanded View for this article is available online.

Acknowledgements

We thank L.J. Janssen (LUMC) for DNA constructs and B. Cabukusta (LUMC) for help with homology analysis and Fiji software. This work was supported by an ERC Adv grant (ERCOPE 694307) to J. Neefjes. This work is dedicated to M.O. Cremer.

Author contributions

Tom Cremer: Conceptualization; data curation; formal analysis; supervision; validation; investigation; visualization; methodology; writing – original draft; project administration. **Lenard M Voortman:** Software; methodology. **Erik Bos:** Data curation; formal analysis; investigation. **Marlieke LM Jongsma:** Data curation; formal analysis; investigation; writing – review and editing. **Laurens R ter Haar:** Investigation. **Jimmy JLL Akkermans:** Resources. **Cami MP Talavera Ormeño:** Resources. **Ruud HM Wijdeven:** Investigation. **Jelle de Vries:** Resources. **Robbert Q Kim:** Supervision; methodology. **George MC Janssen:** Investigation. **Peter A van Veelen:** Data curation; supervision; investigation. **Roman I Koning:** Formal analysis; supervision; investigation. **Jacques Neefjes:** Conceptualization; supervision; funding acquisition; investigation; project administration; writing – review and editing. **Ilana Berlin:** Conceptualization; supervision; visualization; writing – original draft; project administration; writing – review and editing.

Disclosure and competing interests statement

The authors declare that they have no conflict of interest.

References

Ajoolabady A, Kaplowitz N, Lebeaupin C, Kroemer G, Kaufman RJ, Malhi H, Ren J (2023) Endoplasmic reticulum stress in liver diseases. *Hepatology* 77: 619 – 639

Ang SF, Fölsch H (2012) The role of secretory and endocytic pathways in the maintenance of cell polarity. *Essays Biochem* 53: 29 – 39

Assil S, Paludan SR (2023) STING is ESCRTed to degradation by microautophagy. *Nat Cell Biol* 25: 379 – 380

Bae D, Moore KA, Mella JM, Hayashi SY, Hollien J (2019) Degradation of Blos1 mRNA by IRE1 repositions lysosomes and protects cells from stress. *J Cell Biol* 218: 1118 – 1127

Branon TC, Bosch JA, Sanchez AD, Udeshi ND, Svinkina T, Carr SA, Feldman JL, Perrimon N, Ting AY (2018) Efficient proximity labeling in living cells and organisms with TurboID. *Nat Biotechnol* 36: 880 – 887

Bravo R, Vicencio JM, Parra V, Troncoso R, Munoz JP, Bui M, Quiroga C, Rodriguez AE, Verdejo HE, Ferreira J *et al* (2011) Increased ER-mitochondrial coupling promotes mitochondrial respiration and bioenergetics during early phases of ER stress. *J Cell Sci* 124: 2143 – 2152

Chang L, Barlan K, Chou YH, Grin B, Lakonishok M, Serpinskaya AS, Shumaker DK, Herrmann H, Gelfand VI, Goldman RD (2009) The dynamic properties of intermediate filaments during organelle transport. *J Cell Sci* 122: 2914 – 2923

Christianson JC, Carvalho P (2022) Order through destruction: how ER-associated protein degradation contributes to organelle homeostasis. *EMBO J* 41: e109845

Cohen S, Valm AM, Lippincott-Schwartz J (2018) Interacting organelles. *Curr Opin Cell Biol* 53: 84 – 91

Cremer T, Neefjes J, Berlin I (2020) The journey of Ca^{2+} through the cell - pulsing through the network of ER membrane contact sites. *J Cell Sci* 133: jcs249136

Cremer T, Jongsma MLM, Trulsson F, Vertegaal ACO, Neefjes J, Berlin I (2021) The ER-embedded UBE2J1/RNF26 ubiquitylation complex exerts spatiotemporal control over the endolysosomal pathway. *Cell Rep* 34: 108659

Duarte S, Viedma-Poyatos A, Navarro-Carrasco E, Martinez AE, Pajares MA, Perez-Sala D (2019) Vimentin filaments interact with the actin cortex in mitosis allowing normal cell division. *Nat Commun* 10: 4200

Eisenberg-Bord M, Shai N, Schuldiner M, Bohnert M (2016) A tether is a tether is a tether: tethering at membrane contact sites. *Dev Cell* 39: 395 – 409

© 2023 The Authors

Elangovan M, Chong HK, Park JH, Yeo EJ, Yoo YJ (2017) The role of ubiquitin-conjugating enzyme Ube2j1 phosphorylation and its degradation by proteasome during endoplasmic stress recovery. *J Cell Commun Signal* 11: 265 – 273

Etienne-Manneville S (2018) Cytoplasmic intermediate filaments in cell biology. *Annu Rev Cell Dev Biol* 34: 1 – 28

Farias GG, Freal A, Tortosa E, Stucchi R, Pan X, Portegies S, Will L, Altelaar M, Hoogenraad CC (2019) Feedback-driven mechanisms between microtubules and the endoplasmic reticulum instruct neuronal polarity. *Neuron* 102: 184 – 201

Fenech EJ, Lari F, Charles PD, Fischer R, Laetitia-Thezenas M, Bagola K, Paton AW, Paton JC, Gyrd-Hansen M, Kessler BM *et al* (2020) Interaction mapping of endoplasmic reticulum ubiquitin ligases identifies modulators of innate immune signalling. *Elife* 9: e57306

Franke WW, Hergt M, Grund C (1987) Rearrangement of the vimentin cytoskeleton during adipose conversion: formation of an intermediate filament cage around lipid globules. *Cell* 49: 131 – 141

Fumagalli F, Noack J, Bergmann TJ, Cebollero E, Pisoni GB, Fasana E, Fregno I, Galli C, Loi M, Solda T *et al* (2016) Translocon component Sec62 acts in endoplasmic reticulum turnover during stress recovery. *Nat Cell Biol* 18: 1173 – 1184

Gan Z, Ding L, Burckhardt CJ, Lowery J, Zaritsky A, Sitterley K, Mota A, Costigliola N, Starker CG, Voytas DF *et al* (2016) Vimentin intermediate filaments template microtubule networks to enhance persistence in cell polarity and directed migration. *Cell Syst* 3: 252 – 263

Gao YS, Vrielink A, MacKenzie R, Sztul E (2002) A novel type of regulation of the vimentin intermediate filament cytoskeleton by a Golgi protein. *Eur J Cell Biol* 81: 391 – 401

Goyal U, Blackstone C (2013) Untangling the web: mechanisms underlying ER network formation. *Biochim Biophys Acta* 1833: 2492 – 2498

Herrmann H, Aebi U (2016) Intermediate filaments: structure and assembly. *Cold Spring Harb Perspect Biol* 8: a018242

Hetz C, Saxena S (2017) ER stress and the unfolded protein response in neurodegeneration. *Nat Rev Neurol* 13: 477 – 491

Hirokawa N, Noda Y, Tanaka Y, Niwa S (2009) Kinesin superfamily motor proteins and intracellular transport. *Nat Rev Mol Cell Biol* 10: 682 – 696

Hookway C, Ding L, Davidson MW, Rappoport JZ, Danuser G, Gelfand VI (2015) Microtubule-dependent transport and dynamics of vimentin intermediate filaments. *Mol Biol Cell* 26: 1675 – 1686

Huang CH, Chu YR, Ye Y, Chen X (2014) Role of HERP and a HERP-related protein in HRD1-dependent protein degradation at the endoplasmic reticulum. *J Biol Chem* 289: 4444 – 4454

Ivaska J (2011) Vimentin: central hub in EMT induction? *Small GTPases* 2: 51 – 53

Jain A, Holthuis JCM (2017) Membrane contact sites, ancient and central hubs of cellular lipid logistics. *Biochim Biophys Acta Mol Cell Res* 1864: 1450 – 1458

Jia R, Bonifacino JS (2019) Lysosome positioning influences mTORC2 and AKT signaling. *Mol Cell* 75: 26 – 38

Jiu Y, Lehtimaki J, Tojkander S, Cheng F, Jaalinoja H, Liu X, Varjosalo M, Eriksson JE, Lappalainen P (2015) Bidirectional interplay between vimentin intermediate filaments and contractile actin stress fibers. *Cell Rep* 11: 1511 – 1518

Johnson DE, Ostrowski P, Jaumouillé V, Grinstein S (2016) The position of lysosomes within the cell determines their luminal pH. *J Cell Biol* 212: 677 – 692

Johnston JA, Ward CL, Kopito RR (1998) Aggresomes: a cellular response to misfolded proteins. *J Cell Biol* 143: 1883 – 1898

Jongsma ML, Berlin I, Wijdeven RH, Janssen L, Janssen GM, Garstka MA, Janssen H, Mensink M, van Veelen PA, Spaapen RM *et al* (2016) An ER-associated pathway defines endosomal architecture for controlled cargo transport. *Cell* 166: 152 – 166

Jongsma ML, Bakker J, Cabukusta B, Liv N, van Elsland D, Fermie J, Akkermans JL, Kuijl C, van der Zanden SY, Janssen L *et al* (2020) SKIP-HOPS recruits TBC1D15 for a Rab7-to-Arl8b identity switch to control late endosome transport. *EMBO J* 39: e102301

Jumper J, Evans R, Pritzel A, Green T, Figurnov M, Ronneberger O, Tunyasuvunakool K, Bates R, Žídek A, Potapenko A *et al* (2021) Highly accurate protein structure prediction with AlphaFold. *Nature* 596: 583 – 589

Ketema M, Kreft M, Secades P, Janssen H, Sonnenberg A (2013) Nesprin-3 connects plectin and vimentin to the nuclear envelope of Sertoli cells but is not required for Sertoli cell function in spermatogenesis. *Mol Biol Cell* 24: 2454 – 2466

Klopfenstein DRC, Kappeler F, Hauri H-P (1998) A novel direct interaction of endoplasmic reticulum with microtubules. *EMBO J* 17: 6168 – 6177

Kny M, Standera S, Hartmann-Petersen R, Kloetzel PM, Seeger M (2011) Herp regulates Hrd1-mediated ubiquitylation in a ubiquitin-like domain-dependent manner. *J Biol Chem* 286: 5151 – 5156

Kondratyev M, Avezov E, Shenkman M, Groisman B, Lederkremer GZ (2007) PERK-dependent compartmentalization of ERAD and unfolded protein response machineries during ER stress. *Exp Cell Res* 313: 3395 – 3407

Konietzny A, Grendel J, Kadek A, Bucher M, Han Y, Hertrich N, Dekkers DHW, Demmers JAA, Grünewald K, Uetrecht C *et al* (2022) Caldendrin and myosin V regulate synaptic spine apparatus localization via ER stabilization in dendritic spines. *EMBO J* 41: e106523

Korolchuk VI, Saiki S, Lichtenberg M, Siddiqi FH, Roberts EA, Imarisio S, Jahreiss L, Sarkar S, Futter M, Menzies FM *et al* (2011) Lysosomal positioning coordinates cellular nutrient responses. *Nat Cell Biol* 13: 453 – 460

Kremer JR, Mastronarde DN, McIntosh JR (1996) Computer visualization of three-dimensional image data using IMOD. *J Struct Biol* 116: 71 – 76

Kuchitsu Y, Mukai K, Uematsu R, Takaada Y, Shinojima A, Shindo R, Shoji T, Hamano S, Ogawa E, Sato R *et al* (2023) STING signalling is terminated through ESCRT-dependent microautophagy of vesicles originating from recycling endosomes. *Nat Cell Biol* 25: 453 – 466

Leitman J, Ron E, Ogen-Shtern N, Lederkremer GZ (2013) Compartmentalization of endoplasmic reticulum quality control and ER-associated degradation factors. *DNA Cell Biol* 32: 2 – 7

Leitman J, Shenkman M, Gofman Y, Shtern NO, Ben-Tal N, Hendershot LM, Lederkremer GZ (2014) Herp coordinates compartmentalization and recruitment of HRD1 and misfolded proteins for ERAD. *Mol Biol Cell* 25: 1050 – 1060

Loi M, Raimondi A, Morone D, Molinari M (2019) ESCRT-III-driven piecemeal micro-ER-phagy remodels the ER during recovery from ER stress. *Nat Commun* 10: 5058

Lowery J, Kuczmarski ER, Herrmann H, Goldman RD (2015) Intermediate filaments play a pivotal role in regulating cell architecture and function. *J Biol Chem* 290: 17145 – 17153

Lu M, van Tartwijk FW, Lin JQ, Nijenhuis W, Parutto P, Fantham M, Christensen CN, Avezov E, Holt CE, Tunnacliffe A *et al* (2020) The structure and global distribution of the endoplasmic reticulum network are actively regulated by lysosomes. *Sci Adv* 6: eabc7209

Miyazaki K, Wakana Y, Noda C, Arasaki K, Furuno A, Tagaya M (2012) Contribution of the long form of syntaxin 5 to the organization of the endoplasmic reticulum. *J Cell Sci* 125: 5658 – 5666

Morrow CS, Moore DL (2020) Vimentin's side gig: regulating cellular proteostasis in mammalian systems. *Cytoskeleton (Hoboken)* 77: 515 – 523

Nathan JA, Kim HT, Ting L, Gygi SP, Goldberg AL (2013) Why do cellular proteins linked to K63-polyubiquitin chains not associate with proteasomes? *Embo J* 32: 552 – 565

 © 2023 The Authors

Neefjes J, Jongsma MM, Berlin I (2017) Stop or go? Endosome positioning in the establishment of compartment architecture, dynamics, and function. *Trends Cell Biol* 27: 580–594

Nekrasova OE, Mendez MG, Chernoivanenko IS, Tyurin-Kuzmin PA, Kuczmarski ER, Gelfand VI, Goldman RD, Minin AA (2011) vimentin intermediate filaments modulate the motility of mitochondria. *Mol Biol Cell* 22: 2282–2289

Noda C, Kimura H, Arasaki K, Matsushita M, Yamamoto A, Wakana Y, Inoue H, Tagaya M (2014) Valosin-containing protein-interacting membrane protein (VIMP) links the endoplasmic reticulum with microtubules in concert with cytoskeleton-linking membrane protein (CLIMP)-63. *J Biol Chem* 289: 24304–24313

Ogawa-Goto K, Tanaka K, Ueno T, Tanaka K, Kurata T, Sata T, Irie S (2007) p180 is involved in the interaction between the endoplasmic reticulum and microtubules through a novel microtubule-binding and bundling domain. *Mol Biol Cell* 18: 3741–3751

Oikonomou C, Hendershot LM (2020) Disposing of misfolded ER proteins: a troubled substrate's way out of the ER. *Mol Cell Endocrinol* 500: 110630

Patteson AE, Vahabikashi A, Pogoda K, Adam SA, Mandal K, Kittisopikul M, Sivagurunathan S, Goldman A, Goldman RD, Janmey PA (2019) Vimentin protects cells against nuclear rupture and DNA damage during migration. *J Cell Biol* 218: 4079–4092

Perez-Riverol Y, Bai J, Bandla C, García-Seisdedos D, Hewapathirana S, Kamatchinathan S, Kundu DJ, Prakash A, Frericks-Zipper A, Eisenacher M *et al* (2022) The PRIDE database resources in 2022: a hub for mass spectrometry-based proteomics evidences. *Nucleic Acids Res* 50: D543–D552

Prinz WA, Toulmay A, Balla T (2020) The functional universe of membrane contact sites. *Nat Rev Mol Cell Biol* 21: 7–24

Qin Y, Zhou MT, Hu MM, Hu YH, Zhang J, Guo L, Zhong B, Shu HB (2014) RNF26 temporally regulates virus-triggered type I interferon induction by two distinct mechanisms. *PLoS Pathog* 10: e1004358

Reck-Peterson SL, Redwine WB, Vale RD, Carter AP (2018) The cytoplasmic dynein transport machinery and its many cargoes. *Nat Rev Mol Cell Biol* 19: 382–398

Reggiori F, Molinari M (2022) ER-phagy: mechanisms, regulation, and diseases connected to the lysosomal clearance of the endoplasmic reticulum. *Physiol Rev* 102: 1393–1448

Ren J, Bi Y, Sowers JR, Hetz C, Zhang Y (2021) Endoplasmic reticulum stress and unfolded protein response in cardiovascular diseases. *Nat Rev Cardiol* 18: 499–521

Reynolds ES (1963) The use of lead citrate at high pH as an electron-opaque stain in electron microscopy. *J Cell Biol* 17: 208–212

Risco C, Rodríguez JR, López-Iglesias C, Carrascosa JL, Esteban M, Rodríguez D (2002) Endoplasmic reticulum-Golgi intermediate compartment membranes and vimentin filaments participate in vaccinia virus assembly. *J Virol* 76: 1839–1855

Rocha N, Kuijl C, van der Kant R, Janssen L, Houben D, Janssen H, Zwart W, Neefjes J (2009) Cholesterol sensor ORP1L contacts the ER protein VAP to control Rab7-RILP-p150 glued and late endosome positioning. *J Cell Biol* 185: 1209–1225

Rodriguez-Garcia R, Volkov VA, Chen CY, Katrukha EA, Olieric N, Aher A, Grigoriev I, Lopez MP, Steinmetz MO, Kapitein LC *et al* (2020) Mechanisms of motor-independent membrane remodeling driven by dynamic microtubules. *Curr Biol* 30: 972–987

Rudinskiy M, Bergmann TJ, Molinari M (2022) Quantitative and time-resolved monitoring of organelle and protein delivery to the lysosome with a tandem fluorescent Halo-GFP reporter. *Mol Biol Cell* 33: ar57

Sandoz PA, Denhardt-Eriksson RA, Abrami L, Abriata LA, Spreemann G, Maclachlan C, Ho S, Kunz B, Hess K, Knott G *et al* (2023) Dynamics of CLIMP-63 S-acylation control ER morphology. *Nat Commun* 14: 264

Schaedel L, Lorenz C, Schepers AV, Klumpp S, Koster S (2021) vimentin intermediate filaments stabilize dynamic microtubules by direct interactions. *Nat Commun* 12: 3799

Schulz J, Avci D, Queisser MA, Gutschmidt A, Dreher LS, Fenech EJ, Volkmar N, Hayashi Y, Hoppe T, Christianson JC (2017) Conserved cytoplasmic domains promote Hrd1 ubiquitin ligase complex formation for ER-associated degradation (ERAD). *J Cell Sci* 130: 3322–3335

Schwarz N, Leube RE (2016) Intermediate filaments as organizers of cellular space: how they affect mitochondrial structure and function. *Cell* 5: 30

Serres MP, Samwer M, Truong Quang BA, Lavoie G, Perera U, Gorlich D, Charras G, Petronczki M, Roux PP, Paluch EK (2020) F-Actin interactome reveals vimentin as a key regulator of actin organization and cell mechanics in mitosis. *Dev Cell* 52: 210–222

Shen B, Zheng P, Qian N, Chen Q, Zhou X, Hu J, Chen J, Teng J (2019) Calumenin-1 interacts with Climp63 to cooperatively determine the luminal width and distribution of endoplasmic reticulum sheets. *iScience* 22: 70–80

Shibata Y, Shemesh T, Prinz WA, Palazzo AF, Kozlov MM, Rapoport TA (2010) Mechanisms determining the morphology of the peripheral ER. *Cell* 143: 774–788

Sihag RK, Inagaki M, Yamaguchi T, Shea TB, Pant HC (2007) Role of phosphorylation on the structural dynamics and function of types III and IV intermediate filaments. *Exp Cell Res* 313: 2098–2109

Spits M, Heesterbeek IT, Voortman LM, Akkermans JJ, Wijdeven RH, Cabukusta B, Neefjes J (2021) Mobile late endosomes modulate peripheral endoplasmic reticulum network architecture. *EMBO Rep* 22: e50815

Starling GP, Yip YY, Sanger A, Morton PE, Eden ER, Dodding MP (2016) Folliculin directs the formation of a Rab34-RILP complex to control the nutrient-dependent dynamic distribution of lysosomes. *EMBO Rep* 17: 823–841

Svitkina T (2018) The actin cytoskeleton and actin-based motility. *Cold Spring Harb Perspect Biol* 10: a018267

Tang HL, Lung HL, Wu KC, Le AH, Tang HM, Fung MC (2008) vimentin supports mitochondrial morphology and organization. *Biochem J* 410: 141–146

Tikhomirova MS, Kadosh A, Saukko-Paavola AJ, Shemesh T, Klemm RW (2022) A role for endoplasmic reticulum dynamics in the cellular distribution of microtubules. *Proc Natl Acad Sci USA* 119: e2104309119

Toivola DM, Tao GZ, Habtezion A, Liao J, Omary MB (2005) Cellular integrity plus: organelle-related and protein-targeting functions of intermediate filaments. *Trends Cell Biol* 15: 608–617

Usman S, Waseem NH, Nguyen TKN, Mohsin S, Jamal A, Teh M-T, Waseem A (2021) Vimentin is at the heart of epithelial mesenchymal transition (EMT) mediated metastasis. *Cancers* 13: 4985

Vedrenne C, Klopfenstein DR, Hauri HP (2005) Phosphorylation controls CLIMP-63-mediated anchoring of the endoplasmic reticulum to microtubules. *Mol Biol Cell* 16: 1928–1937

Vennegoor C, Rümke P (1986) Circulating melanoma-associated antigen detected by monoclonal antibody NKI/C-3. *Cancer Immunol Immunother* 23: 93–100

Viedma-Poyatos Á, Pajares MA, Pérez-Sala D (2020) Type III intermediate filaments as targets and effectors of electrophiles and oxidants. *Redox Biol* 36: 101582

Waterman-Storer CM, Salmon ED (1998) Endoplasmic reticulum membrane tubules are distributed by microtubules in living cells using three distinct mechanisms. *Curr Biol* 8: 798–806

© 2023 The Authors

Westrate LM, Lee JE, Prinz WA, Voeltz GK (2015) Form follows function: the importance of endoplasmic reticulum shape. *Annu Rev Biochem* 84: 791 – 811

Wu H, Carvalho P, Voeltz GK (2018) Here, there, and everywhere: the importance of ER membrane contact sites. *Science* 361: eaan5835

Yamamoto K, Yoshida H, Kokame K, Kaufman RJ, Mori K (2004) Differential contributions of ATF6 and XBP1 to the activation of endoplasmic reticulum stress-responsive cis-acting elements ERSE, UPRE and ERSE-II. *J Biochem* 136: 343 – 350

Zheng P, Obara CJ, Szczesna E, Nixon-Abell J, Mahalingan KK, Roll-Mecak A, Lippincott-Schwartz J, Blackstone C (2022) ER proteins decipher the tubulin code to regulate organelle distribution. *Nature* 601: 132 – 138

License: This is an open access article under the terms of the Creative Commons Attribution License, which permits use, distribution and reproduction in any medium, provided the original work is properly cited.

Expanded View Figures

Figure EV1. (Related to Fig 1): RNF26 controls perinuclear ER organization.

A, B Validation of CRISPR-Cas9-generated RNF26 knockout U2OS cells. (A) DNA sequence analysis of RNF26 5′ CDS region, showing a CRISPR-induced 42 base pair deletion that spans the ATG start codon sequence. (B) WB analysis of RNF26 KO lysates using a validated RNF26 antibody. Vinculin was used as a loading control. MW markers as indicated.

C, D Effect of RNF26 knockout on the intracellular distribution of mCherry-KDEL. (C) Representative confocal z-projections of U2OS parental and RNF26 KO cells ectopically expressing mCherry-KDEL (*white*). Cell and nuclear boundaries are demarcated using dashed and continuous lines, respectively. (D) Quantification of Cherry-KDEL signal distribution expressed as perinuclearity ratio. Plotted are $n_{\mathrm{Parental}} = 89$, $n_{\mathrm{RNF26\ KO}} = 49$ technical replicates from three independent experiments.

E–H Effect of RNF26 depletion on the intracellular distribution of ER markers. U2OS cells transfected with the indicated siRNAs were fixed and immunostained against endogenous (E) VAP-A or (G) CLIMP63. Representative confocal fluorescence images are shown. Cell and nuclear boundaries are demarcated using dashed and continuous lines, respectively. Quantification of signal distribution as perinuclearity ratio for (F) VAP-A ($n_{\mathrm{siC}} = 59$, $n_{\mathrm{siRNF26\#1}} = 48$, $n_{\mathrm{siRNF26\#2}} = 103$ technical replicates) and (H) CLIMP63 ($n_{\mathrm{control}} = 75$, $n_{\mathrm{siRNF26\#2}} = 74$ cells, $n_{\mathrm{R26\ KO}} = 49$ technical replicates) from three independent experiments.

Data information: Graphs report median (red line) and 95% confidence interval (error bars) of sample values (open circles). Scale bar = 10 µm. Significance was assessed using a Mann–Whitney U; $^{***}P < 0.001$.

© 2023 The Authors

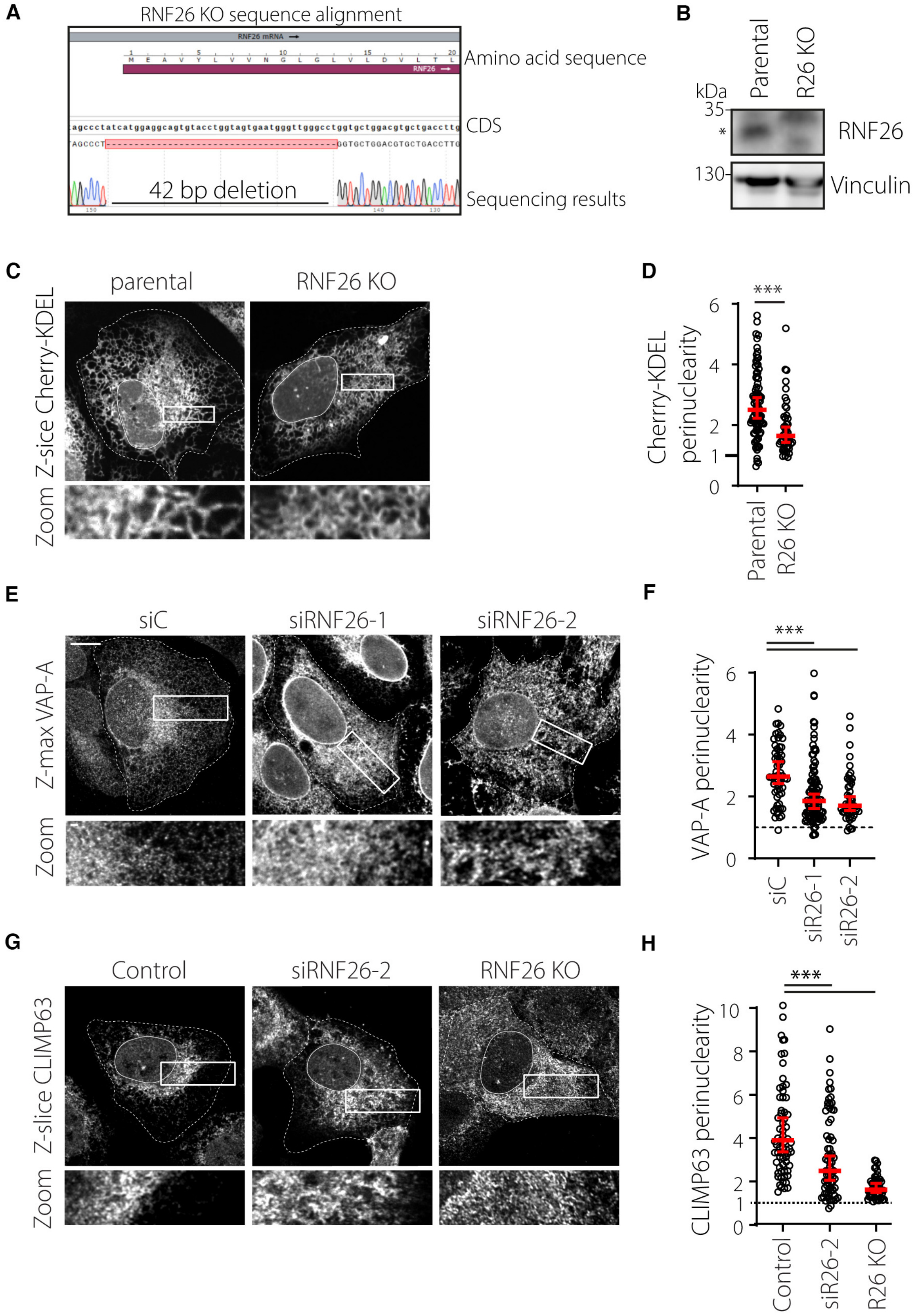

Figure EV1.

© 2023 The Authors

Figure EV2. (Related to Fig 2): Recombinant RNF26 binds vimentin *in vitro* and identification and synthesis of C-terminal vimentin binding peptide.

A Identification of interactors of the RNF26 cytoplasmic tail, originally described in and (Fig 2B). Shown is a photograph of the silver-stained SDS–PAGE gel from specific bands were cut out and analyzed by LC–MS. Mass-spec analysis results can be accessed via PRIDE#040760 and correspond to the numbers on this photograph.

B Affinity purification of recombinant RNF26 304–432 fused to a C-terminal GST-3C-Strep tag from *E. coli*. P—pellet, S—soluble fraction, FT—flow-through, E—elution fraction. GST domain was cleaved off using overnight 3C digestion before incubation with NHS-Rhodamine and use in downstream experiments.

C Deposition of RNF26 tail region on assembled vimentin filaments as in (Fig 1H), using GST-3C-STREP-RNF26 304–432 and his-Vim. His-tagged vimentin was (+NaCl) or was not (−NaCl) allowed to assemble into filaments for 1 h in PBS ± 100 mM NaCl at RT in wells containing standard coverslips before addition of GST-3C-STREP-RNF26 RING or not. After wash-out of excess RNF26, coverslips were fixed in PFA, blocked, and immunostained with anti-his and anti-GST antibodies. Samples were imaged on a spinning-disk confocal microscope. Shown are single channel panels of GST- and his- signals. Scale bar = 1 μM.

D Homology analysis of RNF26 C-terminus (aa 419–433) in H. Sapiens.

E Alphafold structure prediction of RNF26 RING domain. C-terminal vimentin interaction peptide (422–433) is indicated.

F Analysis of synthesized RNF26 C terminal peptide. Shown are *m/z* spectra of LC–MS analyzed peptide RNF26 422–433.

© 2023 The Authors

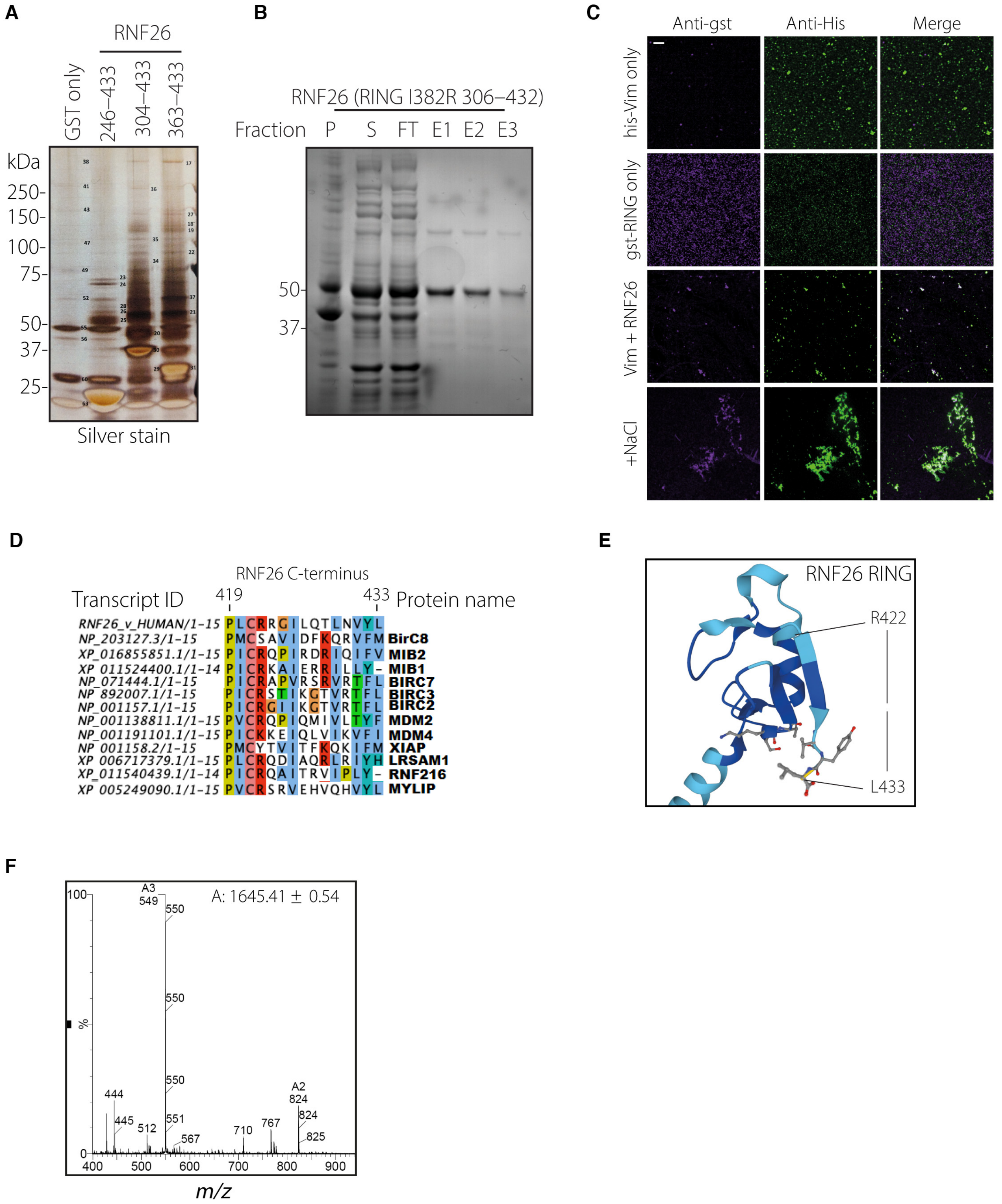

Figure EV2.

 © 2023 The Authors

Figure EV3. (Related to Fig 4): Supporting data on vimentin-mediated RNF26 and endosome organization.

A, B Knockout validation and RNF26 distribution in HeLa Vim KO cells. (A) Representative image of HeLa Vim KO cells that were transfected with vimentin-IRES-GFP and RFP-RNF26 I382R. Shown are merged images of GFP and RFP-RNF26 signals with indicated zoom ins of (1) a GFP-negative Vim KO#1 cell and (2) a GFP-positive vimentin re-expressing cell, showing vimentin-dependent filamentous perinuclear distribution of RNF26 I382R. (B) Validation of vimentin knockout. Representative immunoblots against endogenous vimentin and Vinculin (loading control) of a Vim KO clone and their parental HeLa cells are shown. Molecular weight markers are indicated.

C Analysis of inactive RNF26 I382R lacking its C-terminal vimentin binding motif with respect to vimentin and VAP-A. Representative image of a U2OS cell transfected with RFP-RNF26 I382R/ΔC and immunostained for endogenous vimentin and VAP-A. Images show overlay of either vimentin (green) or VAP-A (green) with RFP signals, zoom-ins of overlay and single channels that indicate aggregation of RNF26 in vimentin-negative/VAP-A positive regions.

D, E Effect of vimentin depletion on the intracellular distribution early endosomes. (D) U2OS cells transfected with the indicated siRNAs were fixed and immunostained against endogenous EEA1. (E) Quantification of signal distribution as perinuclearity ratio for EEA1 ($n_{\text{Parental}} = 69$, $n_{\text{Vim KO}} = 74$) technical replicates from *t* independent experiments.

F–H Effect of vimentin knockout on the intracellular distribution of endolysosomes in another knockout clone. (G) Validation of vimentin knockout. Representative immunoblots against endogenous vimentin and Vinculin (loading control) of a Vim KO clonal cell line and their parental U2OS cells are shown. Same blot as in Fig 4B. Molecular weight markers are as indicated. (E) Representative confocal images of parental and vimentin KO#2 U2OS cells, fixed and immunostained against endogenous LAMP1. Parental image same as in Fig 4E. (H) Quantification of LAMP1 signal distribution expressed as perinuclearity ratio. Graph reports on $n_{\text{PARENTAL}} = 132$, $n_{\text{Vim KO\#2}} = 96$ technical replicates from three independent experiments. Values were capped at 6.

Data information: Graphs report median (red line) and 95% confidence interval (error bars) of sample values (open circles). Scale bar = 10 μm. Significance was assessed using a Mann–Whitney U test; ***$P < 0.001$.

© 2023 The Authors

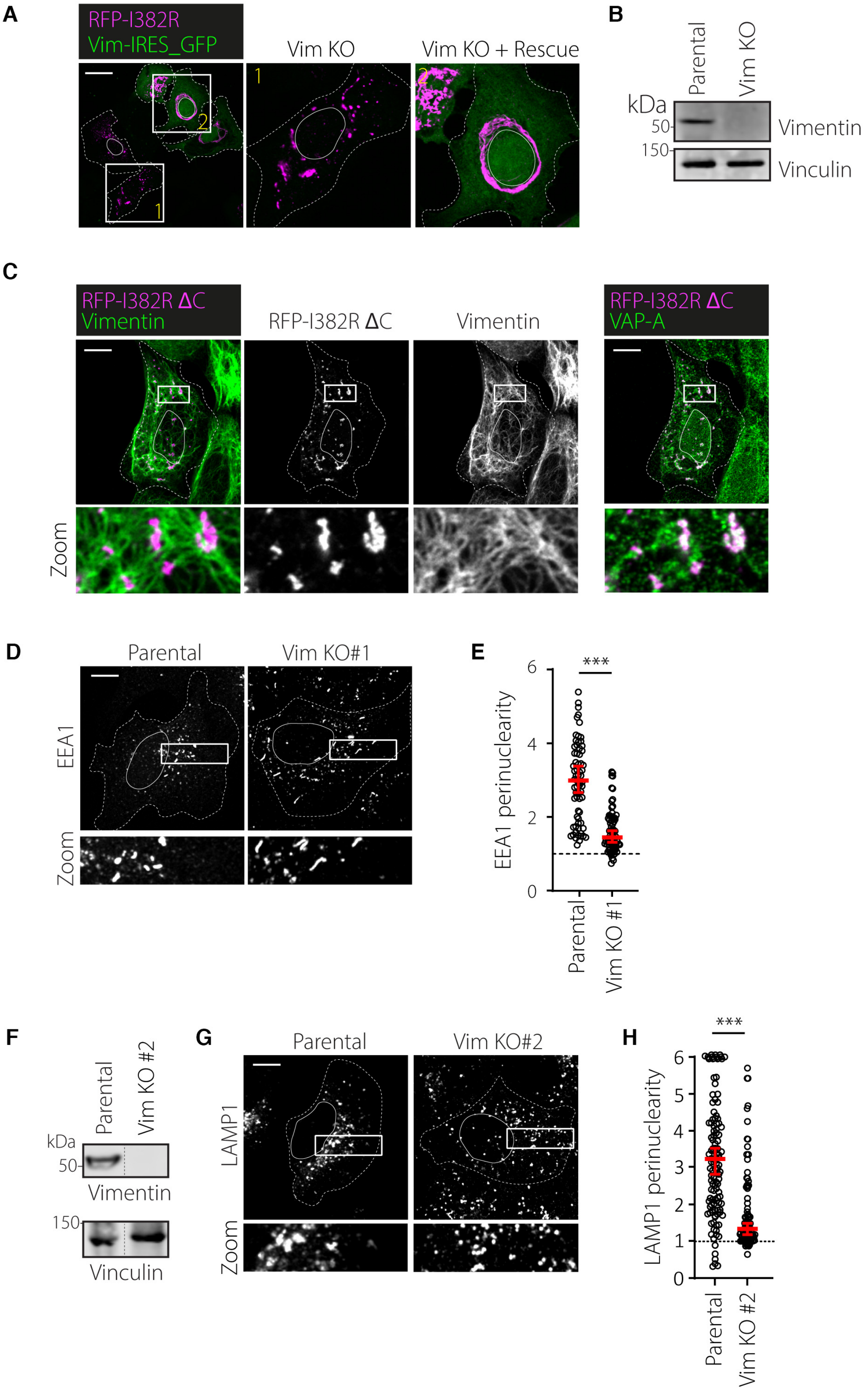

Figure EV3.

 © 2023 The Authors

Figure EV4. (Related to Fig 5): vimentin IFs interact with and organize perinuclear ER membranes.

A Zero tilt EM image of a perinuclear zoom-in region showing raw data used for tomography in (Fig 5A). Pseudo-colored annotation of the ER (cyan), mitochondria (red), lysosome (green) Golgi apparatus (magenta), and intermediate filaments (yellow) as manually drawn in. Scale bar = 1 µm.

B Examples of vimentin-ER connections from select regions in (A) and adjoined serial sections used for tomography. Arrows indicate vimentin (yellow), ER structures (cyan) and densities that bridged both elements (white). Scale bar = 100 nm.

C Zero-tilt EM image of region showing coalescence of microtubules, actin fibers, and Ifs. We discriminate between the three cytoskeletal elements based on their thickness and general appearance (left). While microtubules have a thick (~24.3 nm) and hollow tube-like appearance, actin filaments often appear branched are thin (~7.8 nm), and IFs are sized in between (~12.7 nm) and are not branched but appear as long stretches. Our identification method was validated by the lack of IFs in Vim KO U2OS cells, whereas the actin and microtubule cytoskeleton remained detectable (right).

D–G Effect of vimentin ablation on the intracellular distribution of ER markers. Parental and two Vim KO U2OS cell lines were fixed and immunostained against endogenous (D) VAP-A or (F) CLIMP63. Representative confocal fluorescence images are shown with boxed zoom-ins highlighting perinuclear regions. Cell and nuclear boundaries are demarcated using dashed and continuous lines, respectively. Quantification of signal distribution as perinuclearity ratio for (E) VAP-A ($n_{\text{Parental}} = 95$, $n_{\text{Vim KO\#1}} = 55$, $n_{\text{Vim KO\#2}} = 26$ technical replicates) and (G) CLIMP63 ($n_{\text{Parental}} = 127$, $n_{\text{Vim KO\#2}} = 69$ technical replicates) from three independent experiments.

Data information: Graphs report median (red line) and 95% confidence interval (error bars) of sample values (open circles). Scale bar = 10 µm. Significance was assessed using a Mann–Whitney U test; *P < 0,05, ***P < 0.001.

© 2023 The Authors

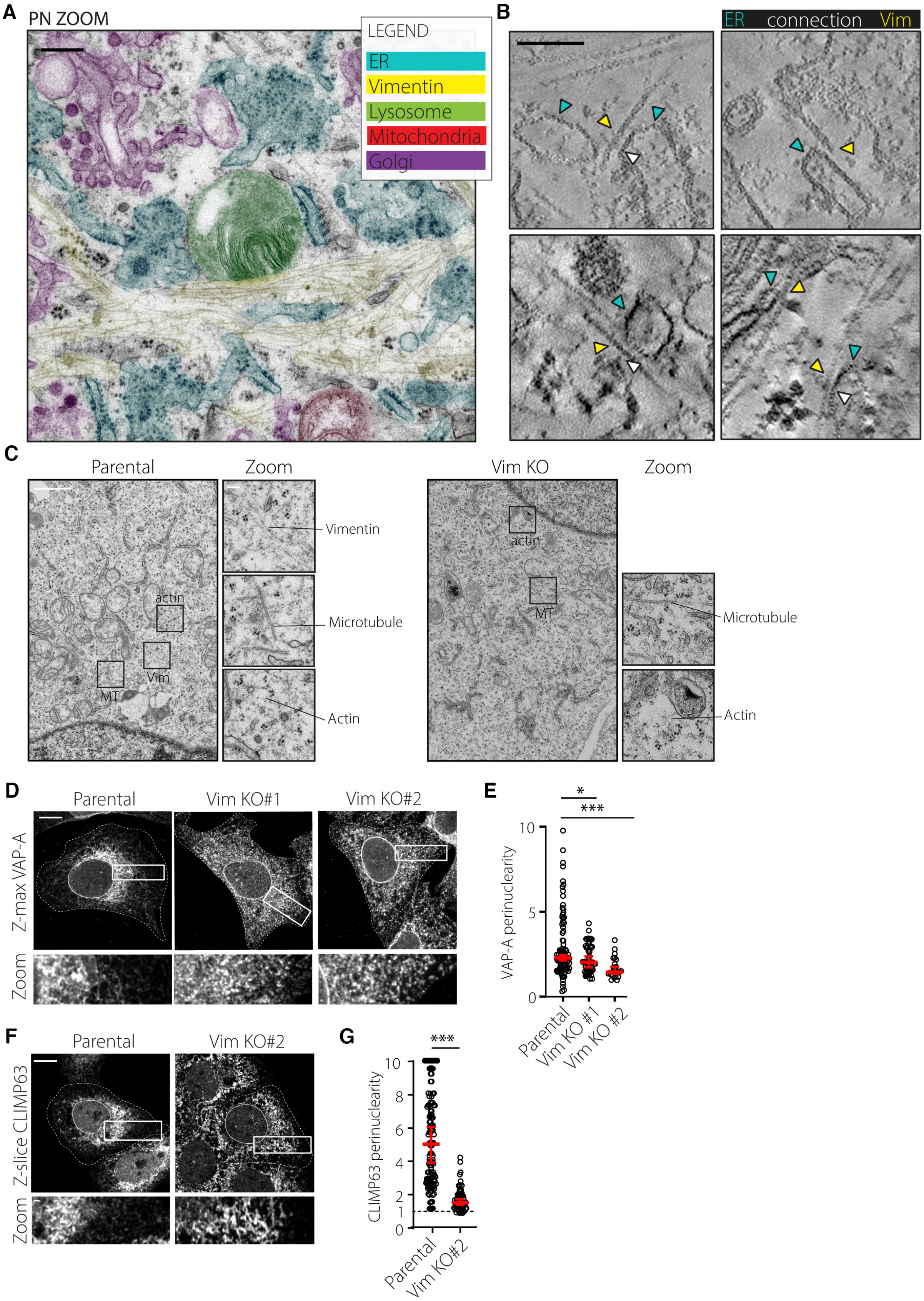

Figure EV4.

 © 2023 The Authors

Figure EV5. (Related to Fig 6): RNF26 and vimentin are required for ER and endosome organization during ER stress.

A, B Calnexin distribution under Tunicamycin-induced ER stress as a function of RNF26 or vimentin. (A) Shown are representative Z-slices of parental HeLa cells, HeLa Vim KO cells, or HeLa cells silenced for RNF26 (si#1 or #2) that are treated overnight with tunicamycin (5 μg/ml). (B) Perinuclearity analysis of Calnexin signal in cells from (A) (n_{siC} = 71, n_{siC+} = 58, $n_{siRNF26\#1}$ = 36, $n_{siRNF26\#2}$ = 41, $n_{Vim\ KO}$ = 68) technical replicates from three independent experiments.

C, D Tunicamycin-induced late endosome clustering as a function of RNF26. (D) Representative Z-slices of parental U2OS cells, U2OS RNF26 KO cells, or U2OS cells silenced for RNF26 (si#2) or HERP1 (pooled siRNAs), treated with tunicamycin (5 μg/ml) as indicated before fixation and immunostaining for CD63. (E) Perinuclearity analysis of CD63 signal in U2OS cells from (D). $n_{control-}$ = 113, $n_{control+}$ = 228, $n_{siHERP1}$ = 35, $n_{siRNF26\#1}$ = 86, $n_{R26\ KO}$ = 90 technical replicates from three independent experiments.

E, F Tunicamycin-induced UPR signaling as a function of RNF26 or vimentin. XBP1 and XBP1s (E) or BiP (F) transcripts isolated from U2OS cells transfected or genetically modified as indicated and treated in the absence (−) or presence (+) of tunicamycin (2.5 μg/ml, O/N) were detected by qPCR and normalized to GAPDH; $n_{siC\ (-)}$ = 4, $n_{siC\ (+)}$ = 4, $n_{siRNF26\#1\ (+)}$ = 3, $n_{siRNF26\#2\ (+)}$ = 4, $n_{PARENTAL\ (+)}$ = 4, $n_{Vim\ KO\ (+)}$ = 4 independent experiments (error bar = mean and SEM).

G Calnexin distribution under Cyclopiazonic acid-induced ER stress as a function of RNF26 or vimentin. Shown are representative images of siC, Vim KO#2 cells and RNF26 depleted (si#2) U2OS cells treated with cyclopiazonic acid (10 μM) overnight, stained for calnexin and imaged by confocal microscopy.

Data information: Cell and nuclear boundaries are demarcated using dashed and continuous lines, respectively. Scale bar = 10 μm. Significance in (B) and (D) was assessed using a Mann–Whitney U test (error bars reflecting median and 95% confidence interval), and Student's *t*-test (E, F with error bars reflecting mean ± SD); **$P < 0.01$, ***$P < 0.001$, ****$P < 0.0001$.

© 2023 The Authors

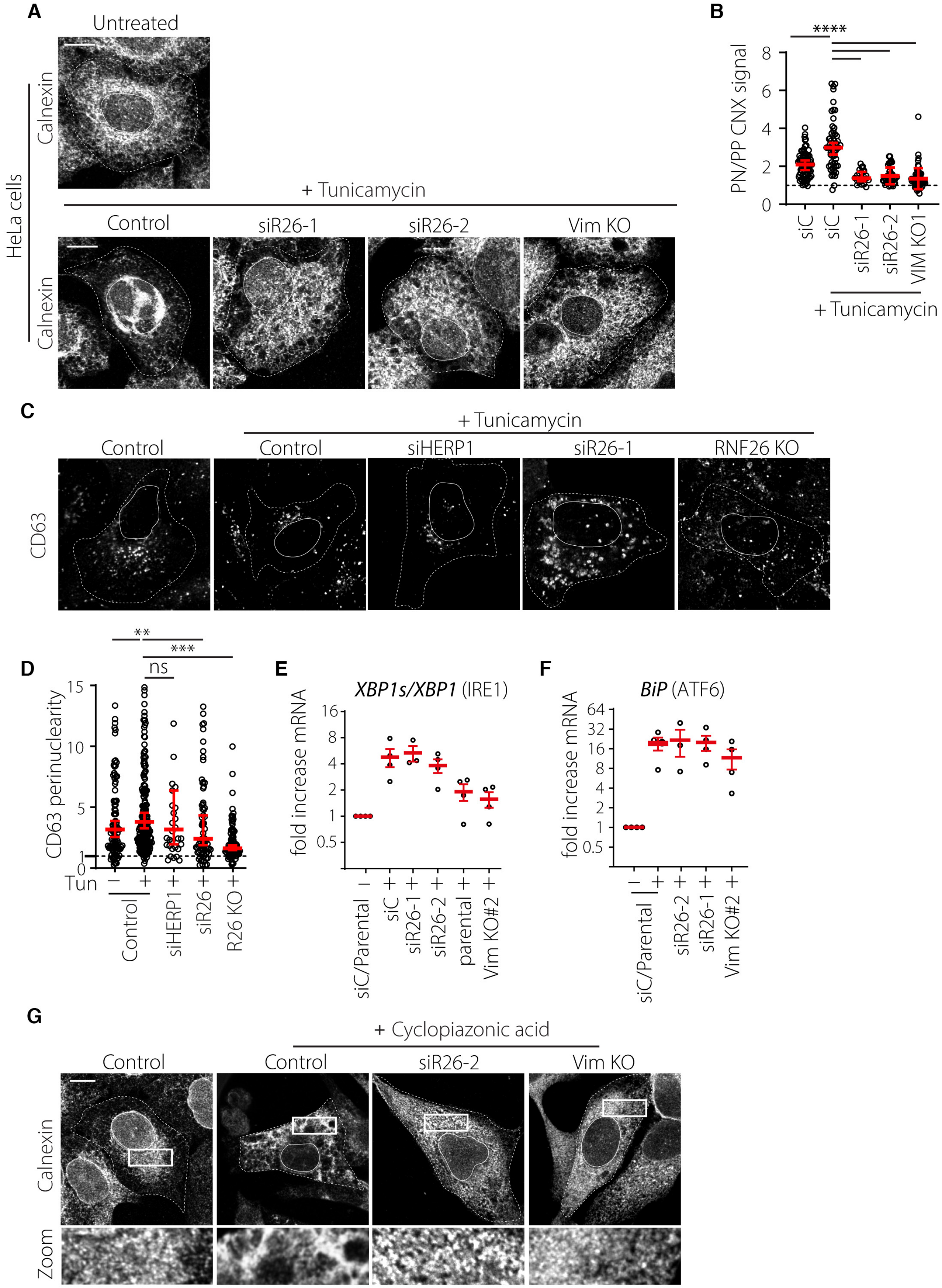

Figure EV5.

© 2023 The Authors
