# Supplementary figures and images for: RNF26 binds perinuclear vimentin filaments to integrate ER and endolysosomal responses to proteotoxic stress
Source: EMBO J. 2023 Jul 31;42(18):e111252. doi: 10.15252/embj.2022111252 (PMC10505911; doi:10.15252/embj.2022111252)

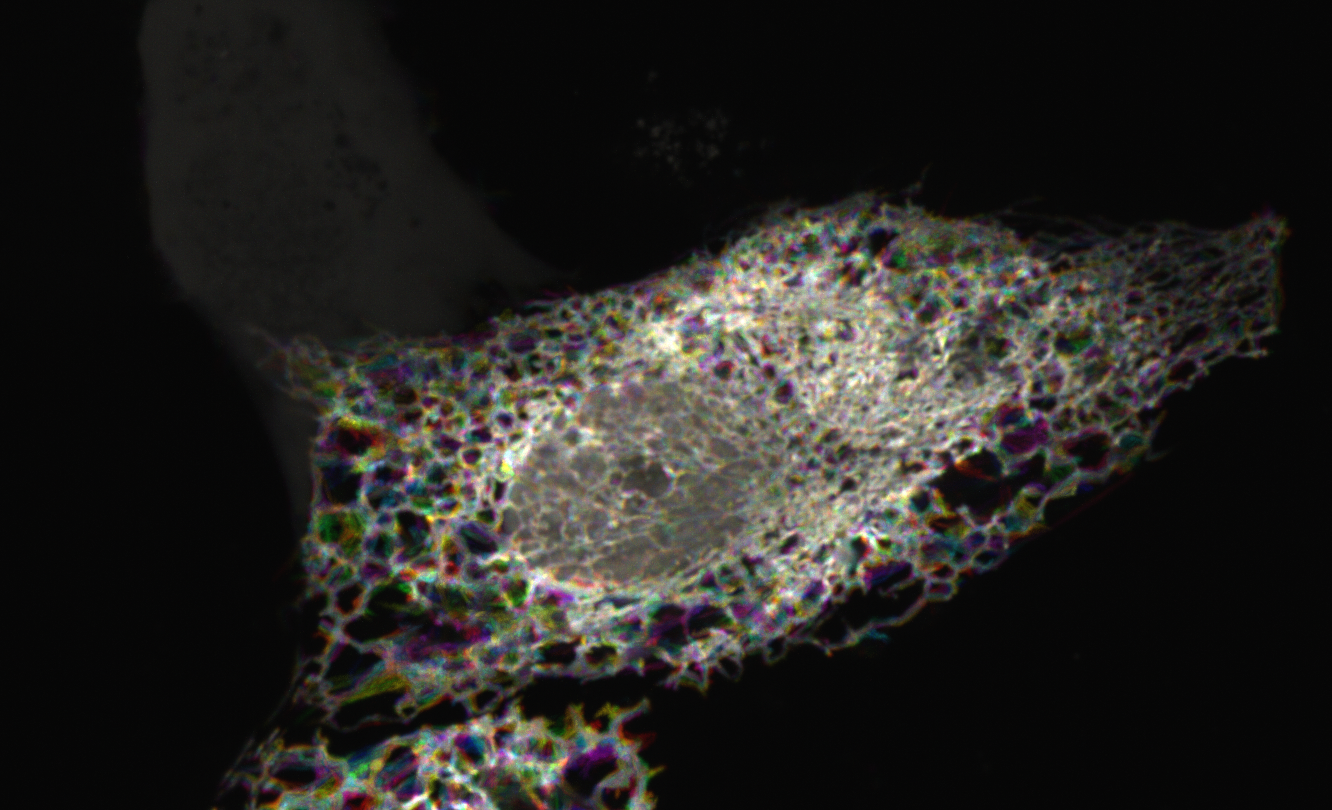

Supplement: Supplementary file 12 — Source Data for Figure 1 [file EMBJ-42-e111252-s008.zip › Figure 1/1B/siC TCC.tif]

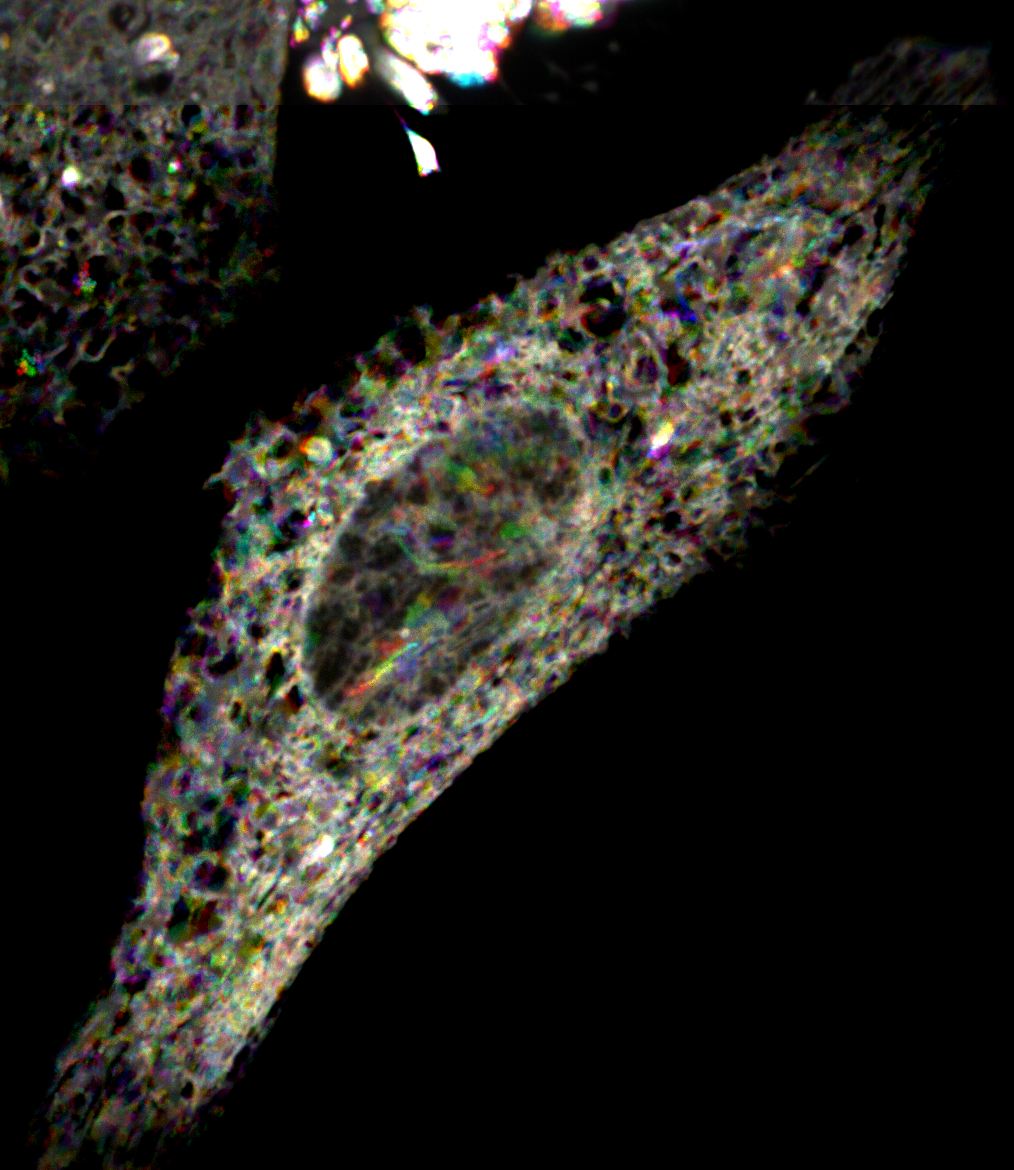

Supplement: Supplementary file 12 — Source Data for Figure 1 [file EMBJ-42-e111252-s008.zip › Figure 1/1B/siR26 TCC.tif]

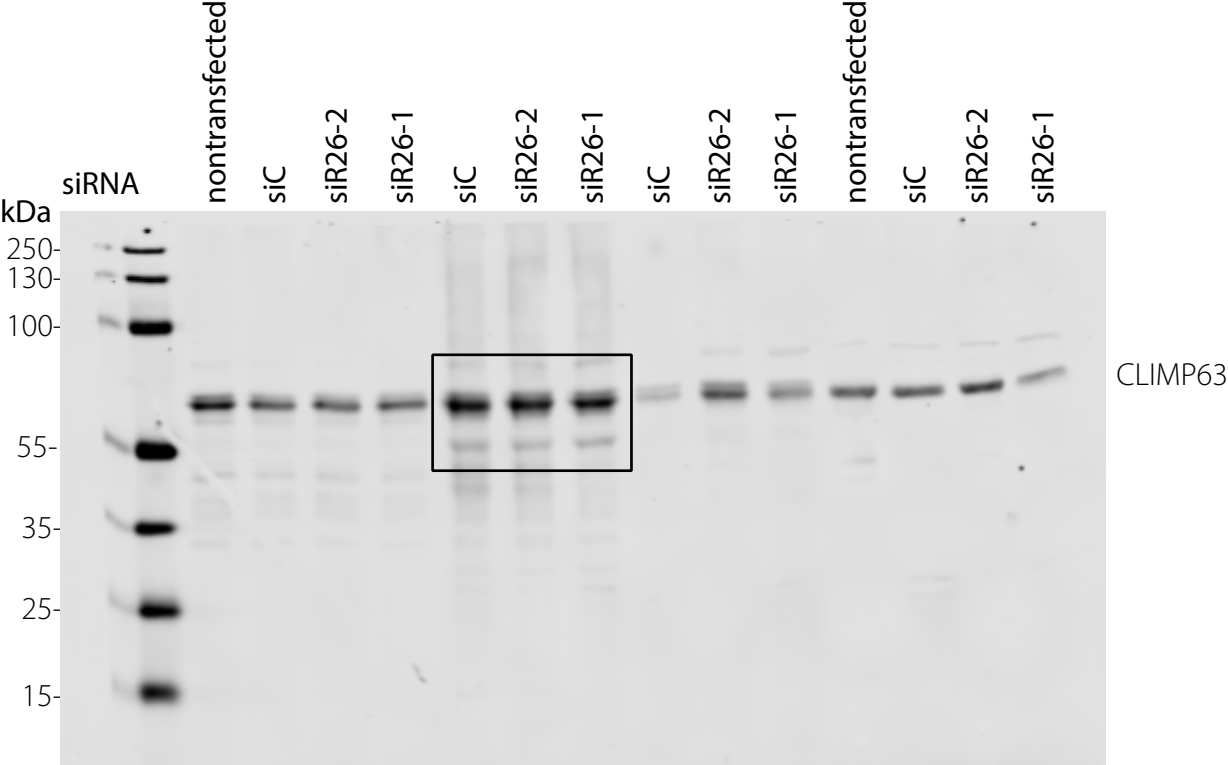

Supplement: Supplementary file 12 — Source Data for Figure 1 [file EMBJ-42-e111252-s008.zip › Figure 1/1I/CLIMP63.pdf]

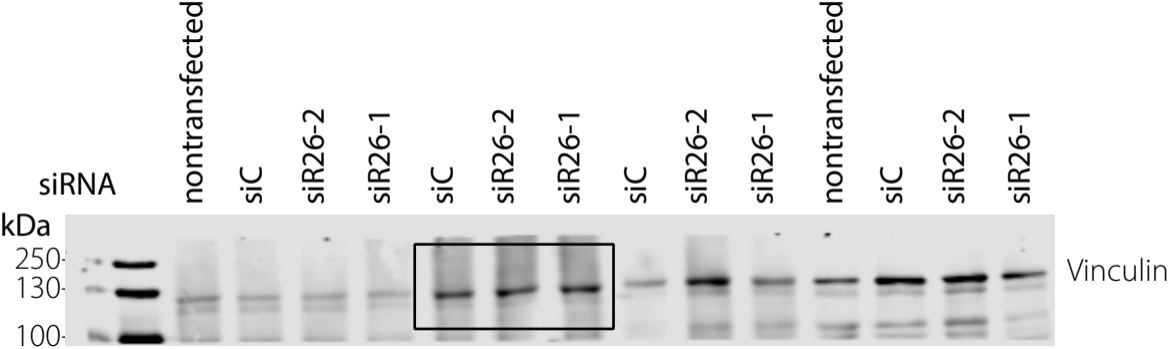

Supplement: Supplementary file 12 — Source Data for Figure 1 [file EMBJ-42-e111252-s008.zip › Figure 1/1I/Vinculin.pdf]

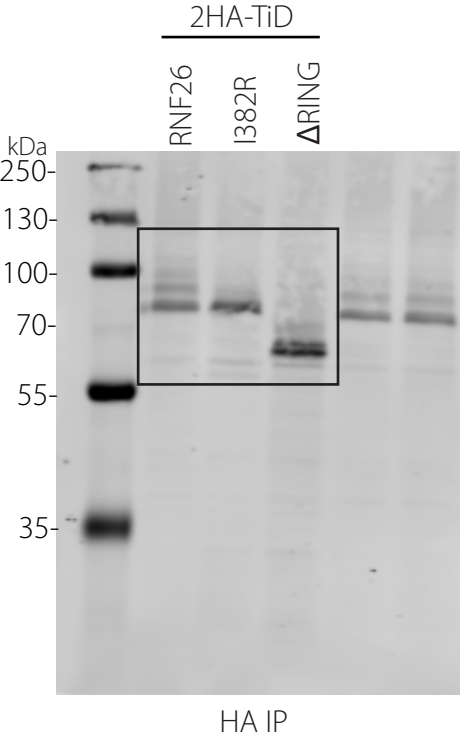

Supplement: Supplementary file 13 — Source Data for Figure 2 [file EMBJ-42-e111252-s020.zip › Figure 2/2C/HA pulldown.pdf]

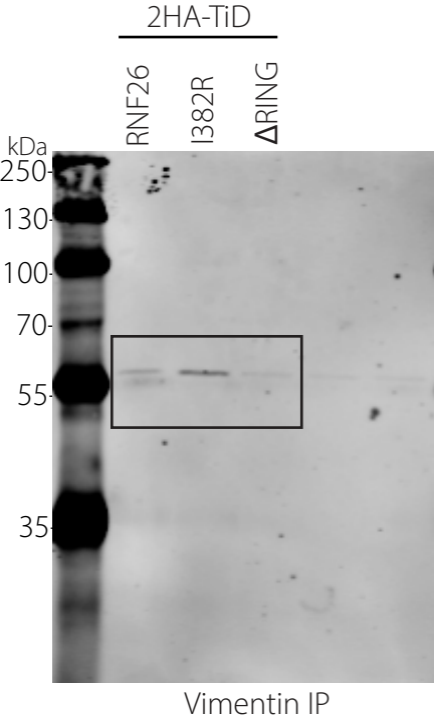

Supplement: Supplementary file 13 — Source Data for Figure 2 [file EMBJ-42-e111252-s020.zip › Figure 2/2C/Vimentin pulldown.pdf]

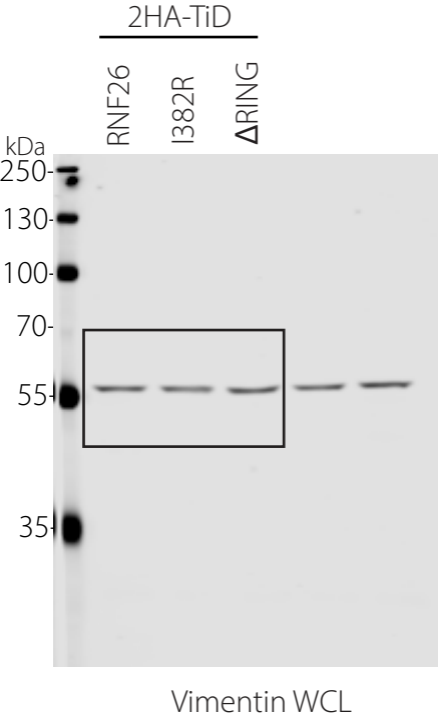

Supplement: Supplementary file 13 — Source Data for Figure 2 [file EMBJ-42-e111252-s020.zip › Figure 2/2C/Vimentin WCL.pdf]

|                |   |   |       |
|----------------|---|---|-------|
| Rho-RING-I382R | + | + | input |
| His-Vimentin   | - | + |       |

250-  
130-  
100-  
70-  
55-  
35-  
25-  
15-  
10-

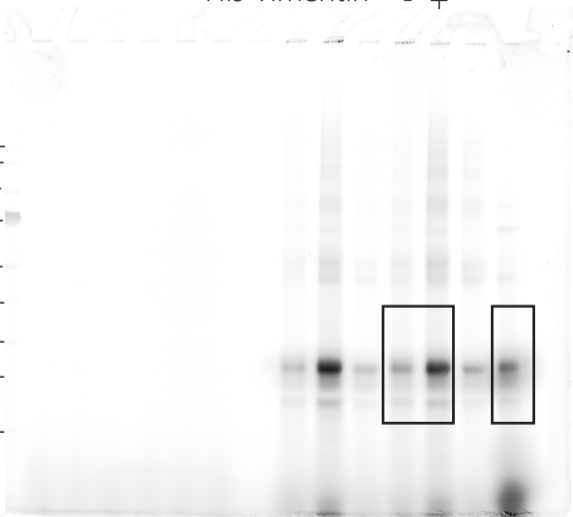

Rhodamine

Supplement: Supplementary file 13 — Source Data for Figure 2 [file EMBJ-42-e111252-s020.zip › Figure 2/2E/Fluoscan Rhodamine.pdf]

Rho-RING-I382R + +  
His-Vimentin - +

input

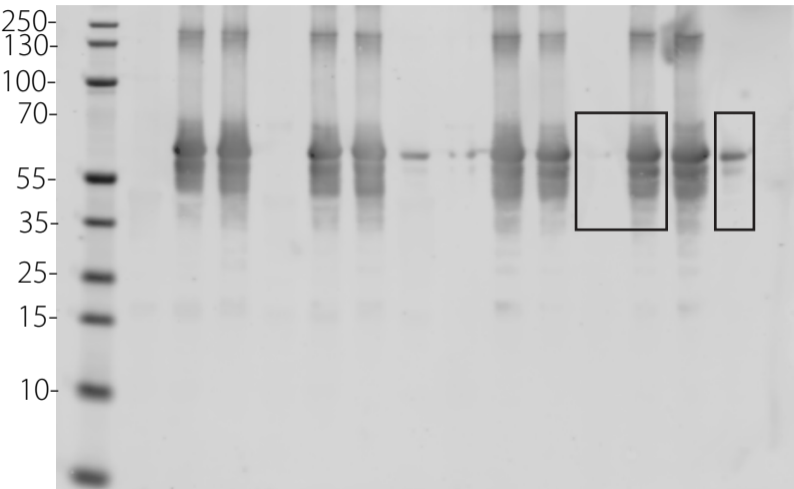

WB: His

Supplement: Supplementary file 13 — Source Data for Figure 2 [file EMBJ-42-e111252-s020.zip › Figure 2/2E/WB his.pdf]

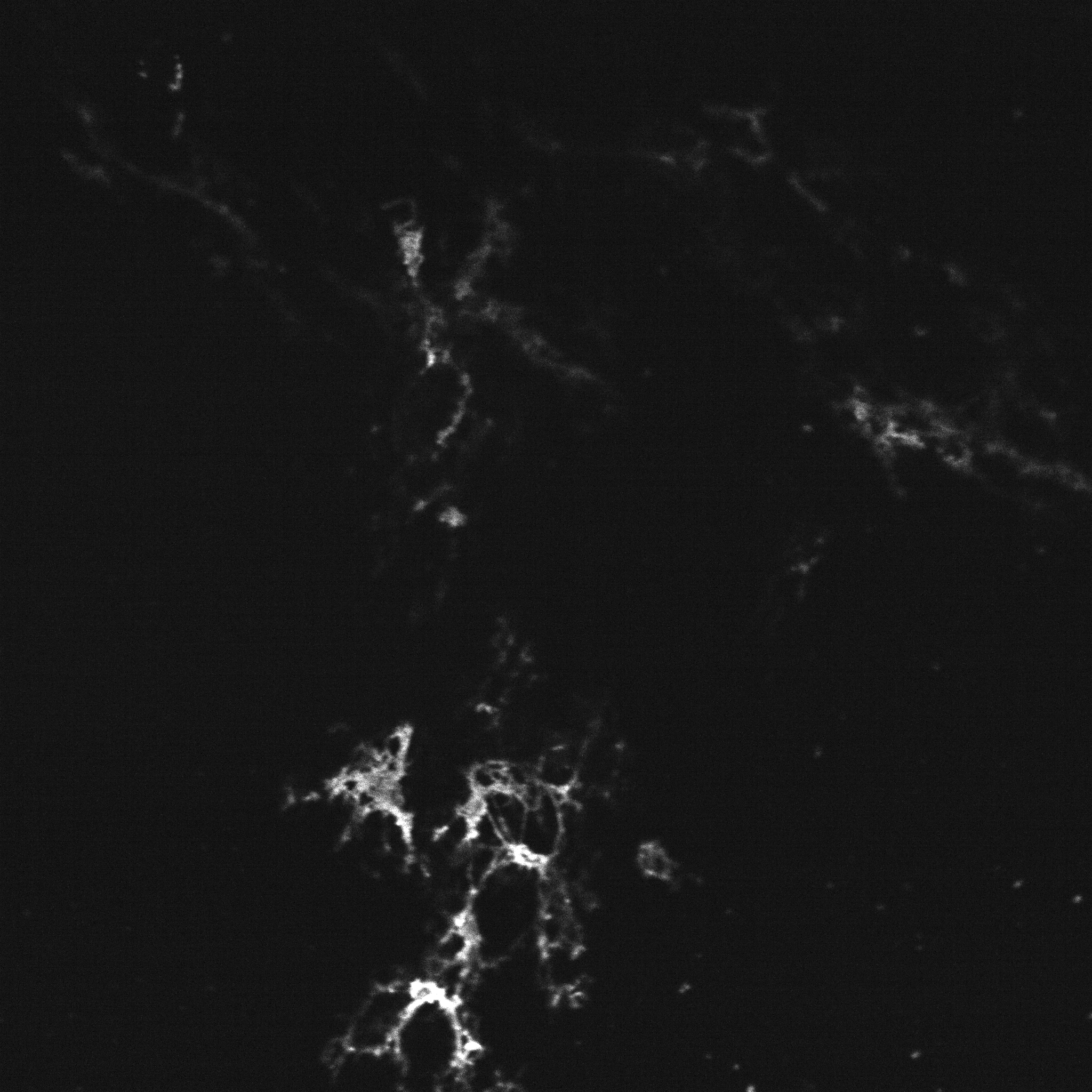

Supplement: Supplementary file 13 — Source Data for Figure 2 [file EMBJ-42-e111252-s020.zip › Figure 2/2F/Composite his-Vim + Rho-RNF26 RING I382R + NaCl.tif]

2HA-TiD-RNF26

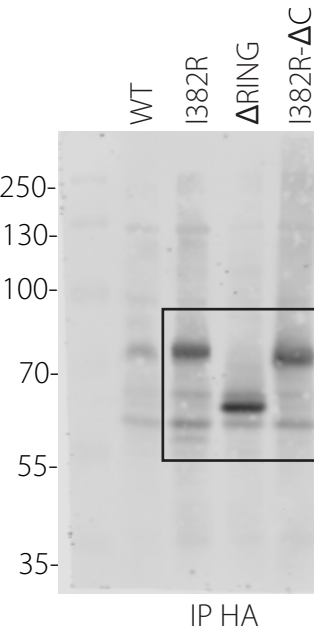

Supplement: Supplementary file 13 — Source Data for Figure 2 [file EMBJ-42-e111252-s020.zip › Figure 2/2G/HA pulldown.pdf]

# 2HA-TiD-RNF26

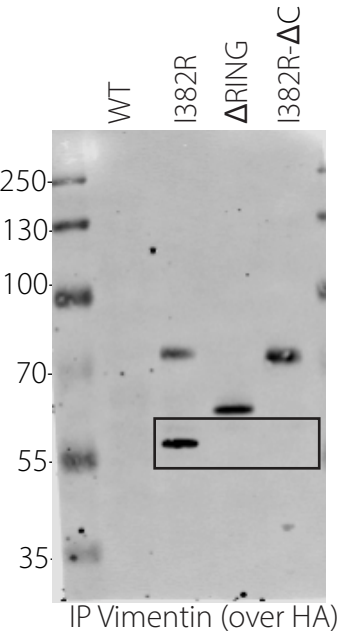

Supplement: Supplementary file 13 — Source Data for Figure 2 [file EMBJ-42-e111252-s020.zip › Figure 2/2G/Vimentin pulldown.pdf]

2HA-TiD-RNF26

WT

I382R

$\Delta$ RING

I382R- $\Delta$ C

250-  
130-  
100-  
70-  
55-  
35-

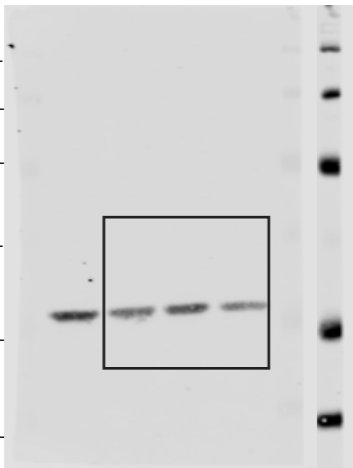

WCL Vimentin

Supplement: Supplementary file 13 — Source Data for Figure 2 [file EMBJ-42-e111252-s020.zip › Figure 2/2G/Vimentin WCL.pdf]

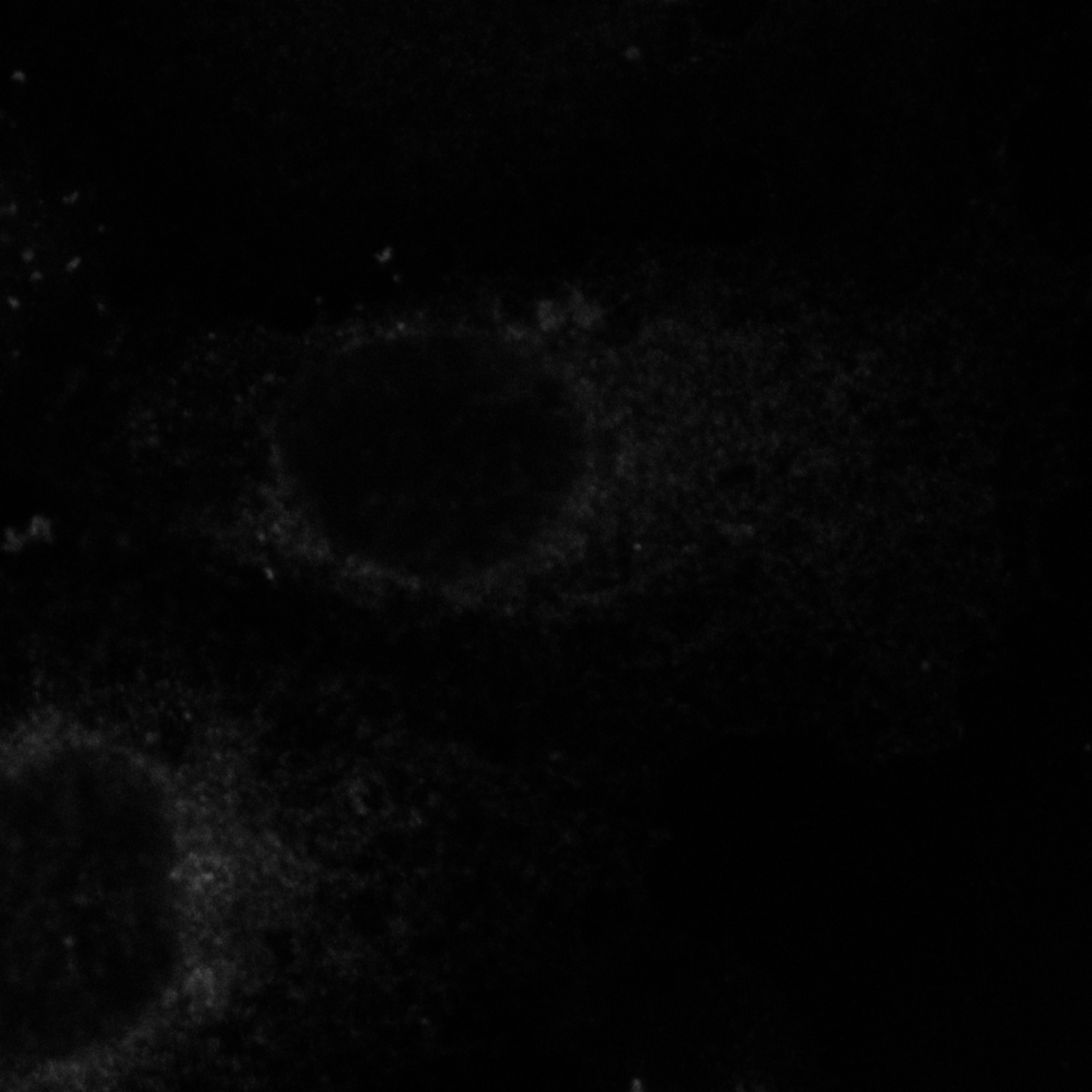

Supplement: Supplementary file 14 — Source Data for Figure 3 [file EMBJ-42-e111252-s002.zip › Figure 3/3A/Composite RNF26 deltaC Vimentin VAPA.tif]

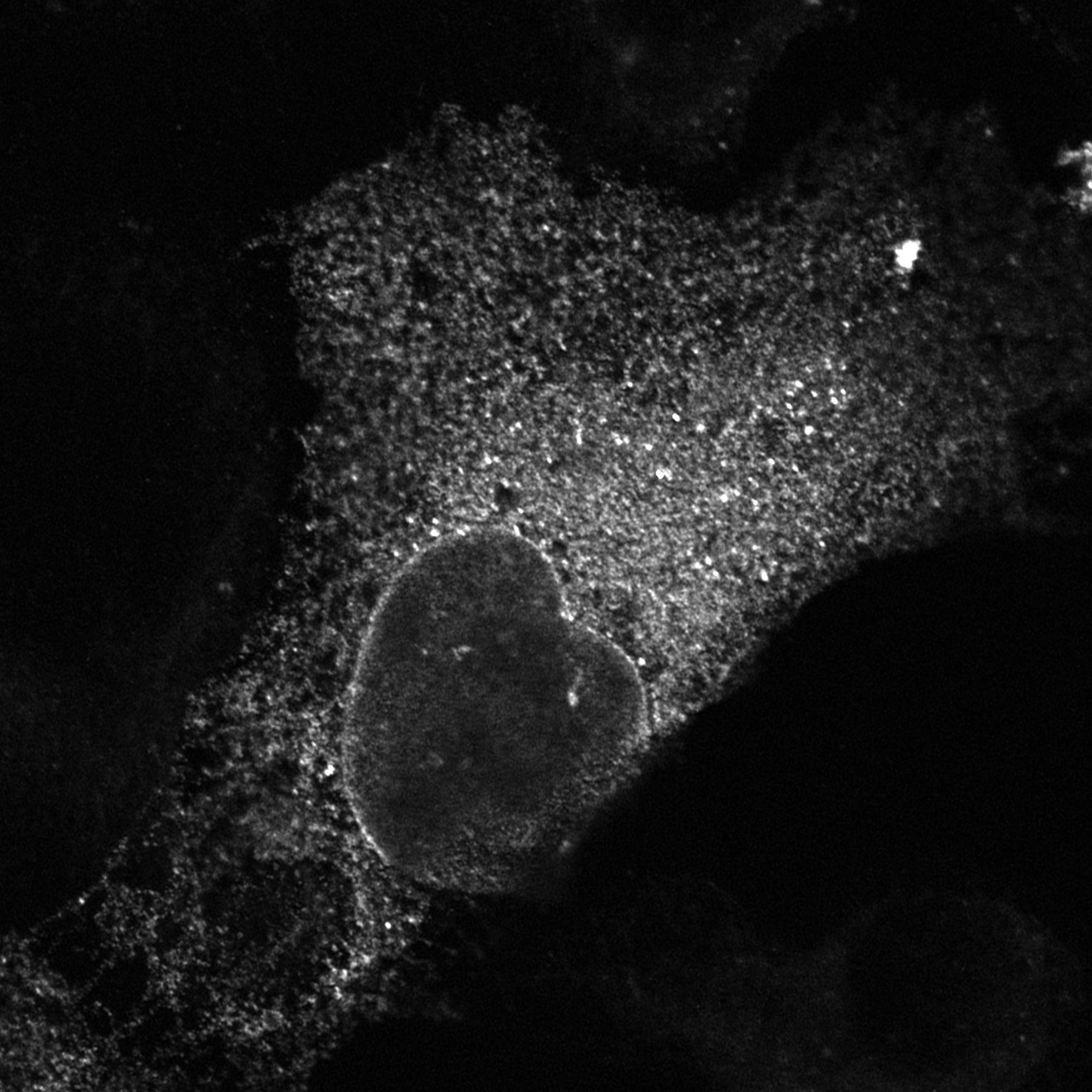

Supplement: Supplementary file 14 — Source Data for Figure 3 [file EMBJ-42-e111252-s002.zip › Figure 3/3A/Composite RNF26 deltaRING Vimentin VAPA.tif]

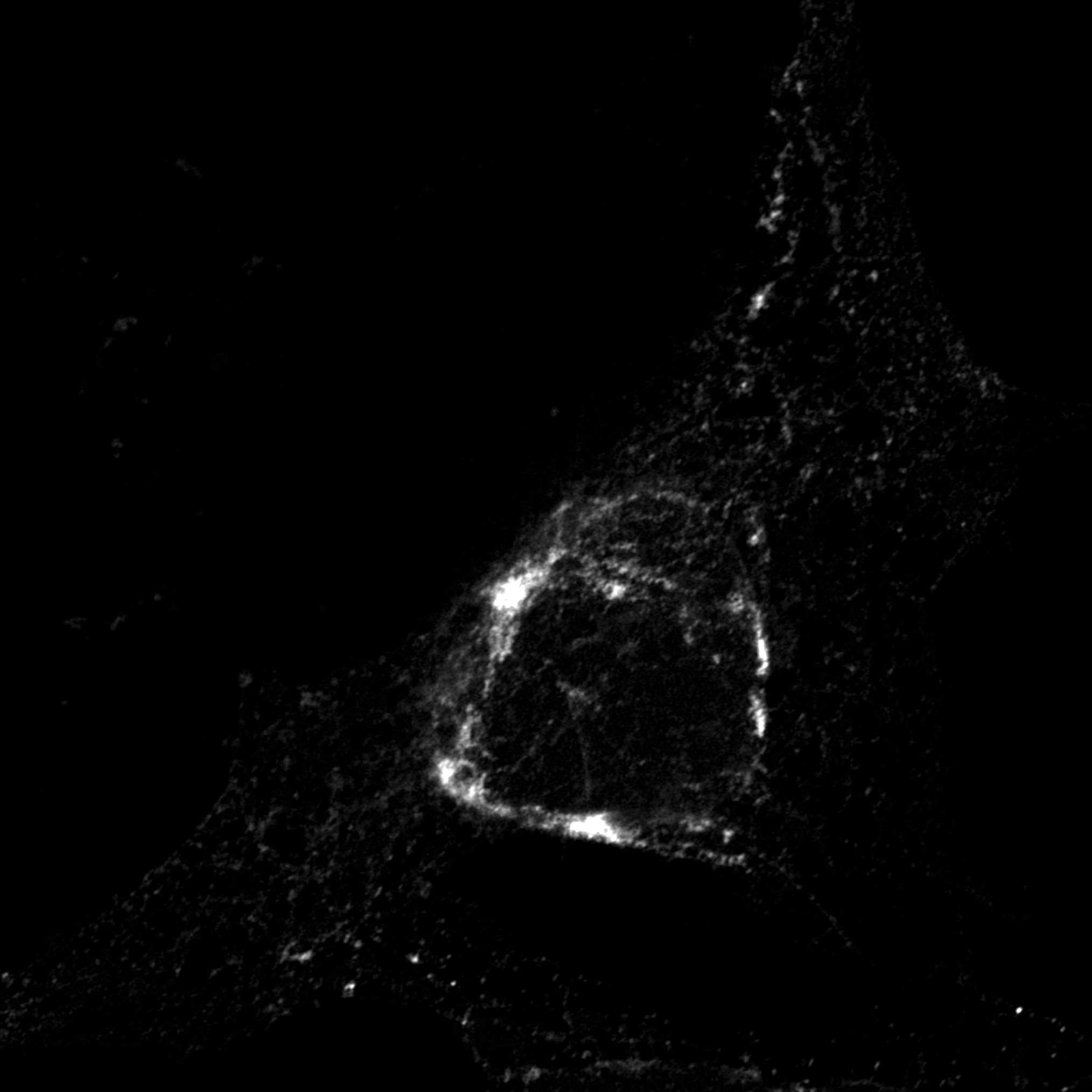

Supplement: Supplementary file 14 — Source Data for Figure 3 [file EMBJ-42-e111252-s002.zip › Figure 3/3A/Composite RNF26 IR Vimentin VAPA.tif]

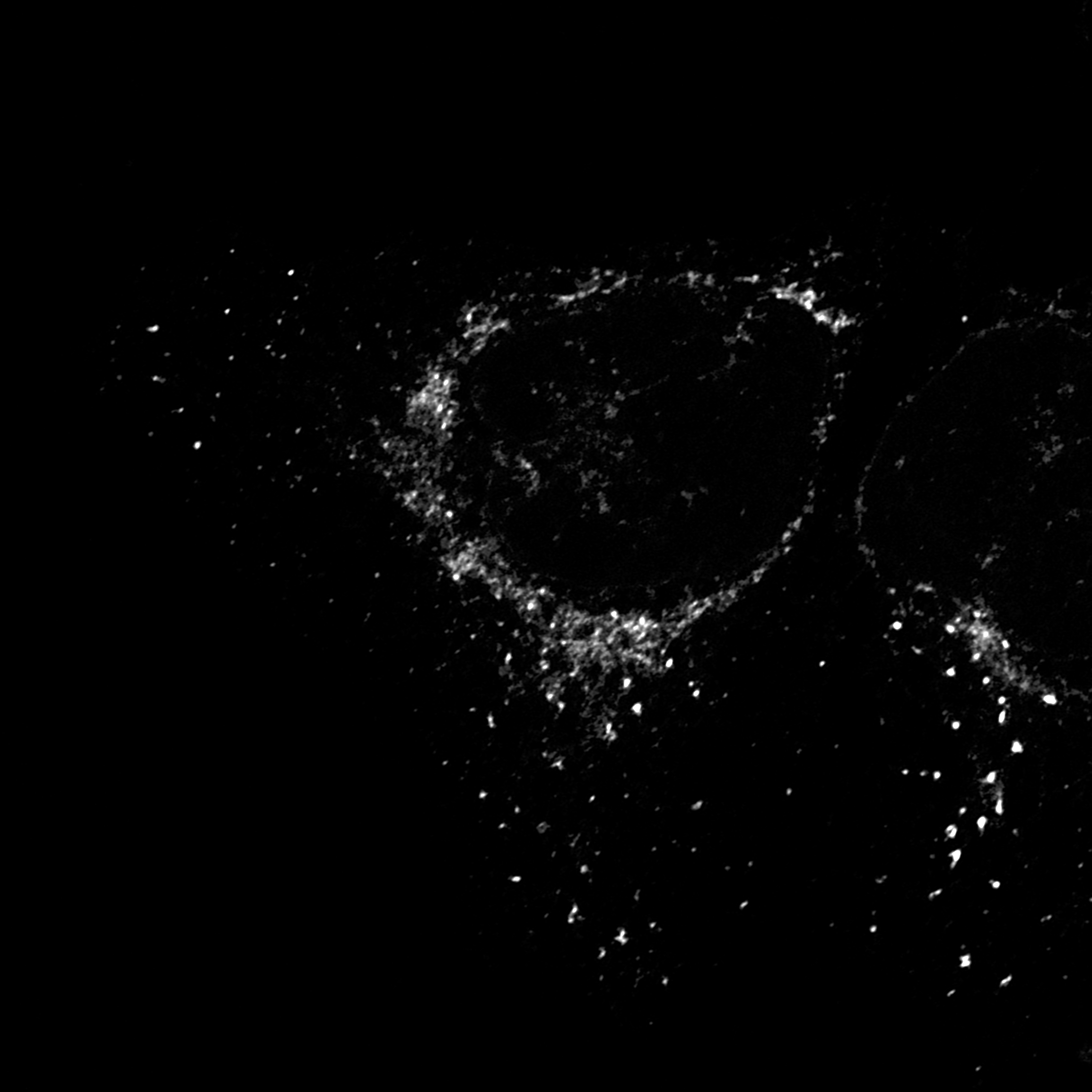

Supplement: Supplementary file 14 — Source Data for Figure 3 [file EMBJ-42-e111252-s002.zip › Figure 3/3A/Composite RNF26 WT Vimentin VAPA.tif]

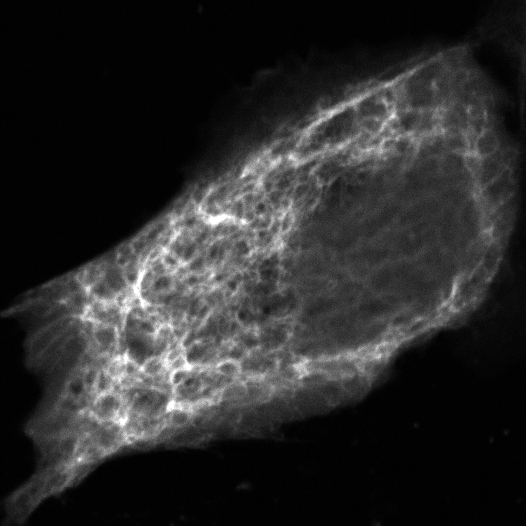

Supplement: Supplementary file 14 — Source Data for Figure 3 [file EMBJ-42-e111252-s002.zip › Figure 3/3F/RNF26 KO Vimentin.tif]

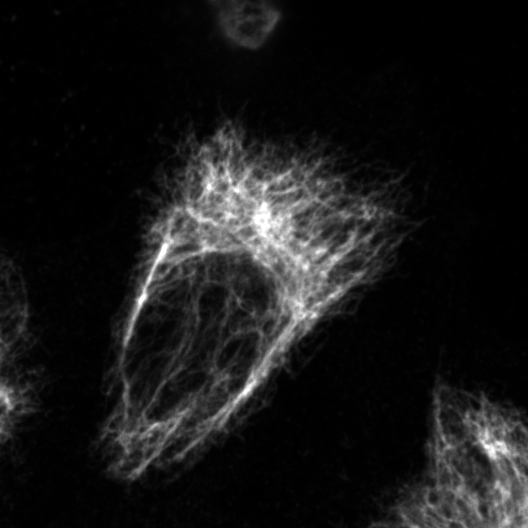

Supplement: Supplementary file 14 — Source Data for Figure 3 [file EMBJ-42-e111252-s002.zip › Figure 3/3F/siC Vimentin.tif]

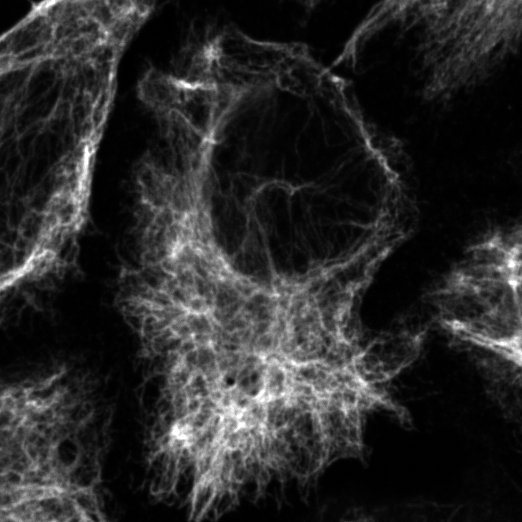

Supplement: Supplementary file 14 — Source Data for Figure 3 [file EMBJ-42-e111252-s002.zip › Figure 3/3F/siRNF26-1 Vimentin.tif]

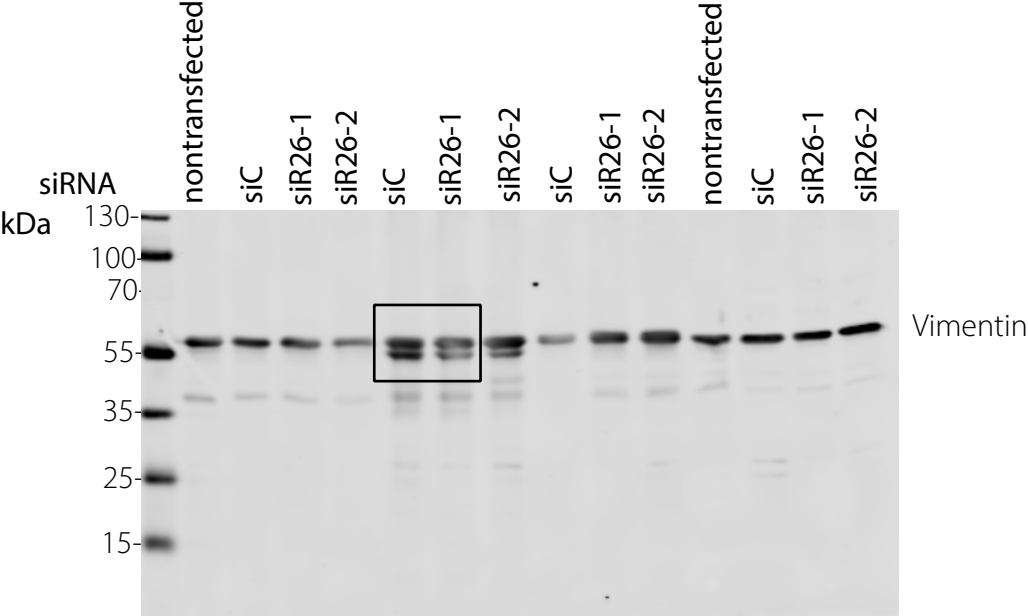

Supplement: Supplementary file 14 — Source Data for Figure 3 [file EMBJ-42-e111252-s002.zip › Figure 3/3H/Vimentin.pdf]

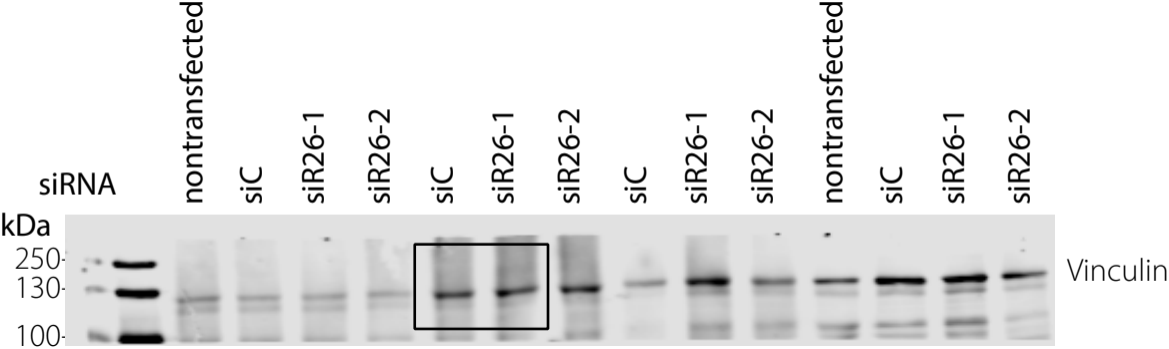

Supplement: Supplementary file 14 — Source Data for Figure 3 [file EMBJ-42-e111252-s002.zip › Figure 3/3H/Vinculin.pdf]

Parental

Vim KO #1

Vim KO #2

100

100

70

55

37

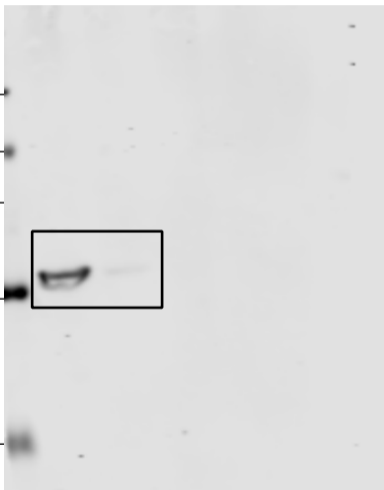

Vimentin

Supplement: Supplementary file 15 — Source Data for Figure 4 [file EMBJ-42-e111252-s016.zip › Figure 4/4B/WB Vimentin.pdf]

Parental

VimKO #1

Vim KO #2

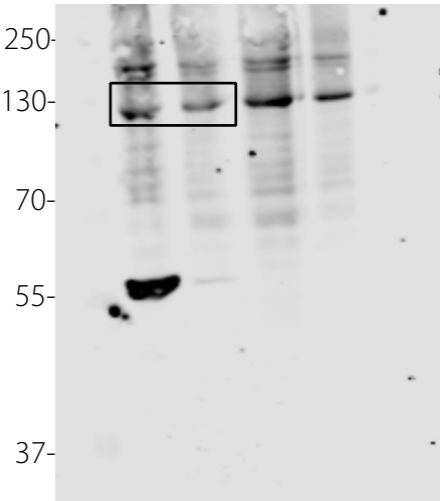

Vinculin

Supplement: Supplementary file 15 — Source Data for Figure 4 [file EMBJ-42-e111252-s016.zip › Figure 4/4B/WB Vinculin.pdf]

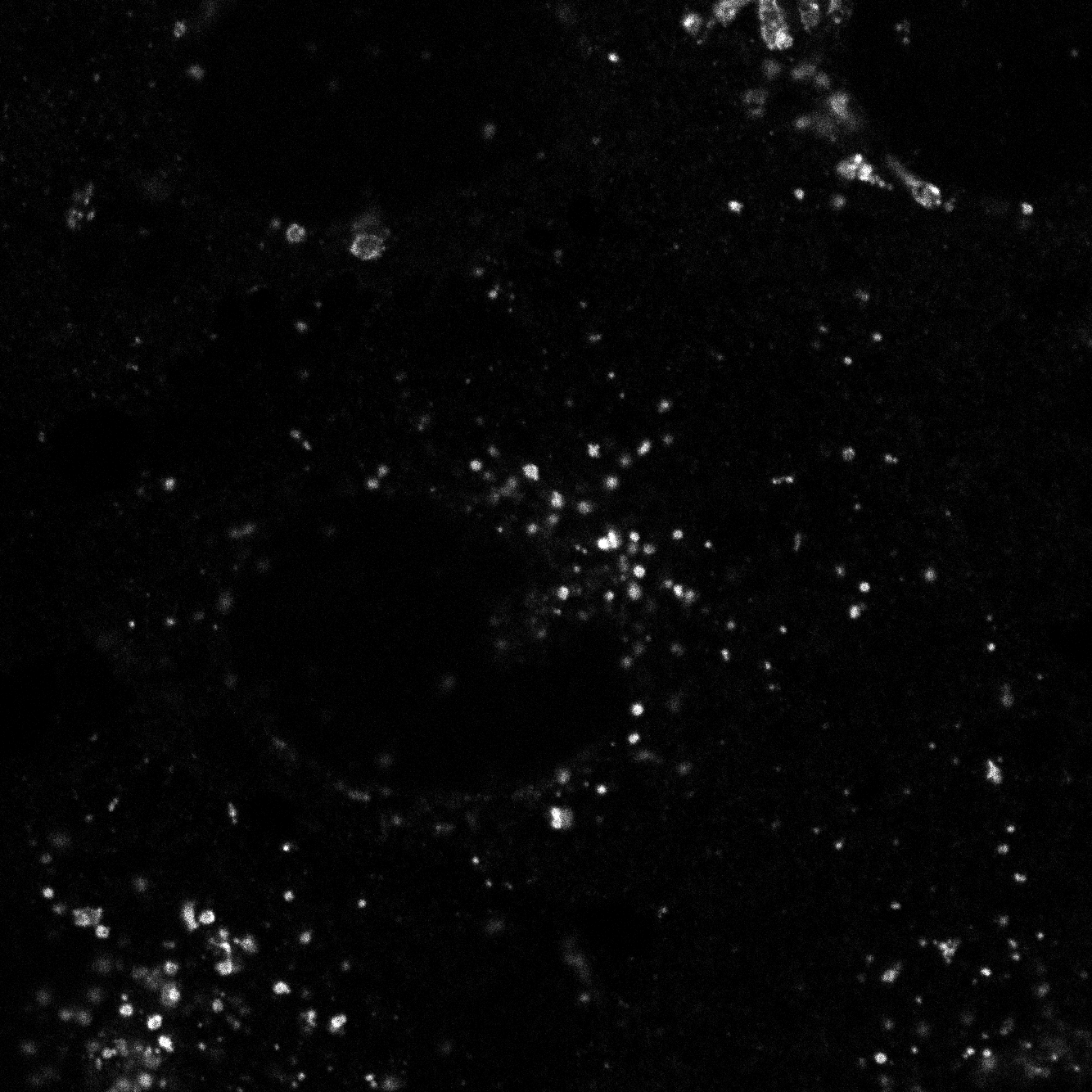

Supplement: Supplementary file 15 — Source Data for Figure 4 [file EMBJ-42-e111252-s016.zip › Figure 4/4E/Composite VIM KO Rescue LAMP1 Vimentin.tif]

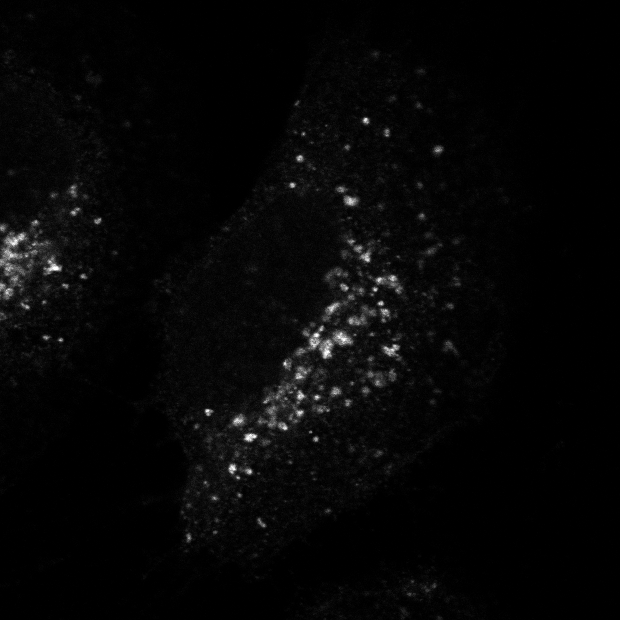

Supplement: Supplementary file 15 — Source Data for Figure 4 [file EMBJ-42-e111252-s016.zip › Figure 4/4E/Parental LAMP1.tif]

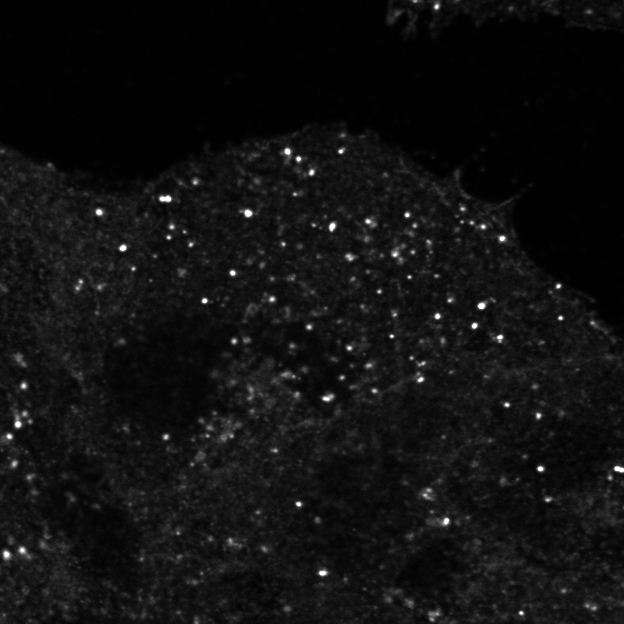

Supplement: Supplementary file 15 — Source Data for Figure 4 [file EMBJ-42-e111252-s016.zip › Figure 4/4E/RNF26 KO LAMP1.tif]

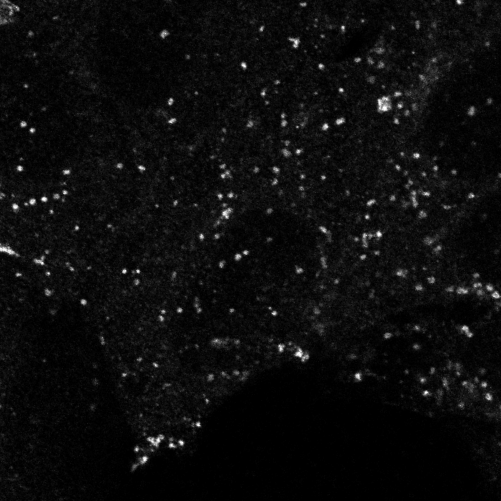

Supplement: Supplementary file 15 — Source Data for Figure 4 [file EMBJ-42-e111252-s016.zip › Figure 4/4E/Vim KO LAMP1.tif]

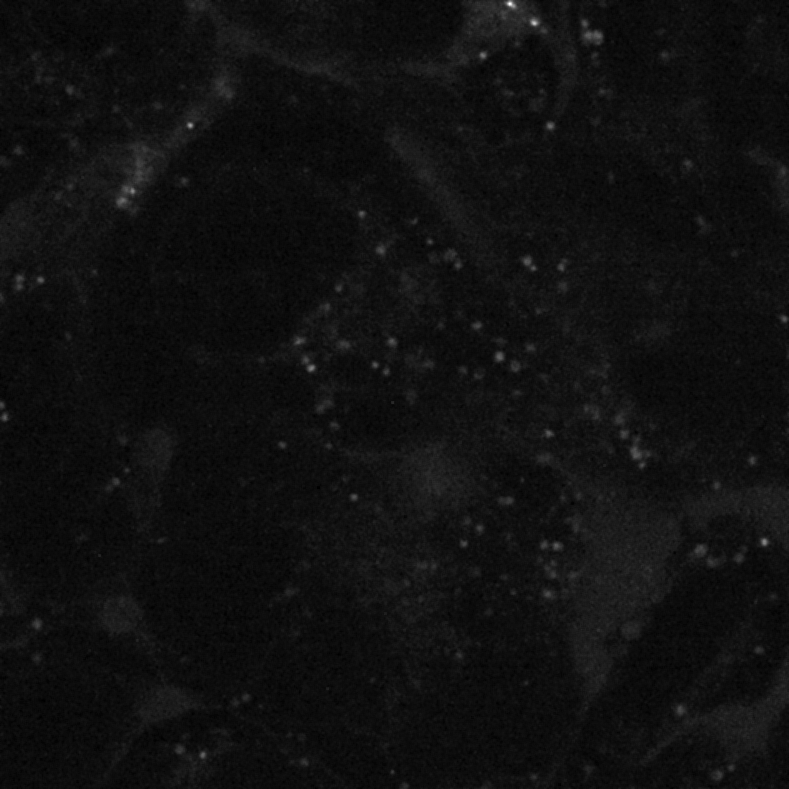

Supplement: Supplementary file 15 — Source Data for Figure 4 [file EMBJ-42-e111252-s016.zip › Figure 4/4G/Composite Vim KO t=120.tif]

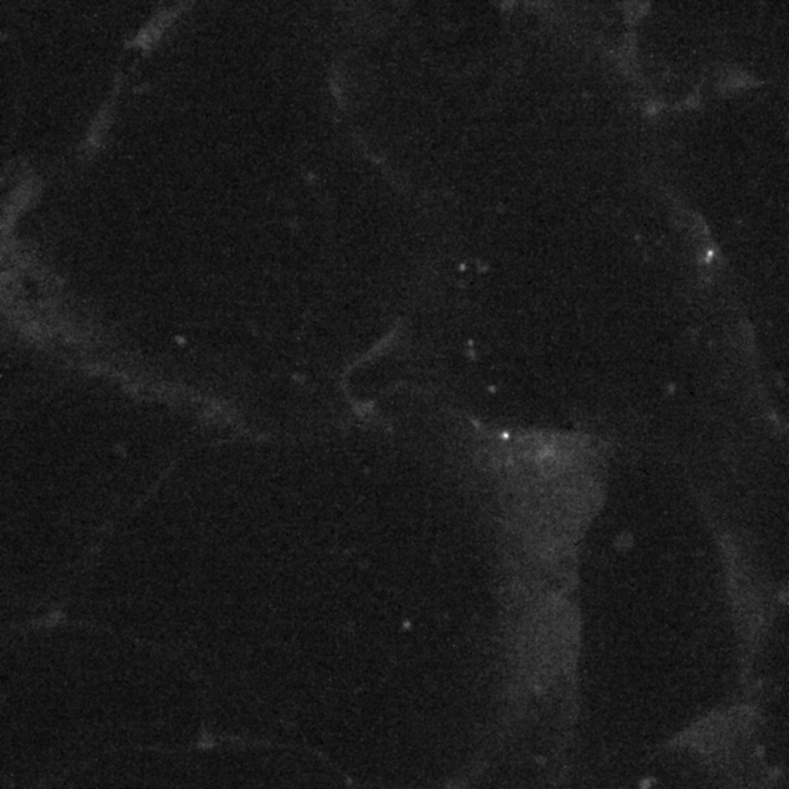

Supplement: Supplementary file 15 — Source Data for Figure 4 [file EMBJ-42-e111252-s016.zip › Figure 4/4G/Composite Vim KO t=5.tif]

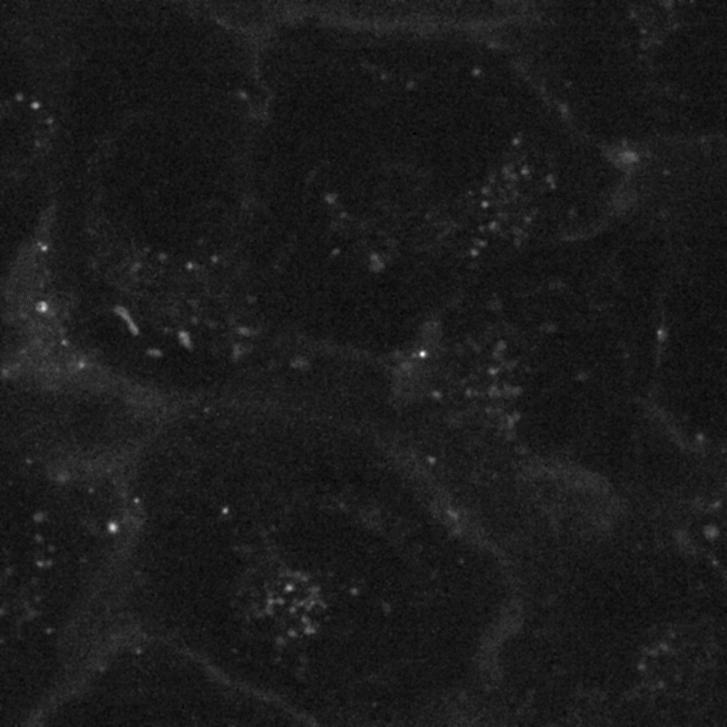

Supplement: Supplementary file 15 — Source Data for Figure 4 [file EMBJ-42-e111252-s016.zip › Figure 4/4G/Composite WT t=120.tif]

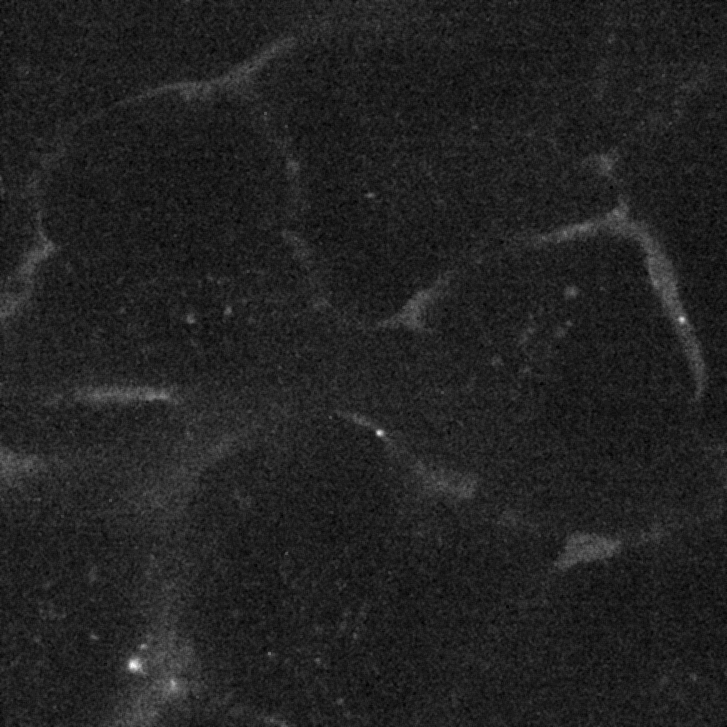

Supplement: Supplementary file 15 — Source Data for Figure 4 [file EMBJ-42-e111252-s016.zip › Figure 4/4G/Composite WT t=5.tif]

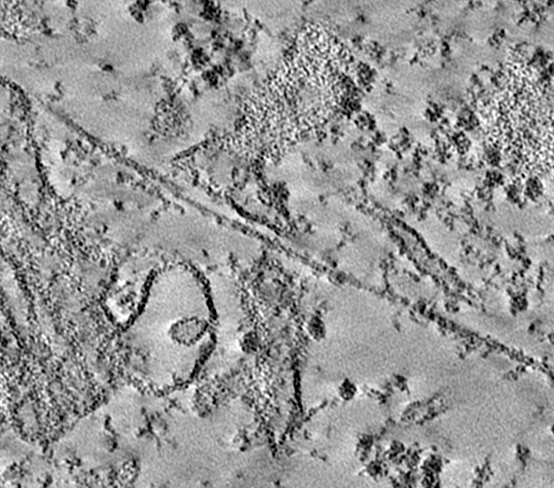

Supplement: Supplementary file 16 — Source Data for Figure 5 [file EMBJ-42-e111252-s005.zip › Figure 5/5A/micrograph zoom.tif]

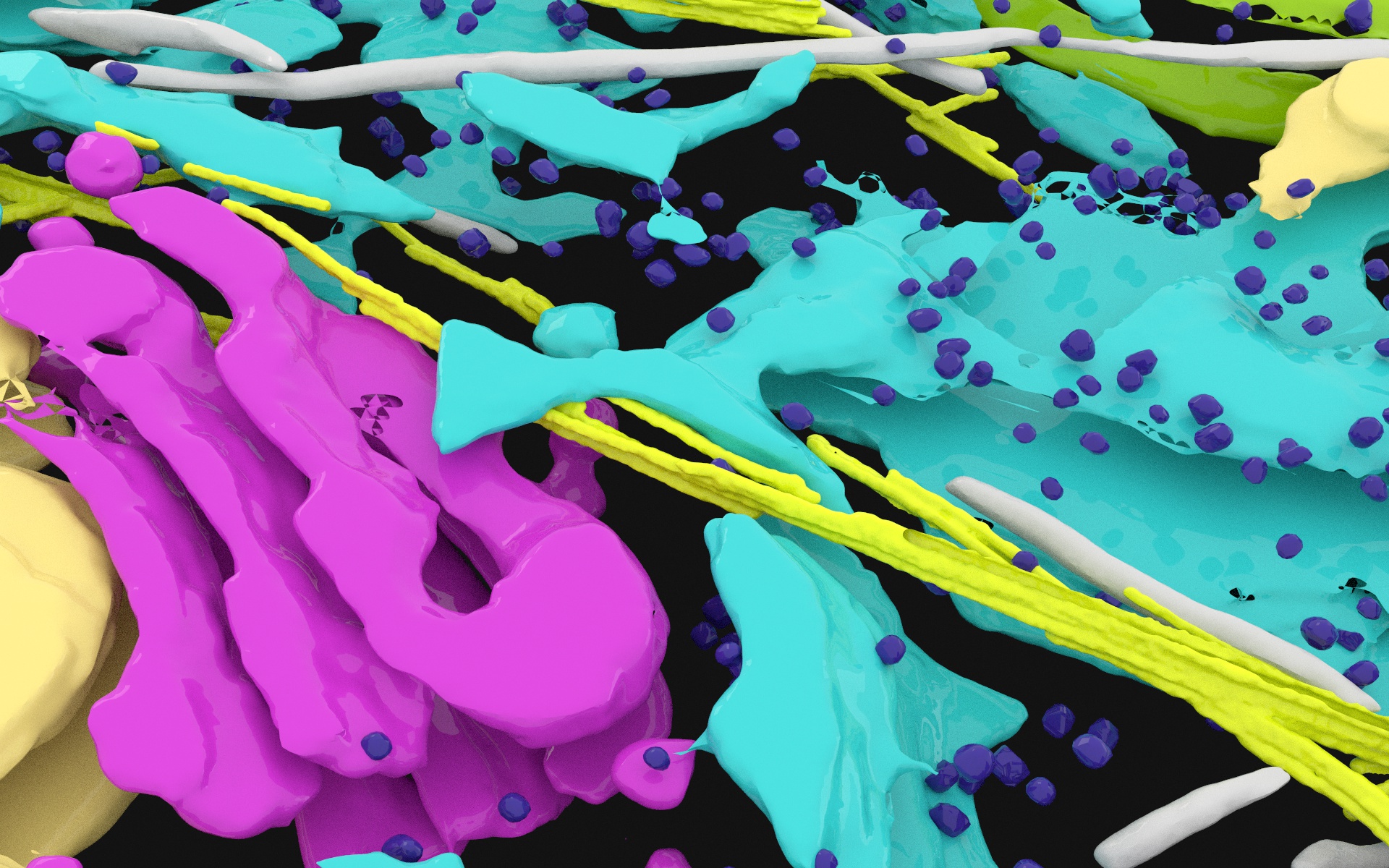

Supplement: Supplementary file 16 — Source Data for Figure 5 [file EMBJ-42-e111252-s005.zip › Figure 5/5A/Tomogram zoom.jpg]

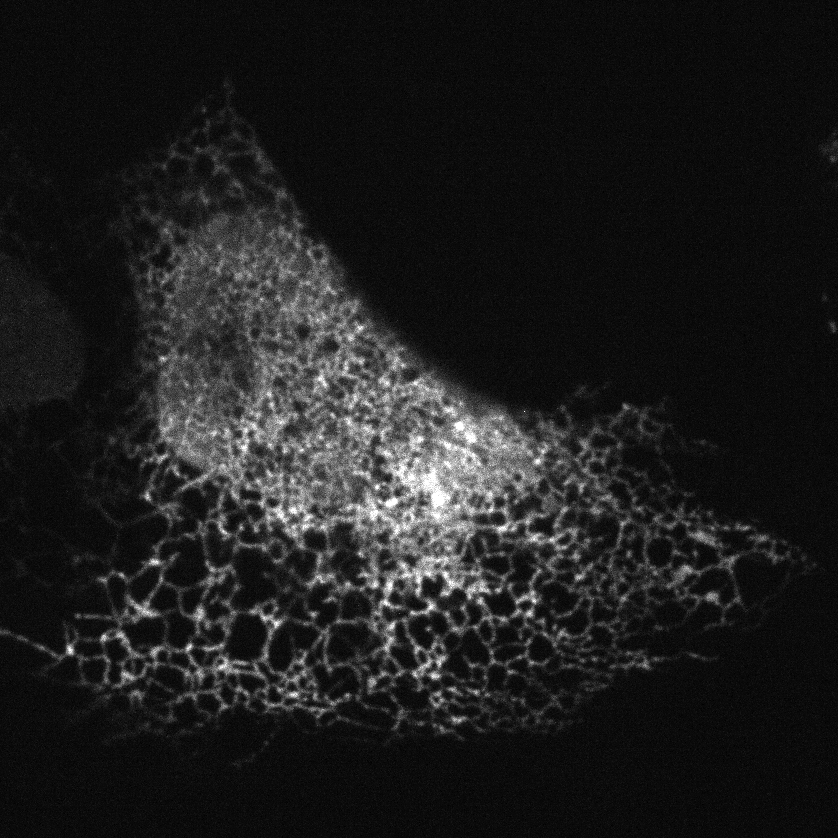

Supplement: Supplementary file 16 — Source Data for Figure 5 [file EMBJ-42-e111252-s005.zip › Figure 5/5B/Composite GFP-Vimentin Cherry-KDEL.tif]

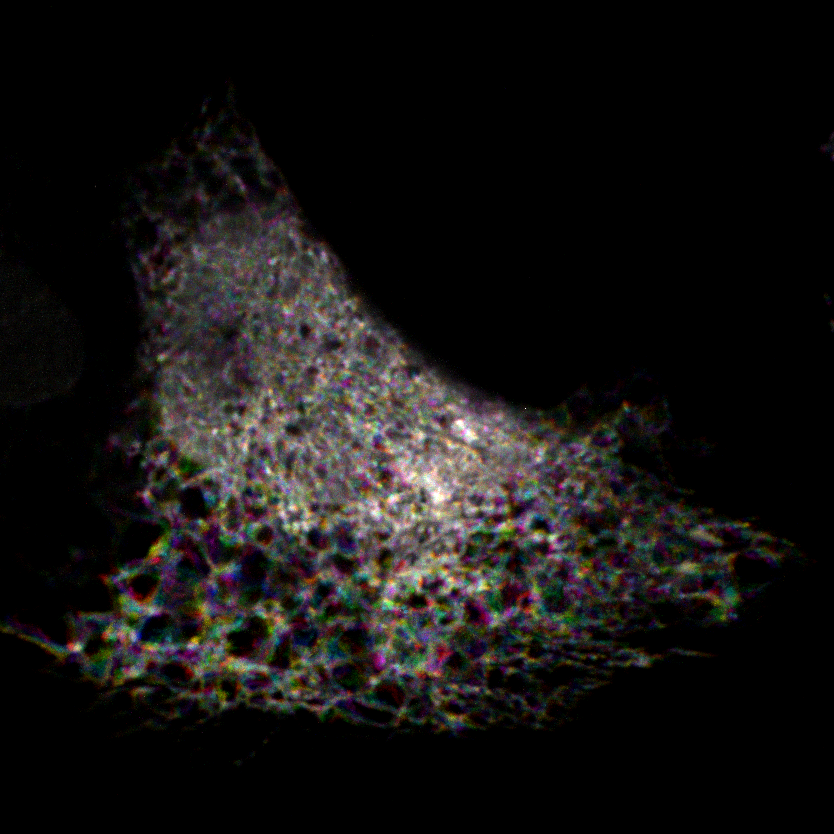

Supplement: Supplementary file 16 — Source Data for Figure 5 [file EMBJ-42-e111252-s005.zip › Figure 5/5B/TCC Cherry-KDEL.tif]

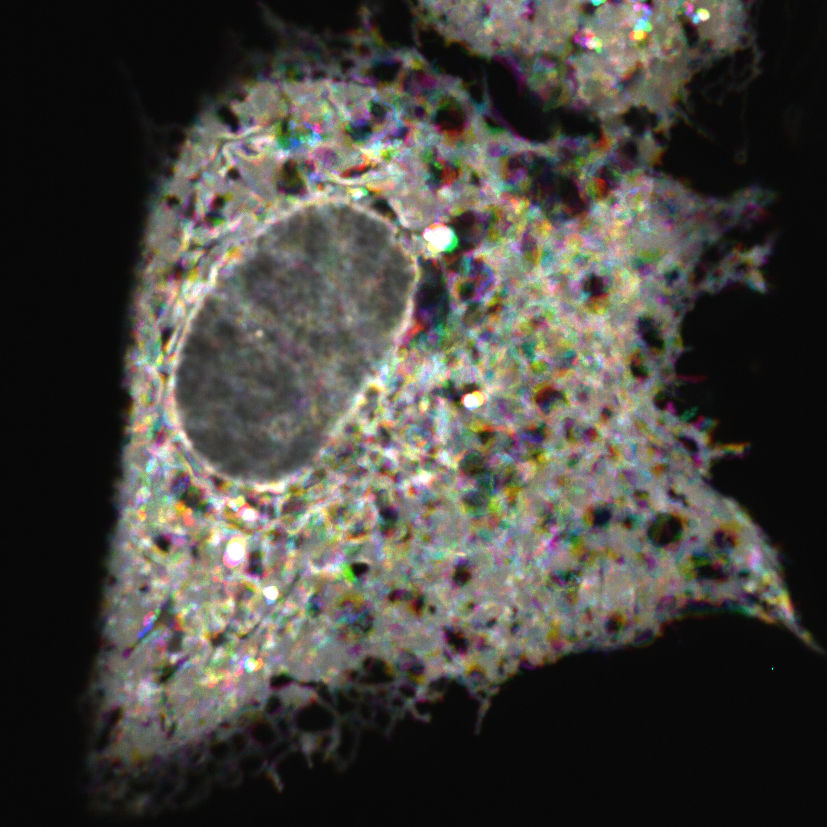

Supplement: Supplementary file 16 — Source Data for Figure 5 [file EMBJ-42-e111252-s005.zip › Figure 5/5D/Vim KO TCC.tif]

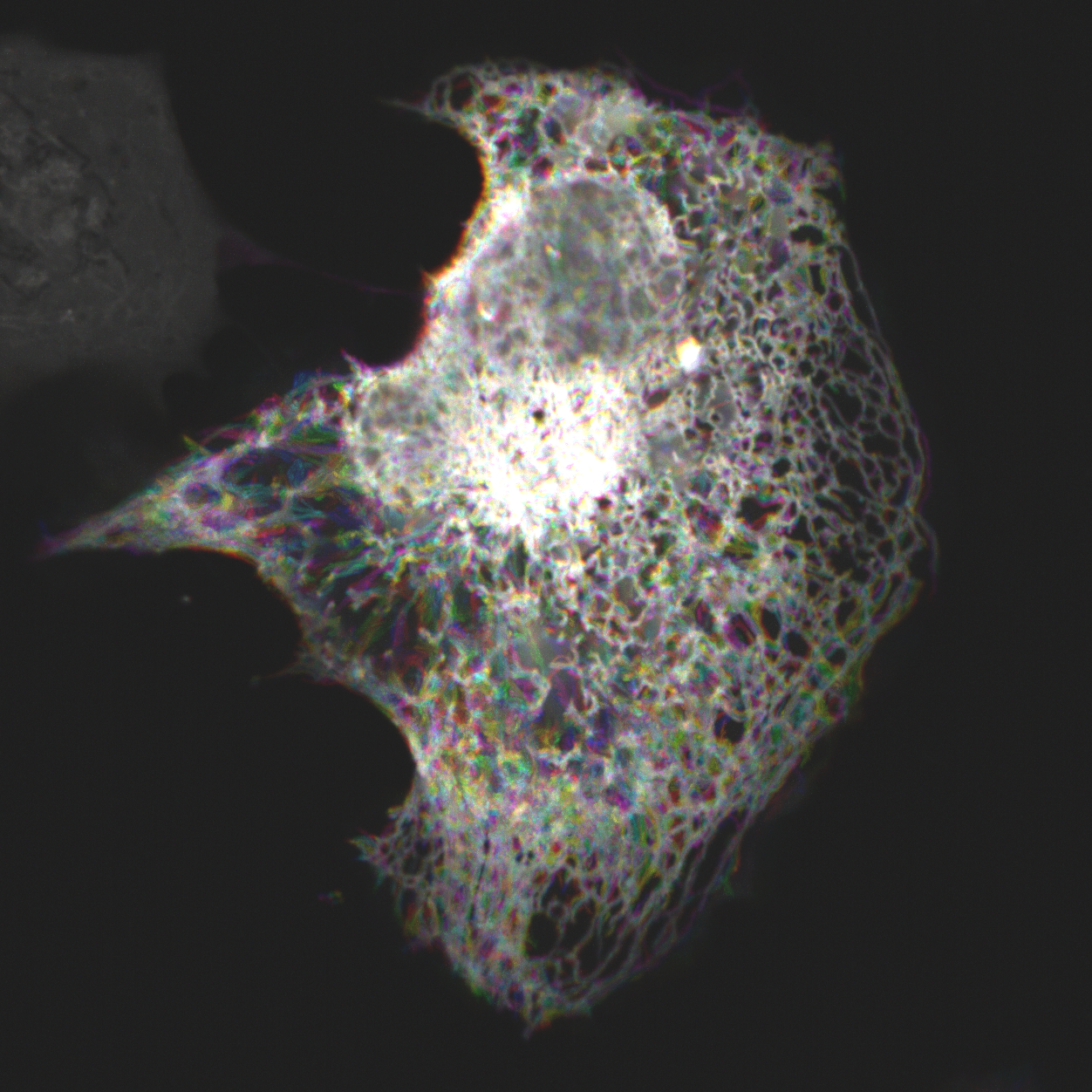

Supplement: Supplementary file 16 — Source Data for Figure 5 [file EMBJ-42-e111252-s005.zip › Figure 5/5D/WT TCC.tif]

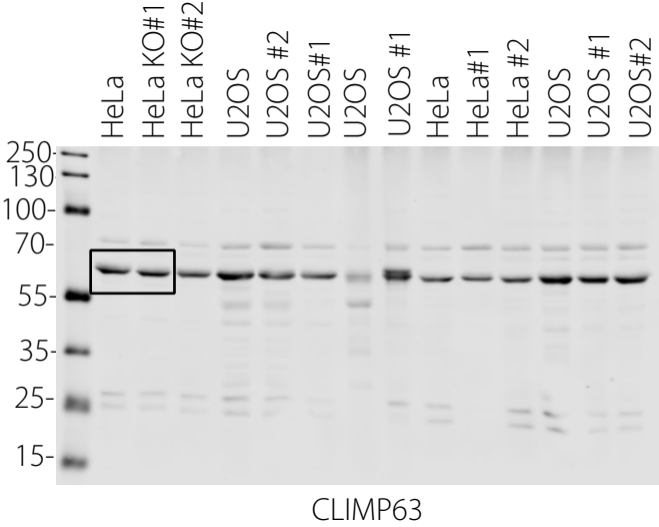

Supplement: Supplementary file 16 — Source Data for Figure 5 [file EMBJ-42-e111252-s005.zip › Figure 5/5I/CLIMP63.pdf]

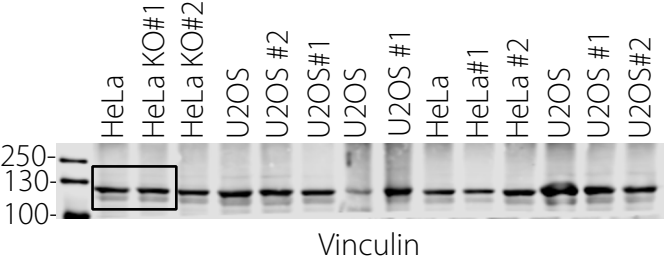

Supplement: Supplementary file 16 — Source Data for Figure 5 [file EMBJ-42-e111252-s005.zip › Figure 5/5I/Vinculin.pdf]

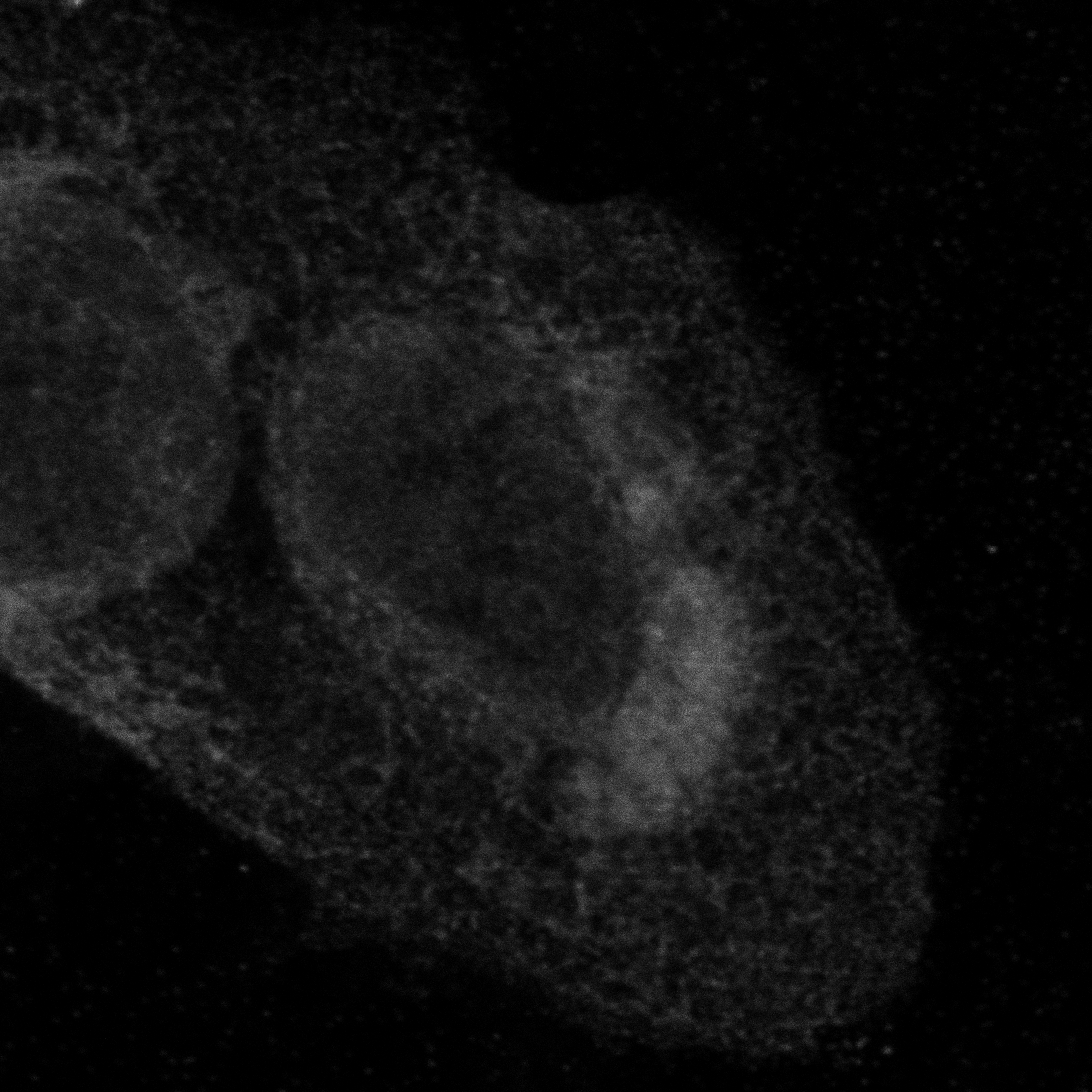

Supplement: Supplementary file 17 — Source Data for Figure 6 [file EMBJ-42-e111252-s011.zip › Figure 6/6D/control Tunicamycin.tif]

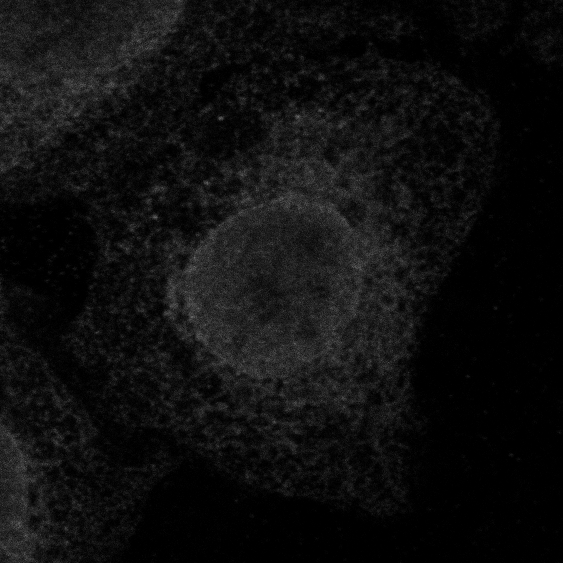

Supplement: Supplementary file 17 — Source Data for Figure 6 [file EMBJ-42-e111252-s011.zip › Figure 6/6D/control.tif]

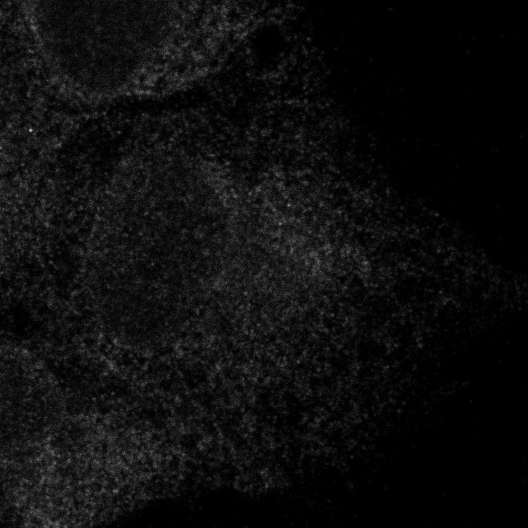

Supplement: Supplementary file 17 — Source Data for Figure 6 [file EMBJ-42-e111252-s011.zip › Figure 6/6D/si2J1 tunicamycin.tif]

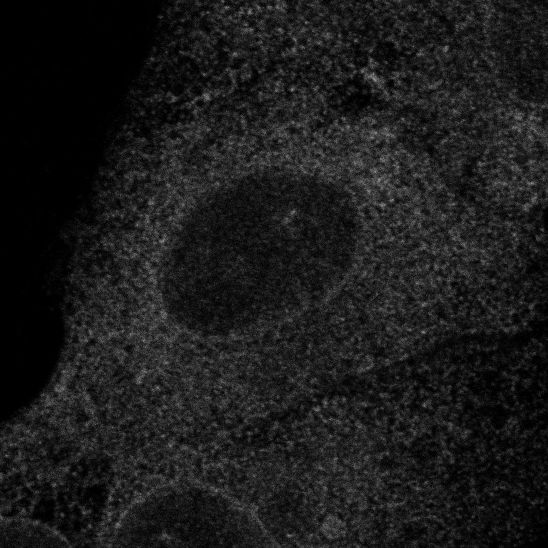

Supplement: Supplementary file 17 — Source Data for Figure 6 [file EMBJ-42-e111252-s011.zip › Figure 6/6D/siHERP1 Tunicamycin.tif]

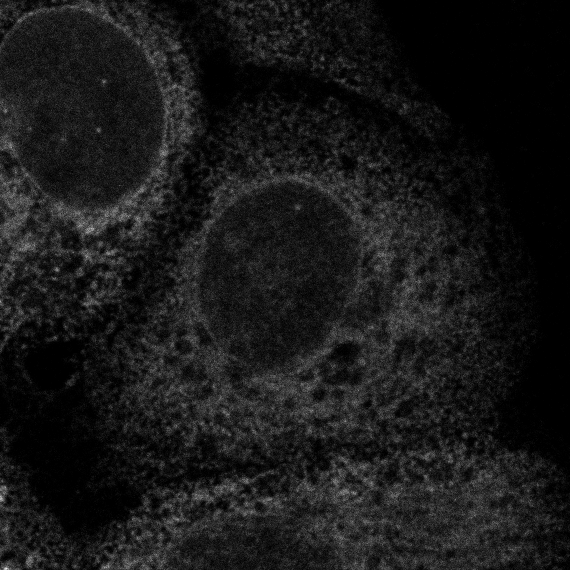

Supplement: Supplementary file 17 — Source Data for Figure 6 [file EMBJ-42-e111252-s011.zip › Figure 6/6D/siHERP1 untreated.tif]

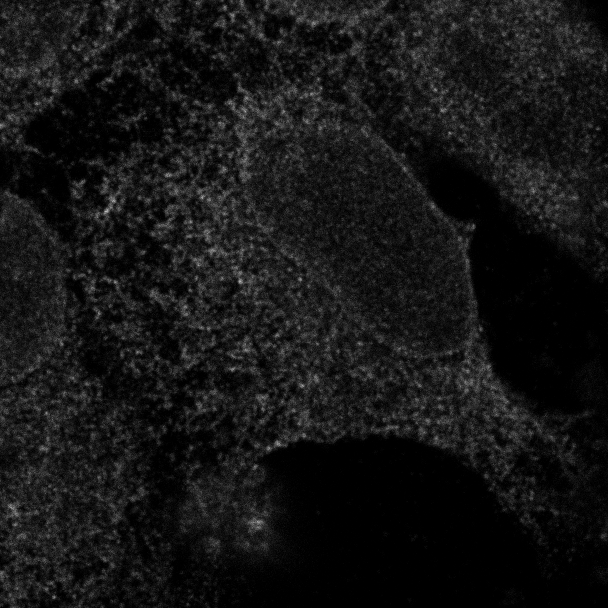

Supplement: Supplementary file 17 — Source Data for Figure 6 [file EMBJ-42-e111252-s011.zip › Figure 6/6D/siRNF26-1 Tunicamycin.tif]

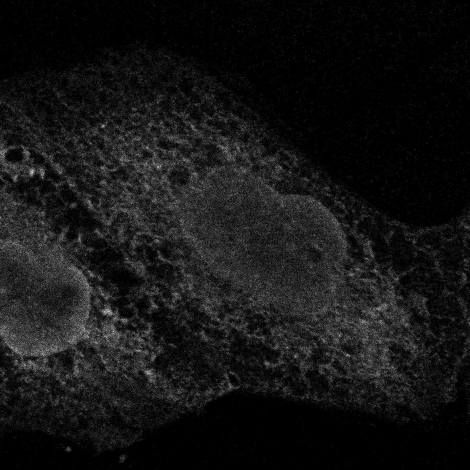

Supplement: Supplementary file 17 — Source Data for Figure 6 [file EMBJ-42-e111252-s011.zip › Figure 6/6D/siRNF26-1 untreated.tif]

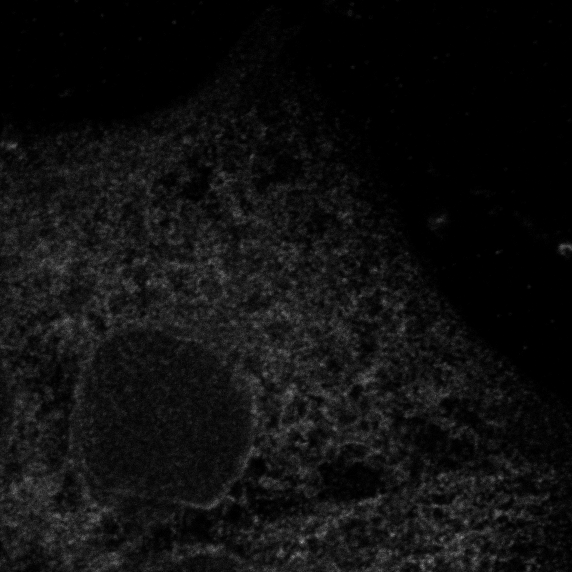

Supplement: Supplementary file 17 — Source Data for Figure 6 [file EMBJ-42-e111252-s011.zip › Figure 6/6D/VIM KO Tunicamycin.tif]

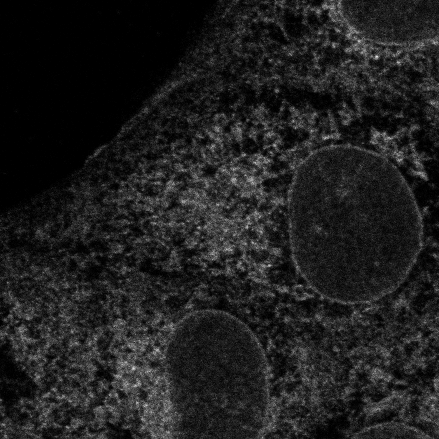

Supplement: Supplementary file 17 — Source Data for Figure 6 [file EMBJ-42-e111252-s011.zip › Figure 6/6D/VIM KO.tif]

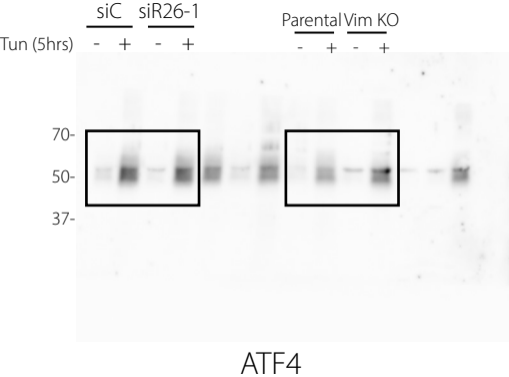

Supplement: Supplementary file 17 — Source Data for Figure 6 [file EMBJ-42-e111252-s011.zip › Figure 6/6F/ATF4.pdf]

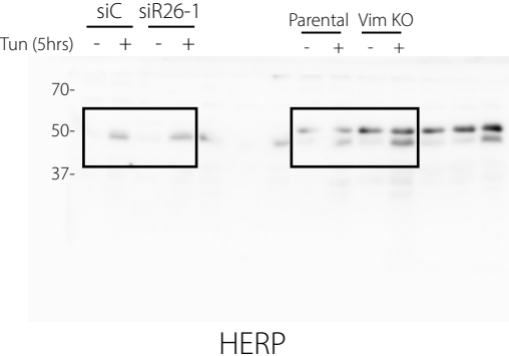

Supplement: Supplementary file 17 — Source Data for Figure 6 [file EMBJ-42-e111252-s011.zip › Figure 6/6F/HERP.pdf]

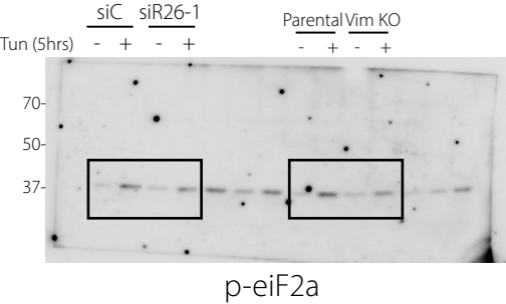

Supplement: Supplementary file 17 — Source Data for Figure 6 [file EMBJ-42-e111252-s011.zip › Figure 6/6F/p-eif2a.pdf]

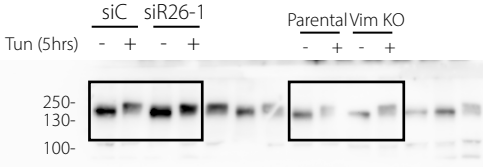

PERK

Supplement: Supplementary file 17 — Source Data for Figure 6 [file EMBJ-42-e111252-s011.zip › Figure 6/6F/PERK.pdf]

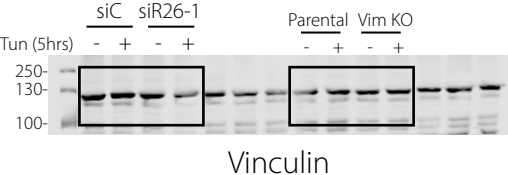

Supplement: Supplementary file 17 — Source Data for Figure 6 [file EMBJ-42-e111252-s011.zip › Figure 6/6F/Vinculin.pdf]

HA-HERP1

EV RNF26 I382R  $\Delta$ RING

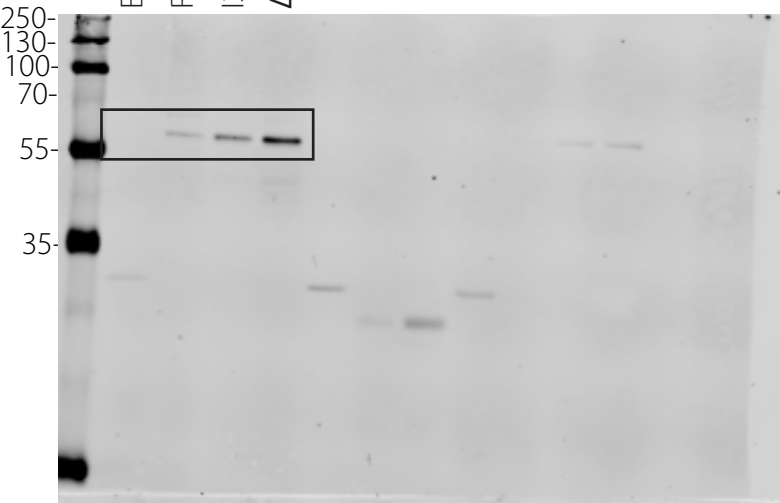

HA (IP)

Supplement: Supplementary file 18 — Source Data for Figure 7 [file EMBJ-42-e111252-s019.zip › Figure 7/7C/HERP1 IP HA.pdf]

HA-HERP1

EV  
RNF26  
I382R  
 $\Delta$ RING

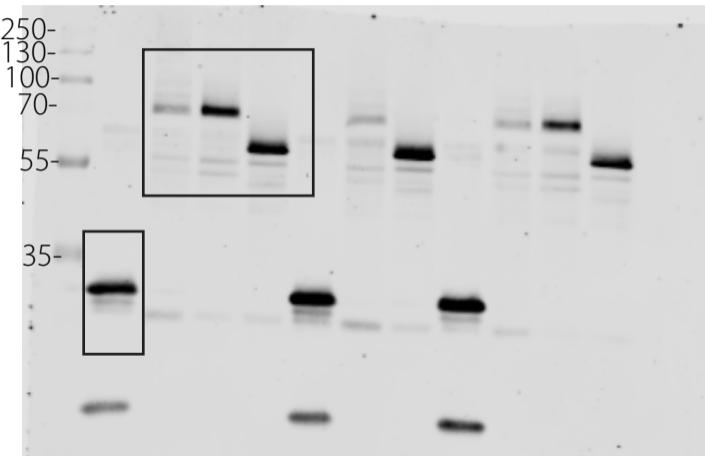

RFP (IP)

Supplement: Supplementary file 18 — Source Data for Figure 7 [file EMBJ-42-e111252-s019.zip › Figure 7/7C/HERP1 IP RFP.pdf]

HA-HERP1

EV  
RNF26  
I382R  
 $\Delta$ RING

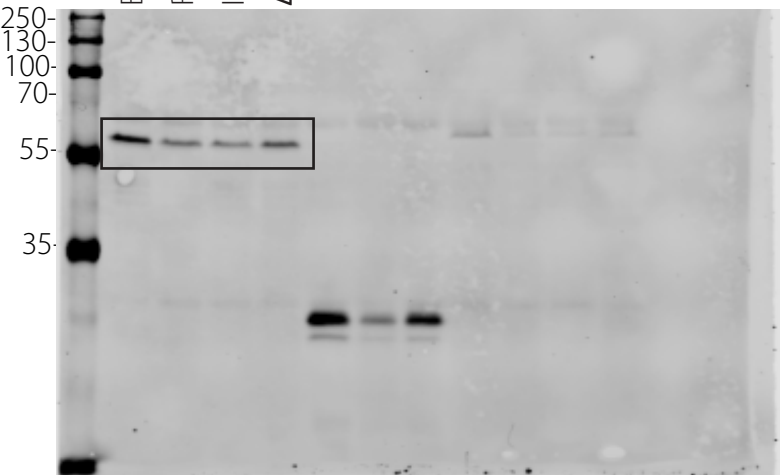

HA (WCL)

Supplement: Supplementary file 18 — Source Data for Figure 7 [file EMBJ-42-e111252-s019.zip › Figure 7/7C/HERP1 WCL HA.pdf]

HA-HRD1

EV  
RNF26  
I382R  
 $\Delta$ RING

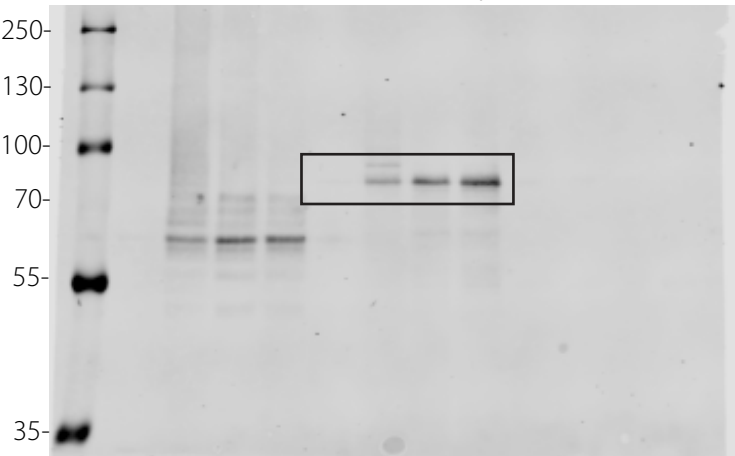

HA (IP)

Supplement: Supplementary file 18 — Source Data for Figure 7 [file EMBJ-42-e111252-s019.zip › Figure 7/7C/HRD1 IP HA.pdf]

HA-HRD1

EV  
RNF26  
I382R  
 $\Delta$ RING

250-  
130-  
100-  
70-  
55-  
35-

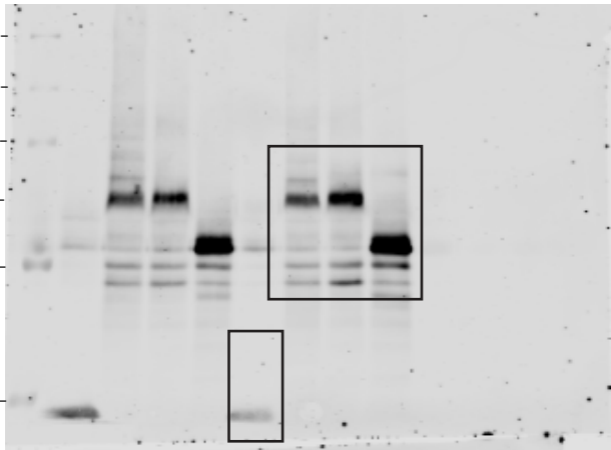

RFP (IP)

Supplement: Supplementary file 18 — Source Data for Figure 7 [file EMBJ-42-e111252-s019.zip › Figure 7/7C/HRD1 IP RFP.pdf]

HA-HRD1

EV

RNF26

I382R

$\Delta$ RING

250

130

100

70

55

35

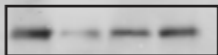

HA (WCL)

Supplement: Supplementary file 18 — Source Data for Figure 7 [file EMBJ-42-e111252-s019.zip › Figure 7/7C/HRD1 WCL HA.pdf]

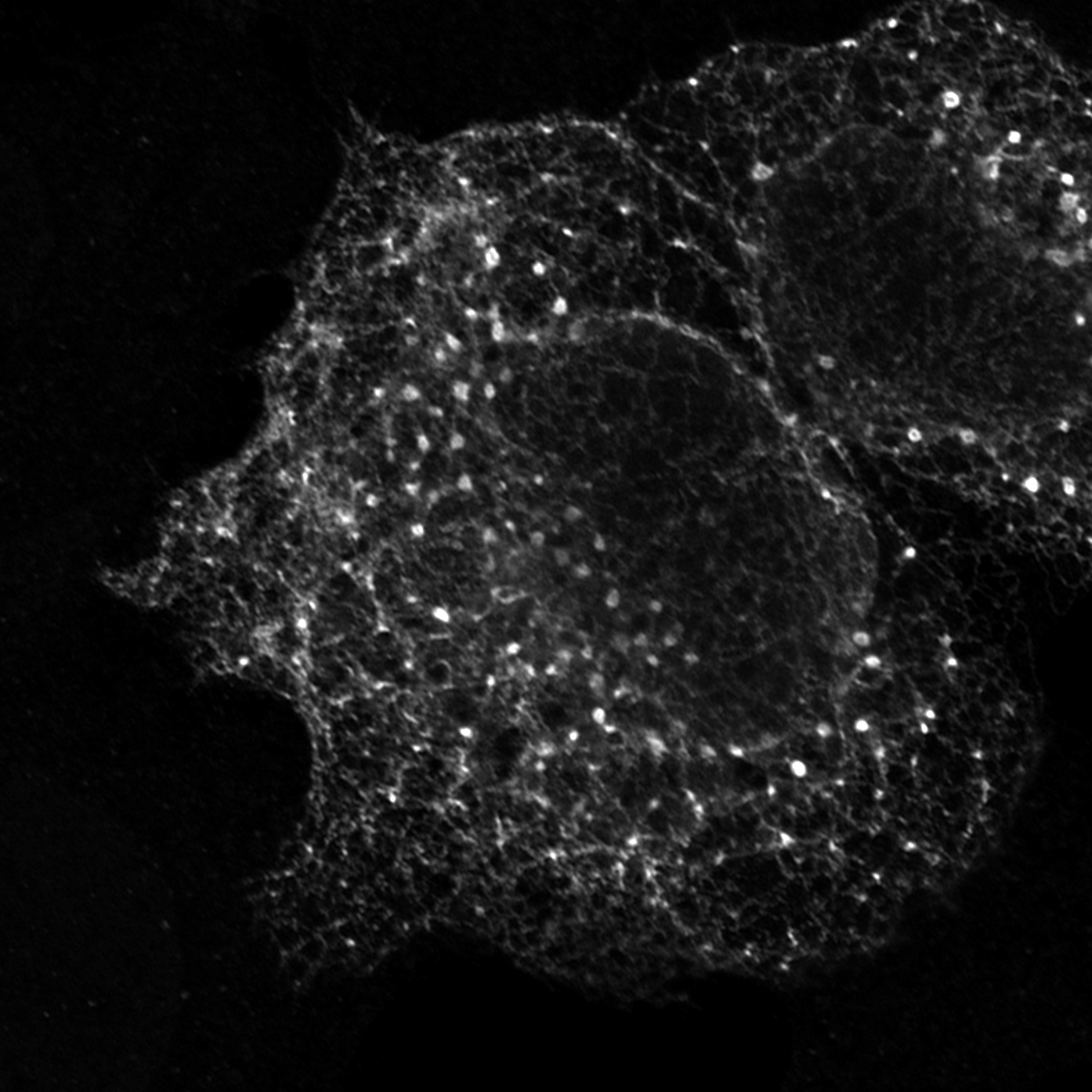

Supplement: Supplementary file 18 — Source Data for Figure 7 [file EMBJ-42-e111252-s019.zip › Figure 7/7D/composite Vimentin HERP1.tif]

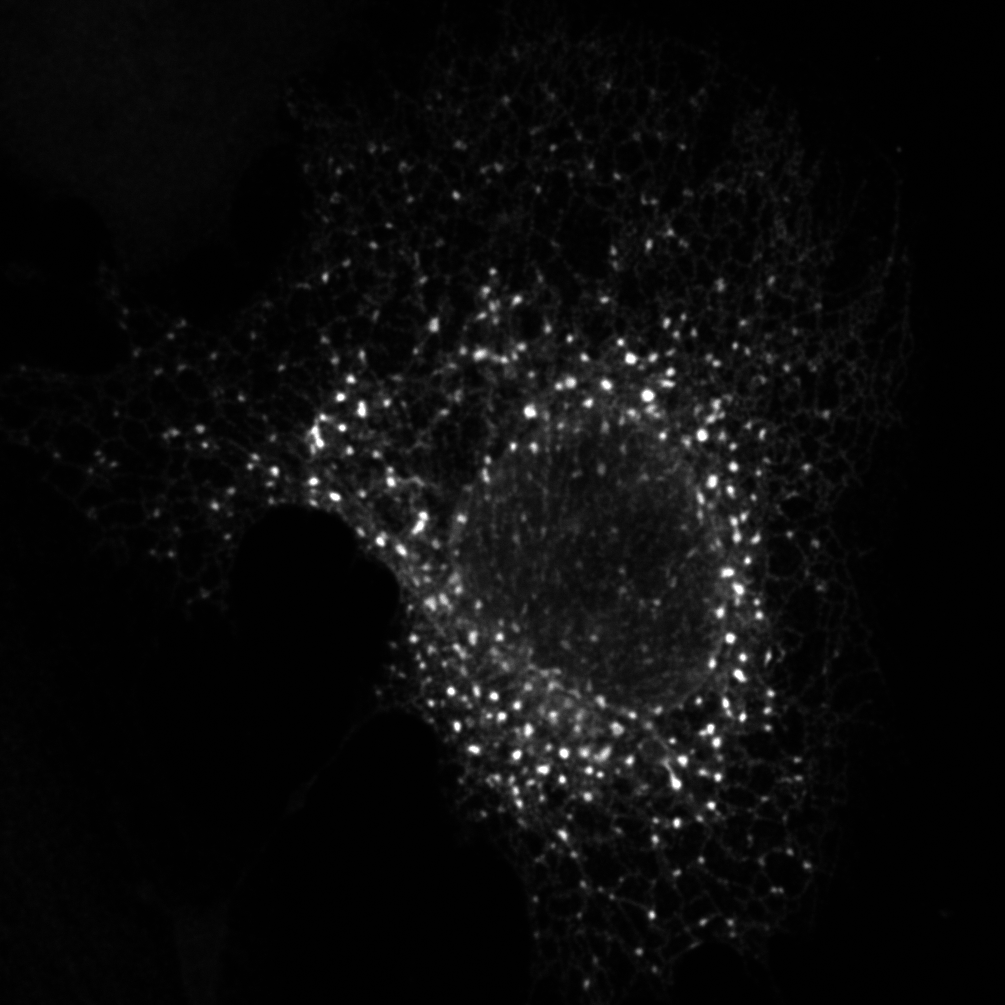

Supplement: Supplementary file 18 — Source Data for Figure 7 [file EMBJ-42-e111252-s019.zip › Figure 7/7F/Parental HA-HERP1.tif]

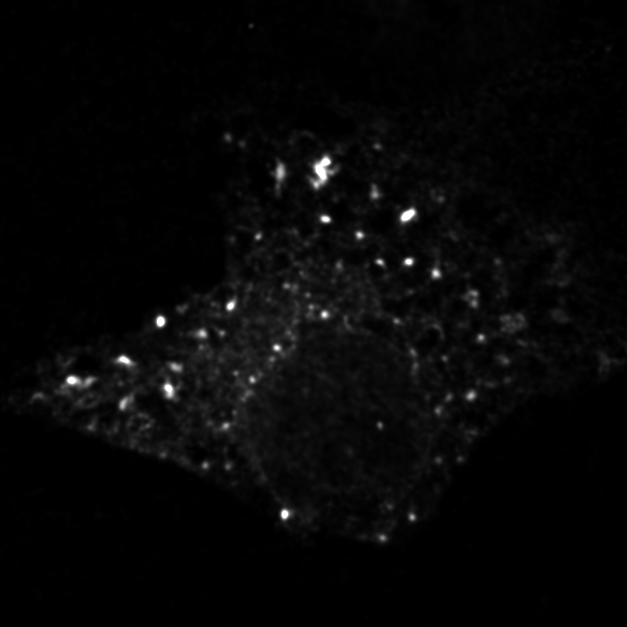

Supplement: Supplementary file 18 — Source Data for Figure 7 [file EMBJ-42-e111252-s019.zip › Figure 7/7F/R26 KO HA-HERP1.tif]

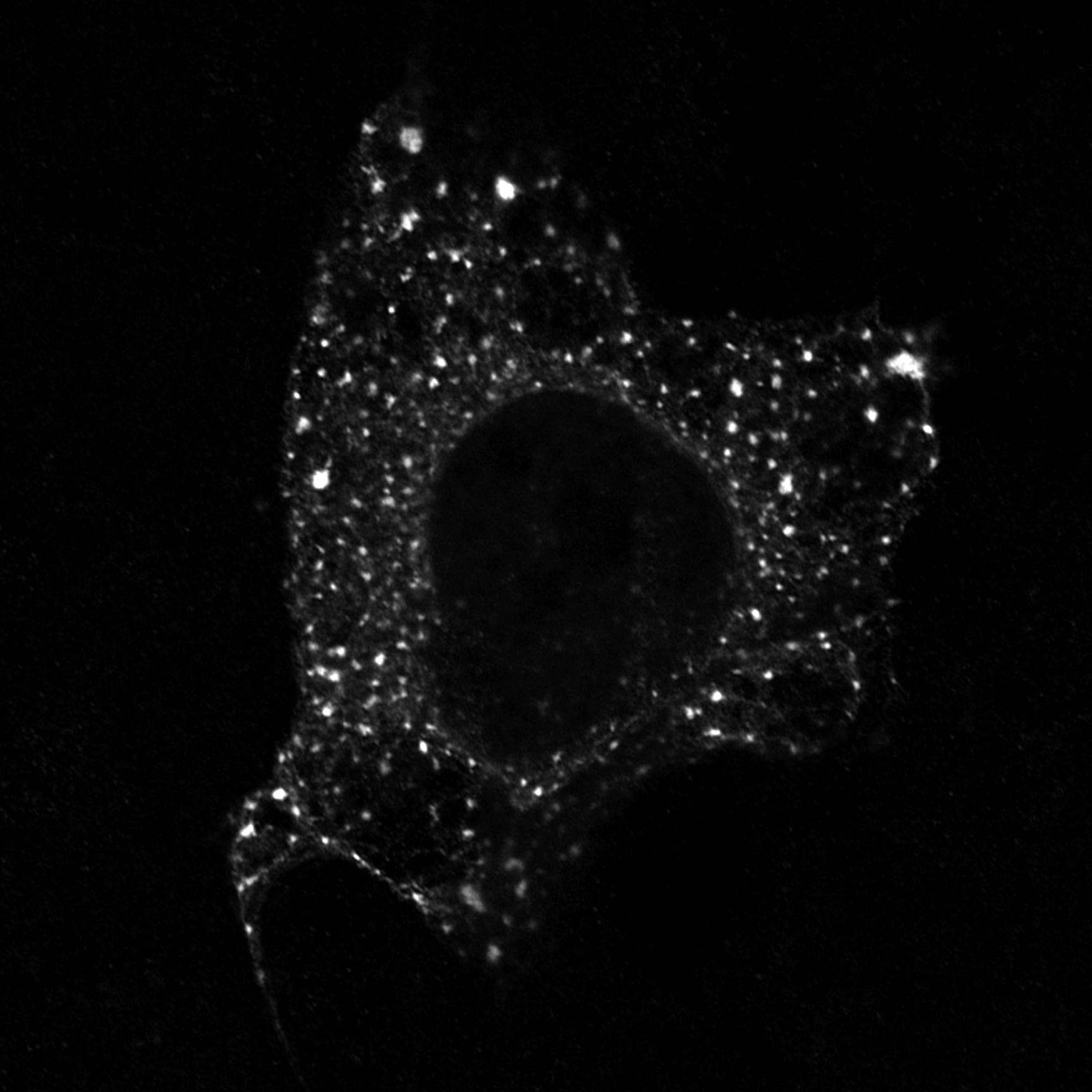

Supplement: Supplementary file 18 — Source Data for Figure 7 [file EMBJ-42-e111252-s019.zip › Figure 7/7F/Vim KO HA-HERP1.tif]

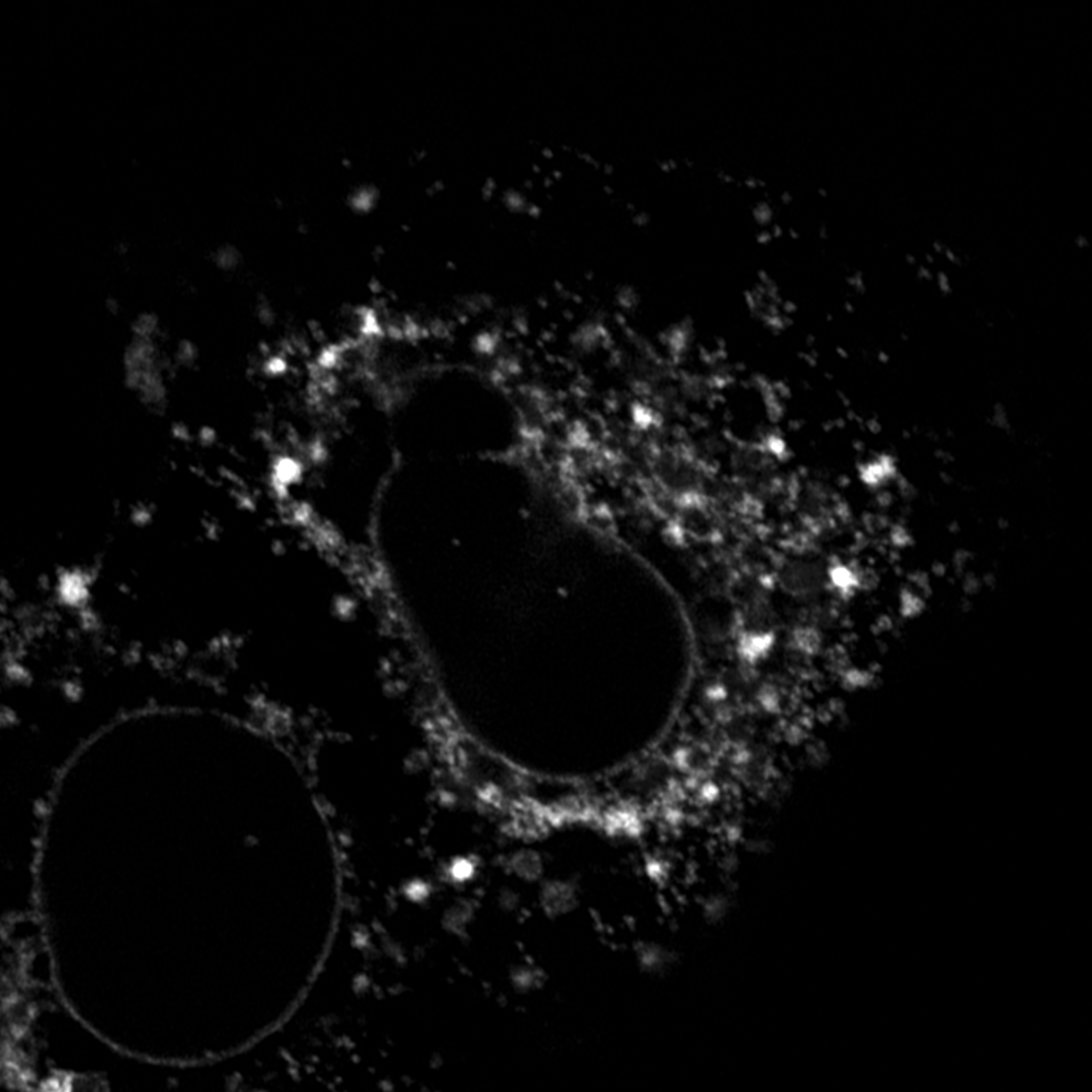

Supplement: Supplementary file 19 — Source Data for Figure 8 [file EMBJ-42-e111252-s004.zip › Figure 8/8A/Composite Sec62-HALO-GFP HERP1.tif]

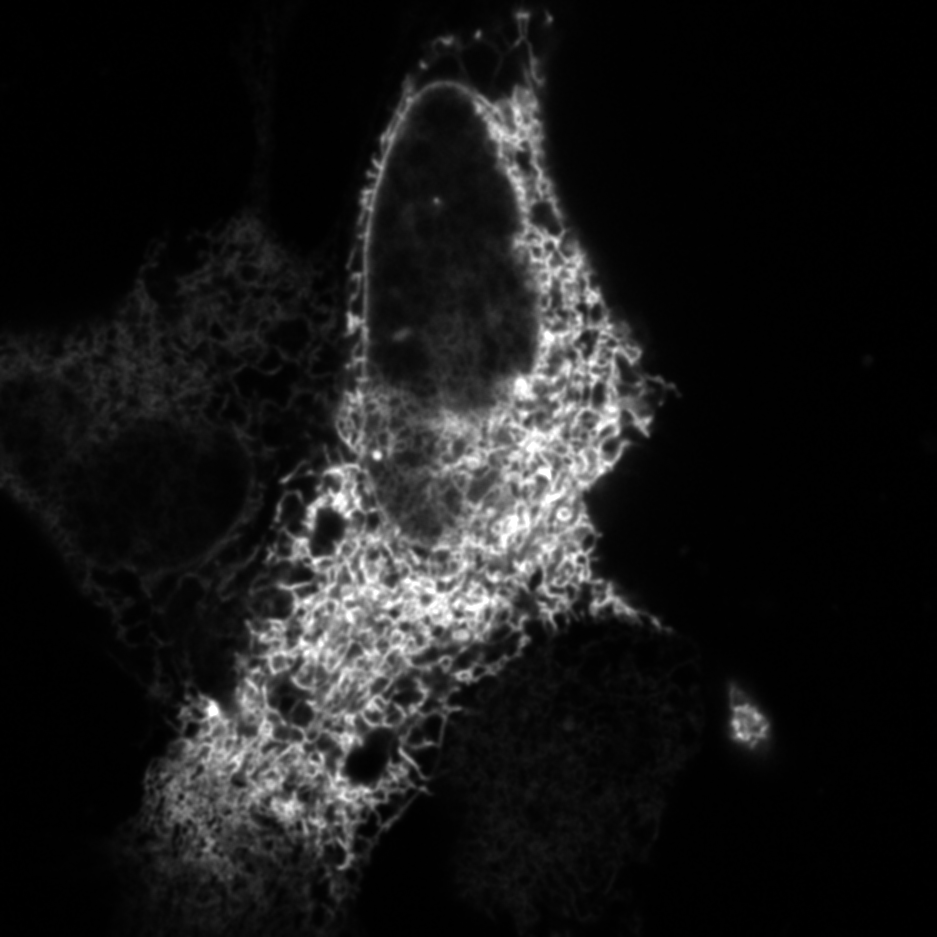

Supplement: Supplementary file 19 — Source Data for Figure 8 [file EMBJ-42-e111252-s004.zip › Figure 8/8B/Composite Sec62-HALO-GFP LysoTracker.tif]

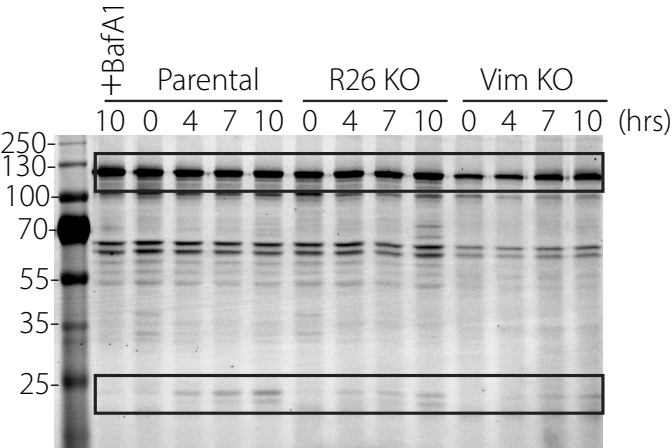

Rhodamine scan

Supplement: Supplementary file 19 — Source Data for Figure 8 [file EMBJ-42-e111252-s004.zip › Figure 8/8C/Rhodamine sec62-HALO-GFP.pdf]

+BafA1  
Parental R26 KO Vim KO  
10 0 4 7 10 0 4 7 10 0 4 7 10 (hrs)

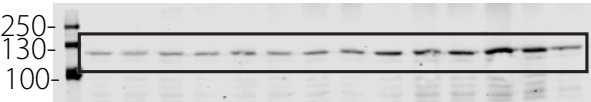

WB: Vinculin

Supplement: Supplementary file 19 — Source Data for Figure 8 [file EMBJ-42-e111252-s004.zip › Figure 8/8C/WB Vinculin.pdf]

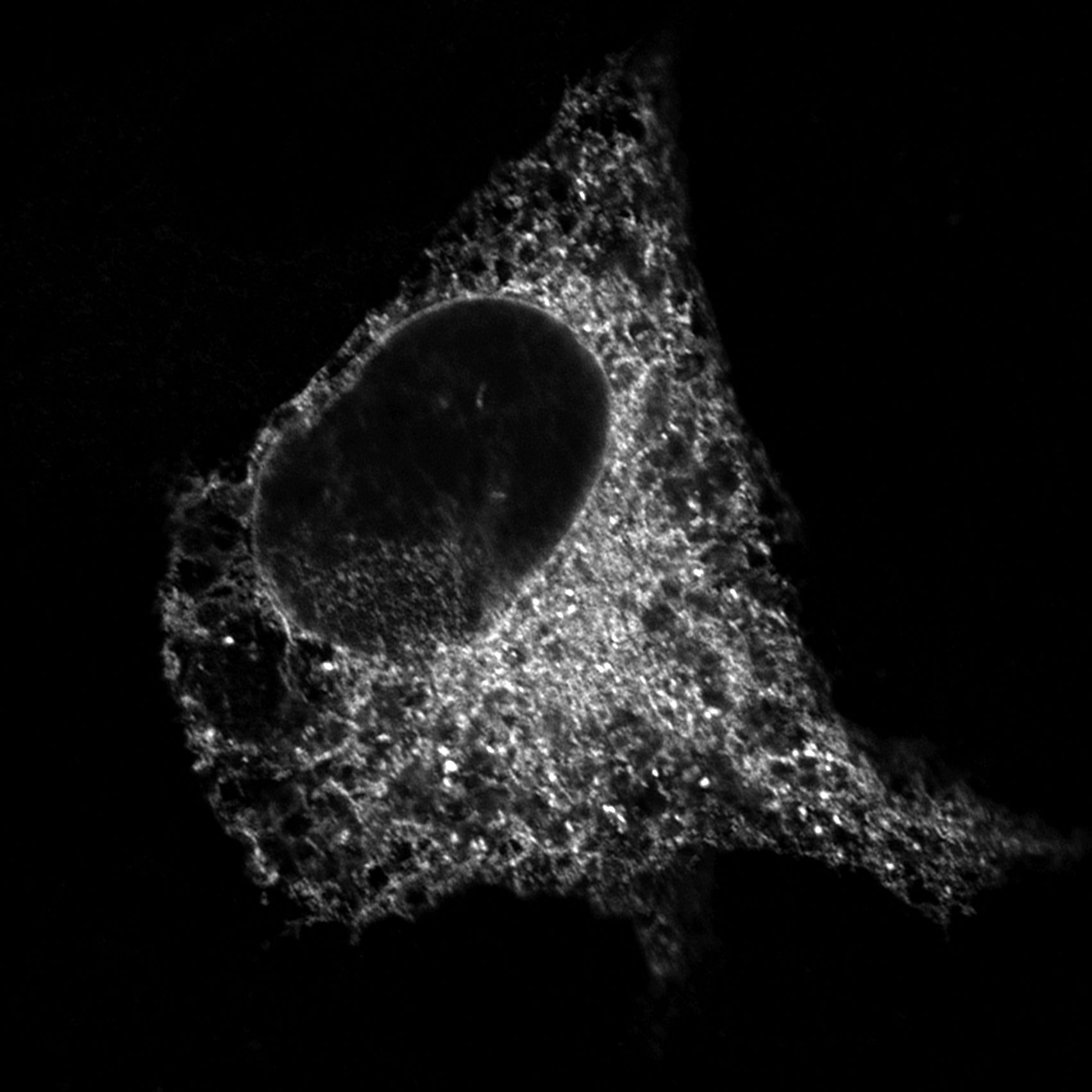

Supplement: Supplementary file 19 — Source Data for Figure 8 [file EMBJ-42-e111252-s004.zip › Figure 8/8E/Composite RNF26 KO Sec62-GFP CD63.tif]

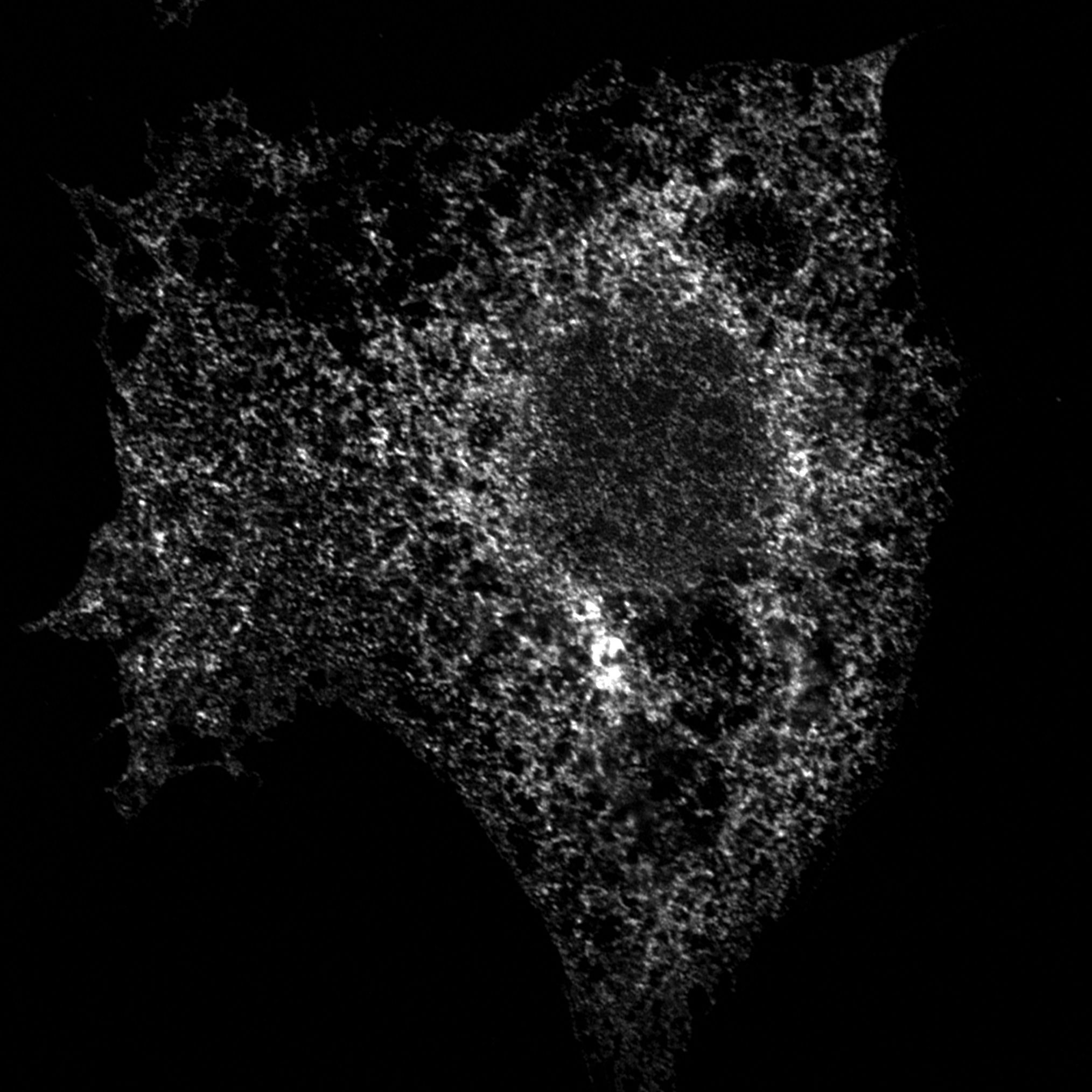

Supplement: Supplementary file 19 — Source Data for Figure 8 [file EMBJ-42-e111252-s004.zip › Figure 8/8E/Composite Vim KO Sec62-GFP CD63.tif]

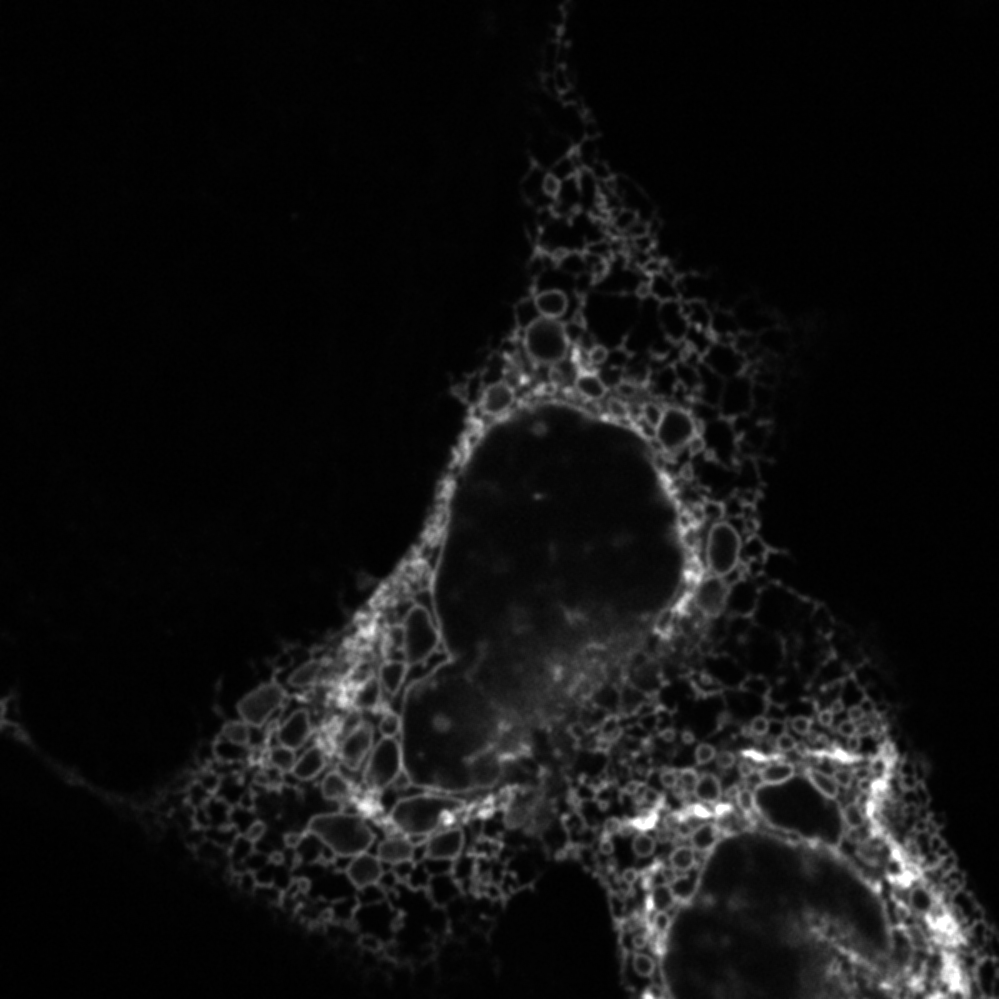

Supplement: Supplementary file 19 — Source Data for Figure 8 [file EMBJ-42-e111252-s004.zip › Figure 8/8G/composite RNF26 I382R Sec62-HALO-GFP.tif]

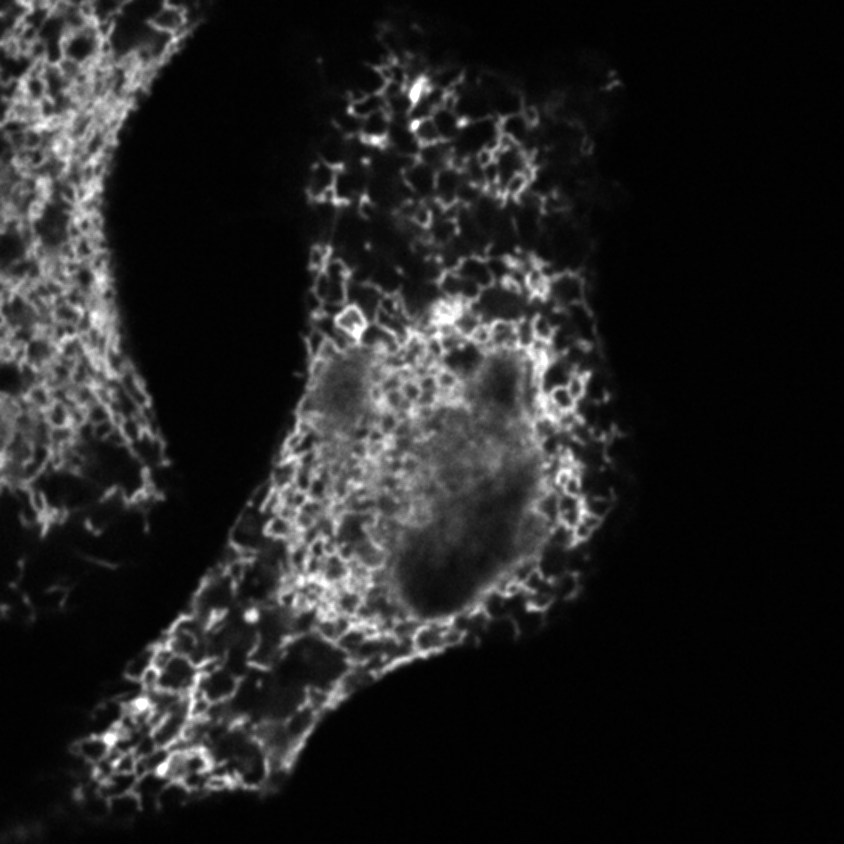

Supplement: Supplementary file 19 — Source Data for Figure 8 [file EMBJ-42-e111252-s004.zip › Figure 8/8G/composite RNF26 WT Sec62-HALO-GFP.tif]
