# Supplementary material for: RNF26 binds perinuclear vimentin filaments to integrate ER and endolysosomal responses to proteotoxic stress
Source: EMBO J. 2023 Jul 31;42(18):e111252. doi: 10.15252/embj.2022111252 (PMC10505911; doi:10.15252/embj.2022111252)
Supplement: Supplementary file 20 [file EMBJ-42-e111252-s009.pdf]

THE EMBO JOURNAL

RNF26 binds perinuclear Vimentin filaments to integrate ER and endolysosomal responses to proteotoxic stress

Tom Cremer, Lenard Voortman, Erik Bos, Marlieke Jongsma, Laurens Ter Haar, Jimmy Akkermans, Cami Talavera Ormeno, Ruud Wijdeven, Jelle Vries, Robbert Kim, George Janssen, Peter van Veelen, Roman Koning, Jacques Neefjes, and Ilana Berlin
DOI: 10.15252/embj.2022111252

Corresponding author(s): Jacques Neefjes (j.j.c.neefjes@lumc.nl) , Ilana Berlin (I.Berlin@lumc.nl)

Review Timeline:

Submission Date:	23rd Mar 22
Editorial Decision:	11th May 22
Appeal Received:	7th Jun 22
Editorial Decision:	28th Apr 23
Revision Received:	3rd Jun 23
Editorial Decision:	21st Jun 23
Revision Received:	28th Jun 23
Accepted:	3rd Jul 23

Editor: William Teale

Transaction Report:

(Note: With the exception of the correction of typographical or spelling errors that could be a source of ambiguity, letters and reports are not edited. Depending on transfer agreements, referee reports obtained elsewhere may or may not be included in this compilation. Referee reports are anonymous unless the Referee chooses to sign their reports.)

1st Editorial Decision 11th May 2022

Dear Jacques,

Thank you for submitting your study, "Vimentin intermediate filaments organize organellar architecture in response to ER stress", for consideration by the EMBO Journal. It has now been seen by three referees whose comments are shown below. The referees' feedback was constructive, and all appreciated your work; however, two gaps were highlighted which, in my opinion, preclude publication in EMBO Journal.

Firstly, the work would need to be framed in a wider physiological context. For example, what are the triggers that regulate Vimentin-dependent ER and endolysosome positioning? In which contexts does a permanent anchor between the ER and Vimentin alter endolysosome function? Secondly, I agree with referee 2 that the interaction data between RNF26 and Vimentin need to be strengthened.

Although EMBO Journal would need these aspects to be addressed in a re-submitted manuscript (which would be returned to the same set of reviewers for appraisal), I have discussed your work and the accompanying referee reports with my colleagues at EMBO Reports. They are, I am happy to say, potentially interested in publishing your work. To proceed in this direction, all technical concerns raised by the referees need to be addressed, the data on the interaction between RNF126 and Vimentin need to be strengthened and key experiments should be repeated in U2OS cells. It would not be essential to provide further data on the physiological significance for EMBO Reports (referee 2, point 6) but they mention that if you have data on whether or not the stress resistance/survival is altered, it would further strengthen the study. My colleague Martina Rembold (martina.rembold@embo.org) at EMBO Reports would be happy to discuss this matter further and answer any questions you may have.

I am sorry we cannot be more positive on this occasion. I nevertheless hope that (whichever direction this work takes towards publication) you will find our referees' comments helpful.

Yours sincerely,

William

William Teale, PhD
Editor
The EMBO Journal
w.teale@embojournal.org

Referee #1:

In the current submission, the authors describe a functional link between the intermediate filament (IF) cytoskeleton, specifically the protein vimentin, and an ER localized ubiquitin ligase ring finger protein 26 (RNF26). These proteins work together to control ER morphology and organelle localization/ compartmentalization during cellular homeostasis and ER stress.

In this study the authors show that the IF cytoskeletal protein vimentin can act as perinuclear anchors of the ER and the endo-lysosomal system by binding to RNF26 and that these anchors can be perturbed by knock out of vimentin or the silencing or mutation of the ER localized RNF26. When the link between the two proteins is broken, the ER as well as the other organelles of the endo-lysosomal system lose their perinuclear localization and take on a more evenly dispersed localization throughout the cell. The authors show that vimentin preferentially binds to inactive RNF26, and that this binding holds the RNF26 in the perinuclear region where vimentin is enriched. The enrichment is shown by colocalization studies and quantitation using Manders' coefficients, immunogold labelling, development of an ImageJ plug-in used to show preferential perinuclear localization of the two proteins over peripheral localization, proximity biotinylation using TurboID, vimentin knockout cell lines, siRNA against RNF26, point mutation or truncated forms of RNF26, and treatment of cells with tunicamycin to induce ER stress. The authors go so far as to show that the last 10 C-terminal amino acid residues of RNF26 are required for the binding of RNF26 with vimentin and therefore localization to the perinuclear region. The study has much to like with some busy work suggestions provide below that will not change the outcome of the story but make it more rigorous and consistent. Perhaps the most substantial issue to address is mentioning the paper by Rodriguez and coworkers (J virol 2002) that demonstrated a link between vimentin and the ER/ERGIC. Im not sure this study was even cited ? So the authors need to make the case that their current study makes a meaningful advance beyond this 20 year old story.

Suggestions by Figures:
Fig.1F
- What patten of RNF26- RING is displayed in U2OS cells?

- What is the relationship of RNF26- RING with vimentin filaments?
- Please show the IF data.

Fig.2E
- Show RNF26 and vimentin protein levels in U2OS cells under the control, RNF26 siRNA KD & Vim KO by WB.

Fig.3B & G
- Please be consistent in using the same cell type (U2OS) (HeLa cells data can be put in a suppl. Figure).
- Same as Fig.1D, please express HA-tagged mutant RNF26 in U2OS cells. IP by anti-vimentin & blot with anti-HA.
- When loading a control can actin/tubulin be used? The use of vimentin is confusing.

Fig.4A
- Show WB of parental, Vim KO#1 & RNF26 -2 siRNA KD in U2OS cells. Blot by anti-Vim & anti-RNF26 antibodies.

Fig.4H
- Missing figure legend.

Fig.5 is a key figure in this paper. For ER stress, can blotting with ER stress marker antibodies, such as BIP, p-eIF2a, eIF2a, ATF4, CHO after TM treatment be included? Also, some viability data after TM treatment at different concentrations would be good.

Fig.5D
- Can RNF26 & vimentin protein levels in parental, RNF26 siRNA treated & Vim KO U2OS cells by WB be shown?

Fig.1A & 4D used Cherry-tagged KDEL. But in M & M Constructs, only GFP-KDEL was mentioned.

Minor issues:
1) What is near-super-resolution? Please describe in detail or change to high resolution or super resolution.
2) The immuno-gold image in figure1 is very difficult to see what is going on. The gold looks sharp, but the ultrastructure is uninterpretable.
3) There is quantitation in a couple of figures where the reader is referred to other images in other figures. Please put images of what is being quantitated and the graphs in the same figures.
4) Figure 4C has no legend, and all the rest are shifted down a letter.
5) Materials and methods section needs part on cell lines used.
6) Most of the experiments are done in U2OS cells except the immuno-precipitations and BioID which were done with HeLa. Can these expts be done in the U2OS cells?
7) The ER stress component was not explored very well as only an increase in the perinuclear localization upon Tunicamycin treatment. It would have been nice to show the converse (if possible) when treating cells with ER stress inhibitors.

Referee #2:

This manuscript entitled "Vimentin intermediate filaments organize organellar architecture in response to ER stress" by T. Cremer and collaborators describes the role of Vimentin intermediate filaments in the spatial organization of organelles such as endosomes and the endoplasmic reticulum (ER).
The authors build on their previous findings identifying a key role for the ER-bound ubiquitin ligase RNF26 (Ring finger protein 26) in the localization of endocytic organelles. They first confirmed from their previous work, that Vimentin is a binding partner of RNF26, and showed that this interaction requires the C-terminal tail of RNF26. The authors then showed that Vimentin controls the distribution of RNF26 in the ER network, and consequently the localization of endolysosomes. Finally, the authors link the function of vimentin and RNF26 to the localization of the ER network and to ER stress. The observation of a role of intermediate filament in the distribution of organelles is novel and an interesting. However, there are several major issues with the manuscript that need to be addressed to fully support this idea: in particular, the data showing the interaction between RNF26 and Vimentin are quite weak, and the physiological consequences for the interaction are missing.

Major points:
1. The data showing the interaction between RNF26 and Vimentin are quite weak:
a. The authors refer to Jongsma et al, 2016 (Line 181-182) to claim that Vimentin co-precipitates with the RING domain of RNF26. I could not find data on Vimentin in this article, nor the mass spectrometry data on a repository (see for instance https://thebiogrid.org/206011/publication/an-er-associated-pathway-defines-endosomal-architecture-for-controlled-cargo-transport.html).
b. The interaction assay the authors used is based on biotin-ligation mediated by TurboID: it is sensu stricto a proximity assay rather than an interaction assay. These data should be confirmed by other approaches: if this reviewer understands what has been done in Jongsma et al correctly, GST-pull-down experiments were performed with the RING domain of RNF26. These

experiments should be repeated to show the interaction with Vimentin. Besides, another more cellular approach should be performed (co-immunoprecipitation, FRET etc...).
c. There are no data on the interaction site in Vimentin. The authors should identify the binding surface of RNF26 on Vimentin using deletion mutants, point mutants...
d. The authors should ideally reconstitute the complex in vitro using recombinant vimentin and peptides if they suspect that the interaction is mediated solely by the short C-terminal extremity of RNF26.
2. This reviewer is not convinced by the way data are analyzed in the manuscript. Most of the time (Fig. 2C, 2D, 2H, 2I, 3D, 3E, 4B, 4C, 4F, 4H, 5E and supplementary), for data obtained from image analyses, data from individual cells are compared and used to perform statistical analyses. The authors should use data from independent experiments (means) to perform statistical analyses.
3. Is Vimentin localization affected by RNF26? For instance, the localization of Vimentin seems different Fig. 1F between cells expressing RNF26 WT or RNF26 I382R. This should be quantified.
4. Does vimentin deficiency affect the localization of other organelles (mitochondria, Golgi, lipid droplets...). This should be analyzed.
5. In absence of RNF26 or Vimentin, is the morphology of the ER affected? The density of ER structures, the quantity of ER-resident proteins should be characterized.
6. The functional consequence of the ERQC formation defects in RNF16 and Vimentin-deficient cells needs to be explored; is the cell response to ER stress induction altered (activation of the different ER stress pathways)? are the cells able to cope with ER stress induction or are they more sensitive to cell death? Etc...
7. Line 272-286: "Collectively, these results suggest that RNF26 and Vimentin are required to maintain compartmentalization of membrane composition in the ER.". Is the complex RNF26-vimentin required, or is the enzymatic activity of RNF26 involved? A rescue experiment using the WT and IR mutant RNF26 could answer this question.

Minor points:
1. Fig 1A: the choice of LUT is not the best to look at co-localizations.
2. Line 34-36: The sentence "While the ubiquitin...tail." is odd: it links two unrelated elements: localization on the one hand, and protein-protein interaction on the other.
3. Fig 1C: the authors should describe how they discriminated vimentin filaments in their tomograms.
4. Fig. 1F: the 2 images shown here are apparently not at the same z level, which might affect the appearance of cellular structures.
5. Fig 1G: the image is difficult to apprehend. Could the authors annotate it better or show other images (as supplementary data maybe). Besides, the conclusion drawn from this experiment is not entirely convincing based on these data: "RNF26-IR remains in the ER membrane when in proximity to Vimentin (Fig. 1G)."
6. Does the knock-down of UBE2J1 has an effect on RNF26 localization?
7. Fig. 1E-F: FRAP experiments
a. The graph in Fig. 1F-left should show the standard deviation on each curve. It is surprising that the WT curves has so many irregularities for a mean.
b. The recovery is really low; could the authors analyze the recovery obtained with other ER-bound proteins to be able to interpret this data properly. It is for instance really strange that the RING-deleted mutant does not recover more, suggesting that the protein in quite immobile in the ER (even when not attached to vimentin).
8. Fig. 2B, C: the choice of the quantification method is not really the best one. Why didn't the authors use the method they show Fig. 2G to quantify the distribution of RNF26?
9. Fig. 2B: rescue of the phenotype upon re-expression of Vimentin is missing.
10. Fig. 2D: this panel is not described in the text.
11. Fig. EV2A: the localization of VAP-A seems to be altered in Vimentin KO cells.
12. Fig. 2G: the method, in particular how it is weighed for bin size, should be described in the materials and methods section.
13. Fig. 2F: rescue of the phenotype upon re-expression of Vimentin is missing.
14. Fig. EV3: Is VAP-A distribution affected by RNF26 expression; for instance, in cells expressing the IR mutant, the localization of VAPA seems to be punctiform. Does this reflect a modification of the ER or a specific change in the localization of VAPA?
15. Line 273: the term bilaterally is not really appropriate: lateral implies that it concerns the sides of the cell.
16. Line 304: are proteolytic lysosomes really recruited to PN ER after tunicamycin treatment? Indeed, in Fig. 1 the authors describe the fact that lysosomes are already present in the PN in untreated cells (line 169-170).
17. Line 307-309: there are no co-labelling between RNF26 and the ERQC.
18. Line 275: "Meanwhile" is odd.
19. Line 283: why "once again"?

Referee #3:

In this manuscript Cremer et al. studied the interaction of vimentin intermediate filaments with RNF26 and the effect on the ER. The authors used a comprehensive set of experiments to study a possible interaction between the two proteins. They clearly, the impact of vimentin filaments on the ER architecture as well as the effect of altered RNF26 on vimentin meshwork

organization at the nuclear perinuclear cellular regions. The finding that vimentin is important and involved in ER organization is of high importance.

1. While the effect of each protein on the other (namely RNF26 and Vimentin) is well established in this work. Evidence for a direct interaction is missing. The proximity experiments shown here (based on TurboID) have a very limited resolution (not better than 20nm). This is very large distance on single molecular scale.
2. The tomography analysis missing statistics. The authors show several instances in which a density (maybe) bridges between the ER and Vimentin filaments. A distance analysis would be relevant. This reviewer does not detect a clear significant density that bridges the gap.
3. Similarly, to the previous point. Gold labeling experiments require a proper statistics (as the authors used in their other experiments)
4. P.7 The authors discuss a direct binding between RNP26 and ER based on Fig. EV1D- Please show a collection of the interactions that were identified.
5. Some experiments were conducted with U2OS cells and some other with HeLA cells. While additional cell line is always welcome, it would have been useful to show that all the experiments can be conduced using U2OS.
6. The IR mutant displays a filamenous appearance (Fig.1F), however, it seems that there is an over expression in the mutated cell in comparison to the wildtype.
7. P. 8-"Structural prediction..." Which structural predictions ? how were they made ?
8. The Tyrosine mutation that inactivate the RNF26 also affect the interaction with vimentin. This would suggest that vimentin-ER proximity somehow depends on RNF26 but make the suggestion that it is a direct interaction, weaker. A phenylalanine would have been a better choice for mutation than alanine

** As a service to authors, EMBO Press provides authors with the possibility to transfer a manuscript that one journal cannot offer to publish to another EMBO publication or the open access journal Life Science Alliance launched in partnership between EMBO Press, Rockefeller University Press and Cold Spring Harbor Laboratory Press. The full manuscript and if applicable, reviewers' reports, are automatically sent to the receiving journal to allow for fast handling and a prompt decision on your manuscript. For more details of this service, and to transfer your manuscript please click on Link Not Available. **

Please do not share this URL as it will give anyone who clicks it access to your account.

Point-by-point response

Below you can find detailed responses of the authors to the points raised during the review process for the manuscript by Cremer et al., entitled "RNF26 binds Vimentin intermediate filaments to integrate ER and endolysosomal responses to proteotoxic stress" **(EMBOJ-2022-111252)**. We thank the Editor and Reviewers for their insightful commentary and constructive suggestions and feel that the revised (and greatly expanded) manuscript has improved markedly as a result. We hope that all concerned deem our revision efforts compelling and look forward to finalizing our study for publication in the *EMBO Journal*.

Referee #1: In the current submission, the authors describe a functional link between the intermediate filament (IF) cytoskeleton, specifically the protein vimentin, and an ER localized ubiquitin ligase ring finger protein 26 (RNF26). These proteins work together to control ER morphology and organelle localization/ compartmentalization during cellular homeostasis and ER stress.

In this study the authors show that the IF cytoskeletal protein vimentin can act as perinuclear anchors of the ER and the endo-lysosomal system by binding to RNF26 and that these anchors can be perturbed by knock out of vimentin or the silencing or mutation of the ER localized RNF26. When the link between the two proteins is broken, the ER as well as the other organelles of the endo-lysosomal system lose their perinuclear localization and take on a more evenly dispersed localization throughout the cell. The authors show that vimentin preferentially binds to inactive RNF26, and that this binding holds the RNF26 in the perinuclear region where vimentin is enriched. The enrichment is shown by colocalization studies and quantitation using Manders' coefficients, immunogold labelling, development of an ImageJ plug-in used to show preferential perinuclear localization of the two proteins over peripheral localization, proximity biotinylation using TurboID, vimentin knockout cell lines, siRNA against RNF26, point mutation or truncated forms of RNF26, and treatment of cells with tunicamycin to induce ER stress. The authors go so far as to show that the last 10 C-terminal amino acid residues of RNF26 are required for the binding of RNF26 with vimentin and therefore localization to the perinuclear region. The study has much to like with some busy work suggestions provide below that will not change the outcome of the story but make it more rigorous and consistent.
We thank the Reviewer for their favorable assessment of our study as having *"much to like"* and appreciate the constructive suggestions offered to improve it.

1. Perhaps the most substantial issue to address is mentioning the paper by Rodriguez and coworkers (J virol 2002) that demonstrated a link between vimentin and the ER/ERGIC. I'm not sure this study was even cited? So the authors need to make the case that their current study makes a meaningful advance beyond this 20 year old story.
We apologize for the aforementioned omission of important literature. The suggested citation has now been incorporated in the revised manuscript on **line 437** (Risco, C., et al. (2002). "Endoplasmic reticulum-Golgi intermediate compartment membranes and vimentin filaments participate in vaccinia virus assembly." J Virol **76**(4): 1839-1855). Regarding the question of novelty, our work goes far beyond a mere visual association between Vimentin and the ERGIC/VV viral factories to define a new mode of architectural control of two types of organelles – the ER and endolysosomal system – whose interplay through RNF26 and Vimentin facilitates cellular homeostasis and resolution of ER stress responses.

2. Fig.5 is a key figure in this paper. For ER stress, can blotting with ER stress marker antibodies, such as BIP, p-eIF2a, eIF2a, ATF4, CHO after TM treatment be included? Also, some viability data after TM treatment at different concentrations would be good.
The suggested ER stress marker analyses have been performed, demonstrating that ER stress-associated signaling is still induced in cells deficient for either RNF26 or Vimentin (**Fig. 6F, G**). This indicates that failure to form the perinuclear ERQC compartment upon tunicamycin challenge (**Fig. 6A-E**) is not due to lack of stress-sensing capabilities. We did however identify a substantial effect of RNF26 and Vimentin on Sec62-

mediated ERphagy (**Fig. 8**) operational during resolution of ER stress. See also the response to point #7 below.

Cell viability was also examined as requested, and the results of these experiments are summarized in the Rebuttal-associated **Fig. R1**. Here, tunicamycin-induced apoptosis was measured using AnnexinV labeling, followed by flow cytometry analysis. Using this method, we observed a moderate increase in cell death after 24 hours of tunicamycin treatment (5µg/mL) following RNF26 silencing or Vimentin knock-out. However, in light of no appreciable effects of either genetic perturbation on stress-mediated (apoptotic) signaling (**Fig. 6G**), cell viability data were not included in the revised manuscript.

Fig.1F
- What patten of RNF26-ΔRING is displayed in U2OS cells? What is the relationship of RNF26-ΔRING with vimentin filaments? Please show the IF data. All relevant images are now included in **Fig. 3A**.

Fig.2E - Show RNF26 and vimentin protein levels in U2OS cells under the control, RNF26 siRNA KD & Vim KO by WB. Immunoblot and quantification of Vimentin protein levels as a function of RNF26 depletion are now shown in **Fig. 3H**.

Fig.3B & G - Please be consistent in using the same cell type (U2OS) (HeLa cells data can be put in a suppl. Figure). To show consistency in our observations across cell lines, proximity-based biotinylation experiments previously shown in HeLa cells have been reproduced in U2OS cells. As expected, increase in Vimentin recovery after neutravidin pulldown from U2OS cells expressing TurboID-I382R as compared to wild type RNF26 and the ΔRING truncation mutant. Results from both cell lines are now presented in **Fig. 2D**.

-When loading a control can actin/tubulin be used? The use of vimentin is confusing.
Vimentin is used to demonstrate input levels in precipitation assays. Throughout the manuscript, Vinculin (not Vimentin) is used as lysate control for whole cell lysate analyses.

Fig.4A - Show WB of parental, Vim KO#1 & RNF26 -2 siRNA KD in U2OS cells. Blot by anti-Vim & anti-RNF26 antibodies. Confirmatory western blots demonstrating Vimentin and RNF26 ablation in U2OS cells can now be found in **Figs. 4B** and **EV1B**, respectively.

Fig.4H - Missing figure legend. We apologize for this negligence. All figures and legends have been thoroughly revised.

Fig.5D - Can RNF26 & vimentin protein levels in parental, RNF26 siRNA treated & Vim KO U2OS cells by WB be shown? Please refer to **Figs. EV1B** and **Fig. 4B**, respectively.

Also, Fig.1A & 4D used Cherry-tagged KDEL. But in M & M Constructs, only GFP-KDEL was mentioned. This has been corrected.

Collectively, the points above (cosmetic, textual, or pertaining to the choice of cell lines) have been addressed as requested. We appreciate the Reviewer's detailed approach.

Minor issues:
1) What is near-super-resolution? Please describe in detail or change to high resolution or super resolution.
The term near-super-resolution has been removed.

2) The immuno-gold image in figure1 is very difficult to see what is going on. The gold looks sharp, but the ultrastructure is uninterpretable. Immuno-gold labeling data has been removed from the manuscript. Instead, more extensive analysis of the cryoEM dataset is now provided in **Fig. 5A**.

3) There is quantitation in a couple of figures where the reader is referred to other images in other figures. Please put images of what is being quantitated and the graphs in the same figures. Data presentation has been rearranged accordingly. We apologize for prior inconvenience.

4) Figure 4C has no legend, and all the rest are shifted down a letter. All figures and legends have been thoroughly revised.

5) Materials and methods section needs part on cell lines used. This has now been included.

6) Most of the experiments are done in U2OS cells except the immuno-precipitations and BioID which were done with HeLa. Can these expts be done in the U2OS cells? All relevant experiments previously performed in HeLa cells have now been recapitulated in U2OS cells as requested. BioID data from both cell lines is now presented in **Fig. 2D**.

7) The ER stress component was not explored very well as only an increase in the perinuclear localization upon Tunicamycin treatment. It would have been nice to show the converse (if possible) when treating cells with ER stress inhibitors.
The revised manuscript greatly expands upon our original findings regarding the role of perinuclear localization of the ER and endolysosomes under conditions of ER stress. **A number of new insights are provided implicating RNF26 and Vimentin as positive regulators of cellular responses to proteotoxic stress.** Notably, we demonstrate that RNF26 associates with the known ERQC organizer HERP1 (by co-IP and colocalization, **Fig. 7A-C**) and the ERAD ubiquitin ligase HRD1 (by co-IP, **Fig. 7C**), and that perinuclear localization of HERP1-positive ER subdomains relies on the presence of RNF26 and Vimentin (**Fig. 7D-G**). Furthermore, we find that Sec62-mediated ERphagy, or direct feeding of ER cargoes into the lysosome operational during recovery from ER stress (Fumagalli *et al*, 2016; Loi *et al*, 2019), depends on a functional RNF26/Vimentin axis (**Fig. 8A-H**). For further elaboration on these new findings please refer to our response to point #6 of Reviewer 2.

Referee #2: This manuscript entitled "Vimentin intermediate filaments organize organellar architecture in response to ER stress" by T. Cremer and collaborators describes the role of Vimentin intermediate filaments in the spatial organization of organelles such as endosomes and the endoplasmic reticulum (ER).

The authors build on their previous findings identifying a key role for the ER-bound ubiquitin ligase RNF26 (Ring finger protein 26) in the localization of endocytic organelles. They first confirmed from their previous work, that Vimentin is a binding partner of RNF26, and showed that this interaction requires the C-terminal tail of RNF26. The authors then showed that Vimentin controls the distribution of RNF26 in the ER network, and consequently the localization of endolysosomes. Finally, the authors link the function of vimentin and RNF26 to the localization of the ER network and to ER stress. The observation of a role of intermediate filament in the distribution of organelles is novel and an interesting. However, there are several major issues with the manuscript that need to be addressed to fully support this idea: in particular, the data showing the interaction between RNF26 and Vimentin are quite weak, and the physiological consequences for the interaction are missing. We thank the Reviewer for their favorable assessment of our study as *"novel and interesting"* and appreciate the constructive suggestions offered to improve it. All points raised have now been thoroughly addressed in the revised manuscript as follows.

Major points:

1. The data showing the interaction between RNF26 and Vimentin are quite weak:

a. The authors refer to Jongsma et al, 2016 (Line 181-182) to claim that Vimentin co-precipitates with the RING domain of RNF26. I could not find data on Vimentin in this article, nor the mass spectrometry data on a repository (see for instance https://thebiogrid.org/206011/publication/an-er-associated-pathway-defines-endosomal-architecture-for-controlled-cargo-transport.html).

The proteomics data referred to above has now been made available in connection with the present manuscript. Silver stain bands analyzed by mass spectrometry are shown in **Fig. EV2A** and protein partners identified are summarized in **Fig. 2B** (selected) and **EV Table 1** (full list). PRIDE accession code PXD040760 (Reviewer account details: Username: reviewer_pxd040760@ebi.ac.uk Password: NahWWKrE). We apologize for prior inconvenience locating this relevant data.

b. The interaction assay the authors used is based on biotin-ligation mediated by TurboID: it is sensu stricto a proximity assay rather than an interaction assay. These data should be confirmed by other approaches: if this reviewer understands what has been done in Jongsma et al correctly, GST-pull-down experiments were performed with the RING domain of RNF26. These experiments should be repeated to show the interaction with Vimentin. Besides, another more cellular approach should be performed (co-immunoprecipitation, FRET etc...). In the course of the revision process, we were able to demonstrate that RNF26 and Vimentin bind directly using several independent *in vitro* methods (**Figs. 2** and **EV2**), which are detailed under point (d) below. We feel that these lines of evidence, together with the proteomic data (see point (a) above), proximity-based labeling (**Fig. 2**) and colocalization studies (**Fig. 3**), provide abundant support for the interaction between Vimentin and RNF26.

c. There are no data on the interaction site in Vimentin. The authors should identify the binding surface of RNF26 on Vimentin using deletion mutants, point mutants...
We agree with the Reviewer that it would be desirable to learn where on Vimentin the interaction with RNF26 occurs. To address this point, Vimentin truncation mutants were constructed (**Fig. R3A**) and expressed in Vimentin KO cells. Regrettably, we observed that several of these mutants are highly prone to aggregation and/or mislocalization in cells (**Fig. R3B**), making interpretations tricky. Nevertheless, one truncation mutant containing Rod 2 of Vimentin was found to exhibit ER-like localization and partial overlap with ectopically co-expressed GFP-RNF26-I382R (**Fig. R3C**, white overlay), suggesting that this region could facilitate binding to RNF26.

Additionally, we defined the binding surface of Vimentin on RNF26, as described under point (d) below.

d. The authors should ideally reconstitute the complex in vitro using recombinant vimentin and peptides if they suspect that the interaction is mediated solely by the short C-terminal extremity of RNF26.
To address this, we carried out several *in vitro* interaction studies as follows. Firstly, co-precipitation between purified Vimentin and purified (catalytically inactive) RING domain of RNF26 (**Fig. EV2B**) was performed, demonstrating direct binding between them (**Fig. 2E**). This was further validated by preassembling Vimentin filaments unto glass slides and incubating with Rhodamine-labeled purified (catalytically inactive) RING domain of RNF26 (**Fig. 2F** and **EV2C**). Lastly, fluoresce polarization of Rhodamine-labeled peptide containing the last 10 residues of RNF26 in the presence of purified Vimentin (**Fig. 2H**) showed concentration-dependent binding, and removal of this sequence abrogated proximity-based biotinylation of endogenous Vimentin by RNF26 (**Fig. 2G**). **Taken together, these data imply that the short C-terminal sequence of RNF26 is necessary and sufficient to mediate a direct interaction with Vimentin**.

2. This reviewer is not convinced by the way data are analyzed in the manuscript. Most of the time (Fig. 2C, 2D, 2H, 2I, 3D, 3E, 4B, 4C, 4F, 4H, 5E and supplementary), for data obtained from image analyses, data from individual cells are compared and used to perform statistical analyses. The authors should use data from independent experiments (means) to perform statistical analyses.
Respectfully, to the best of our knowledge, consideration of individual cells as 'independent' in statistical terms is widely accepted, including in recent publications in the *EMBO Journal* (Bulthuis *et al*, 2023; Jongsma *et al*, 2020) when similar numbers of cells are analyzed between samples, and the exact number of cells plotted for each condition is indicated in the legend. In our view, plotting of individual data points provides an overview of the variation observed within a population of cells, which is hidden when means only are used.

3. Is Vimentin localization affected by RNF26? For instance, the localization of Vimentin seems different Fig. 1F between cells expressing RNF26 WT or RNF26 I382R. This should be quantified.
Indeed, we find that RNF26 expression and activity have a measurable impact on the intracellular localization of Vimentin filaments. New data pertaining to this point can be found in **Fig. 3A, E-H**. We thank the Reviewer for this suggestion.

4. Does vimentin deficiency affect the localization of other organelles (mitochondria, Golgi, lipid droplets...). This should be analyzed.
The Reviewer brings up an interesting point, particularly in light of recent findings by others demonstrating that alterations in perinuclear ER distribution (such as under conditions of disturbed microtubule modification (Zheng *et al*, 2022)) coincide with changes in the localization of other organelles. We therefore examined the distribution of mitochondria, lipid droplets and the Golgi apparatus in our U2OS Vimentin KO cells relative to parental controls. Recapitulating previous reports (Tang *et al*, 2008), we observe that perinuclear distribution of mitochondria depends on Vimentin (**Fig. R4A**). Furthermore, enhanced Golgi clustering was observed in Vimentin KO cells ectopically re-expressing Vimentin as compared to untransfected Vimentin KO cells (**Fig. R4B**). The latter observation is in line with a previously identified interaction between Vimentin and the Golgi protein FTCD (Gao *et al*, 2002), as well as a recent preprint thoroughly investigating Golgi morphology in Vimentin KO cells (Vitali *et al*, 2022). By contrast, lipid droplets do not appear to exhibit a strong perinuclear accumulation in U2OS cells, and their distribution is therefore not significantly affected by Vimentin ablation (**Fig. R4C**).

5. In absence of RNF26 or Vimentin, is the morphology of the ER affected? The density of ER structures, the quantity of ER-resident proteins should be characterized.
We have now greatly expanded our characterization of ER organization and dynamics under conditions of RNF26 (**Figs. 1 and EV1**) and Vimentin (**Figs. 5 and EV4**) loss. We show that absence of either protein causes spreading of ER sheets towards the cell periphery and increases motility of the perinuclear ER. Additionally, in the case of Vimentin ablation, cellular abundance of ER sheets is significantly increased (**Fig. 5F**), implying that interactions with Vimentin intermediate filaments regulate ER morphology and distribution of functional elements therein. **Collectively, these data strongly implicate RNF26 and Vimentin as important players in the spatiotemporal control of ER architecture and membrane dynamics.**

6. The functional consequence of the ERQC formation defects in RNF26 and Vimentin-deficient cells needs to be explored; is the cell response to ER stress induction altered (activation of the different ER stress pathways)? are the cells able to cope with ER stress induction or are they more sensitive to cell death? Etc...
This point pertains to the overarching question of cellular physiology. We fully agree with the Reviewer that characterization of the role of RNF26 and Vimentin in the context of cellular responses to ER stress constitute an important addition to the study. **The revised manuscript greatly expands upon the findings reported in**

the first submission, implicating both RNF26 and Vimentin as positive regulators of cellular responses to proteotoxic stress:

Firstly, we show that loss of RNF26 or Vimentin does not appreciably affect sensing of ER stress, but that the response to stress – in the form of organellar remodeling – is abrogated (**Fig. 6**).

Secondly, we demonstrate that RNF26 interacts with the established components of the ERQC, including HERP1 and HRD1, and that the resulting complexes are underpinned by the Vimentin cytoskeleton (**Fig. 7**).

Finally, we find that Sec62-dependent ERphagy is dependent on a functional RNF26/Vimentin axis (**Fig. 8**). The Sec62 ERphagy pathway has recently been described to govern direct deposition of ER membrane segments into lysosomes for clearance during the recovery phase following ER stress induction (Fumagalli *et al.*, 2016). **Our new evidence therefore strongly supports the hypothesis that compartmentalization of the ER membrane and its juxtaposition with lysosomes in the perinuclear region of the cell – as afforded by RNF26 and Vimentin – enables efficient ER clearance in response to proteotoxic stress.**

7. Line 272-286: "Collectively, these results suggest that RNF26 and Vimentin are required to maintain compartmentalization of membrane composition in the ER.". Is the complex RNF26-vimentin required, or is the enzymatic activity of RNF26 involved? A rescue experiment using the WT and IR mutant RNF26 could answer this question.
We have previously demonstrated that enzymatic activity of RNF26 supports perinuclear accumulation of endolysosomes (Jongsma *et al*, 2016). We now show that cells expressing catalytically inactive RNF26 fail to form GFP-negative, HALO-positive Sec62-containing acidic compartments, as compared to either control cells or those overexpressing wild type RNF26 (**Fig. 8G, H**). In addition, we find that depletion of UBE2J1, the E2 collaborating with RNF26 in the organization of the perinuclear endolysosomal cloud (Cremer et al, 2021), phenocopies removal of RNF26 in the context of tunicamycin-induced formation of perinuclear ERQC domains (**Fig. 6D, E**). Taken together, these considerations strongly suggest that – in addition to ubiquitylation-independent interactions with the Vimentin cytoskeleton – **ligase activity of RNF26 is relevant in the context of ERQC formation and ER clearance by the endolysosomal system**.

Minor points:

1. Fig 1A: the choice of LUT is not the best to look at co-localizations. Colocalization data presentation has been revised throughout the manuscript to improve visualization of signal overlaps.

2. Line 34-36: The sentence "While the ubiquitin...tail." is odd: it links two unrelated elements: localization on the one hand, and protein-protein interaction on the other. Text pertaining to this point has been revised.

3. Fig 1C: the authors should describe how they discriminated vimentin filaments in their tomograms.
Cytoskeletal elements were discriminated primarily based on width. Microtubules have a width of ~ 25 nm and form hollow tubes, which Vimentin filaments do not. Differences between Vimentin and Actin were more difficult to ascertain, however, tomographic slices enable filament thicknesses to be quite accurately determined (**Fig. EV4**).

Apart from their thickness, the major cytoskeletal elements differ in their spatial distribution. Actin for instance is generally found in bundles at the cell surface or borders, while Vimentin predominates in the central region of the cell. Also, actin is often more branched and Vimentin filaments are typically smoother.

To validate our identification of Vimentin filaments, EM analysis was performed on U2OS Vimentin KO cells, detecting no filamentous structures previously annotated as intermediate filaments (**Fig. R5**).

4. Fig. 1F: the 2 images shown here are apparently not at the same z level, which might affect the appearance of cellular structures.
Data presentation in Figure 1 has been revised. We have also ensured that all microcopy images shown throughout the manuscript show Z-slices from the same height.

5. Fig 1G: the image is difficult to apprehend. Could the authors annotate it better or show other images (as supplementary data maybe). Besides, the conclusion drawn from this experiment is not entirely convincing based on these data: "RNF26-IR remains in the ER membrane when in proximity to Vimentin (Fig. 1G)."
Immunolabeling of RNF26 in combination with preservation of ER membrane structures has been removed from the manuscript.

6. Does the knock-down of UBE2J1 has an effect on RNF26 localization?
To answer this question, we would like to refer the Reviewer to our previous publication, where we investigated RNF26 localization in UBE2J1 KO cells (Cremer *et al*, 2021). Data presented in Fig. S3D of that study shows that RNF26 accumulation in the perinuclear area is independent of UBE2J1 expression.

7. Fig. 1E-F: FRAP experiments
a. The graph in Fig. 1F-left should show the standard deviation on each curve. It is surprising that the WT curves has so many irregularities for a mean.
b. The recovery is really low; could the authors analyze the recovery obtained with other ER-bound proteins to be able to interpret this data properly. It is for instance really strange that the RING-deleted mutant does not recover more, suggesting that the protein in quite immobile in the ER (even when not attached to vimentin).
The Reviewer makes a valid point. To address this, we repeated the experiment to compare the recovery of RFP-RNF26 with that of mCherry-Calnexin (**Fig. R6**). We find that the latter recovers much faster than RFP-RNF26, indeed suggesting that mobility of RNF26 in the ER membrane is quite restricted. This could be explained for instance by the difference in transmembrane interactions, for Calnexin harbors only one TMD, while RNF26 possesses 5. This possibility is further supported by RING-independent interactions of RNF26 with ERAD/ERQC proteins HERP1 and HRD1 (**Fig. 7C**) and UBE2J1 (Cremer *et al.*, 2021).

8. Fig. 2B, C: the choice of the quantification method is not really the best one. Why didn't the authors use the method they show Fig. 2G to quantify the distribution of RNF26?
This has been addressed using our perinuclearity quantification method (**Fig. 1C, D**). Results of the new analysis are in line with the conclusions asserted in the first submission.

9. Fig. 2B: rescue of the phenotype upon re-expression of Vimentin is missing.
We refer the reviewer to the data in **Fig. EV3A**, where RNF26-I382R exhibits strongly perinuclear filamentous distribution in Vimentin KO cells re-expressing Vimentin as compared to their Vimentin-deficient counterparts.

10. Fig. 2D: this panel is not described in the text.
Data presentation has been revised and RNF26/VAP-A overlap is now explicitly mentioned in the Results section.

11. Fig. EV2A: the localization of VAP-A seems to be altered in Vimentin KO cells.
As thoroughly described in **Figs. 5** and **EV4**, localization of VAP-A (as well as that of KDEL and CLIMP63) is indeed dependent on the presence of Vimentin.

12. Fig. 2G: the method, in particular how it is weighed for bin size, should be described in the materials and methods section. We now include a thorough description of our perinuclearity quantification method in the

Materials and Method section. The macro measures mean fluorescence intensity (MFI), which is a parameter independent of bin size.

13. Fig. 2F: rescue of the phenotype upon re-expression of Vimentin is missing.
Vimentin rescue for the distribution of endolysosomes appears in **Fig. 4E, F**. We have previously implicated RNF26 in the architecture of the endolysosomal system (Jongsma *et al.*, 2016) as a whole and now show that Vimentin KO phenocopies the effect of RNF26 silencing on fluid-phase endocytosis in the revised manuscript (**Fig. 4G, H**). These data support the hypothesis that **Vimentin regulates endosome biology by ensuring perinuclear distribution of RNF26 in the ER membrane**.

14. Fig. EV3: Is VAP-A distribution affected by RNF26 expression; for instance, in cells expressing the IR mutant, the localization of VAPA seems to be punctiform. Does this reflect a modification of the ER or a specific change in the localization of VAPA?
RNF26 has been shown to interact with numerous proteins within the ER membrane (Fenech *et al*, 2020). RNF26 is therefore likely to exert substantial influence on the ER through these partnerships, which fall outside the scope of the present study focused on the interplay between RNF26 and the Vimentin cytoskeleton.

15. Line 273: the term bilaterally is not really appropriate: lateral implies that it concerns the sides of the cell.
Terminology has been adjusted.

16. Line 304: are proteolytic lysosomes really recruited to PN ER after tunicamycin treatment? Indeed, in Fig. 1 the authors describe the fact that lysosomes are already present in the PN in untreated cells (line 169-170).
While the bulk of lysosomes resides within the perinuclear area under steady state conditions, in line with another report (Bae *et al*, 2019), exaggerated perinuclear clustering of lysosomes is observed in cells that experience ER stress (**Fig. EV5D, E**). Furthermore, perinuclear positioning of lysosomes – both in unperturbed cells (Cremer *et al.*, 2021) and in response to tunicamycin treatment (**Fig. EV5D, E**) – depends on RNF26, underscoring the functional importance of this proteins in the context of ER stress.

17. Line 307-309: there are no co-labelling between RNF26 and the ERQC.
In the revised manuscript, we show colocalization and interaction of RNF26 with HERP1 (**Fig. 7A-C**), as well as HRD1 (**Fig. 7C**), which a mediated through transmembrane determinants. From this we conclude that RNF26 is a member of the ERQC.

18. Line 275: "Meanwhile" is odd. The text has been adjusted accordingly.

19. Line 283: why "once again"? The text has been adjusted accordingly.

Referee #3: In this manuscript Cremer et al. studied the interaction of vimentin intermediate filaments with RNF26 and the effect on the ER. The authors used a comprehensive set of experiments to study a possible interaction between the two proteins. They clearly, the impact of vimentin filaments on the ER architecture as well as the effect of altered RNF26 on vimentin meshwork organization at the nuclear perinuclear cellular regions. The finding that vimentin is important and involved in ER organization is of high importance.
We thank the Reviewer for their favorable assessment of our study's value to the community with *"high importance"* and the constructive suggestions and criticisms offered to improve it. These will be thoroughly addressed in the revised manuscript as follows.

1. While the effect of each protein on the other (namely RNF26 and Vimentin) is well established in this work.

Evidence for a direct interaction is missing. The proximity experiments shown here (based on TurboID) have a very limited resolution (not better than 20nm). This is very large distance on single molecular scale.
The Reviewer makes an important point, which has now been thoroughly addressed with several new experiments presented in the revised **Fig. 2**. Collectively, our data support a direct interaction between Vimentin and the C-terminal tail sequence of RNF26. For details on these new data please refer to the response to point #1a-d of Reviewer 2.

2. The tomography analysis missing statistics. The authors show several instances in which a density (maybe) bridges between the ER and Vimentin filaments. A distance analysis would be relevant. This reviewer does not detect a clear significant density that bridges the gap.
Quantification is now provided in **Fig. 5A**, which was obtained by measuring the shortest distance between IFs and ER membranes satisfying the following conditions: 1 IF running parallel to the ER and 2 that a clear density could be detected anywhere along the space between the two elements (in any Z-stack within the tomogram). For clarity, we included an arrow at such point where a density was detected (**Fig. EV4B**, white).

3. Similarly, to the previous point. Gold labeling experiments require a proper statistics (as the authors used in their other experiments). These data have been removed from the revised manuscript. See also the response to minor point #5 of Reviewer 2.

4. P.7 The authors discuss a direct binding between RNF26 and ER based on Fig. EV1D- Please show a collection of the interactions that were identified.
RNF26 has previously been extensively described as an ER embedded protein by us and others (Qin et al, Plos Paathog 2014; Jongsma and Berlin et al, Cell 2016; Fenech et al, ELife 2020). We are now providing a full proteomic dataset on precipitates recovered with the purified RING domain (**Fig. 2B**, **Table EV1** and **PRIDE PXD040760**).

5. Some experiments were conducted with U2OS cells and some other with HeLA cells. While additional cell line is always welcome, it would have been useful to show that all the experiments can be conducted using U2OS. All relevant experiments have been repeated in U2OS cells. See also response to comment of Reviewer 1 under 'Fig.3B & G'.

6. The IR mutant displays a filamenous appearance (Fig.1F), however, it seems that there is an over expression in the mutated cell in comparison to the wildtype.
The IR mutant is more stable relative to its wild type counterpart (as can be gleamed from immunoblots against tagged RNF26 and mutants), which functions in ubiquitin ligation. Wild type RNF26 is itself ubiquitinated and turned over in a ubiquitin-dependent manner (Fenech *et al.*, 2020; Jongsma *et al.*, 2016). For this reason, cells often express wild type RNF26 at lower levels as compared to its inactive variants.

7. P. 8-"Structural prediction..." Which structural predictions? how were they made? This has been clarified in the text pertaining to the new **Fig. EV2D, E**.

8. The Tyrosine mutation that inactivate the RNF26 also affect the interaction with vimentin. This would suggest that vimentin-ER proximity somehow depends on RNF26 but make the suggestion that it is a direct interaction, weaker. A phenylalanine would have been a better choice for mutation than alanine.
We agree that the combination of activities carried out by a single residue may be confusing. For this reason, the data pertaining to the tyrosine mutation of RNF26 have been removed from the revised manuscript.

References:

Bae D, Moore KA, Mella JM, Hayashi SY, Hollien J (2019) Degradation of Blos1 mRNA by IRE1 repositions lysosomes and protects cells from stress. *Journal of Cell Biology* 218: 1118-1127

Bulthuis EP, Dieteren CEJ, Bergmans J, Berkhout J, Wagenaars JA, van de Westerlo EMA, Podhumljak E, Hink MA, Hesp LFB, Rosa HS *et al* (2023) Stress-dependent macromolecular crowding in the mitochondrial matrix. *Embo j*: e108533

Cremer T, Jongsma MLM, Trulsson F, Vertegaal ACO, Neefjes J, Berlin I (2021) The ER-embedded UBE2J1/RNF26 ubiquitylation complex exerts spatiotemporal control over the endolysosomal pathway. *Cell Rep* 34: 108659

Fenech EJ, Lari F, Charles PD, Fischer R, Laetitia-Thezenas M, Bagola K, Paton AW, Paton JC, Gyrd-Hansen M, Kessler BM *et al* (2020) Interaction mapping of endoplasmic reticulum ubiquitin ligases identifies modulators of innate immune signalling. *Elife* 9: 2020.2003.2018.993998

Fumagalli F, Noack J, Bergmann TJ, Cebollero E, Pisoni GB, Fasana E, Fregno I, Galli C, Loi M, Solda T *et al* (2016) Translocon component Sec62 acts in endoplasmic reticulum turnover during stress recovery. *Nat Cell Biol* 18: 1173-1184

Gao YS, Vrielink A, MacKenzie R, Sztul E (2002) A novel type of regulation of the vimentin intermediate filament cytoskeleton by a Golgi protein. *Eur J Cell Biol* 81: 391-401

Jongsma ML, Bakker J, Cabukusta B, Liv N, van Elsland D, Fermie J, Akkermans JL, Kuijl C, van der Zanden SY, Janssen L *et al* (2020) SKIP-HOPS recruits TBC1D15 for a Rab7-to-Arl8b identity switch to control late endosome transport. *Embo j* 39: e102301

Jongsma ML, Berlin I, Wijdeven RH, Janssen L, Janssen GM, Garstka MA, Janssen H, Mensink M, van Veelen PA, Spaapen RM *et al* (2016) An ER-Associated Pathway Defines Endosomal Architecture for Controlled Cargo Transport. *Cell* 166: 152-166

Loi M, Raimondi A, Morone D, Molinari M (2019) ESCRT-III-driven piecemeal micro-ER-phagy remodels the ER during recovery from ER stress. *Nature Communications* 10: 5058

Tang HL, Lung HL, Wu KC, Le AH, Tang HM, Fung MC (2008) Vimentin supports mitochondrial morphology and organization. *Biochem J* 410: 141-146

Vitali T, Witkos TM, Cutiongco MFA, Yan G, Mironov AA, Swift J, Lowe M (2022) Vimentin intermediate filaments provide structural stability to the mammalian Golgi apparatus. *bioRxiv*: 2022.2008.2025.505293

Zheng P, Obara CJ, Szczesna E, Nixon-Abell J, Mahalingan KK, Roll-Mecak A, Lippincott-Schwartz J, Blackstone C (2022) ER proteins decipher the tubulin code to regulate organelle distribution. *Nature* 601: 132-138

Rebuttal Figure Legends

R1. ER stress-induced cell death as a function of RNF26 and Vimentin. Cells were treated with Tunicamycin (5ug/mL, 24hr) induced apoptosis (or not), trypsinized and labeled for cell surface PS using AnnexinV (BioLegend, 1:20) prior to analysis by flow cytometry. Shown are a representative FACS plot of Annexin-V signals in tunicamycin- and untreated control cells as well as graphs of combined experiments (% of AnnexinV-positive cells) in (A) control U2OS cells or U2OS cells silenced for RNF26 (#1 or #2), (B) parental HeLa and U2OS cells (Parental) or Vim KO#2 U2OS and Vim KO#1 HeLa cells (Vim KO), from 4 (siRNF26) or 8 (Vim KO) independent experiments. Statistical analysis tested with paired t-test (circles = means, error bar = SEM).

R2. Proximity biotinylation of endogenous Vimentin by 2HA-TurboID-RNF26, catalytically inactive point mutant (I382R), or RING domain truncation mutant (ΔRING) in U2OS cells following 30 min incubation with or without biotin prior to lysis. Shown are representative immunoblots of neutravidin precipitates and lysate inputs stained against HA and Vimentin.

R3. Localization of truncated Vimentin versions in U2OS cells. A) Schematic representation of Vimentin truncation mutants generated alongside the full-length construct. All proteins were tagged with RFP on C-terminus. Vimentin globular domains are indicated (1A/B, and 2) as well as intrinsically disordered head and tail regions. B) Truncations were transiently expressed in Vimentin KO#2 cells and imaged by confocal microscopy. Shown are representative Z-slices. Cell boundaries and nucleus demarcated. Scale bar = 10µm.
C) Vimentin Rod2 truncation (245-407) was co-expressed with GFP-RNF26 I382R in U2OS Vim KO#2 cells, fixed, and imaged by confocal microscopy. Boxed-Zoom ins indicate overlap between Vimentin Rod 2 and GFP-RNF26 I382R (white). Scale bar = 10µm.

R4. Intracellular localization of mitochondria, lipid droplets, or the Golgi complex as a function of Vimentin. **A/C.** Shown are representative images of parental and Vimentin KO#2 cells that were incubated with Mitotracker Far-Red (A) or BodipY-493/453 (C) before live cell imaging, accompanied by perinuclearity analysis of mitochondria (A) and lipid droplets (C). **C.** U2OS Vimentin KO#2 cells were transfected with Vimentin_IRES-GFP (grey), fixed, and stained with a cocktail of golgin97 and GM130 antibody to visualize the Golgi complex (green). For quantification, cells were scored for a dispersed (or more around the nucleus) or clustered (tight and on one side of the nucleus) Golgi phenotype, as exemplified by microsopy images. HeLa sample set (HeLa parental or Vim KO#1) was also scored. Scale bar = 10m. Nucleus and cell boundaries demarcated.

R5. Identification of intermediate filaments among other cytoskeletal elements. Shown are zero tilt micrographs of a perinuclear region in Epon-embedded parental U2OS or Vim KO#2 cells. Boxed zoom-ins reveal presence of microtubules, actin filaments and intermediate filaments in parental cells, and only microtubules and actin filaments in Vim KO cells. Scale bar = 1µm. Zoom scale bar = 100nm.

R6. RNF26 mobility in ER membrane as a function of RING presence or activity. Fluorescence recovery after photobleaching (FRAP) of red fluorescence signal in HeLa cells expressing RFP-RNF26 WT, I382R or ΔRING or mCherry-Calnexin. Signal was bleached to approximately 20% of original before imaging of signal recovery every 3 sec for 50 frames. **B.** Quantifications of FRAP in (A), showing average signal recovery of RNF26 WT, I382R or

ΔRING or mCherry-Calnexin Scatter plot shows mean +/- SD of total recovery at endpoint (t=150s) in 13 (WT), 8 (I382R), 8 (ΔRING), 5 cells (mCherry-CNX).

Figures for reviewers

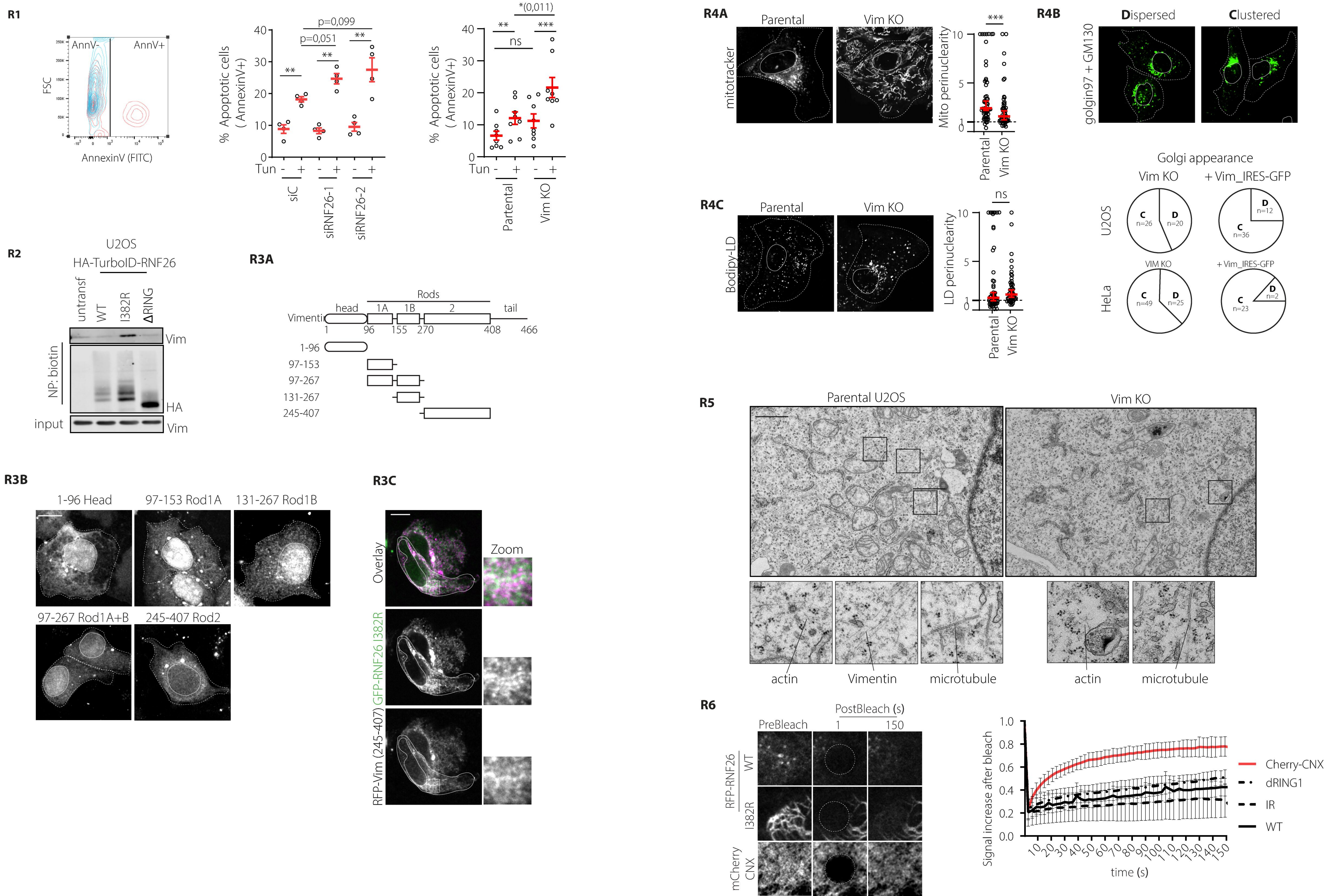

1st Revision - Editorial Decision 28th Apr 2023

Dear Jacques and Ilana,

We have now received re-review reports from one of the three referees that originally appraised your manuscript. The comments are given at the bottom of this email. As you will see, you have addressed the concerns satisfactorily. However, I would like you to consider in more detail the referee's point about the reproducibility of the data in the panels specified in the first review. In this regard, would you please state in the figure legends that the blots shown are representative of n individual experiments. If n=1, please repeat the experiment twice and state the same. For plots that represent image analysis, please state in the legend that each point represents a technical replicate (cells from the same plate), and that the experiment is representative of n individual experiments. Likewise, if n=1, please repeat.

There are also some remaining editorial points which need to be addressed. In this regard would you please:

• upload the manuscript text with no figures, no track changes, and with the Supporting Information section and synopsis text removed,
• include a Data Availability section which contains links to data sets generated in the study such as from the mass spectrometry analysis,
• rename the Conflict of Interest Section as the "DISCLOSURE AND COMPETING INTERESTS STATEMENT" according to the instructions on our website,
•remove the AC/CRediT section from the manuscript text,
• include the vimentin ko image (Fig EV4 panel C) currently labelled "not shown" according to the instructions on our website,
• include callouts for Movies EV1-EV8. The legends should be zipped with each movie file,
• include an Appendix 1 file which contains a table of contents with page numbers,
• reorganize the section order according to the instructions on our website, and
• check the email address given for the co-author George Janssen.

We require the publication of source data for electrophoretic gels and blots, with the aim of making primary data more accessible and transparent to the reader. Please therefore provide me with a PDF file per figure that contains the original, uncropped and unprocessed scans of all or key gels used in the figures. The PDF files should be labeled with the appropriate figure/panel number, and should have molecular weight markers; further annotation could be useful but is not essential. The PDF files will be published online with the article as supplementary "Source Data" files. Source Data can also include Excel tables to accompany your graphs. We anticipate that their inclusion will make your work more discoverable and usable to scientists in the future. Hannah Sonntag will contact you separately about the Source Data for this manuscript.

EMBO Press is an editorially independent publishing platform for the development of EMBO scientific publications.

Best wishes,

William

Instructions for preparing your revised manuscript:

Please check that the title and abstract of the manuscript are brief, yet explicit, even to non-specialists.

When assembling figures, please refer to our figure preparation guideline in order to ensure proper formatting and readability in print as well as on screen:
https://bit.ly/EMBOPressFigurePreparationGuideline
See also figure legend guidelines: https://www.embopress.org/page/journal/14602075/authorguide#figureformat

IMPORTANT: When you send the revision we will require
- a point-by-point response to the referees' comments, with a detailed description of the changes made (as a word file).
- a word file of the manuscript text.
- individual production quality figure files (one file per figure)
- a complete author checklist, which you can download from our author guidelines
(https://www.embopress.org/page/journal/14602075/authorguide).
- Expanded View files (replacing Supplementary Information)
Please see out instructions to authors
https://www.embopress.org/page/journal/14602075/authorguide#expandedview

Please remember: Digital image enhancement is acceptable practice, as long as it accurately represents the original data and conforms to community standards. If a figure has been subjected to significant electronic manipulation, this must be noted in the figure legend or in the 'Materials and Methods' section. The editors reserve the right to request original versions of figures and the original images that were used to assemble the figure.

Further information is available in our Guide For Authors: https://www.embopress.org/page/journal/14602075/authorguide

We realize that it is difficult to revise to a specific deadline. In the interest of protecting the conceptual advance provided by the work, we recommend a revision within 3 months (27th Jul 2023). Please discuss the revision progress ahead of this time with the editor if you require more time to complete the revisions. Use the link below to submit your revision:

https://emboj.msubmit.net/cgi-bin/main.plex

Referee #2:

This revised manuscript entitled "RNF26 binds perinuclear Vimentin filaments to integrate ER and endolysosomal responses to proteotoxic stress " (previously entitled "Vimentin intermediate filaments organize organellar architecture in response to ER stress") by T. Cremer and collaborators describes the role of Vimentin intermediate filaments in the spatial organization of organelles such as endosomes and the endoplasmic reticulum (ER) and its role in proteostasis.

The authors followed the suggestions of the reviewers and now provide data that strengthen their conclusions, and therefore improve the manuscript.

The only point I want to discuss is the answer to my main point 3:
2. This reviewer is not convinced by the way data are analyzed in the manuscript. Most of the time (Fig. 2C, 2D, 2H, 2I, 3D, 3E, 4B, 4C, 4F, 4H, 5E and supplementary), for data obtained from image analyses, data from individual cells are compared and used to perform statistical analyses. The authors should use data from independent experiments (means) to perform statistical analyses.
Respectfully, to the best of our knowledge, consideration of individual cells as 'independent' in statistical terms is widely accepted, including in recent publications in the EMBO Journal (Bulthuis et al, 2023; Jongsma et al, 2020) when similar numbers of cells are analyzed between samples, and the exact number of cells plotted for each condition is indicated in the legend. In our view, plotting of individual data points provides an overview of the variation observed within a population of cells, which is hidden when means only are used.
I just want to cite a sentence from a paper published in The EMBO reports about this issue (Vault et al, . EMBO Reports (2012)13:291-296): "As biological experiments can be complicated, replicate measurements are often taken to monitor the performance of the experiment, but such replicates are not independent tests of the hypothesis, and so they cannot provide evidence of the reproducibility of the main results."
Plotting data points corresponding to individual cells does not preclude the use of means to perform the statistical analysis. There are visualization tools now that allow this, such as Superplots (Lord et al., JCB, 2020).
I leave this to the editor to decide, as it depends on the policy of the journal.

Minor points:

1. Line 556: words are missing
2. Line 818: what does the word unmodified means? Do you mean a wild type cell?

Dear Editor,

With this letter we would like to inform you of the resubmission of our manuscript entitled **"RNF26 binds perinuclear Vimentin filaments to integrate ER and endolysosomal responses to proteotoxic stress"** by Cremer et al (EMBOJ-2022-111252R-Q). We were pleased with the positive reception of our recently revised manuscript by you and the expert referees and have now exhaustively addressed the remaining editorial points regarding data reproducibility and textual ambiguities (stated per email communication to Jacques Neefjes). We sincerely hope our re-revised manuscript meets all requirements for publication in the *EMBO Journal* and look forward to the next steps in the process.

With Kind Regards,

Ilana Berlin and Jacques Neefjes

2nd Revision - Editorial Decision 21st Jun 2023

Dear Jacques, dear Ilana,

Firstly, sorry that the latest iteration of this manuscript has been with me for longer than it should have; I have been away from my desk recently. There are some very minor details for you to address before I can finally accept your work for publication. Firstly, there are some comments in the Word version of the file about, for example, defining error bars and stating statistical tests applied which remain open. Please go through this document carefully one more time and address all comments. In addition, please be sure to state explicitly in figure legends wherever the same image has been used (Figure 4 B&E and Figure EV3 F&G; Figure 1i and Figure 3 H; EV1E and Figure EV4D). Table EV1 is missing its legend above the table. Please remove the 'Supporting Information' section from the manuscript file. Please also include a valid email address for George Janssen. In figure EV4, maybe it would look cleaner if the 'n.d.' box and 'vimentin' label would be removed from the knockout (right-hand) panel?

Hannah Sonntag has contacted you separately about Source Data; please communicate with her directly about the most appropriate amount of unprocessed data to include in this context.

Best wishes,

William

William Teale, PhD
Editor
The EMBO Journal
w.teale@embojournal.org

Instructions for preparing your revised manuscript:

Please check that the title and abstract of the manuscript are brief, yet explicit, even to non-specialists.

When assembling figures, please refer to our figure preparation guideline in order to ensure proper formatting and readability in print as well as on screen:
https://bit.ly/EMBOPressFigurePreparationGuideline
See also figure legend guidelines: https://www.embopress.org/page/journal/14602075/authorguide#figureformat

IMPORTANT: When you send the revision we will require
- a point-by-point response to the referees' comments, with a detailed description of the changes made (as a word file).
- a word file of the manuscript text.
- individual production quality figure files (one file per figure)
- a complete author checklist, which you can download from our author guidelines (https://www.embopress.org/page/journal/14602075/authorguide).
- Expanded View files (replacing Supplementary Information)

Please see out instructions to authors
https://www.embopress.org/page/journal/14602075/authorguide#expandedview

Please remember: Digital image enhancement is acceptable practice, as long as it accurately represents the original data and conforms to community standards. If a figure has been subjected to significant electronic manipulation, this must be noted in the figure legend or in the 'Materials and Methods' section. The editors reserve the right to request original versions of figures and the original images that were used to assemble the figure.

Further information is available in our Guide For Authors: https://www.embopress.org/page/journal/14602075/authorguide

We realize that it is difficult to revise to a specific deadline. In the interest of protecting the conceptual advance provided by the work, we recommend a revision within 3 months (19th Sep 2023). Please discuss the revision progress ahead of this time with the editor if you require more time to complete the revisions. Use the link below to submit your revision:

https://emboj.msubmit.net/cgi-bin/main.plex

All editorial and formatting issues were resolved by the authors.

Dear Jacques and Ilana,

I am pleased to inform you that your manuscript has been accepted for publication in the EMBO Journal.

Congratulations! I am really glad to see this in The EMBO Journal - please also pass on my congratulations and best wishes to Tom.

Please note that it is EMBO Journal policy for the transcript of the editorial process (containing referee reports and your response letter) to be published as an online supplement to each paper. If you do NOT want this, you will need to inform the Editorial Office via email immediately. More information is available here: https://www.embopress.org/page/journal/14602075/authorguide#transparentprocess

Your manuscript will be processed for publication in the journal by EMBO Press. Manuscripts in the PDF and electronic editions of The EMBO Journal will be copy edited, and you will be provided with page proofs prior to publication. Please note that supplementary information is not included in the proofs.

You will be contacted by Wiley Author Services to complete licensing and payment information. The required 'Page Charges Authorization Form' is available here: https://www.embopress.org/pb-assets/embo-site/tej_apc.pdf - please download and complete the form and return to embopressproduction@wiley.com

EMBO Press participates in many Publish and Read agreements that allow authors to publish Open Access with reduced/no publication charges. Check your eligibility: https://authorservices.wiley.com/author-resources/Journal-Authors/open-access/affiliation-policies-payments/index.html

Should you be planning a Press Release on your article, please get in contact with embojournal@wiley.com as early as possible, in order to coordinate publication and release dates.

If you have any questions, please do not hesitate to call or email the Editorial Office. Thank you for your contribution to The EMBO Journal.

Yours sincerely,

William

William Teale, PhD
Editor
The EMBO Journal
w.teale@embojournal.org

** Click here to be directed to your login page: https://emboj.msubmit.net

EMBO Press Author Checklist

Corresponding Author Name: Jacques Neefjes	**USEFUL LINKS FOR COMPLETING THIS FORM**
Journal Submitted to: EMBO J	The EMBO Journal - Author Guidelines
Manuscript Number: 111252R-Q	EMBO Reports - Author Guidelines
	Molecular Systems Biology - Author Guidelines
	EMBO Molecular Medicine - Author Guidelines

Reporting Checklist for Life Science Articles (updated January

This checklist is adapted from Materials Design Analysis Reporting (MDAR) Checklist for Authors. MDAR establishes a minimum set of requirements in transparent reporting in the life sciences (see Statement of Task: 10.31222/osf.io/9sm4x). Please follow the journal's guidelines in preparing your

Please note that a copy of this checklist will be published alongside your article.

Abridged guidelines for figures

1. Data

The data shown in figures should satisfy the following conditions:

- the data were obtained and processed according to the field's best practice and are presented to reflect the results of the experiments in an accurate and unbiased manner.
- ideally, figure panels should include only measurements that are directly comparable to each other and obtained with the same assay.
- plots include clearly labeled error bars for independent experiments and sample sizes. Unless justified, error bars should not be shown for technical
- if n<5, the individual data points from each experiment should be plotted. Any statistical test employed should be justified.
- Source Data should be included to report the data underlying figures according to the guidelines set out in the authorship guidelines on Data

2. Captions

Each figure caption should contain the following information, for each panel where they are relevant:

- a specification of the experimental system investigated (eg cell line, species name).
- the assay(s) and method(s) used to carry out the reported observations and measurements.
- an explicit mention of the biological and chemical entity(ies) that are being measured.
- an explicit mention of the biological and chemical entity(ies) that are altered/varied/perturbed in a controlled manner.
- the exact sample size (n) for each experimental group/condition, given as a number, not a range;
- a description of the sample collection allowing the reader to understand whether the samples represent technical or biological replicates (including how many animals, litters, cultures, etc.).
- a statement of how many times the experiment shown was independently replicated in the laboratory.
- definitions of statistical methods and measures:
  - common tests, such as t-test (please specify whether paired vs. unpaired), simple χ2 tests, Wilcoxon and Mann-Whitney tests, can be unambiguously identified by name only, but more complex techniques should be described in the methods section;
  - are tests one-sided or two-sided?
  - are there adjustments for multiple comparisons?
  - exact statistical test results, e.g., P values = x but not P values < x;
  - definition of 'center values' as median or average;
  - definition of error bars as s.d. or s.e.m.

Please complete ALL of the questions below.
Select "Not Applicable" only when the requested information is not relevant for your study.

Materials

Newly Created Materials	Information included in the manuscript?	In which section is the information available? (Reagents and Tools Table, Materials and Methods, Figures, Data Availability Section)
New materials and reagents need to be available; do any restrictions apply?	Not Applicable	

Antibodies	Information included in the manuscript?	In which section is the information available? (Reagents and Tools Table, Materials and Methods, Figures, Data Availability Section)
For **antibodies** provide the following information:<br>- Commercial antibodies: RRID (if possible) or supplier name, catalogue number and or/clone number<br>- Non-commercial: RRID or citation	Yes	Materials and Methods

DNA and RNA sequences	Information included in the manuscript?	In which section is the information available? (Reagents and Tools Table, Materials and Methods, Figures, Data Availability Section)
**Short novel DNA or RNA including primers, probes**: provide the sequences.	Yes	Materials and Methods

Cell materials	Information included in the manuscript?	In which section is the information available? (Reagents and Tools Table, Materials and Methods, Figures, Data Availability Section)
**Cell lines:** Provide species information, strain. Provide accession number in repository **OR** supplier name, catalog number, clone number, and/**OR** RRID.	Yes	Materials and Methods
**Primary cultures:** Provide species, strain, sex of origin, genetic modification status.	Not Applicable	
Report if the cell lines were recently **authenticated** (e.g., by STR profiling) and tested for mycoplasma contamination.	Yes	Materials and Methods

Experimental animals	Information included in the manuscript?	In which section is the information available? (Reagents and Tools Table, Materials and Methods, Figures, Data Availability Section)
**Laboratory animals or Model organisms:** Provide species, strain, sex, age, genetic modification status. Provide accession number in repository **OR** supplier name, catalog number, clone number, **OR** RRID.	Not Applicable	
**Animal observed in or captured from the field:** Provide species, sex, and age where possible.	Not Applicable	
Please detail **housing and husbandry conditions**.	Not Applicable	

Plants and microbes	Information included in the manuscript?	In which section is the information available? (Reagents and Tools Table, Materials and Methods, Figures, Data Availability Section)
**Plants:** provide species and strain, ecotype and cultivar where relevant, unique accession number if available, and source (including location for collected wild specimens).	Not Applicable	
**Microbes:** provide species and strain, unique accession number if available, and source.	Not Applicable	

Human research participants	Information included in the manuscript?	In which section is the information available? (Reagents and Tools Table, Materials and Methods, Figures, Data Availability Section)
If collected and within the bounds of privacy constraints report on age, sex and gender or ethnicity for all study participants.	Not Applicable	

Core facilities	Information included in the manuscript?	In which section is the information available? (Reagents and Tools Table, Materials and Methods, Figures, Data Availability Section)
If your work benefited from core facilities, was their service mentioned in the acknowledgments section?	Yes	Listed as co-author

Design

Study protocol	Information included in the manuscript?	In which section is the information available? (Reagents and Tools Table, Materials and Methods, Figures, Data Availability Section)
If study protocol has been **pre-registered, provide DOI in the manuscript**. For clinical trials, provide the trial registration number **OR** cite DOI.	Not Applicable	
Report the **clinical trial registration number** (at ClinicalTrials.gov or equivalent), where applicable.	Not Applicable	

Laboratory protocol	Information included in the manuscript?	In which section is the information available? (Reagents and Tools Table, Materials and Methods, Figures, Data Availability Section)
Provide DOI OR other citation details if **external detailed step-by-step protocols** are available.	Not Applicable	

Experimental study design and statistics	Information included in the manuscript?	In which section is the information available? (Reagents and Tools Table, Materials and Methods, Figures, Data Availability Section)
Include a statement about **sample size** estimate even if no statistical methods were used.	Yes	Figure Legends - Statistical analysis
Were any steps taken to minimize the effects of subjective bias when allocating animals/samples to treatment (e.g. **randomization procedure**)? If yes, have they been described?	Not Applicable	
Include a statement about **blinding** even if no blinding was done.	Not Applicable	
Describe **inclusion/exclusion criteria** if samples or animals were excluded from the analysis. Were the criteria pre-established?<br><br>If sample or data points were omitted from analysis, report if this was due to attrition or intentional exclusion and provide justification.	Not Applicable	
For every figure, are **statistical tests** justified as appropriate? Do the data meet the assumptions of the tests (e.g., normal distribution)? Describe any methods used to assess it. Is there an estimate of variation within each group of data? Is the variance similar between the groups that are being statistically compared?	Yes	Figure legends, Materials and methods

Sample definition and in-laboratory replication	Information included in the manuscript?	In which section is the information available? (Reagents and Tools Table, Materials and Methods, Figures, Data Availability Section)
In the figure legends: state number of times the experiment was **replicated** in laboratory.	Yes	Legends
In the figure legends: define whether data describe **technical or biological replicates**.	Yes	Legends (as independent experiments)

Ethics

Ethics	Information included in the manuscript?	In which section is the information available? (Reagents and Tools Table, Materials and Methods, Figures, Data Availability Section)
Studies involving **human participants**: State details of **authority granting ethics approval** (IRB or equivalent committee(s), provide reference number for approval.	Not Applicable	
Studies involving **human participants**: Include a statement confirming that **informed consent** was obtained from all subjects and that the experiments conformed to the principles set out in the WMA Declaration of Helsinki and the Department of Health and Human Services Belmont Report.	Not Applicable	
Studies involving **human participants:** For publication of **patient photos**, include a statement confirming that consent to publish was obtained.	Not Applicable	
Studies involving experimental **animals**: State details of **authority granting ethics approval** (IRB or equivalent committee(s), provide reference number for approval. Include a statement of compliance with ethical regulations.	Not Applicable	
Studies involving **specimen and field samples:** State if relevant **permits** obtained, provide details of authority approving study; if none were required, explain why.	Not Applicable	

Dual Use Research of Concern (DURC)	Information included in the manuscript?	In which section is the information available? (Reagents and Tools Table, Materials and Methods, Figures, Data Availability Section)
Could your study fall under dual use research restrictions? Please check biosecurity documents and list of **select agents and toxins** (CDC): https://www.selectagents.gov/sat/list.htm	Not Applicable	
If you used a select agent, is the security level of the lab appropriate and reported in the manuscript?	Not Applicable	
If a study is subject to dual use research of concern regulations, is the name of the **authority granting approval and reference number** for the regulatory approval provided in the manuscript?	Not Applicable	

Reporting

The MDAR framework recommends adoption of discipline-specific guidelines, established and endorsed through community initiatives. Journals have their own policy about requiring specific guidelines and recommendations to complement MDAR.

Adherence to community standards	Information included in the manuscript?	In which section is the information available? (Reagents and Tools Table, Materials and Methods, Figures, Data Availability Section)
State if relevant guidelines or checklists (e.g., **ICMJE, MIBBI, ARRIVE, PRISMA**) have been followed or provided.	Not Applicable	
For **tumor marker prognostic studies,** we recommend that you follow the **REMARK** reporting guidelines (see link list at top right). See author guidelines, under 'Reporting Guidelines'. Please confirm you have followed these guidelines.	Not Applicable	
For **phase II and III randomized controlled trials**, please refer to the **CONSORT** flow diagram (see link list at top right) and submit the CONSORT checklist (see link list at top right) with your submission. See author guidelines, under 'Reporting Guidelines'. Please confirm you have submitted this list.	Not Applicable	

Data Availability

Data availability	Information included in the manuscript?	In which section is the information available? (Reagents and Tools Table, Materials and Methods, Figures, Data Availability Section)
Have **primary datasets** been deposited according to the journal's guidelines (see 'Data Deposition' section) and the respective accession numbers provided in the Data Availability Section?	Yes	Materials and Methods
Were **human clinical and genomic datasets** deposited in a public access-controlled repository in accordance to ethical obligations to the patients and to the applicable consent agreement?	Not Applicable	
Are **computational models** that are central and integral to a study available without restrictions in a machine-readable form? Were the relevant accession numbers or links provided?	Not Applicable	
If publicly available data were reused, provide the respective **data citations in the reference list**.	Not Applicable	
